# Dinuclear and tetranuclear group 10 metal complexes constructed from linear tetrasilane comprising both Si-H and Si-Si moieties

**DOI:** 10.1038/s42004-023-00892-8

**Published:** 2023-05-15

**Authors:** Yoshihiko Umehara, Ryosuke Usui, Yoshimasa Wada, Yusuke Sunada

**Affiliations:** 1 Department of Applied Chemistry, School of Engineering, The University of Tokyo, 4-6-1 Komaba, Meguro-ku, Tokyo, 153-8505 Japan; 2 Institute of Industrial Science, The University of Tokyo, 4-6-1 Komaba, Meguro-ku, Tokyo, 153-8505 Japan; 3 JST PRESTO, Honcho, Kawaguchi, Saitama, 332-0012 Japan

**Keywords:** Organometallic chemistry, Organometallic chemistry

## Abstract

The activation of Si-H bonds and/or Si-Si bonds in organosilicon compounds by transition-metal species plays a crucial role for the production of functional organosilicon compounds. Although group-10-metal species are frequently used to activate Si-H and/or Si-Si bonds, so far, systematic investigation to clarify the preferences of these metal species with respect to the activation of Si-H and/or Si-Si bonds remain elusive. Here, we report that platinum(0) species that bear isocyanide or *N*-heterocyclic-carbene (NHC) ligands selectively activates the terminal Si-H bonds of the linear tetrasilane Ph_2_(H)SiSiPh_2_SiPh_2_Si(H)Ph_2_ in a stepwise manner, whereby the Si-Si bonds remain intact. In contrast, analogous palladium(0) species are preferably inserted into the Si-Si bonds of the same linear tetrasilane, whereby the terminal Si-H bonds remain intact. Substitution of the terminal hydride groups in Ph_2_(H)SiSiPh_2_SiPh_2_Si(H)Ph_2_ with chloride groups leads to the insertion of platinum(0) isocyanide into all Si-Si bonds to afford an unprecedented zig-zag Pt_4_ cluster.

## Abstract (web summary)

Group 10 metal species are commonly used to activate Si–H and Si–Si bonds, but their preferred reactivity towards Si–H vs Si–Si activation is poorly understood. Here, Pt(0) species are shown to selectively activate the Si–H bonds in a linear tetrasilane, whereas Pd(0) preferentially activates the Si–Si bonds.

## Introduction

**Fig. 1:**
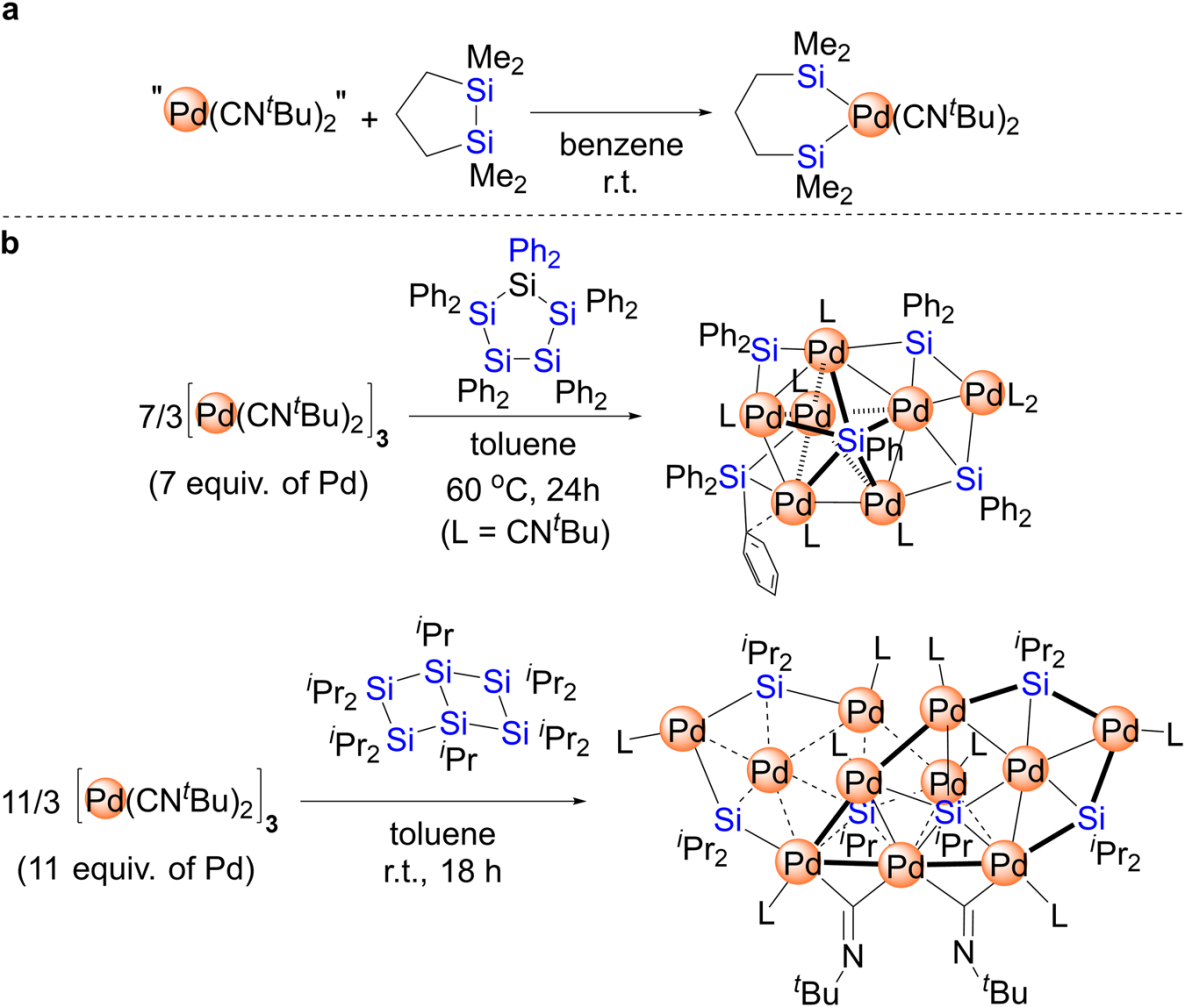
Previous examples of the activation of Si-Si bonds in organosilicon compounds by the reaction with palladium(0) isocyanide. **a** Ito’s work for the insertion of palladium species into a Si-Si bond^25, 26^. **b** Our previous work to synthesize the palladium clusters via sequential insertion of palladium species into multiple Si-Si bonds^32, 36^.

The activation of C-C bonds in hydrocarbons is arguably one of the most challenging issues in modern organometallic and organic chemistry^1–7^. It is well known that the activation of C-C bonds using transition-metal species is both kinetically and thermodynamically less favorable than the activation of C-H bonds. A similar analysis also holds for the silicon congeners of hydrocarbons, albeit that the activation of Si-Si^8–13^ or Si-H^14–16^ bonds is more commonly observed than that of C-C or C-H bonds. This difference in reactivity between Si-Si and Si-H bonds reflects the distinct bond-dissociation energies and the accessibility of the σ-orbitals of the substrates undergoing bond activation, and the bond dissociation energy of Si-H bond in the representative organosilicon compounds was reported to be ca. 85–100 kcal/mol, whereas that for Si-Si bond was described to be ca. 68 – 80 kcal/mol^17, 18^. Thus, a number of Si-H bond-transformation reactions, such as hydrosilylation, have been developed to produce useful organic silicon-containing compounds via the use of an appropriate transition metal^19–24^. Although only a small amount is known about the design of transition-metal compounds suitable for the activation of Si-Si bonds, certain low-valent late transition-metal species are known to facilitate the activation of Si-Si bonds. For instance, Ito et al. have described a palladium(0) bis(isocyanide) species, “Pd(CNR)_2_^”^, that exhibits good reactivity in Si-Si-bond-activation reactions to generate complexes of the type (RNC)_2_Pd(SiR_3_)_2_ (Fig. 1)^25–30^. Moreover, we have recently focused on the sequential insertion of “Pd(CNR)_2_^”^ species into multiple Si-Si bonds in oligosilanes with the aim of efficiently synthesizing palladium clusters. Based on this synthetic strategy, a series of palladium clusters consisting of Pd_3_, Pd_4_, Pd_5_, Pd_6_, Pd_7_, Pd_8_ and Pd_11_ skeletons was easily synthesized in one step via treatment of a “Pd(CNR)_2_^”^ species with the appropriate oligosilane or its derivatives^31–39^. For instance, reaction of [Pd(CN^*t*^Bu)_2_]_3_ with cyclopentasilane Si_5_Ph_10_ led to the formation of Pd_7_ cluster^36^, whereas Pd_11_ cluster was selectively obtained by the reaction of [Pd(CN^*t*^Bu)_2_]_3_ with bicyclic ladder polysilane^32^ (Fig. 1). These results indicate that the use of the appropriate metal precursor leads to the activation of comparatively less reactive Si-Si bonds. However, due to the lack of systematic investigations on the propensity of transition-metal species toward the activation of Si-Si and Si-H bonds, a reliable strategy for the on-demand design of reactive metal species that selectively facilitate the activation of Si-Si and/or Si-H bonds has not yet been established.

We hypothesized that a systematic investigation of the reactivity of low-valent group-10-metal species toward the activation of Si-Si and/or Si-H bonds could be conducted using oligosilanes that bear both Si-Si and Si-H bonds in a precursor molecule. Here, a series of zero-valent group-10-metal species that bear appropriate auxiliary ligands were chosen as the metal precursors, while a linear tetrasilane with two Si-H bonds at its termini, Ph_2_(H)SiSiPh_2_SiPh_2_Si(H)Ph_2_ (**1a**), was used as the oligosilane substrate. Herein, we report that the selective activation of either the Si-Si or Si-H bonds can be realized in a complementary manner depending on the group-10-metal species used. In particular, platinum(0) precursor bearing isocyanide or *N*-heterocyclic-carbene (NHC) ligands tend to activate the Si-H bonds in **1a** with all Si-Si bonds remain intact. In stark contrast, palladium(0) species combined with isocyanide or NHC ligands prefer to be inserted into Si-Si bond in **1a** to afford the dinuclear complexes. Furthermore, we found that when a linear tetrasilane without any Si-H bonds, such as Ph_2_(Cl)SiSiPh_2_SiPh_2_Si(Cl)Ph_2_ (**1b**), was subjected to the reaction with platinum(0) species, the scission of the Si-Si bond could be achieved using a platinum(0) isocyanide to afford a zig-zag Pt_4_ cluster, which indicates that platinum(0) species essentially prefer the activation of Si-H bonds over that of Si-Si bonds.

## Results and discussion

### Insertion of a platinum(0) species into the Si-H bonds of 1a where the Si-Si bonds remain intact

**Fig. 2:**
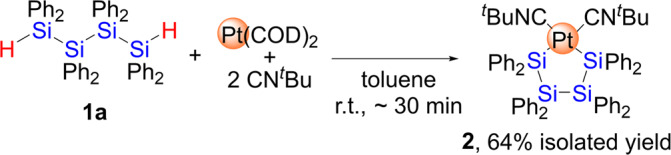
Reaction of **1a** with in-situ-generated “Pt(CN^*t*^Bu)_2_” to afford **2**. A mononuclear platinacyclic complex was formed in this reaction as a single product.

In our previous paper, we have reported that oligosilanes bearing Ph substituents on the Si atoms showed superior reactivity toward the reactions with low-valent metal precursors compared with that having alkyl substituents. For instance, cyclotetrasilane having Ph substituents, Si_4_Ph_8_, readily reacted with nickel(0) precursor to afford the dinuclear and pentanuclear nickel complexes, whereas no reaction took place when alkyl-substituted cyclotetrasilane such as Si_4_^*i*^Pr_8_ and Si_4_(cyclopentyl)_8_ were subjected in the reaction with nickel(0) species^39^. Thus, the linear oligosilane consisting of SiPh_2_ units was selected in this research, and the linear tetrasilanes Ph_2_(H)SiSiPh_2_SiPh_2_Si(H)Ph_2_ (**1a**) and Ph_2_(Cl)SiSiPh_2_SiPh_2_Si(Cl)Ph_2_ (**1b**) were synthesized according to a literature procedure^40^. In this paper, the reactivity of platinum(0) species toward the bond activation in **1a** was first examined. Even though the activation of Si-Si bonds by zero-valent platinum species that bear phosphine ligands has already been reported^41–43^, similar reactions of platinum(0) species that bear other auxiliary ligands have not been reported so far. Thus, the reactivity of platinum(0) precursor combined with isocyanide or NHCs ligand set was investigated, namely a platinum(0) species supported by either CN^*t*^Bu or ^*i*^PrIM^Me^ ligands was tested in the reaction with **1a**. The reaction of **1a** with 1 equiv. of Pt(COD)_2_ in the presence of 2 equiv. of CN^*t*^Bu smoothly furnished the mononuclear complex **2**, which exhibits a platinatetrasilacycle framework, in 64% isolated yield (Fig. 2). Complex **2** was found to be thermally stable even at 80 °C in C_6_D_6_ for 2 days. The three Si-Si bonds derived from **1a** remained intact, whereas the activation of the two terminal Si-H bonds occurred during the course of this reaction. It should be noted here that Mochida et al. have reported that the reaction of a platinum(0) species that bears phosphine ligands, (PPh_3_)_2_Pt(η^2^-ethylene), with Ph_2_(H)Ge(SiMe_2_)_n_Ge(H)Ph_2_ (*n* = 0–3) initially forms platinum-(germyl)(hydride) complexes via insertion of platinum species into one of the two Ge-H bonds, followed by the formation of a mononuclear platinacyclic complex of the type of (PPh_3_)_2_Pt(GePh_2_(SiMe_2_)_n_GePh_2_)^44^. Similarly, the formation of E-E (E = Si, Ge) bonds starting from (dppe)Pt[EAr_2_H]_2_ (dppe = 1,2-bis(diphenylphosphino)ethane, Ar = C_12_H_8_) to form (dppe)Pt(H)[(GeAr_2_)_3_GeAr_2_(H)] as an intermediary species, followed by the formation of mononuclear platinatetragermacyclic complex (PPh_3_)_2_Pt(GeAr_2_(GeAr_2_)_2_GeAr_2_) have been described by Braddock-Wilking and co-workers^45^. In contrast, the selective formation of **2** was confirmed by monitoring the reaction using ^1^H NMR spectroscopy, and no intermediary species, such as a platinum-(silyl)(hydride) complex generated by the activation of only one of the two Si-H moieties, was detected in this reaction. Moreover, further metalation did not take place when **1a** was treated with 4 equiv. of Pt(COD)_2_ and 8 equiv. of CN^*t*^Bu. The molecular structure, as determined by single-crystal XRD analysis, as well as the spectroscopic data are summarized in Supplementary Figs. 1, 2, 22 and 44, Supplementary Table 9 and Supplementary Data 1 and 2. In addition, a theoretical investigation based on a Wiberg bond index (WBI) analysis of **2** was described in Supplementary Fig. 34, Supplementary Table 3 and Supplementary Data 19.

**Fig. 3:**
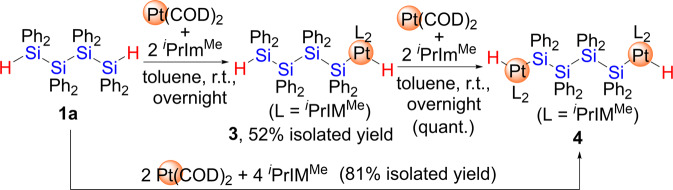
Sequential activation of the Si-H bonds of **1a** via reaction with Pt(COD)_2_/^*i*^PrIM^Me^ to afford **3** and **4**. One platinum species was inserted into one of two Si-H bonds in **1a** to form **3**, then second insertion of platinum species to the remaining Si-H bond followed to produce **4**.

**Fig. 4:**
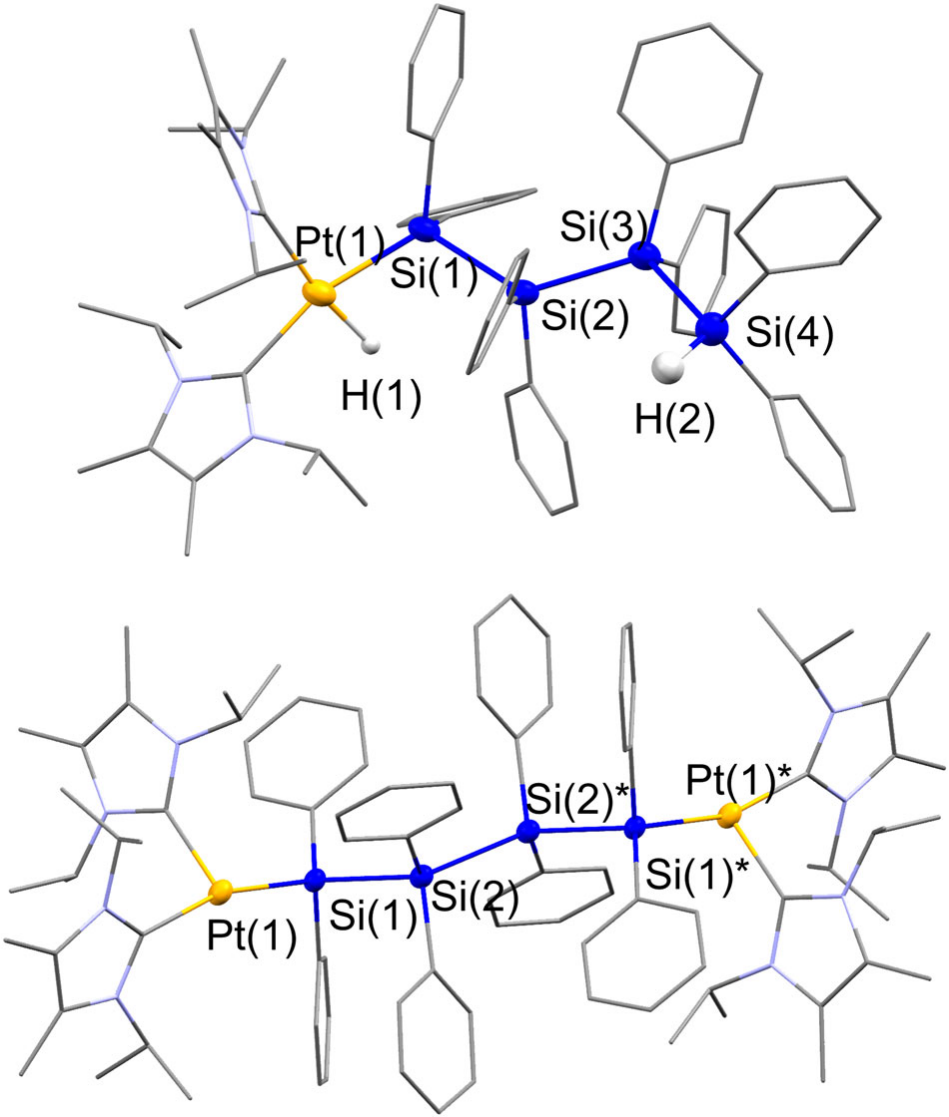
Molecular structures of **3** (upper) and **4** (lower) with thermal ellipsoids at 50% probability. All carbon atoms and nitrogen atoms are shown in capped-sticks style; all hydrogen atoms except for the H(1) and H(2) atoms in **3** are omitted for clarity.

In contrast, a sequential Si-H bond activation process was facilitated when the auxiliary ligand on the platinum center was changed from CN^*t*^Bu to ^*i*^PrIM^Me^ (^*i*^PrIM^Me^ = 1,3-diisopropyl-4,5-dimethylimidazol-2-ylidene). Treatment of **1a** with 1 equiv. of Pt(COD)_2_ and 2 equiv. of ^*i*^PrIM^Me^ led to the selective formation of complex **3** via the activation of only one of the two Si-H bonds. Complex **3** was isolated as a yellow powder in 52% yield (Fig. 3). The molecular structure, which was determined via single-crystal XRD analysis (Fig. 4, Supplementary Fig. 45, Supplementary Table 10 and Supplementary Data 3 and 4), revealed that one platinum atom supported by two *cis*-located ^*i*^PrIM^Me^ ligands was incorporated at the terminus of the linear tetrasilane skeleton, whilst the other Si-H moiety remained intact at the opposite terminus. The position of the hydrogen atom was found using a difference Fourier map, which suggested Pt-H and Pt-Si bond lengths of 1.55(6) Å and 2.3355(17) Å, respectively; these values are comparable to those found in mononuclear Pt(II) complexes that carry both silyl and hydride ligands^46–49^. The Si-Si bonds (Si(1)-Si(2): 2.402(2) Å; Si(2)-Si(3): 2.401(3) Å; Si(3)-Si(4): 2.393(2) Å) are slightly longer than those found in **1a**, albeit that they still fall in the range of normal Si-Si single bonds. In addition, the Si(4)-H(2) bond distance of 1.38(7) Å was identical to that found in **1a**. These structural parameters suggest that none of the Si-Si bonds nor the terminal Si-H group were affected upon complexation. In the ^1^H NMR spectrum, a singlet arising from the Pt-H moiety appears at −7.08 ppm along with a satellite signal due to the coupling (*J*_Pt-H_ = 736.1 Hz) with the ^195^Pt center whilst no apparent coupling with ^29^Si was observed. This suggests that no bonding interaction exists between the H(1) and Si(1) atoms. The remaining Si-H group appears at 5.44 ppm in the ^1^H NMR spectrum, and the ν_Si-H_ absorption band is found at 2035 cm^−1^ in the IR spectrum (Supplementary Figs. 3, 4 and 23).

It is noteworthy that the exclusive activation of both the Si-H bonds in **1a** took place when **1a** was treated with 2 equiv. of Pt(COD)_2_ and 4 equiv. of ^*i*^PrIM^Me^, giving the dinuclear complex **4** in 81% isolated yield (Fig. 3, Supplementary Figs. 5 and 24). Complex **4** was also accessible by treatment of **3** with a 1: 2 mixture of Pt(COD)_2_ and ^*i*^PrIM^Me^ via the activation of the remaining Si-H bond. The molecular structure of **4** was determined by a single-crystal XRD analysis and is shown in Fig. 4. The structure demonstrates that two Pt(H)(^*i*^PrIM^Me^)_2_ units are incorporated into the linear tetrasilane skeleton via a twofold Si-H activation. Unfortunately, the position of the hydrides could not be determined using XRD analysis, but the Pt(1)-Si(1) bond lengths in **4** (2.3352(18) Å) are comparable to that found in the parent complex **3**. In contrast, the Si-Si bonds in **4** (Si(1)-Si(2): 2.454(3) Å; Si(2)-Si(2)*: 2.445(3) Å) are slightly longer than those in **3**. This elongation might be induced by the introduction of the sterically demanding ^*i*^PrIM^Me^ ligand at both ends of the tetrasilane framework (Supplementary Fig. 46, Supplementary Table 11 and Supplementary Data 5 and 6).

That complexes **2,** **3**, and **4** can be formed via Si-H-bond activation suggests that zero-valent platinum species prefer to activate Si-H bonds compared to Si-Si bonds regardless of the auxiliary ligands on the platinum center. It is worth noting that Michalczyk and Fink et al. have reported that a platinum species supported by the bidentate phosphine, 1,2-bis(dicyclohexylphosphino)ethane (dcype), induces the activation of the Si-H bond of two disilanes, R(H)_2_Si-Si(H)_2_R (R = H, Me), to afford monohydride monosilyl complexes of the type (dcype)Pt(H){[Si(H)R]-Si(H)_2_R} as the primary product. This product is subsequently quickly converted into the bis(silyl) complex, (dcype)Pt[Si(H)_2_R]_2_, via activation of the Si-Si bond concomitant with the reformation of the Si-H bond^50^. Mochida and co-workers have reported similar reactivity for platinum species, which involves the activation of a Si-H bond followed by a heat-induced 1,2-silyl migration that involves a Si-Si activation/Si-H-bond-reforming process^48^. Considering these precedents, the possibility of a skeletal rearrangement in both **3** and **4** was investigated by heating their C_6_D_6_ solutions, which revealed that both complexes are thermally stable, even at 60 °C for 2 days, and that the activation of the Si-Si bonds is not promoted. Thus it was concluded that the monohydride monosilyl complexes **3** and **4** supported by ^*i*^PrIM^Me^ ligands are enough thermally stable, and they does not undergo the conversion into bis(silyl) complexes upon heating unlike the previous reports by Michalczyk and Fink et al. as well as Mochida et al. In addition, **4** was not susceptible to any further metalation, i.e., no reaction took place when **4** was treated with a 2: 4 mixture of Pt(COD)_2_/^*i*^PrIM^Me^. On the basis of these results, it can be concluded that platinum(0) species supported by isocyanide or NHC ligands preferably activate Si-H bonds over the Si-Si bonds in **1a**, a molecule that contains both Si-H and Si-Si bonds.

### Selective insertion of palladium(0) species into the Si-Si bonds of tetrasilane 1a

**Fig. 5:**
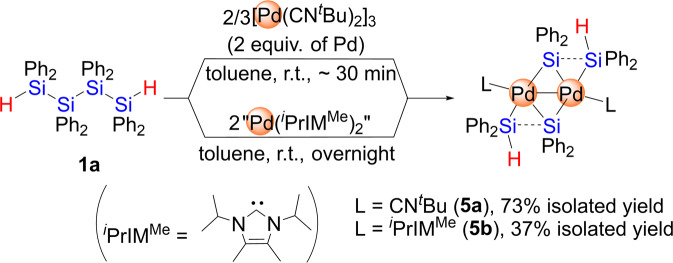
Pd(0)-induced activation of the inner Si-Si bond of **1a** to afford dinuclear complexes **5a** and **5b**. Dinuclear palladium complexes having icocyanide or NHC ligands were selectively formed.

**Fig. 6:**
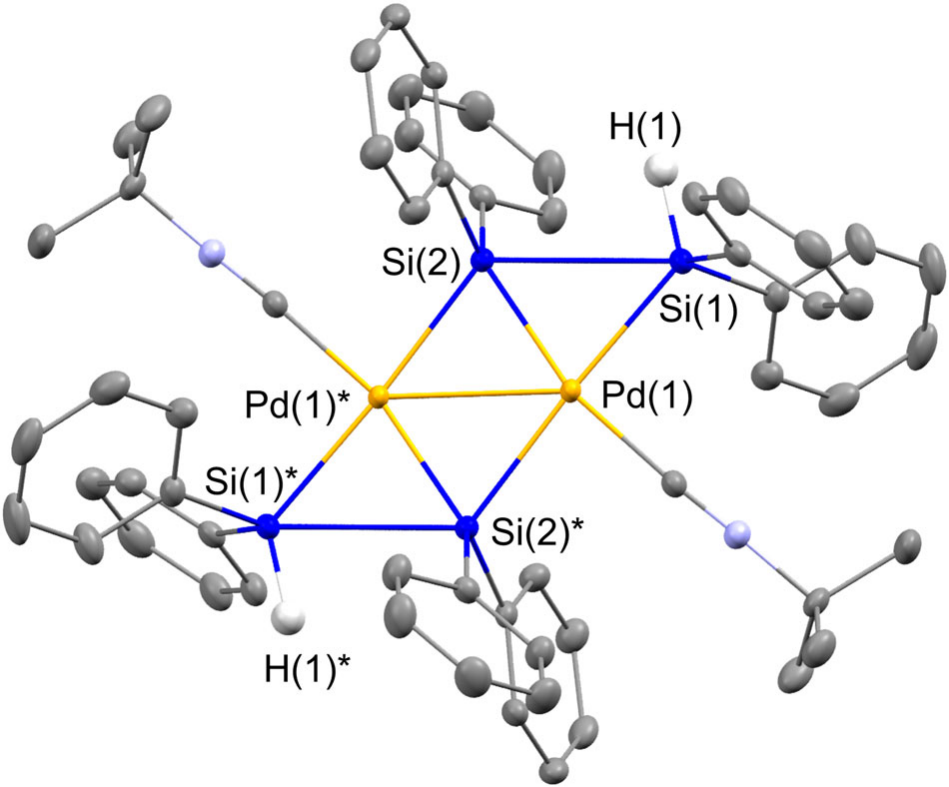
Molecular structure of **5a** with thermal ellipsoids at 50% probability. All hydrogen atoms, expect for H(1) and H(1)*, are omitted for clarity.

The reactivity of the platinum(0) species described in the section above clearly indicates that platinum(0) species preferably activate the Si-Si bond with either electron-donating or electron-withdrawing auxiliary ligands at the metal center. We recently discovered that the palladium(0) bis(isocyanide) [Pd(CN^*t*^Bu)_2_]_3_ exhibits high reactivity toward Si-Si-bond activation in several oligosilanes and thus, [Pd(CN^*t*^Bu)_2_]_3_ was then chosen as the palladium precursor. Thus, **1a** was treated with 2/3 equivalents of [Pd(CN^*t*^Bu)_2_]_3_ (2 equiv. of Pd relative to **1a**) in toluene at room temperature for 30 min, and the dinuclear complex **5a** was isolated in a 73% yield (Fig. 5). Although **5a** was thermally unstable and decomposed within 2 days, even at −20 °C, the molecular structure of **5a** was successfully determined via single-crystal X-ray diffraction (XRD) analysis (Fig. 6, Supplementary Fig. 47, Supplementary Table 12 and Supplementary Data 7 and 8).

There is an inversion center at the midpoint of the Pd-Pd axis in the molecular structure of **5a** and two palladium atoms were incorporated into the molecule via the activation of the inner Si-Si bond of **1a**. The bond distances of the two terminal Si-Si bonds (2.8324(4) Å) were significantly lengthened compared with those found in **1a** (2.3592(4) − 2.3661(6) Å). However, a theoretical investigation based on a WBI analysis suggested the presence of a bonding interaction between these two Si atoms (WBI Si(1)-Si(2) ≈ 0.40; for details, see Supplementary Figs. 35, 36, 37 and 38, Supplementary Tables 4 and 5, Supplementary Data 20 and 21). It should be noted here that the synthesis of a germanium analog of **5a** has been reported by Osakada et al.^51^ in which a similar elongation of the Ge-Ge bonds occurs whilst the bonding interaction is retained. Theoretical calculations indicated that the HOMO of **5a** is localized on the Si(1)-Si(2) bond as well as the Pd(1)-Si(2)* bond. This arrangement of MOs is also comparable to that found in the Ge analog of Osakada. The position of the two hydrogen atoms on the terminal Si-H moieties were determined using a difference Fourier map, which led to an Si(1)-H(1) bond length of 1.344(15) Å. This bond length, in conjunction with the WBI for Si-H ( ~ 0.88), indicates that no bonding interaction exists between the Pd center and the Si-H moieties. The relatively short palladium-palladium separation between Pd(1) and Pd(2) (2.7467(4) Å) suggests the presence of a weak bonding interaction, which is supported by the WBI value of ~0.18^52^. The Pd(1)-Si(1) bond length of 2.4050(5) Å is longer relative to that of Pd(1)-Si(2) (2.2907(5) Å), a finding that was also observed in the germanium congener synthesized by Osakada. The ^1^H, ^13^C, and ^29^Si NMR spectra, as well as the IR spectrum, are consistent with the solid-state structure of **5a** (Supplementary Figs. 6, 7, 8 and 25**)**; in the ^1^H NMR spectrum, a singlet assignable to the Si-H moiety appears at δ = 5.61 ppm, whilst one strong absorption band derived from the Si-H stretching vibration appears at 2055 cm^−1^ in the IR spectrum. The ^29^Si NMR spectrum shows two singlets at −13.44 and 210.44 ppm, and the former was attributed to a silicon atom bearing a hydrogen atom.

The high reactivity of a palladium(0) species that bear NHC ligands (“Pd(NHC)_2_”) toward the inactive Si-Si bond in disilanes has recently been demonstrated by Spencer and co-workers^53^. This report motivated us to employ “Pd(NHC)_2_” species in this study. Due to the strong σ-donating properties of NHCs, both the electronic and steric environment around the palladium center in “Pd(NHC)_2_” can be expected to be quite different to those in palladium(0) bis(isocyanide) complexes. However, we found that a “Pd(NHC)_2_” species was able to activate the Si-Si bonds in **1a** in a manner similar to the reaction with palladium(0) bis(isocyanide) complexes. Thus, “Pd(^*i*^PrIM^Me^)_2_”, which was generated in situ according to Spencer’s protocol, was treated with **1a** and the ^*i*^PrIM^Me^ analog of **5a** was isolated in a 37% yield (Fig. 5, Supplementary Figs. 9, 10, 11 and 12). Complex **5b** was also obtained in quantitative yield via a ligand exchange reaction of **5a** with 2 equiv. of ^*i*^PrIM^Me^ in toluene at room temperature. In contrast to **5a,** **5b** was found to be thermally stable, and no decomposition was observed by heating the C_6_D_6_ solution of **5b** at 80 °C for 2 days. The molecular structure of **5b** was determined via single-crystal XRD analysis (Supplementary Fig. 48, Supplementary Table 13 and Supplementary Data 9 and 10), which revealed that the Pd-Pd separation (2.8297(4) Å) is significantly longer than that in **5a**. A slightly decreased WBI value relative to that of **5a** was calculated for the Pd-Pd bond ( ~ 0.16) of **5b**, which is consistent with the XRD analysis. A similarly elongated Pd-Pd separation induced by σ electron-donating ligands has also been reported for Osakada’s dinuclear Ge complex bearing either CN^*t*^Bu or PMe_3_ ligands^51^. The Si(1)-Si(2) and Si(3)-Si(4) bond separations in **5b** (Si(1)-Si(2): 2.8602(8) Å; Si(3)-Si(4): 2.9323(8) Å) are elongated relative to those of **5a**. However, the WBI values for the Si-Si interactions in **5b** are comparable to those in **5a** (~0.40 for Si(1)-Si(2) and Si(3)-Si(4) in **5b**).

Signals assignable to a Si-H group were confirmed at *δ* = 5.40 ppm in the ^1^H NMR spectrum and at ν_Si-H_ = 2048 cm^−1^ in the IR spectrum of **5b** (Supplementary Figs. 9 and 26). The ^1^H NMR signals due to the ^*i*^PrIM^Me^ moieties and some of the Ph groups at the silicon centers broadened at room temperature. However, at –70 °C, these signals became sharp. For example, the ^1^H NMR spectrum of **5b** at room temperature showed two slightly broadened signals at 0.49 and 1.79 ppm along with an extremely broad signal at 0.22 ppm with an integral ratio of 12: 12: 12. At −70 °C, these signals sharpened into six doublets at *δ* = −0.17, 0.29, 0.47, 0.50, 1.54, and 1.73 ppm with an integral ratio of 6: 6: 6: 6: 6: 6 (Supplementary Figs. 10 and 11). These signals can be assigned to the Me groups of the ^*i*^Pr as well as the Me groups of the ^*i*^PrIM^Me^ ligand in **5b**, respectively. This dynamic behavior is presumably due to the distorted structure of **5b** induced by the sterically demanding ^*i*^PrIM^Me^ ligand. Specifically, in the molecular structure of **5a**, the two carbon atoms of the two CN^*t*^Bu ligands coordinated to the Pd atoms lie on a plane defined by two Pd and four Si atoms with a deviation of 0.004 Å, whereas the corresponding coordinated carbon atoms in **5b** significantly deviate from the Pd_2_Si_4_ plane with a deviation of 0.888 Å and 1.015 Å. This distortion makes the two ^*i*^Pr groups on the nitrogen atoms, as well as the two Me groups on the backbone of the ^*i*^PrIM^Me^ ligand, inequivalent in the solid state. The ^1^H NMR signals obtained at –70 °C are in good agreement with this solid-state structure. The restricted rotation about the Pd-C(^*i*^PrIM^Me^) bond induced by the two sterically demanding ^*i*^Pr groups could be the origin of the dynamic behavior observed in the variable-temperature NMR spectra.

**Fig. 7:**
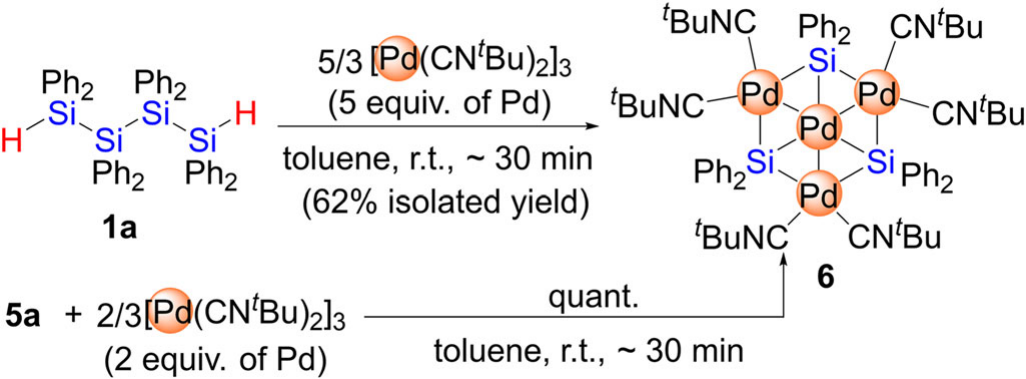
Synthesis of **6** via the reaction of **1a** with 5/3 equiv. of [Pd(CN^*t*^Bu)_2_]_3_ or via the reaction of **5a** with 2/3 equiv. of [Pd(CN^*t*^Bu)_2_]_3_. Construction of tetranuclear planar palladium cluster **6**.

The above results clearly indicate that the selective scission of the inner Si-Si bond in **1a** is favored when 2 equiv. of a palladium(0) precursor are used, affording the dinuclear complex **5** regardless of the auxiliary ligand that is present on the palladium center. Further metalation was found to be promoted when additional equivalents of the palladium(0) precursors were used. For example, treatment of **1a** with 5/3 equiv. of [Pd(CN^*t*^Bu)_2_]_3_ (5 equiv. of Pd relative to **1a**) in toluene at room temperature led to the formation of planar tetranuclear palladium cluster **6**, which was isolated in 62% yield (Fig. 7, Supplementary Figs. 13, 14, 15 and 27). Cluster **6** could also be selectively synthesized via the treatment of **5a** with 2/3 equiv. of [Pd(CN^*t*^Bu)_2_]_3_ (2 equiv. of Pd relative to **5a**) for 30 min at room temperature. Although the detailed reaction mechanism for the formation of **6** remains unclear at present, the generation of silylene (SiPh_2_) units via the successive cleavage of all the Si-Si bonds in **5a** might take place to form cluster **6**. A clue as to the fate of the two hydrogen atoms on the Si-H moieties in **5a** was obtained from the ^1^H NMR spectrum of the crude product, in which the selective production of **6** is accompanied by the generation of HSiPh_3_ in ~20% yield (based on H). It may be that H_2_SiPh_2_ is generated in situ via a Si-Si scission in **1a** induced by Pd(CN^*t*^Bu)_2_ followed by the redistribution of the silicon fragments involving a hydrogen migration to afford HSiPh_3_ and H_3_SiPh (the latter might be removed under vacuum). It was found that cluster **6** was gradually decomposed upon heating the C_6_D_6_ solution of **6** at 60 °C to give the complex mixture in the ^1^H NMR spectrum. Considering the fact that dinuclear palladium complex **5a** supported by CN^*t*^Bu ligands was also thermally unstable, thermal instability of **5a** and **6** presumably due to the dissociation of CN^*t*^Bu ligand from the Pd center(s).

Cluster **6** consists of a planar arrangement of four Pd and three Si atoms and the core Pd_4_Si_3_ framework is supported by the surrounding six CN^*t*^Bu ligands (Supplementary Fig. 49, Supplementary Table 14 and Supplementary Data 11 and 12). Cluster **6** possesses a three-fold axis of symmetry passing through the central Pd atom, Pd(2). It is noteworthy that Osakada et al. have reported the synthesis of an analogous palladium cluster supported by three bidentate phosphine ligands, whereby the structural parameters in **6**, including the Pd-Pd (2.7433(5) Å) and Pd-Si bonds (Pd(1)-Si(1): 2.5305(8); Pd(1)-Si(2): 2.5550(10) Å; Pd(2)-Si(1): 2.2760(8) Å), are comparable to those found in Osakada’s cluster^54–57^. Moreover, similar to Osakada’s cluster, **6** contains 58 cluster valence electrons, whereas we were able to synthesize analogous Pd_4_Si_3_ clusters with 54 cluster valence electrons by introducing more strongly electron-donating alkyl-substituted bridging silylene SiR_2_ (R = ^*i*^Pr, cyclopentyl) units instead of the SiPh_2_ moieties. The results of theoretical investigation including a WBI analysis of **6** were described in Supplementary Fig. 39, Supplementary Table 6 and Supplementary Data 22.

### Reaction of nickel(0)/isocyanide species with 1a to afford a dinuclear nickel complex

**Fig. 8:**
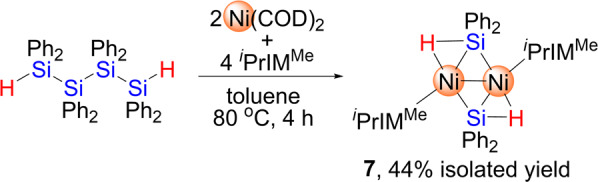
Synthesis of dinuclear nickel complex **7** via the reaction of **1a** with Ni(COD)_2_/^*i*^PrIM^Me^. Dinuclear nickel complex supported by two ^*i*^PrIM^Me^ ligands was formed as a single product.

We have recently reported that a nickel(0) isocyanide species generated in situ by mixing Ni(COD)_2_ and CN^*t*^Bu efficiently activates the Si-Si bonds in cyclotetrasilane, Si_4_Ph_8_, to afford a silylene-supported nickel cluster in conjunction with the activation of the C ≡ N bonds of the isocyanide ligands^37^. Thus, **1a** was treated with Ni(COD)_2_/CN^*t*^Bu, which generated only a complex mixture. However, dinuclear complex **7** was successfully isolated in 44% yield as pale yellow crystals when **1a** was treated with 2 equiv. of Ni(COD)_2_ in the presence of 4 equiv. of ^*i*^PrIM^Me^ (Fig. 8, Supplementary Figs. 16, 17, 18 and 28). Although the fate of the two “missing” SiPh_2_ units derived from the precursor **1a** remains unclear so far, **7** contains two μ-Si(H)Ph_2_ moieties, which might imply that nickel(0) species supported by ^*i*^PrIM^Me^ ligands preferably activate the Si-Si bonds in **1a** upon complexation. No decomposition was observed in the ^1^H NMR spectrum when C_6_D_6_ solution of **7** was heated at 80 °C for 2 days.

The molecular structure of **7** was determined using single-crystal XRD analysis (Supplementary Fig. 50, Supplementary Table 15 and Supplementary Data 13 and 14). This molecule contains a crystallographic inversion center at the midpoint of the Ni−Ni bond. The Ni(1)-Si(1) bond (2.2388(10) Å) is slightly longer than the Ni(1)-Si(1)* bond (2.1940(9) Å). These structural features, as well as the Ni(1)-H(1), Si(1)-H(1), and Ni(1)-Ni(1)* separations of 1.59(3) Å, 1.70(4) Å, and 2.5112(9) Å, respectively, are comparable to those found in the analogous complex, {(IPr)Ni[μ-Si(H)Ph_2_]}_2_ (IPr = 1,3-di(2,6-di-iso-propylphenyl)imidazolin-2-ylidene)^58–61^. The WBI for the Ni-Ni separation ( ~ 0.26) suggests the presence of a weak bonding interaction (Supplementary Figs. 40 and 41, Supplementary Table 7, Supplementary Data 23). The Si(1)-H(1) bond is significantly longer than those in **1a** (1.38(2) Å), indicating that the Si-H moieties are somewhat activated by back donation from the Ni center. The WBI values for Si(1)-H(1) ( ~ 0.55) and Ni(1)-H(1) ( ~ 0.30), respectively, support this conclusion. The bridging hydrogen atoms (H(1) and H(1)*) are arranged almost coplanar with the [Ni_2_Si_2_] core fragment with a deviation from the [Ni_2_Si_2_] plane of 0.129 Å. The presence of bridging hydrogen atoms between Ni(1) and Si(1) is also supported by the appearance of a singlet at *δ* = − 0.96 ppm in the ^1^H NMR spectrum (Supplementary Fig. 16). This signal is significantly high-field shifted, which is indicative of the presence of back donation from the Ni center to the Si-H moiety. This is consistent with the decreased WBI value of 0.55 for Si(1)-H(1), suggesting that the Si-H bond is slightly activated by back donation from the nickel center.

### Insertion of a platinum(0) isocyanide species into the Si-Si bonds of 1b to afford a zig-zag Pt_4_ cluster

The results in the former section above clearly demonstrate that the activation of the Si-H bonds predominates over the activation of the Si-Si bonds when using a platinum(0) species as the metal precursor. Theoretical calculations on **1a** and **1b**, an analog of **1a** bearing a Cl atom at its termini instead of an H atom, showed that their HOMOs and LUMOs are located at similar energy levels (Supplementary Figs. 30, 31, 32 and 33, Supplementary Tables 1 and 2, Supplementary Data 17 and 18). The energy levels of the HOMO and LUMO of **1a** were estimated to be −5.82 eV and −0.94 eV, respectively, whereas those of **1b** were calculated to be −6.02 eV and −1.06 eV, respectively. In addition, the molecular orbitals that constitute the HOMO and LUMO of **1a** and **1b** are also fairly comparable; the HOMO of **1a** and **1b** mainly comes from the σ-bonding interactions between the Si-Si-Si-Si bonds, whereas the main contribution to the LUMO of **1a** and **1b** comes from the σ*-bonding interaction between the Si-Si-Si-Si bonds. Although the reported Si-Si bond lengths in **1b** determined from the single-crystal XRD analysis are slightly longer than those in **1a** (**1a**: 2.3592(4) - 2.3661(6) Å; **1b**: 2.374(2) - 2.389(2) Å), the electronic as well as structural similarity of **1a** and **1b** prompted us to investigate the reaction of linear tetrasilane **1b**, instead of **1a**, with platinum(0) isocyanide to gain further insight into the bond-activation performance of such platinum(0) species.

**Fig. 9:**
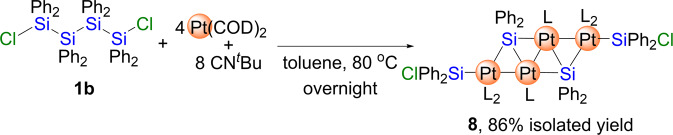
Activation of the Si–Si bonds in **1b** by Pt(COD)_2_/CN^*t*^Bu. Selective formation of a tetranuclear Pt cluster **8** with a ladder-type Pt_4_Si_2_ framework.

**Fig. 10:**
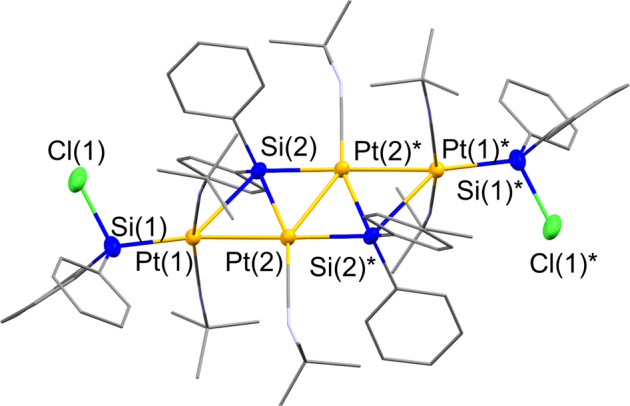
Molecular structure of **8** with thermal ellipsoids at 50% probability. All carbon atoms and nitrogen atoms are shown in capped-sticks style; all hydrogen atoms are omitted for clarity.

Thus, **1b** was treated with 4 equiv. of Pt(COD)_2_ and 8 equiv. of CN^*t*^Bu in toluene at 80 °C, which led to the selective formation of tetranuclear platinum cluster **8** as indicated by the ^1^H NMR spectrum of the crude product. Cluster **8** was isolated in 86% yield (Fig. 9). It is noteworthy that no reaction took place at room temperature, which stands in contrast to the reaction of **1a** with the Pt(COD)_2_/CN^*t*^Bu system. Moreover, the reaction of **1b** with a decreased amount of Pt(COD)_2_/CN^*t*^Bu at 80 °C also afforded **8** as a single product concomitant with the recovery of starting **1b**. The isolated complex **8** was thermally stable at 80 °C in C_6_D_6_ for 2 days. The molecular structure of **8**, determined via single-crystal XRD analysis, is characterized by four platinum atoms arranged in a zig-zag form, which is consistent with the cleavage of all the Si-Si bonds in **1b** (Fig. 10, Supplementary Fig. 51, Supplementary Table 16 and Supplementary Data 15 and 16). The two terminal Si-Cl bonds were found to remain intact in this reaction. Thus, it may be that platinum(0) species supported by isocyanides activate Si-Si bonds at higher reaction temperatures when no Si-H bonds are present in the oligosilane. Considering the structural similarity of **5a** and **8**, it may be that the zig-zag tetranuclear metal cluster is formed from a dinuclear skeleton similar to that found in **5a** via the incorporation of two additional metal species into the remaining two Si-Si bonds of the dinuclear skeleton.

The unique molecular structure of **8**, which was also determined via a single-crystal XRD analysis (Fig. 10), showed that **8** contains an unprecedented ladder-type Pt_4_Si_2_ framework with four platinum atoms and two bridging silylene moieties. Pt_4_Si_2_ is a highly planar framework, wherein all atoms deviate from the Pt_4_Si_2_ plane by 0.008-0.016 Å. The coordination geometry around Pt(1) and Pt(1)* can be regarded as square planar with a Si(Cl)Ph_2_ ligand, two CN^*t*^Bu ligands, and a centroid of the Pt(2)-Si(2) or Pt(2)*-Si(2)* bond. The square-planar coordination environment around the Pt(1) or Pt(1)* atom is almost orthogonally arranged relative to the ladder-type Pt_4_Si_2_ plane (86.78 ^o^). The two silicon atoms of the terminal –Si(Cl)Ph_2_ moieties also lie close to this plane with a deviation of 0.256 Å. The four platinum atoms are arranged in an unprecedented zig-zag fashion, and the inner Pt(2)-Pt(2)* bond (2.7233(5) Å) is slightly longer than that between the Pt atoms located at the edges (Pt(1)-Pt(2) = 2.7058(4) Å). The Pt(2)-Pt(2)* bond is still short enough to invoke a metal-metal bonding interaction^62–65^. This is supported by the WBIs, estimated from theoretical calculations, for Pt(1)-Pt(2) (0.31) and Pt(2)-Pt(2)* (0.23) (Supplementary Figs. 42 and 43, Supplementary Table 8, Supplementary Data 24). The bond separation of the two silylene units symmetrically bridged over the two platinum atoms are almost identical (Pt(1)-Si(2) = 2.3496(11) Å; Pt(2)*-Si(2) = 2.3693(10) Å).

The single-crystal XRD analysis is in good agreement with the solution-state NMR spectrum (Supplementary Figs. 19, 20, 21 and 29), indicating that the ladder-type Pt_4_Si_2_ framework of **8** is maintained in solution. In the ^1^H NMR spectrum of **8** in C_6_D_6_, two singlets due to the coordinated CN^*t*^Bu ligands appear at *δ* = 0.78 and 0.84 ppm with an integral ratio of 36: 18. The latter signal was assigned to the two CN^*t*^Bu ligands coordinated to the inner two Pt centers (Pt(2) and Pt(2)*). The bridging SiPh_2_ moieties appear at 158.22 ppm as a singlet in the ^29^Si NMR spectrum of **8**, whereas a singlet at *δ* = 64.68 ppm was assigned to the terminal Si(Cl)Ph_2_ moieties.

## Conclusions

In conclusion, we found that different zero-valent group-10-metal species are able to facilitate the complementary activation of Si-H and/or Si-Si bonds despite the same auxiliary ligands being introduced onto the metal species. Namely, platinum(0) species selectively activate the Si-H bonds in **1a** regardless of the introduced auxiliary ligands. Specifically, a platinum(0) species combined with an *N*-heterocyclic-carbene ligand sequentially activated the two Si-H bonds in **1a**, whereby all Si-Si bonds remaining intact. Mononuclear complex **3** is formed as the primary product followed by the incorporation of a second platinum fragment into **3** to afford dinuclear complex **4**. In contrast, palladium(0) species tend to be selectively inserted into the Si-Si bonds rather than the Si-H bonds of tetrasilane **1a**. Thus, an internal Si-Si bond in **1a** was activated preferentially to afford the dinuclear palladium complex **5** when **1a** was treated with palladium(0) precursor combined with isocyanide or *N*-heterocyclic carbene ligands. Then, further subsequent activation of the Si-Si bonds followed to form a planar tetranuclear cluster **6** as the final product. A similar tendency to favor the activation of the Si-Si bonds in **1a** was observed in the reaction of a nickel(0) species with **1a**. Despite the exclusive affinity of platinum(0) isocyanide for the activation of Si-H bonds, Si-Si bond cleavage with platinum(0) isocyanide can also be realized when a linear tetrasilane that bears two Si-Cl moieties instead of Si-H units, Ph_2_(Cl)SiSiPh_2_SiPh_2_Si(Cl)Ph_2_ (**1b**), is used as the substrate. The resulting reaction furnished a tetranuclear platinum cluster **8** in which the four platinum atoms are arranged in a zig-zag fashion. The investigation of the reactivity of a series of zero-valent group-10-metal species described in this paper clearly indicate that palladium(0) and nickel(0) species prefer the activation of Si-Si bonds, whereas platinum(0) precursors tend to cleave the Si-H bonds prior to Si-Si bonds. The results shown in this paper contributes to the better understanding of the fundamental reactivity of each metal species with respect to bond activation. On the basis of these findings, efforts to develop new transformations of organosilicon compounds with the aid of group-10-metal catalysts are currently in progress in our laboratories.

## Methods

General information was described in Supplementary Note 1 and Computational Details as well as details for X-ray data collection and reduction were described in Supplementary Notes 2 and 3, respectively.

### Synthesis of 2

In a 50 mL schlenk tube, Ph_2_(H)Si-SiPh_2_SiPh_2_Si(H)Ph_2_ (73 mg, 0.10 mmol) was dissolved in toluene (10 mL), then toluene (5 mL) solution of [Pd(CN^*t*^Bu)_2_]_3_ (41 mg, 0.05 mmol) was added to this solution at room temperature. The solution was stirred at room temperature for 30 min. The solvent was kept at −20 °C for overnight, from which yellow crystals of **2** was obtained in 64% yield (70 mg, 0.064 mmol). ^1^H NMR (400 MHz, r.t., C_6_D_6_): *δ* = 0.60 (s, 18H, ^*t*^Bu), 6.95–7.03 (m, 12 H, Ph), 7.07–7.12 (m, 12H, Ph), 7.59–7.61 (m, 8H, Ph), 7.93–7.96 (m, 8H, Ph). ^13^C NMR (100 MHz, r.t., C_6_D_6_): 28.73 (s, C(*C*H_3_)_3_), 56.75 (s, *C*(CH_3_)_3_), 126.81, 127.02, 127.26, 137.39 (s, *J*_Pt-C_ = 22.0 Hz), 137.85, 138.29 (*J*_Pt-C_ = 28.7 Hz), 144.39 (*J*_Pt-C_ = 15.3 Hz). ^29^Si NMR (119 MHz, r.t., C_6_D_6_): no signal was detectable due to the low solubility of **2** toward C_6_D_6_. IR (ATR): ν_CN_ = 2192, 2170 cm^−1^. Anal calcd for C_58_H_58_N_2_Si_4_Pt; C 63.88, H 5.36, N: 2.57; found: C 63.41, H 5.81, N: 2.71.

### Synthesis of 3

In a 50 mL schlenk tube, Ph_2_(H)Si-SiPh_2_SiPh_2_Si(H)Ph_2_ (73 mg, 0.10 mmol) was dissolved in toluene (20 mL), then toluene (10 mL) solution of the mixture of Pt(COD)_2_ (41 mg, 0.10 mmol) and ^*i*^PrIM^Me^ (36 mg, 0.20 mmol) was added to this solution at room temperature. The solution was stirred at room temperature for overnight. The solvent was removed *in vacuo*, and the obtained solid was washed with Et_2_O to afford **3** as yellow powder in 52% yield (67 mg, 0.052 mmol). Pale yellow crystals suitable for a single crystal X-ray diffratction analysis was obtained by recrystallization from THF at −20 °C. ^1^H NMR (400 MHz, r.t., C_6_D_6_): δ = −7.08 (s, *J*_Pt-H_ = 736.1 Hz, 2H, Pt-H), 0.77 (d, 6H, *J*_H-H_ = 7.3 Hz, CH(C*H*_3_)_2_), 0.85 (d, 6H, *J*_H-H_ = 7.3 Hz, CH(C*H*_3_)_2_), 0.90 (d, 12H, *J*_H-H_ = 7.3 Hz, CH(C*H*_3_)_2_), 1.64 (s, 6H, C = C-Me), 1.69 (s, 6H, C = C-Me), 5.44 (s, Si-H), 5.49 (sept, *J*_H-H_ = 7.3 Hz, 1H, C*H*(CH_3_)_2_), 5.66 (sept, *J*_H-H_ = 7.3 Hz, 1H, C*H*(CH_3_)_2_), 7.10–7.21 (m, 24 H, Ph), 7.67–7.69 (m, 8H, Ph), 7.78–7.80 (m, 8H, Ph). ^13^C NMR (100 MHz, r.t., C_6_D_6_): 9.97(s, CH(*C*H_3_)_2_), 10.13(s, CH(*C*H_3_)_2_), 20.78, 21.22, 21.36, 52.31 (s, *C*H(CH_3_)_2_), 52.52 (s, *C*H(CH_3_)_2_), 123.79, 125.43, 126.10, 126.33, 126.79, 127.02, 127.43, 128.29, 128.36, 129.06, 135.83, 136.70, 137.01, 137.80, 138.55, 138.63, 139.80, 146.41,186.99 (Pt-C), 187.50 (Pt-C). ^29^Si NMR (119 MHz, r.t., C_6_D_6_): no signal was detectable due to the low solubility of **3** toward C_6_D_6_. IR (ATR): ν_SiH_ = 2035 cm^−1^. Anal calcd for C_70_H_82_N_4_Si_4_Pt; C 65.33, H 6.42, N: 4.35; found: C 65.35, H 6.87, N: 4.32.

### Synthesis of 4

In a 50 mL schlenk tube, Ph_2_(H)Si-SiPh_2_SiPh_2_Si(H)Ph_2_ (73 mg, 0.10 mmol) was dissolved in THF (10 mL), then toluene (10 mL) solution of Pt(COD)_2_ (82 mg, 0.20 mmol) and ^*i*^PrIM^Me^ (72 mg, 0.40 mmol) was added to this solution at room temperature. The solution was stirred at room temperature for overnight. The solvent was removed *in vacuo*, and the obtained solid was washed with Et_2_O to afford **4** as yellow powder in 81% yield (149 mg, 0.081 mmol). Pale yellow crystals suitable for a single crystal X-ray diffraction analysis was obtained by recrystallization from THF at −20 °C. ^1^H NMR (400 MHz, r.t., C_6_D_6_): *δ* = −7.20 (s, *J*_Pt-H_ = 747.1 Hz, 2H, Pt-H), 0.77 (d, 12H, *J*_H-H_ = 7.3 Hz, CH(CH_3_)_2_), 0.85 (d, 12H, *J*_H-H_ = 7.3 Hz, CH(CH_3_)_2_), 0.87 (d, 24H, *J*_H-H_ = 7.3 Hz, CH(CH_3_)_2_), 1.65 (s, 12H, C = C-Me), 1.67 (s, 12H, C = C-Me), 5.36-5.44 (sept, *J*_H-H_ = 7.3 Hz, 4H, CH(CH_3_)_2_), 5.62-5.69 (sept, *J*_H-H_ = 7.3 Hz, 4H, CH(CH_3_)_2_), 6.97-7.15 (m, 24 H, Ph), 7.36-7.38 (m, 4H, Ph), 7.47-7.50 (m, 4H, Ph), 7.85-7.89 (m, 8H, Ph). ^13^C and ^29^Si NMR measurements were not successful due to the low solubility of **4** toward C_6_D_6_. IR (ATR): ν_SiH_ = 2050 cm^−1^. Anal calcd for C_92_H_122_N_8_Si_4_Pt_2_; C 59.97, H 6.67, N: 6.08; found: C 59.92, H 6.28, N: 5.58.

### Synthesis of 5a

In a 50 mL schlenk tube, Ph_2_(H)SiSiPh_2_SiPh_2_Si(H)Ph_2_ (116 mg, 0.16 mmol) was dissolved in toluene (20 mL), then toluene (10 mL) solution of [Pd(CN^*t*^Bu)_2_]_3_ (109 mg, 0.13 mmol) was added to this solution at room temperature. The solution was stirred at room temperature for 30 min. The solvent was removed *in vacuo*, and the obtained solid was washed with Et_2_O to afford **5a** as yellow powder in 72% yield (133 mg, 0.12 mmol). Crystals suitable for a single crystal X-ray diffraction analysis was obtained by recrystallization from toluene at −20 °C. ^1^H NMR (400 MHz, r.t., C_6_D_6_): ^1^H NMR (400 MHz, r.t., C_6_D_6_): δ = 0.61 (s, 18H, ^*t*^Bu), 5.61 (s, 2H, Si-H), 7.03–7.05 (m, 12 H, Ph), 7.13–7.19 (m, 14H, Ph), 7.58-7.60 (m, 8H, Ph), 8.01-8.03 (m, 6 H, Ph). ^13^C NMR (100 MHz, r.t., C_6_D_6_): 29.31 (s, C(*C*H_3_)_3_), 56.72 (s, *C*(CH_3_)_3_), 127.44 (s, Ph), 127.61 (s, Ph), 127.77 (s, Ph), 129.05 (s, Ph), 137.01 (s, Ph), 137.30 (s, Ph), 141.72 (s, Ph), 143.30 (s, Ph), 150.33 (s, *C*N^*t*^Bu). ^29^Si NMR (119 MHz, r.t., C_6_D_6_): −13.44 (s), 210.44 (s). IR (ATR): ν_CN_ = 2167 cm^−1^, ν_SiH_ = 2055 cm^−1^. Anal calcd for C_58_H_60_N_2_Si_4_Pd_2_; C 62.74, H 5.45, N: 2.52; found: C 62.78, H 4.96, N 2.70.

### Synthesis of 5b

In a 50 mL schlenk tube, Ph_2_(H)Si-SiPh_2_SiPh_2_Si(H)Ph_2_ (37 mg, 0.05 mmol) was dissolved in toluene (10 mL), then toluene (5 mL) solution of “Pd(^*i*^PrIM^Me^)_2_” (prepared in situ from the reaction of [(η^3^-methallyl)PdCl]_2_ (20 mg, 0.05 mmol), ^*i*^PrIM^Me^ (37 mg, 0.21 mmol), KO^*t*^Bu (11 mg, 0.10 mmol) and 2-propanol (7.6 μL, 0.10 mmol)) was added to this solution at room temperature. The solution was stirred at room temperature for overnight. The solvent was removed *in vacuo*, and the obtained solid was washed with Et_2_O to afford **5b** as yellow powder in 37% yield (48 mg, 0.037 mmol). Crystals suitable for a single crystal X-ray diffraction analysis was obtained by recrystallization from toluene at −20 °C. ^1^H NMR (400 MHz, r.t., C_6_D_6_): *δ* = 0.22 (br, 12H, CH(C*H*_3_)_2_), 0.49 (br d, *J* = 6.9 Hz, 12H, CH(C*H*_3_)_2_), 1.72 (br s, 12H, C=CMe), 4.51 (br m, 4H, C*H*(CH_3_)_2_), 5.40 (s, 2H, Si-H), 7.07–7.09 (m, 12 H, Ph), 7.12–7.24 (m, 14H, Ph), 7.51-7.54 (m, 6H, Ph), 7.99 (br m, 8H, Ph). ^1^H NMR (400 MHz, −70 °C, C_7_D_8_): *δ* = −0.17 (d, *J* = 7.3 Hz, 6H, CH(C*H*_3_)_2_), 0.29 (d, *J* = 7.3 Hz, 6H, CH(C*H*_3_)_2_), 0.47 (d, *J* = 7.3 Hz, 6H, CH(C*H*_3_)_2_), 0.50 (d, *J* = 7.3 Hz, 6H, CH(C*H*_3_)_2_), 1.54 (s, 6H, C=CMe), 1.73 (s, 6H, C=CMe), 4.28 (sept, *J* = 7.3 Hz, 2H, C*H*(CH_3_)_2_), 4.61 (sept, *J* = 7.3 Hz, 2H, C*H*(CH_3_)_2_), 5.40 (s, 2H, Si-H), 7.07–7.09 (m, 26 H, Ph), 7.41–7.48 (m, 4H, Ph), 7.56-7.61 (m, 2H, Ph), 7.63–7.69 (m, 4H, Ph), 8.06-8.13 (m, 2H, Ph), 8.80-8.87 (m, 2H, Ph). ^13^C NMR (100 MHz, r.t., C_6_D_6_): 10.19 (s, CH(*C*H_3_)_2_), 20.48, 21.08, 54.11 (s, *C*H(CH_3_)_2_), 127.16, 127.43, 137.87, 144.02, 146.83, 184.04 (Pd-C). ^29^Si NMR (119 MHz, r.t., C_6_D_6_): no signal was detectable due to the limited solubility of **5b** toward C_6_D_6_. IR (ATR): ν_SiH_ = 2048 cm^−1^. Anal calcd for C_70_H_82_N_4_Si_4_Pd_2_; C 64.45, H 6.34, N: 4.29; found: C 64.57, H 6.39, N: 4.29.

### Synthesis of 6

In a 50 mL schlenk tube, Ph_2_(H)Si-SiPh_2_SiPh_2_Si(H)Ph_2_ (88 mg, 0.12 mmol) was dissolved in toluene (20 mL), then toluene (10 mL) solution of [Pd(CN^*t*^Bu)_2_]_3_ (174 mg, 0.21 mmol) was added to this solution at room temperature. The solution was stirred at room temperature for 30 min. The solvent was kept at −20 °C for overnight, from which yellow crystals of **6** was obtained in 62% yield (148 mg, 0.10 mmol). ^1^H NMR (400 MHz, r.t., C_6_D_6_): δ = 0.86 (s, 54H, ^*t*^Bu), 7.09-7.13 (m, 6 H, Ph), 7.23-7.27 (m, 12H, Ph), 8.20-8.22 (m, 12H, Ph). ^13^C NMR (100 MHz, r.t., C_6_D_6_): 29.59 (s, C(*C*H_3_)_3_), 54.50 (s, *C*(CH_3_)_3_), 125.04, 126.10, 152.09, 152.55. ^29^Si NMR (119 MHz, r.t., C_6_D_6_): 184.80 (s). IR (ATR): ν_CN_ = 2138 cm^−1^. Anal calcd for C_66_H_84_N_8_Si_4_Pd_6_; C 53.88, H 5.75, N: 5.71; found: C 54.31, H 5.30, N: 5.18.

### Synthesis of 7

In a 50 mL schlenk tube, Ph_2_(H)Si-SiPh_2_SiPh_2_Si(H)Ph_2_ (146 mg, 0.05 mmol) was dissolved in toluene (20 mL), then toluene (10 mL) solution of the mixture of Ni(COD)_2_ (109 mg, 0.13 mmol) and ^*i*^PrIM^Me^ (109 mg, 0.13 mmol) was added to this solution at room temperature. The solution was stirred at 80 °C for overnight. The solvent was removed *in vacuo*, and the obtained solid was washed with Et_2_O to afford **7** as red powder in 44% yield (74 mg, 0.088 mmol). Red crystals suitable for a single crystal X-ray diffraction analysis was obtained by recrystallization from toluene at −20 °C. ^1^H NMR (400 MHz, r.t., C_6_D_6_): *δ* = −0.96 (s, 2H, Ni-H), 1.05 (br s, 24H, CH(C*H*_3_)_2_),1.71 (s, 12H, C = C-Me), 4.98 (sept, J_H-H_ = 7.3 Hz, 4H, CH(CH_3_)_2_), 7.20-7.23 (m, 4 H, Ph), 7.28-7.32 (m, 8H, Ph), 8.01-8.03 (m, 8H, Ph). ^13^C NMR (100 MHz, r.t., C_6_D_6_): 10.18 (s, CH(*C*H_3_)_2_), 21.97 (brs, C = C-(CH_3_)), 53.23 (s, *C*H(CH_3_)_2_), 124.51, 127.19, 127.48, 136.52, 146.72, 191.62 (Ni-C). ^29^Si NMR (119 MHz, r.t., C_6_D_6_): 115.61 (s). Anal calcd for C_46_H_62_N_4_Si_2_Ni_2_; C 65.42, H 7.40, N: 6.63; found: C 65.20, H 7.47, N: 6.37.

### Synthesis of 8

In a 50 mL schlenk tube, Ph_2_(Cl)Si-SiPh_2_SiPh_2_Si(Cl)Ph_2_ (87 mg, 0.11 mmol) was dissolved in toluene (3 mL), then toluene (3 mL) solution of the mixture of Pt(COD)_2_ (179 mg, 0.44 mmol) and CN^*t*^Bu (100 μL, 0.88 mmol) was added to this solution at room temperature. The solution was stirred at 80 ^o^C for overnight. The solvent was removed *in vacuo*, and the obtained solid was washed with Et_2_O to afford **8** as red powder in 86% yield (195 mg, 0.094 mmol). Red crystals suitable for a single crystal X-ray diffraction analysis was obtained by recrystallization from toluene at −20 °C. ^1^H NMR (400 MHz, r.t., C_6_D_6_): *δ* = 0.78 (s, 36H, ^*t*^Bu), 0.84 (s, 18H, ^*t*^Bu), 7.12–7.14 (m, 12H, Ph), 7.15-7.33 (m, 12H, Ph), 8.12–8.14 (m, 8H, Ph), 8.42-8.44 (m, 8H, Ph). ^13^C NMR (100 MHz, r.t., C_6_D_6_): 29.40 (s, C(*C*H_3_)_3_), 30.24 (s, C(*C*H_3_)_3_), 56.53 (s, *C*(CH_3_)_3_), 57.60 (s, *C*(CH_3_)_3_), 126.41 (s, Ph), 126.52 (s, Ph), 127.46 (s, Ph), 135.27 (s, Ph), 135.87 (s, Ph), 137.60 (s, *J*_Pt-C_ = 20.0 Hz, Ph), 148.43 (s, Ph), 151.84 (s, Ph). ^29^Si NMR (119 MHz, r.t., C_6_D_6_): 64.68 (s,), 158.22 (s) Anal calcd for C_78_H_94_N_6_Si_4_Cl_2_Pt_4_; C 45.06, H 4.56, N 4.04; found: C 45.01, H 4.49, N 3.63.

## Supplementary information

Supplementary information

Description of Additional Supplementary Files

Supplementary Data 1

Supplementary Data 2

Supplementary Data 3

Supplementary Data 4

Supplementary Data 5

Supplementary Data 6

Supplementary Data 7

Supplementary Data 8

Supplementary Data 9

Supplementary Data 10

Supplementary Data 11

Supplementary Data 12

Supplementary Data 13

Supplementary Data 14

Supplementary Data 15

Supplementary Data 16

Supplementary Data 17

Supplementary Data 18

Supplementary Data 19

Supplementary Data 20

Supplementary Data 21

Supplementary Data 22

Supplementary Data 23

Supplementary Data 24

## Data Availability

The data generated in this study are provided in the Supplementary Information and this published article. X-ray structural data of compound **2** (ccdc 2231760), **3** ccdc (2231758), **4** (ccdc 2231757), **5a** (ccdc 2231762), **5b** (ccdc 2231763), **6** (ccdc 2231761), **7** (ccdc 2231756) and **8** (ccdc 2231759) are available free of charge from the Cambridge Crystallographic Data Center via www.ccdc.cam.ac.uk/data_request/cif.
