# Supplementary material for: Dinuclear and tetranuclear group 10 metal complexes constructed from linear tetrasilane comprising both Si-H and Si-Si moieties
Source: Commun Chem. 2023 May 15;6:93. doi: 10.1038/s42004-023-00892-8 (PMC10185686; doi:10.1038/s42004-023-00892-8)
Supplement: Supplementary file 1 — Supplementary information [file 42004_2023_892_MOESM1_ESM.pdf]

Supplementary Information for

Dinuclear and tetranuclear group 10 metal complexes constructed from linear tetrasilane comprising both Si-H and Si-Si moieties

Yoshihiko Umehara,[1] Ryosuke Usui,[1] Yoshimasa Wada[1,2] and Yusuke Sunada,[1,2,3*]

[1] Department of Applied Chemistry, School of Engineering, The University of Tokyo, 4-6-1 Komaba, Meguro-ku, Tokyo 153-8505 Japan.

[2] Institute of Industrial Science, The University of Tokyo, 4-6-1 Komaba, Meguro-ku, Tokyo 153-8505 Japan.

[3] JST PRESTO, Honcho, Kawaguchi, Saitama, 332-0012 Japan.

Supplementary Note 1

General. Manipulation of air and moisture sensitive compounds was carried out under a dry nitrogen atmosphere using Schlenk tube techniques associated with a high-vacuum line or in the glove box which was filled with dry nitrogen. All solvents were purchased from Kanto Chemical Co. Inc., and was dried over activated molecular sieves. ^{1}H, ^{13}C, ^{29}Si NMR spectra were recorded on a JEOL Lambda 400 spectrometer at ambient temperature unless otherwise noted. ^{1}H, ^{13}C, ^{29}Si NMR chemical shifts (δ values) were given in ppm relative to the solvent signal (^{1}H, ^{13}C) or standard resonances (^{29}Si: external tetramethylsilane). Elemental analyses were performed by a Thermo Scientific FLASH 2000 Organic Elemental Analyzer. IR spectra were recorded on a PerkinElmer Spectrum Two spectrometer. The starting compounds, $Pd(CN^{t}Bu)_2$[1], $Ph_2(H)SiSiPh_2SiPh_2Si(H)Ph_2$ and $Ph_2(Cl)SiSiPh_2SiPh_2Si(Cl)Ph_2$[2] were synthesized by the method reported in the literature. $Pd(^{i}PrIM^{Me})_2$ was generated and used according to the slightly modified method reported in the literature.[3] All reagents were purchased from Tokyo Chemical Industries Co., Ltd. or Sigma-Aldrich, and were used without further purification.

Supplementary Fig. 1. ^{1}H NMR spectrum of solution of **2** in C_6D_6 at room temperature.

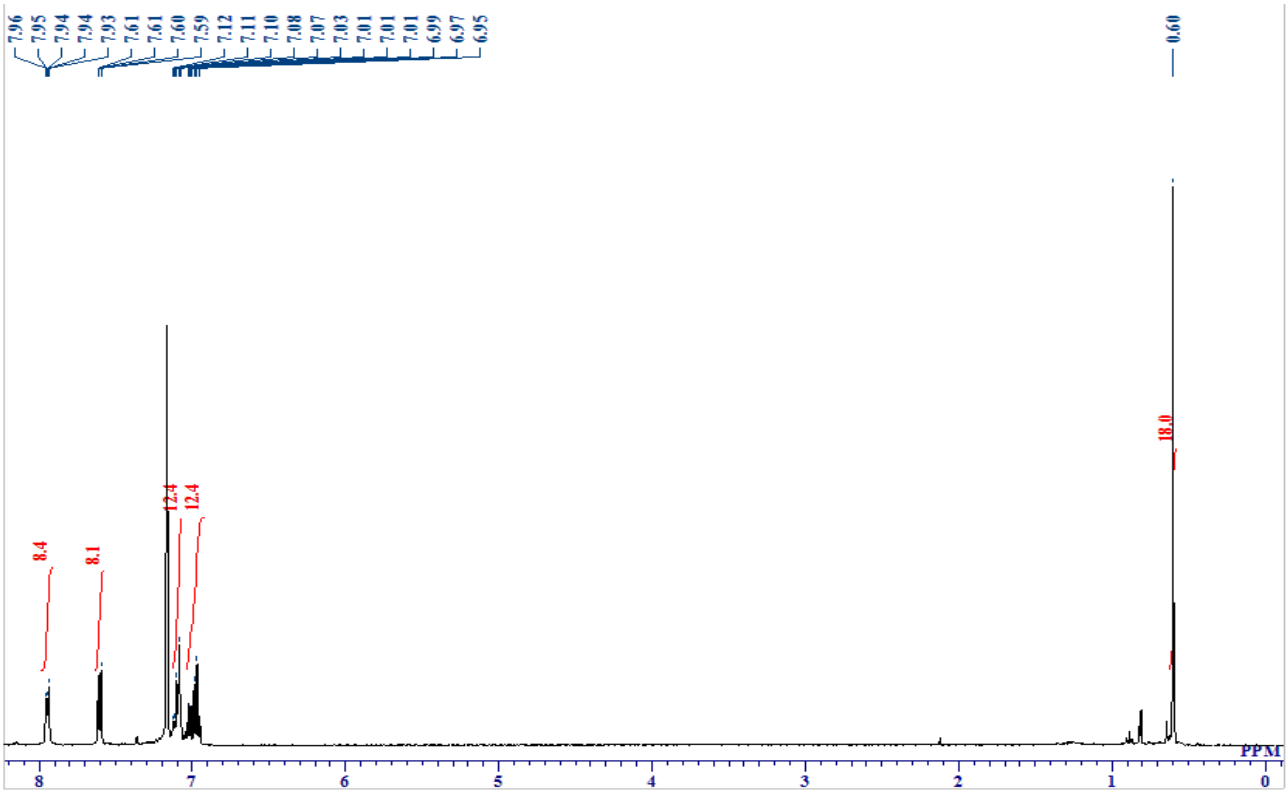

Supplementary Fig. 2. ^{13}C NMR spectrum of solution of **2** in C_6D_6 at room temperature.

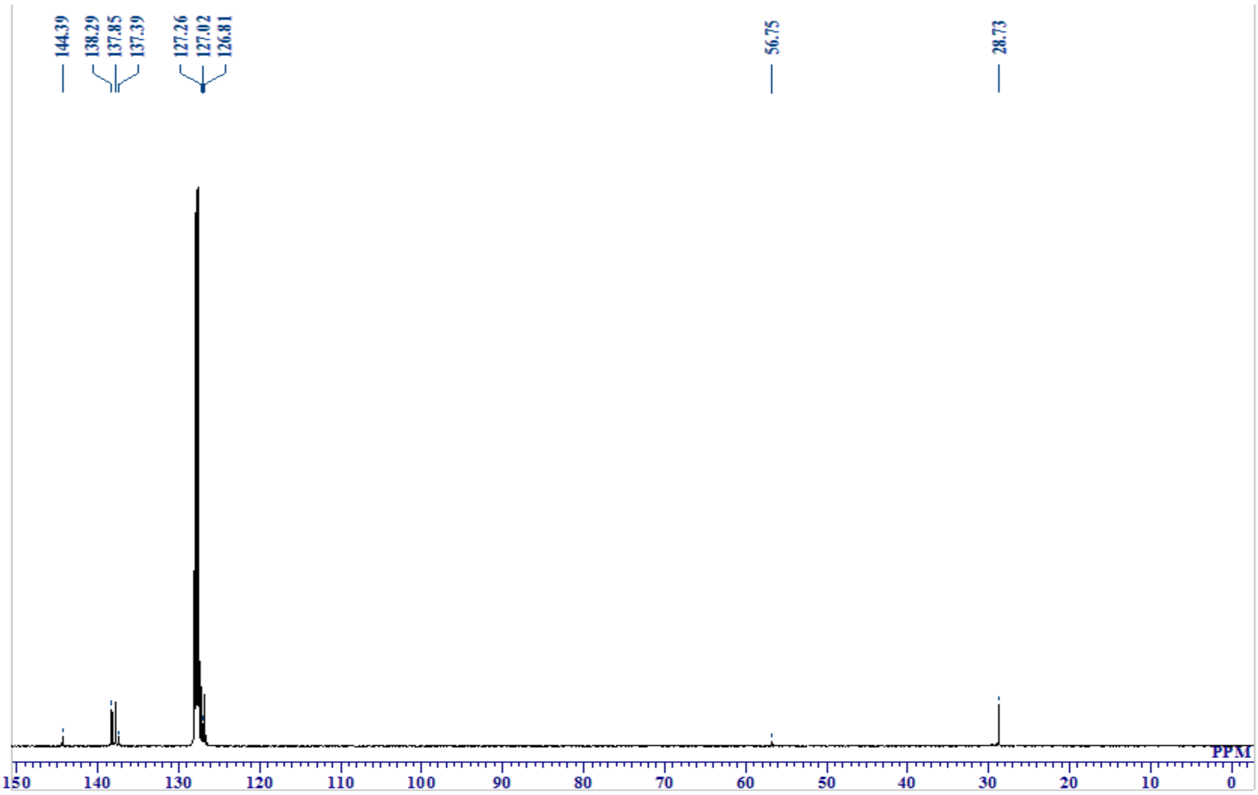

Supplementary Fig. 3. ^{1}H NMR spectrum of solution of **3** in C_6D_6 at room temperature.

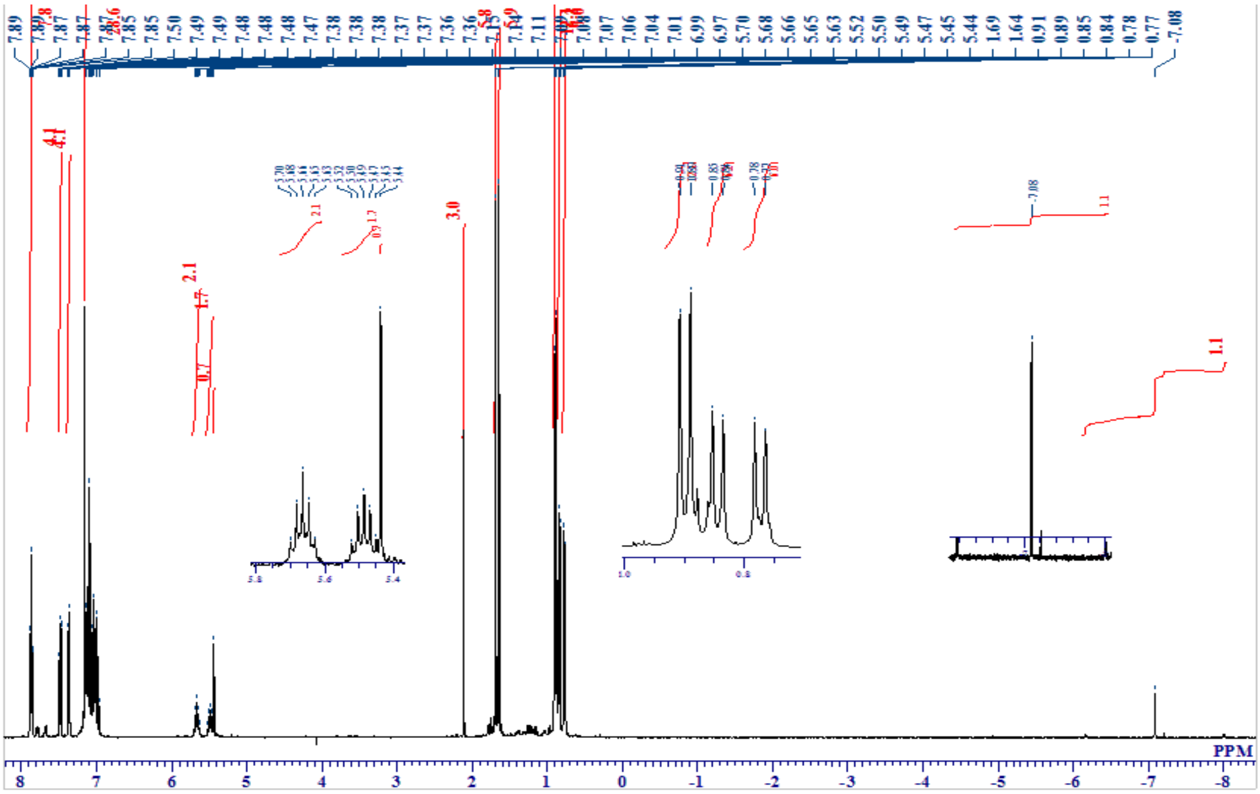

Supplementary Fig. 4. ^{13}C NMR spectrum of solution of **3** in C_6D_6 at room temperature.

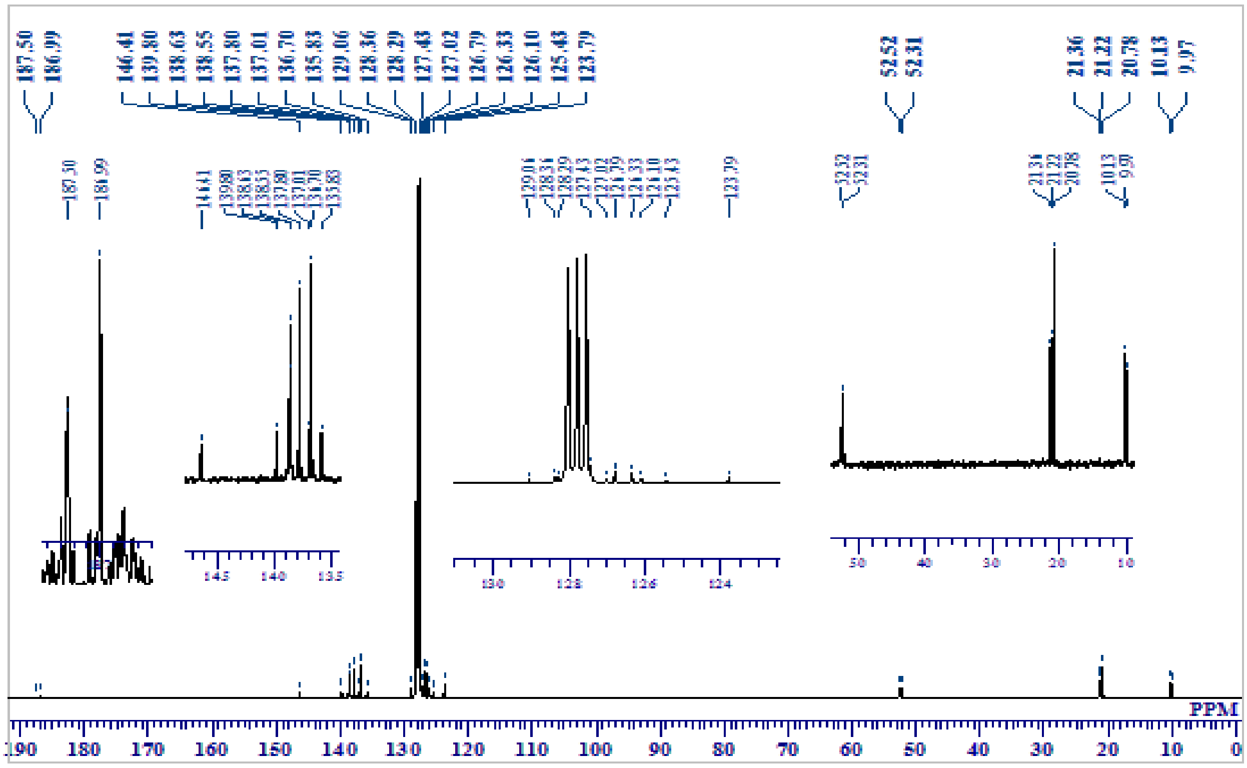

Supplementary Fig. 5. ^{1}H NMR spectrum of solution of **4** in C_6D_6 at room temperature.

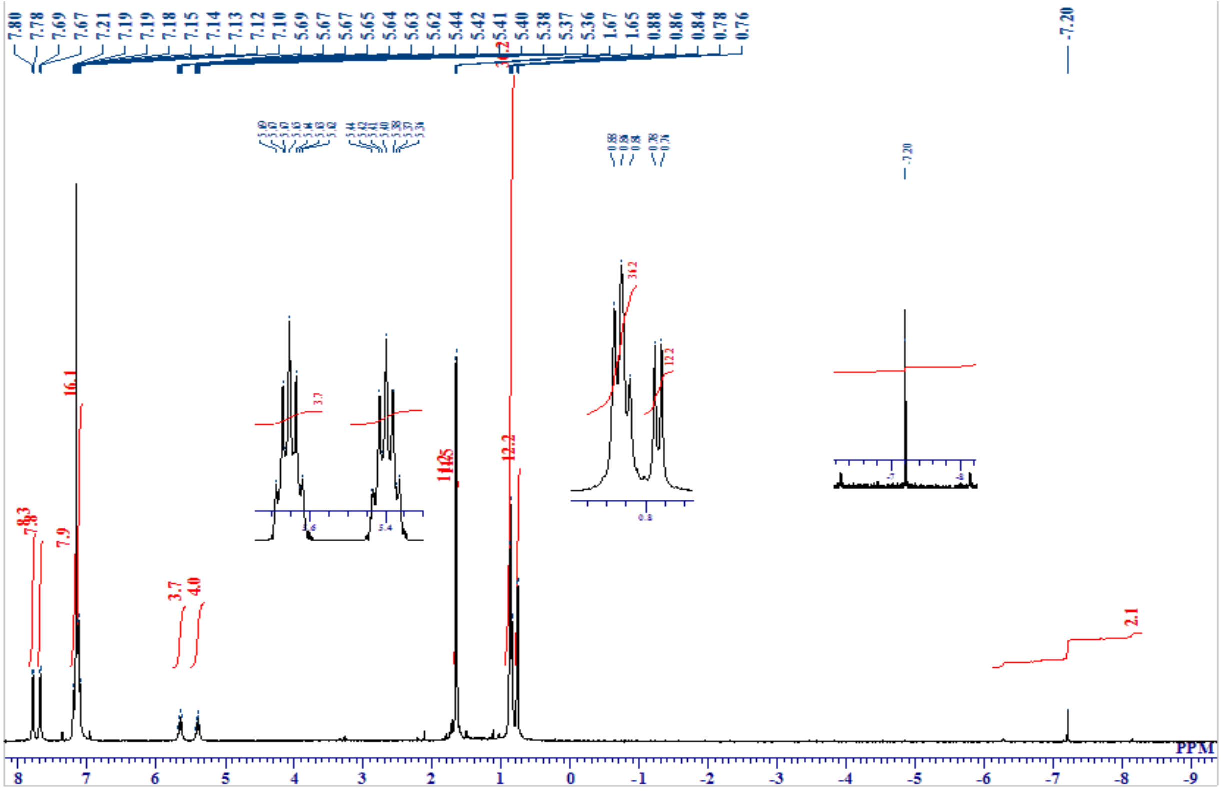

Supplementary Fig. 6. ^{1}H NMR spectrum of solution of **5a** in C_6D_6 at room temperature.

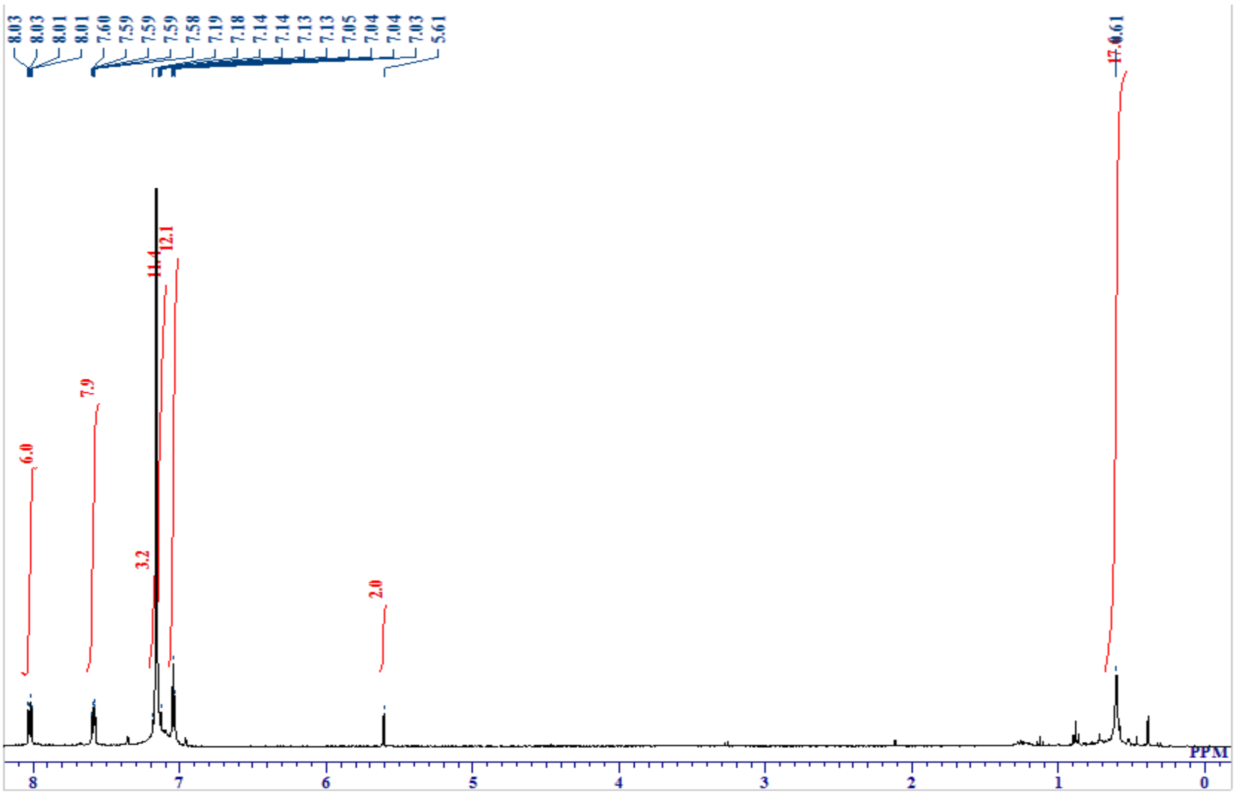

Supplementary Fig. 7. ^{13}C NMR spectrum of solution of **5a** in C_6D_6 at room temperature.

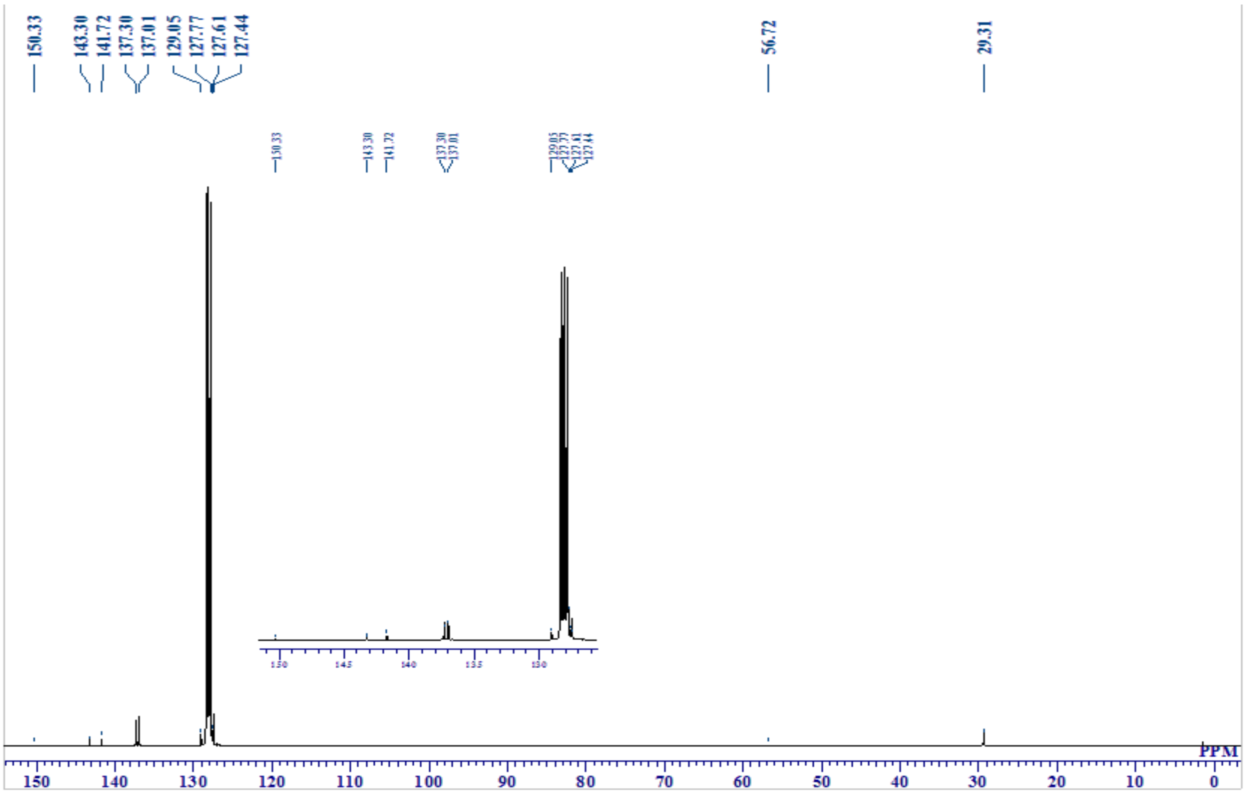

Supplementary Fig. 8. ^{29}Si NMR spectrum of solution of **5a** in C_6D_6 at room temperature.

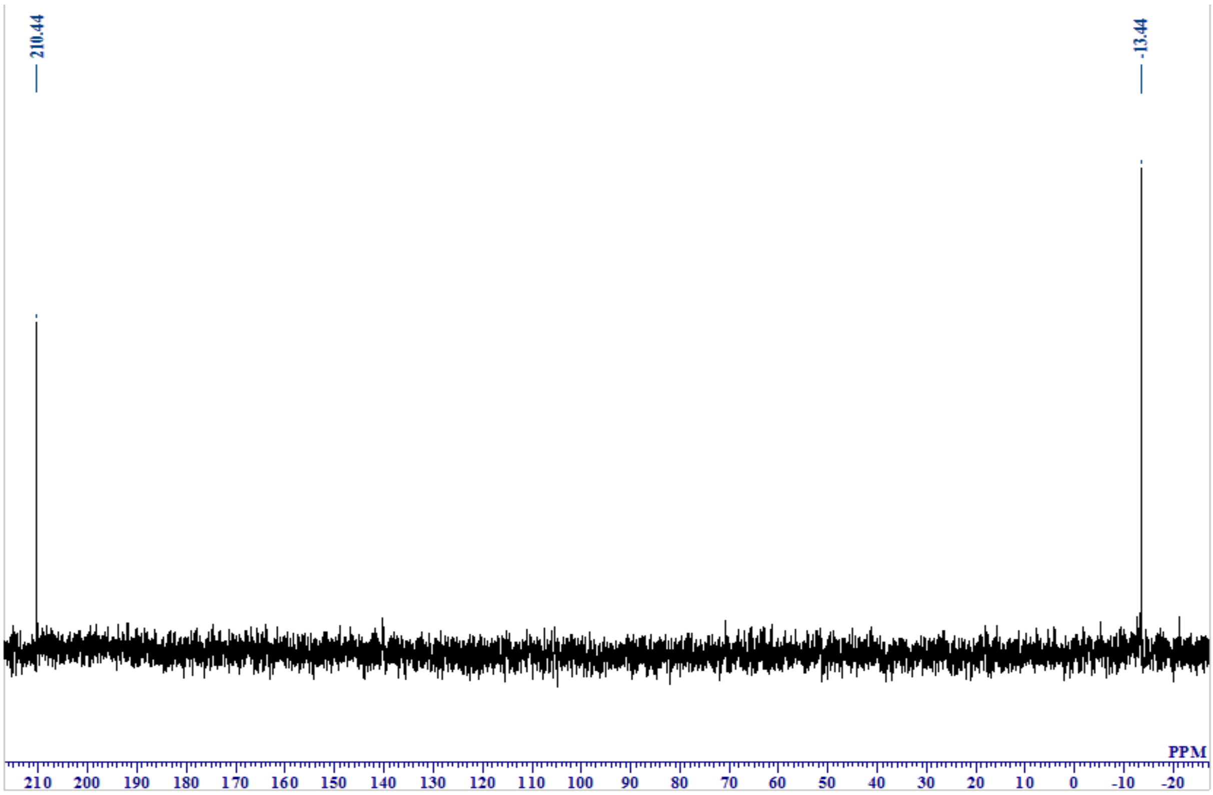

Supplementary Fig. 9. ^{1}H NMR spectrum of solution of **5b** in C_6D_6 at room temperature.

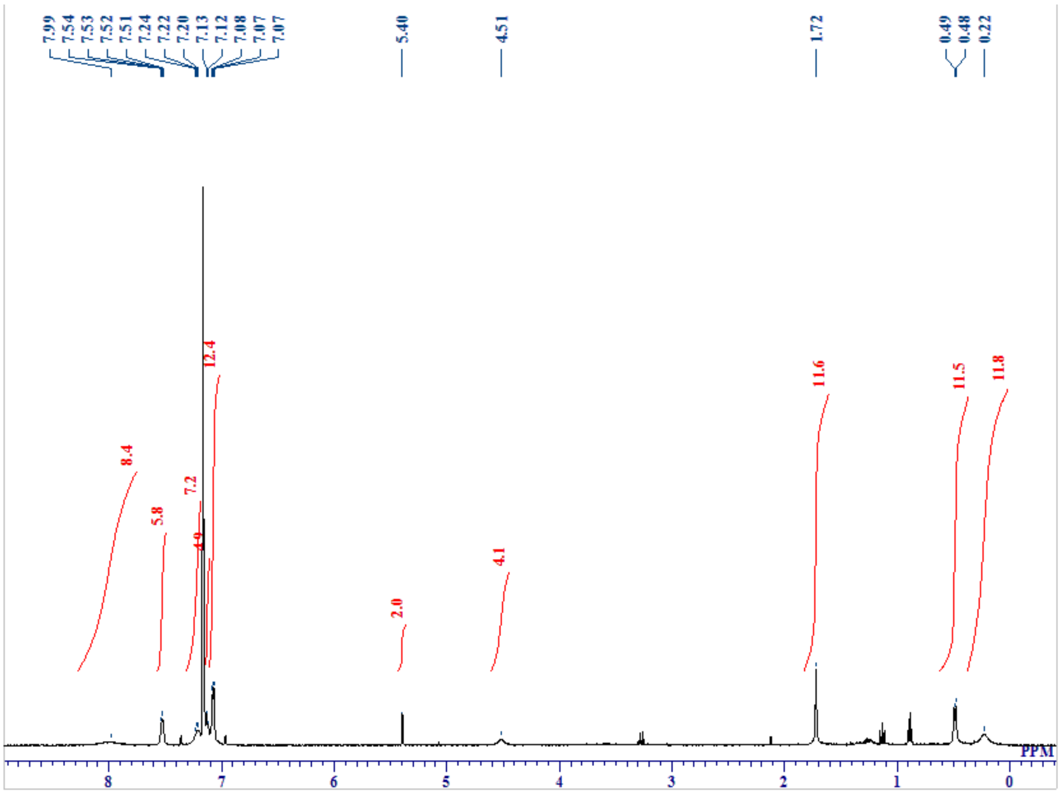

Supplementary Fig. 10. Variable temperature ^{1}H NMR spectrum of solution of **5b** in toluene-d_8.

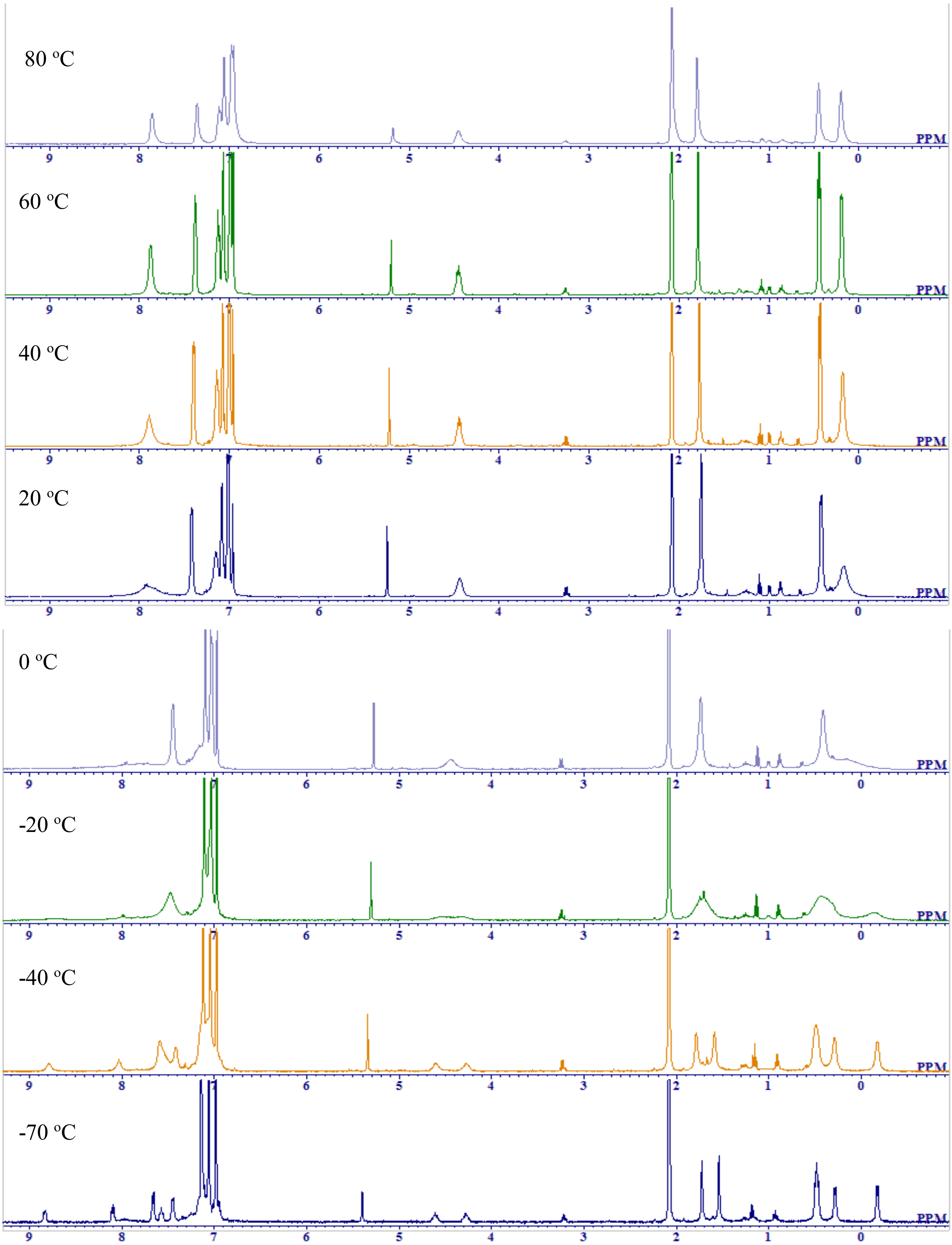

Supplementary Fig. 11. ^{1}H NMR spectrum of solution of **5b** in toluene-d_8 at -70 °C.

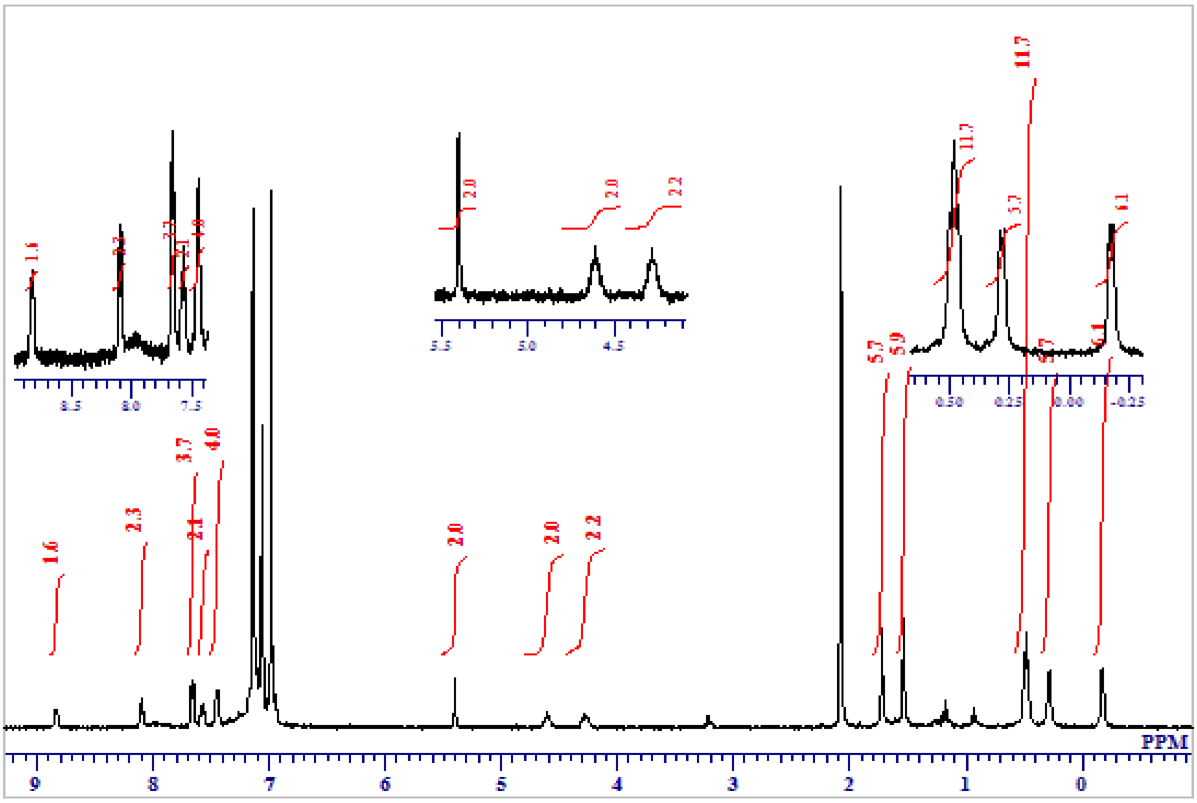

Supplementary Fig. 12. ^{13}C NMR spectrum of solution of **5b** in C_6D_6 at room temperature.

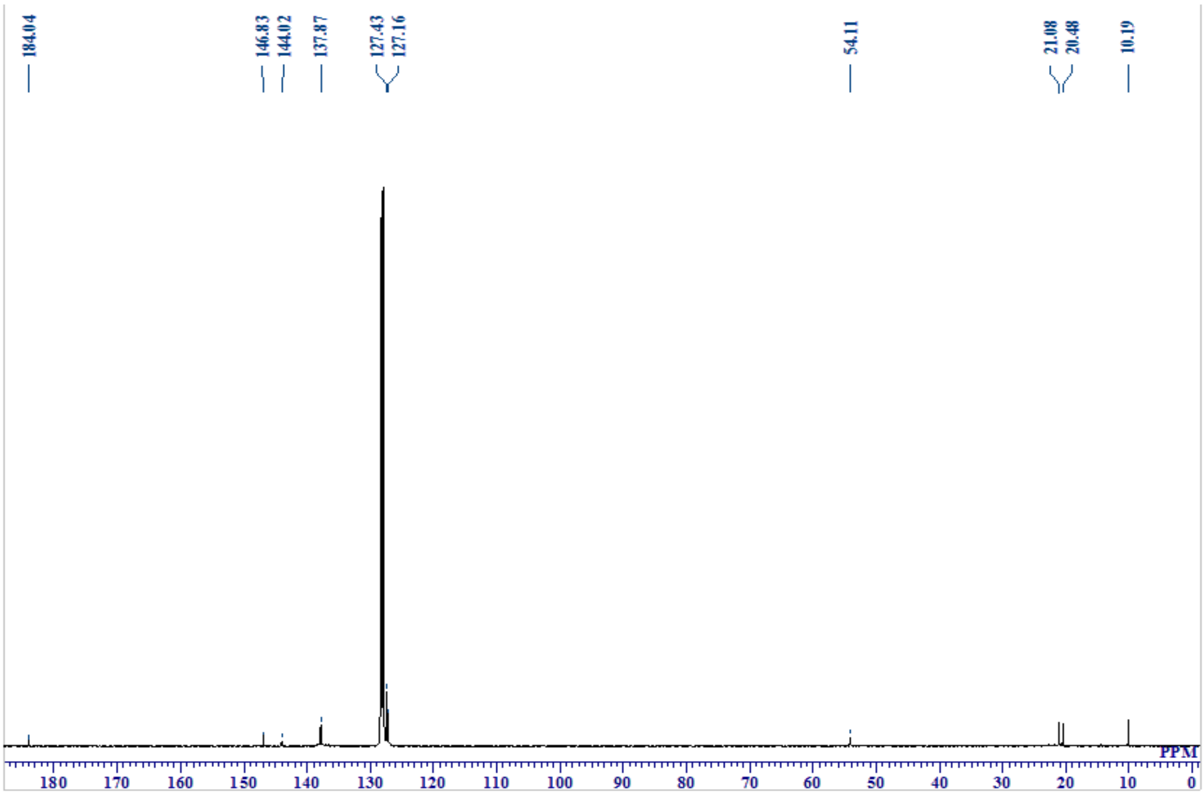

Supplementary Fig. 13. ^{1}H NMR spectrum of solution of **6** in C_6D_6 at room temperature.

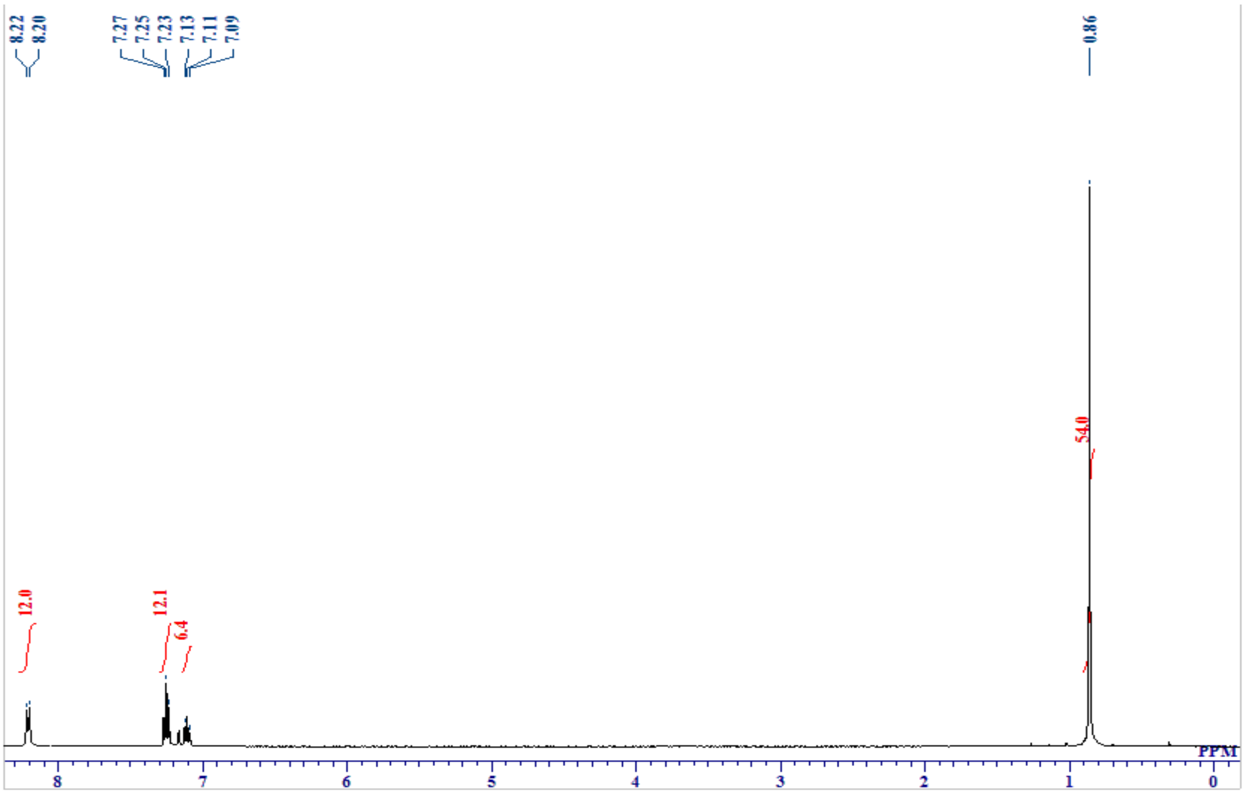

Supplementary Fig. 14. ^{13}C NMR spectrum of solution of **6** in C_6D_6 at room temperature.

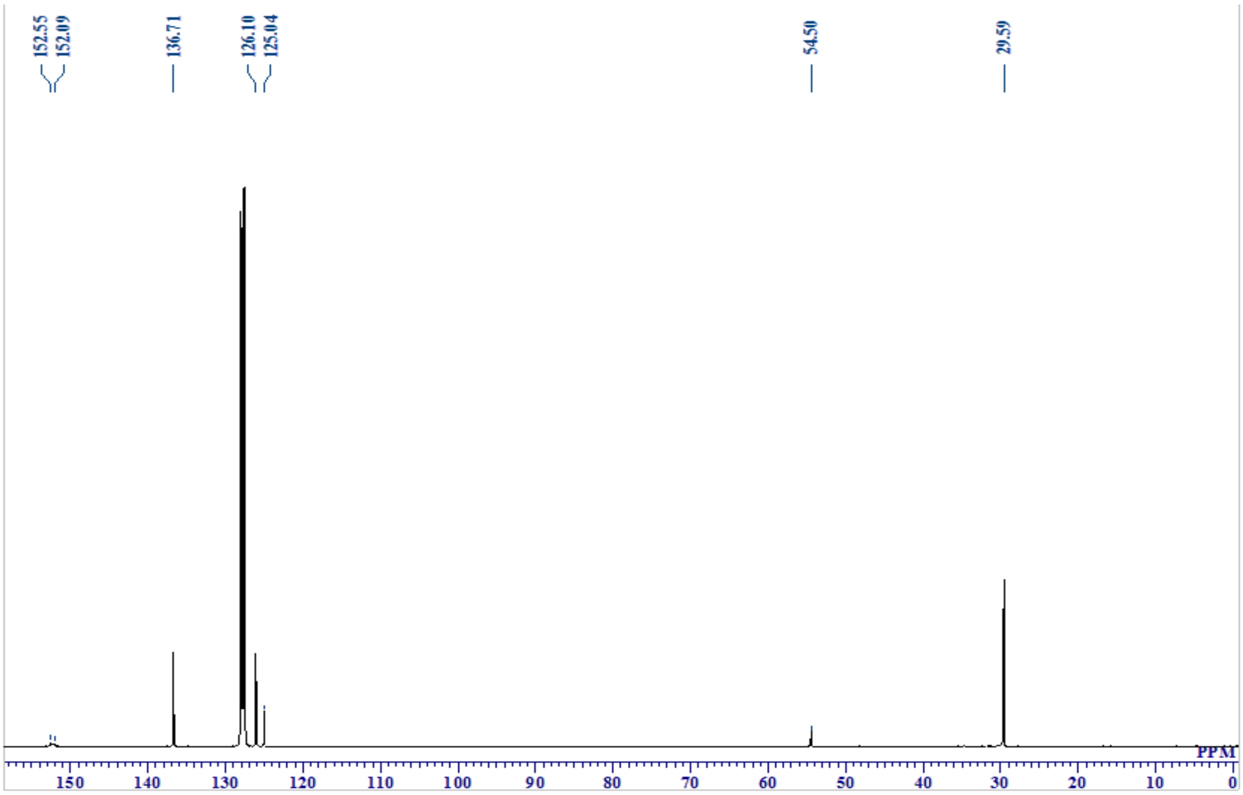

Supplementary Fig. 15. ^{29}Si NMR spectrum of solution of **6** in C_6D_6 at room temperature.

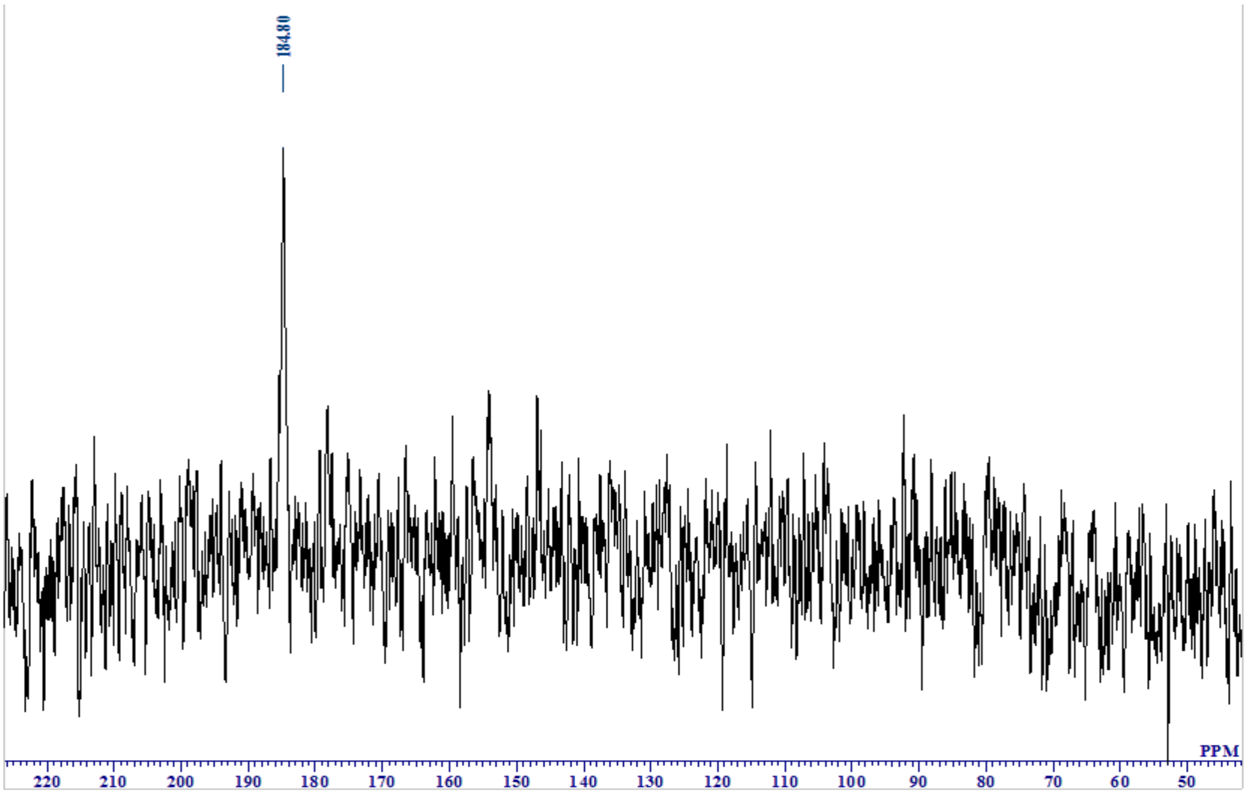

Supplementary Fig. 16. ^{1}H NMR spectrum of solution of **7** in C_6D_6 at room temperature.

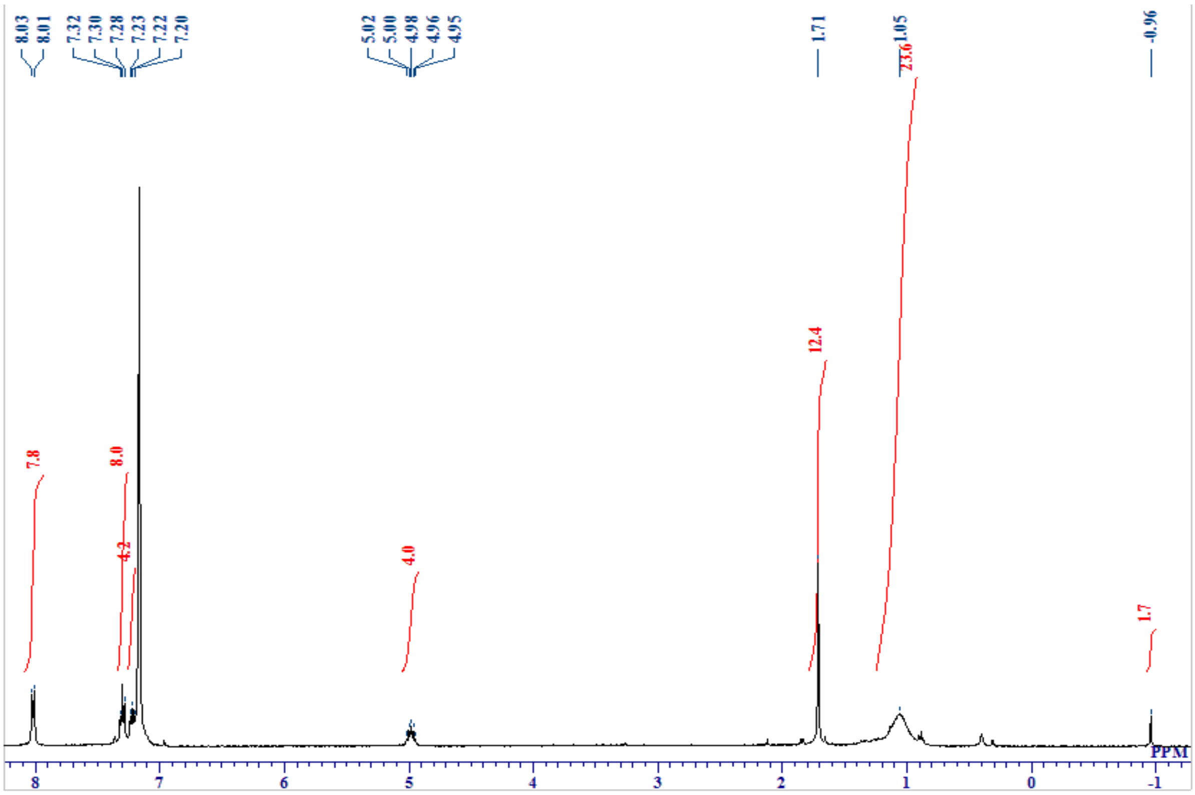

Supplementary Fig. 17. ^{13}C NMR spectrum of solution of **7** in C_6D_6 at room temperature.

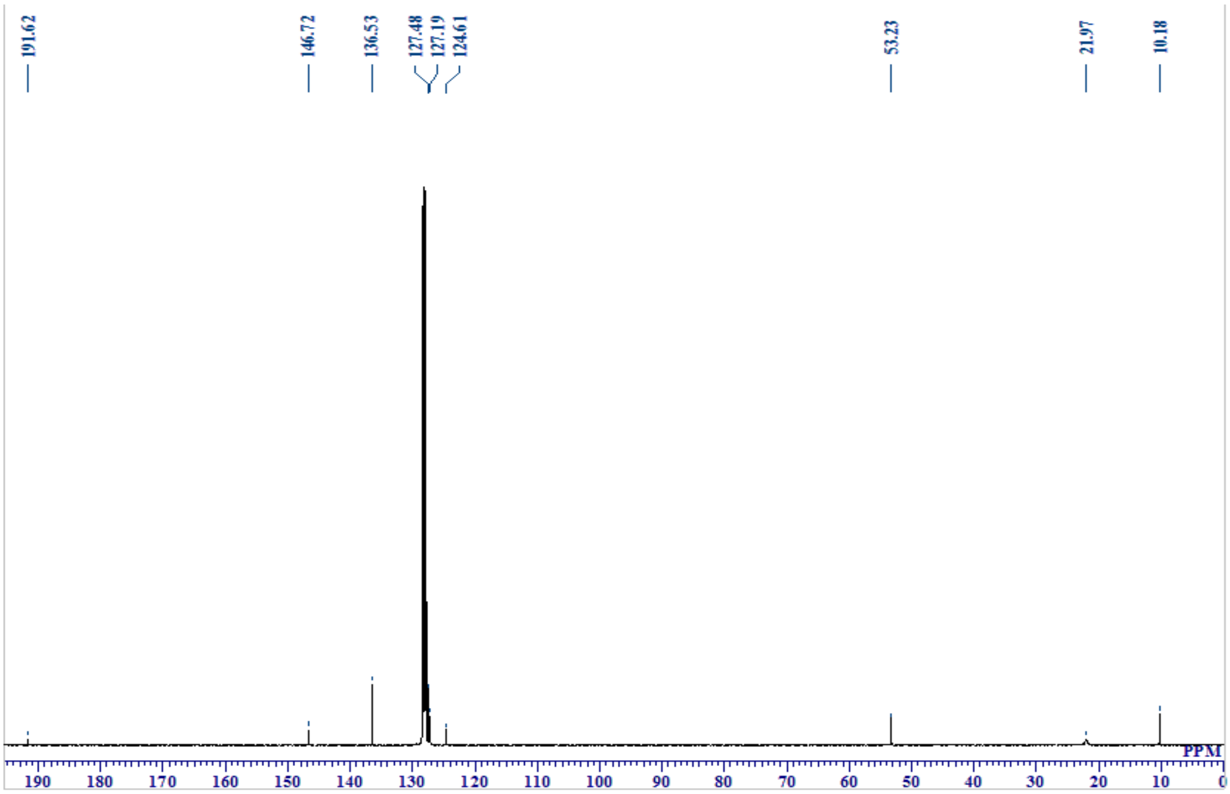

Supplementary Fig. 18. ^{29}Si NMR spectrum of solution of **7** in C_6D_6 at room temperature.

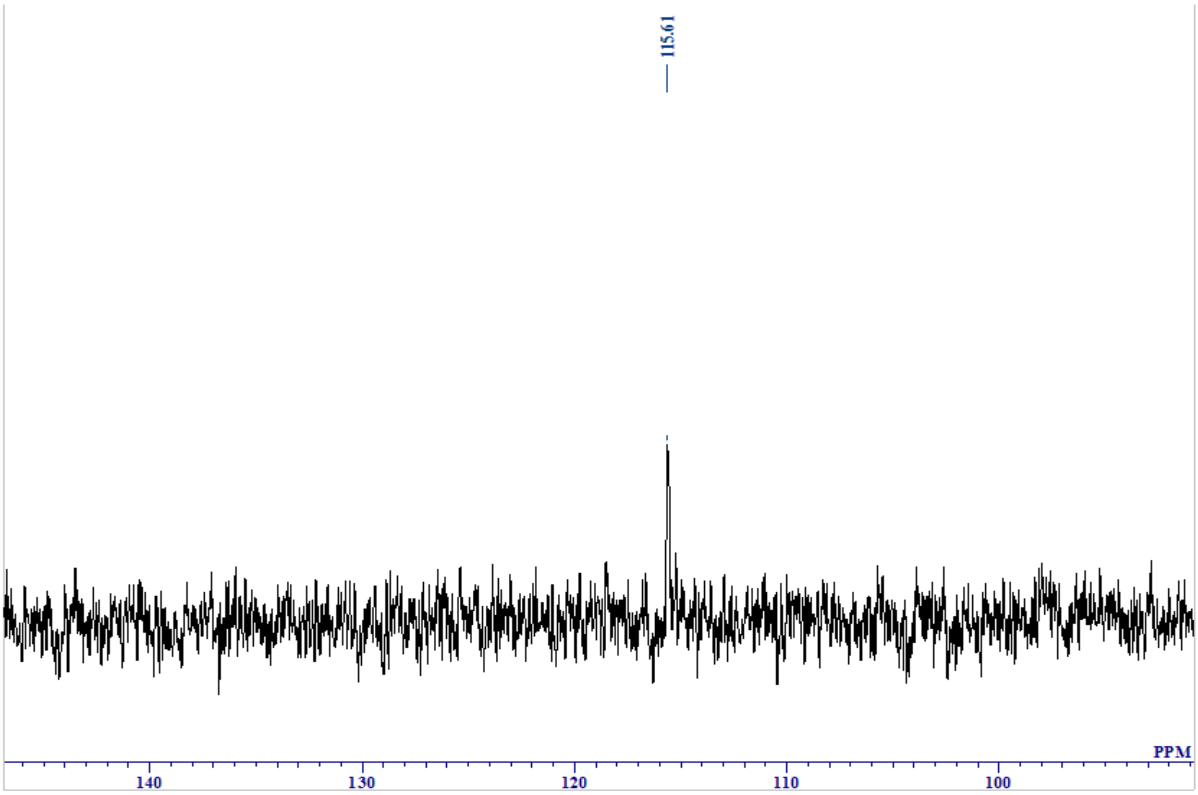

Supplementary Fig. 19. ^{1}H NMR spectrum of solution of **8** in C_6D_6 at room temperature.

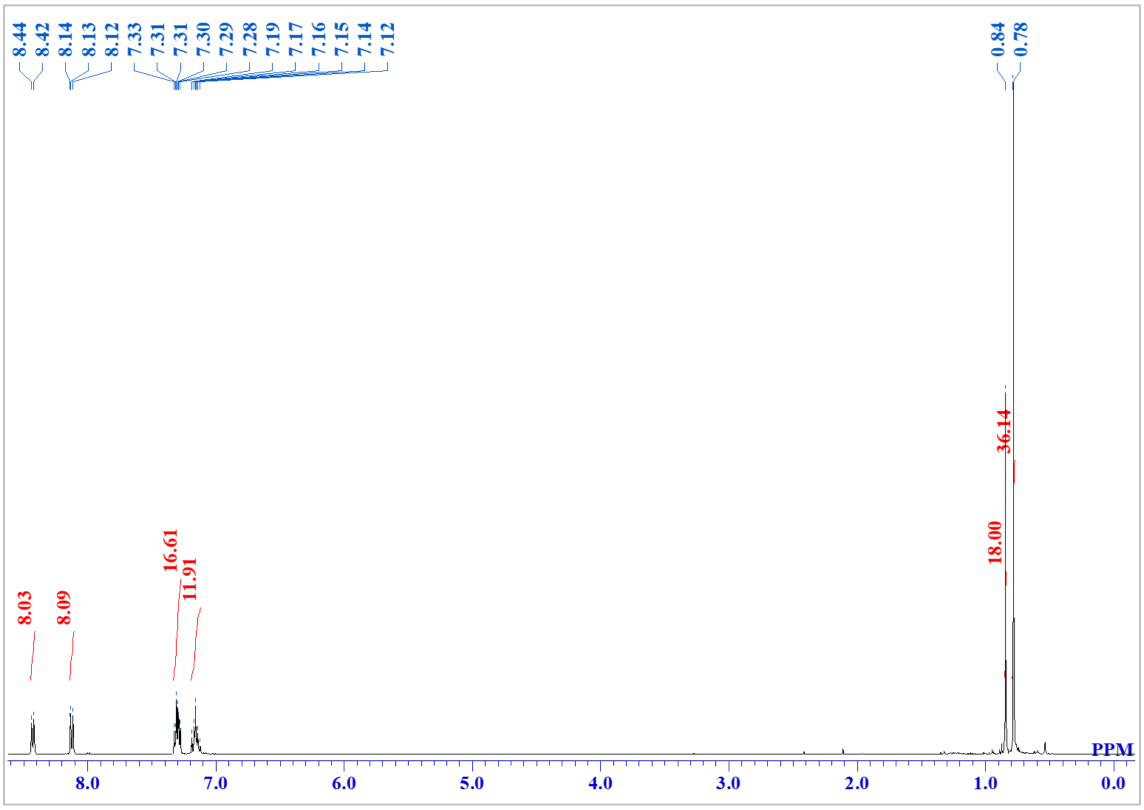

Supplementary Fig. 20. ^{13}C NMR spectrum of solution of **8** in C_6D_6 at room temperature.

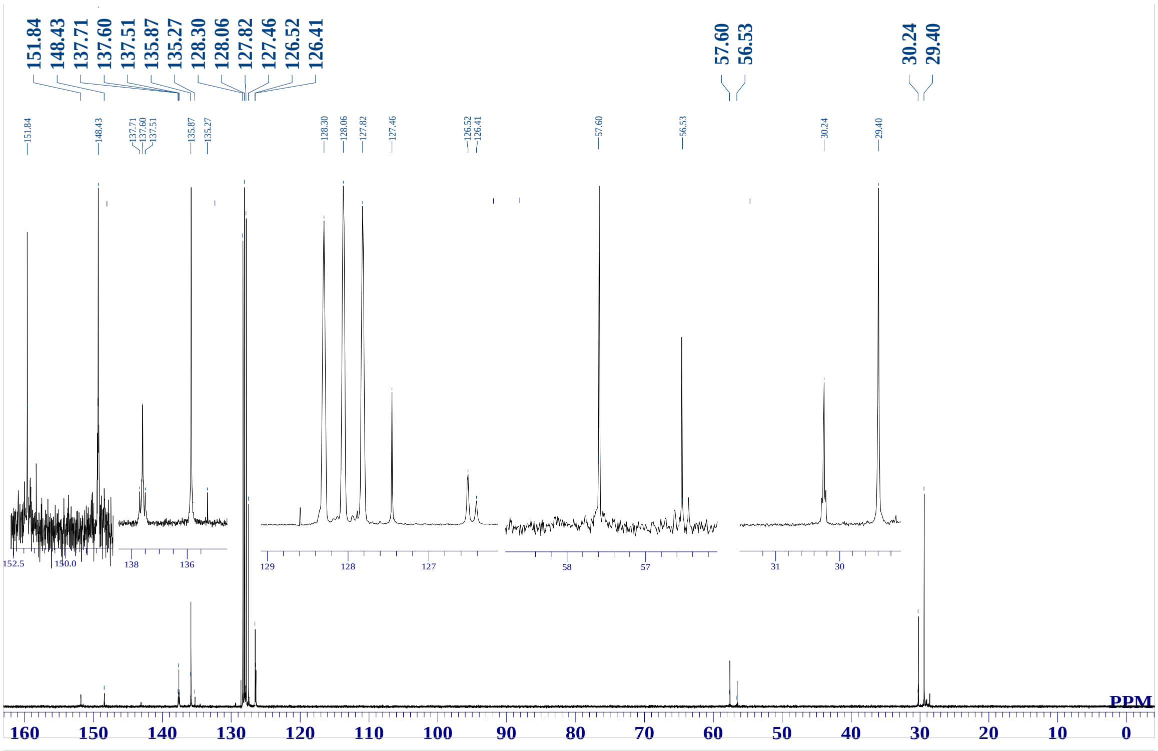

Supplementary Fig. 21. ^{29}Si NMR spectrum of solution of **8** in C_6D_6 at room temperature.

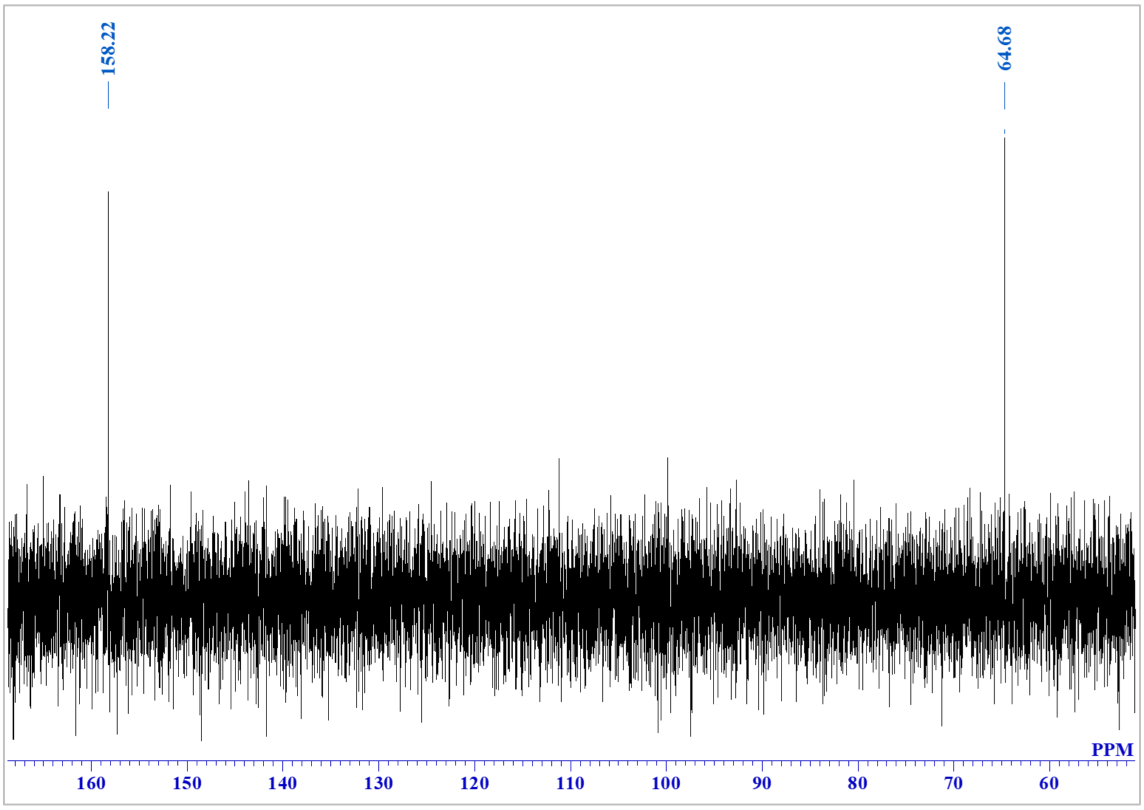

Supplementary Fig. 22. ATR-IR spectrum of **2** in the solid state.

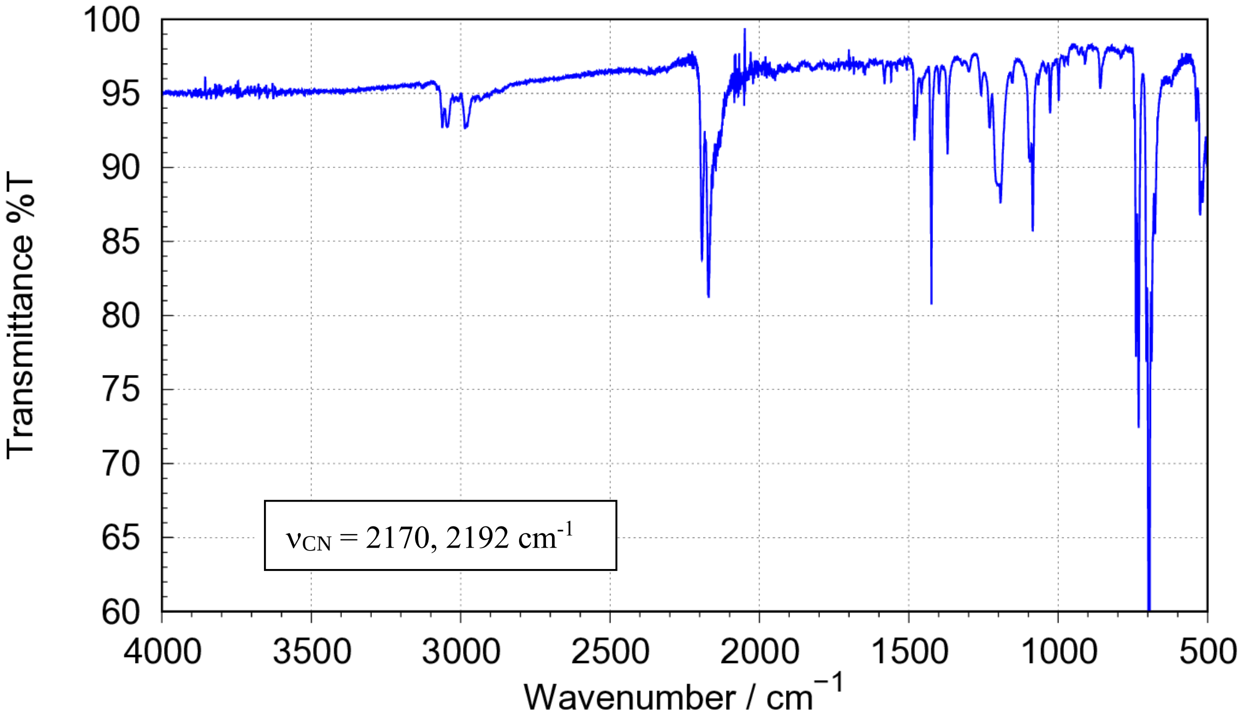

Supplementary Fig. 23. ATR-IR spectrum of **3** in the solid state.

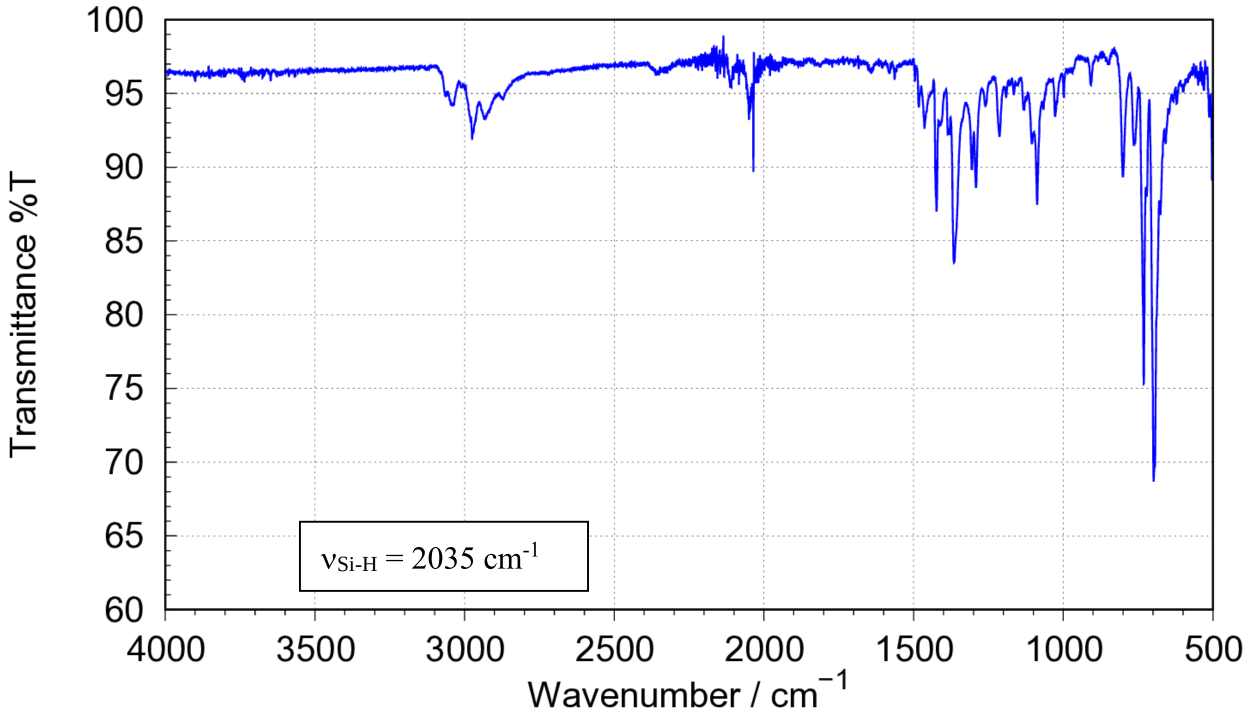

Supplementary Fig. 24. ATR-IR spectrum of **4** in the solid state.

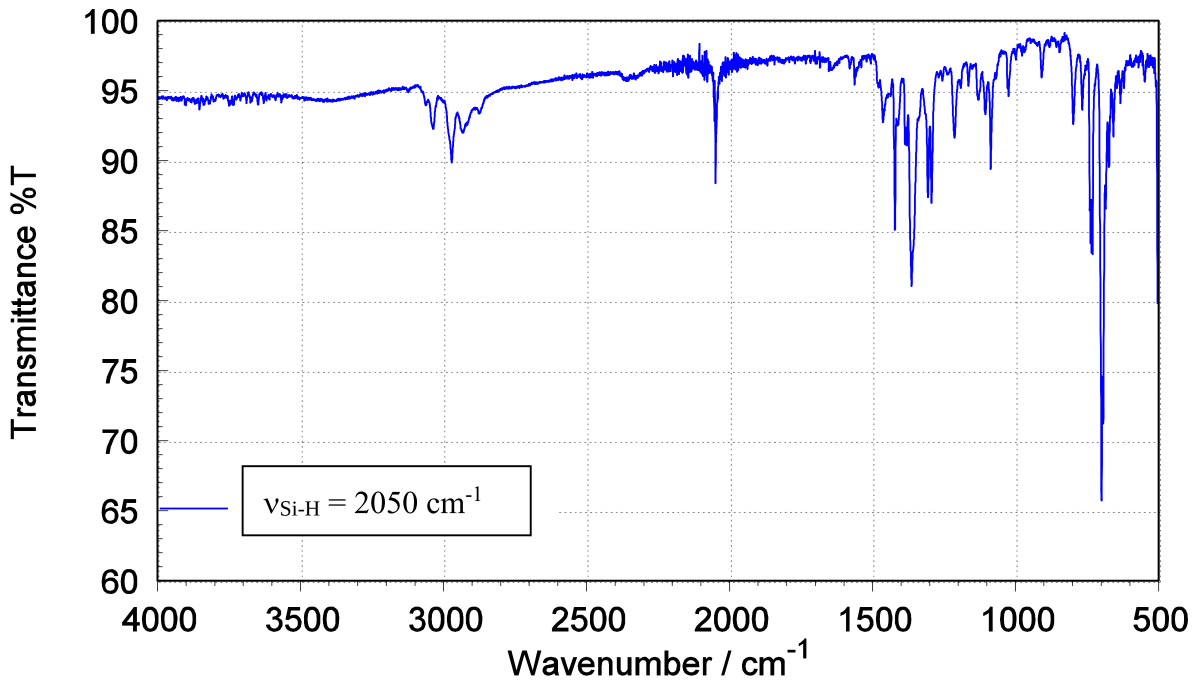

Supplementary Fig. 25. ATR-IR spectrum of **5a** in the solid state.

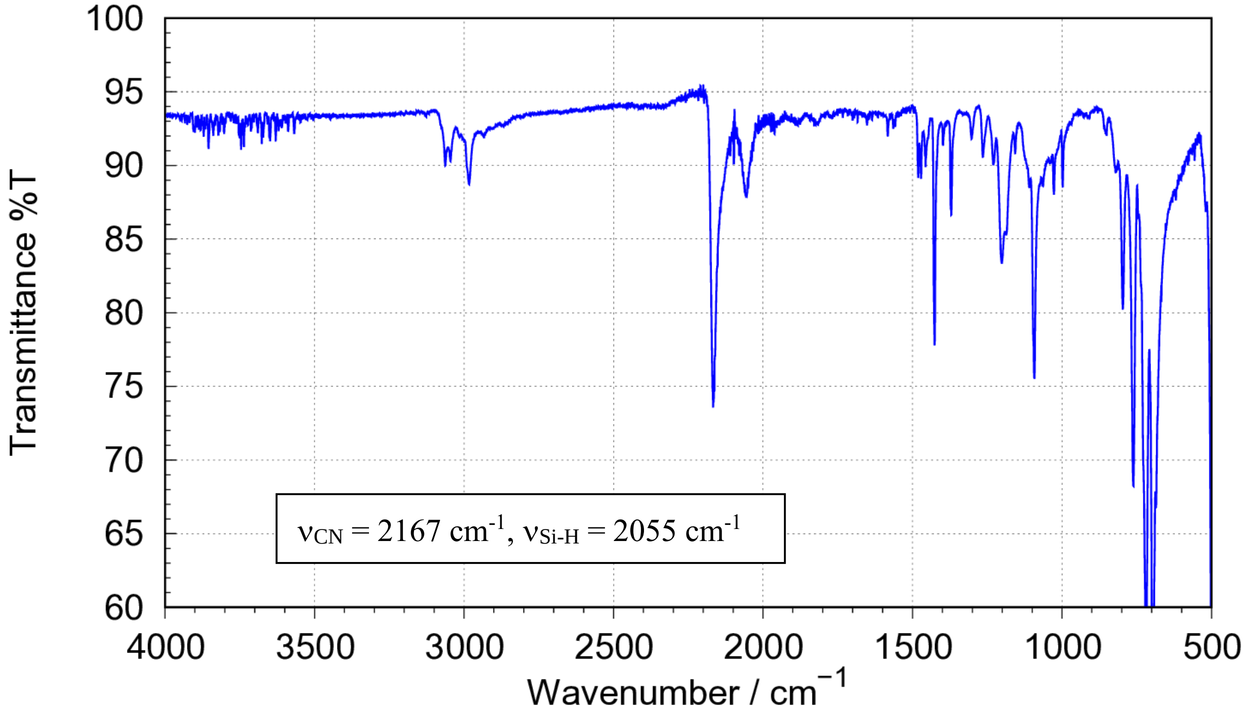

Supplementary Fig. 26. ATR-IR spectrum of **5b** in the solid state.

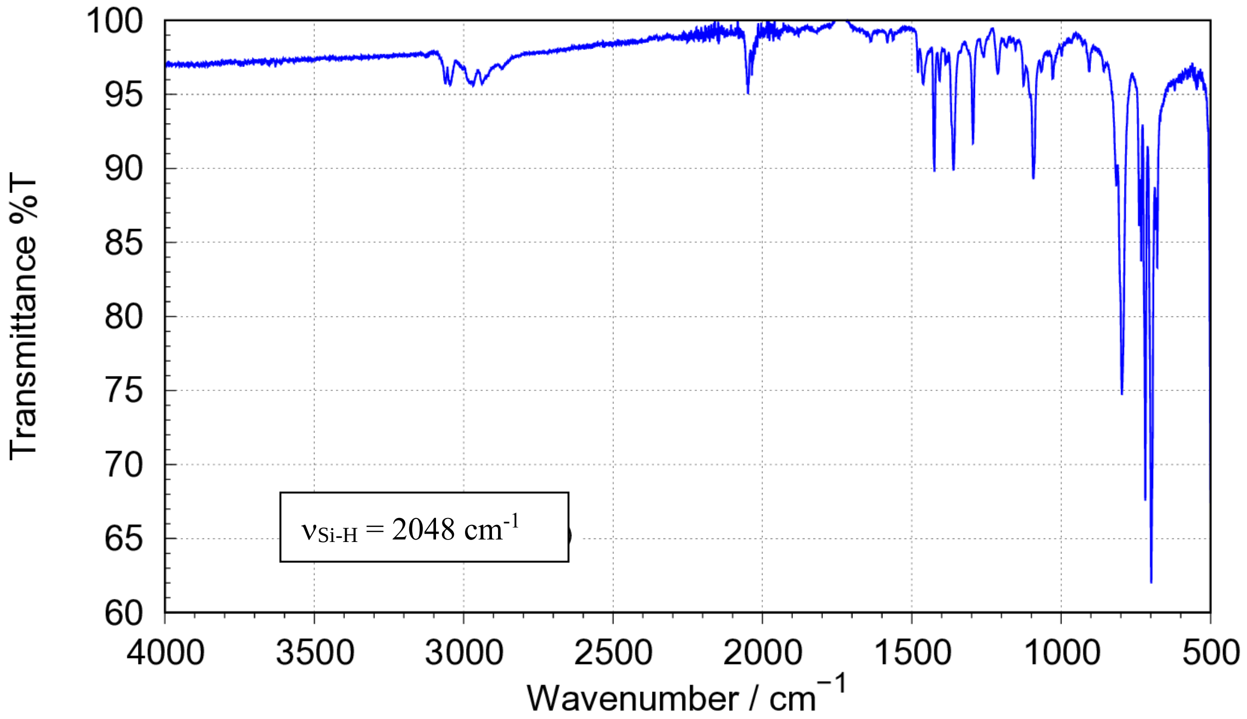

Supplementary Fig. 27. ATR-IR spectrum of **6** in the solid state.

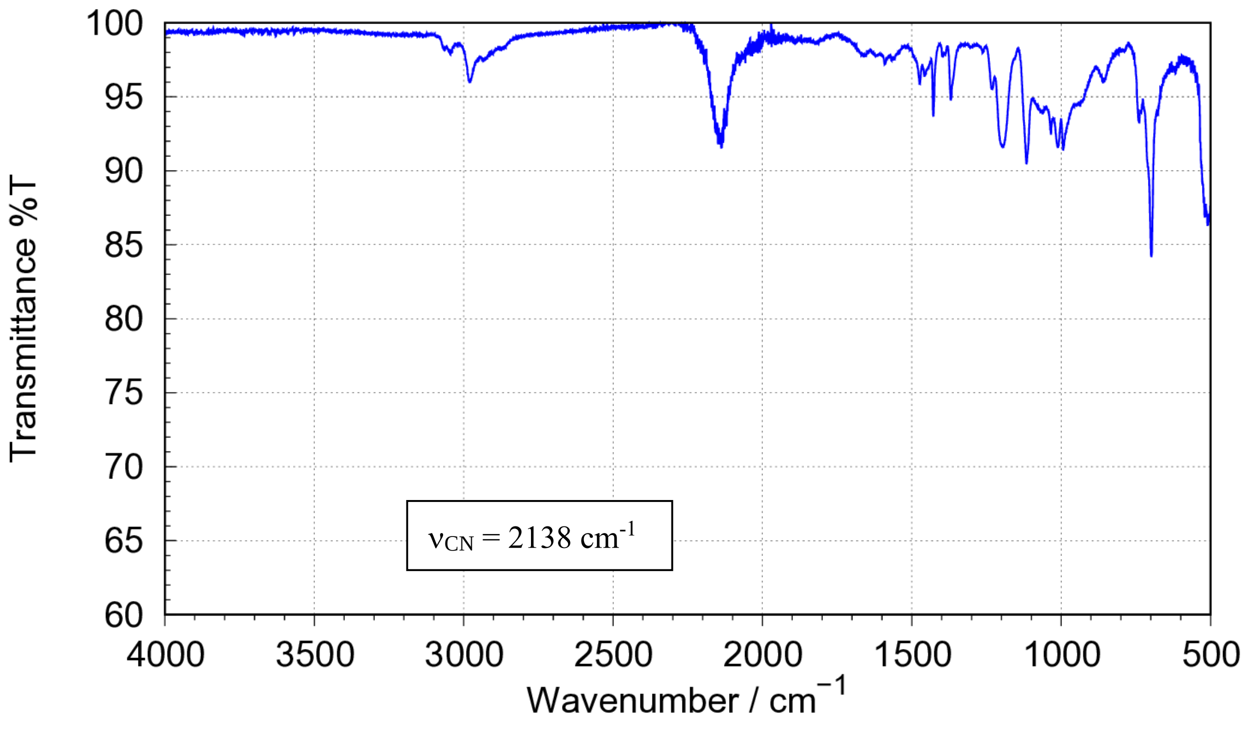

Supplementary Fig. 28. ATR-IR spectrum of **7** in the solid state.

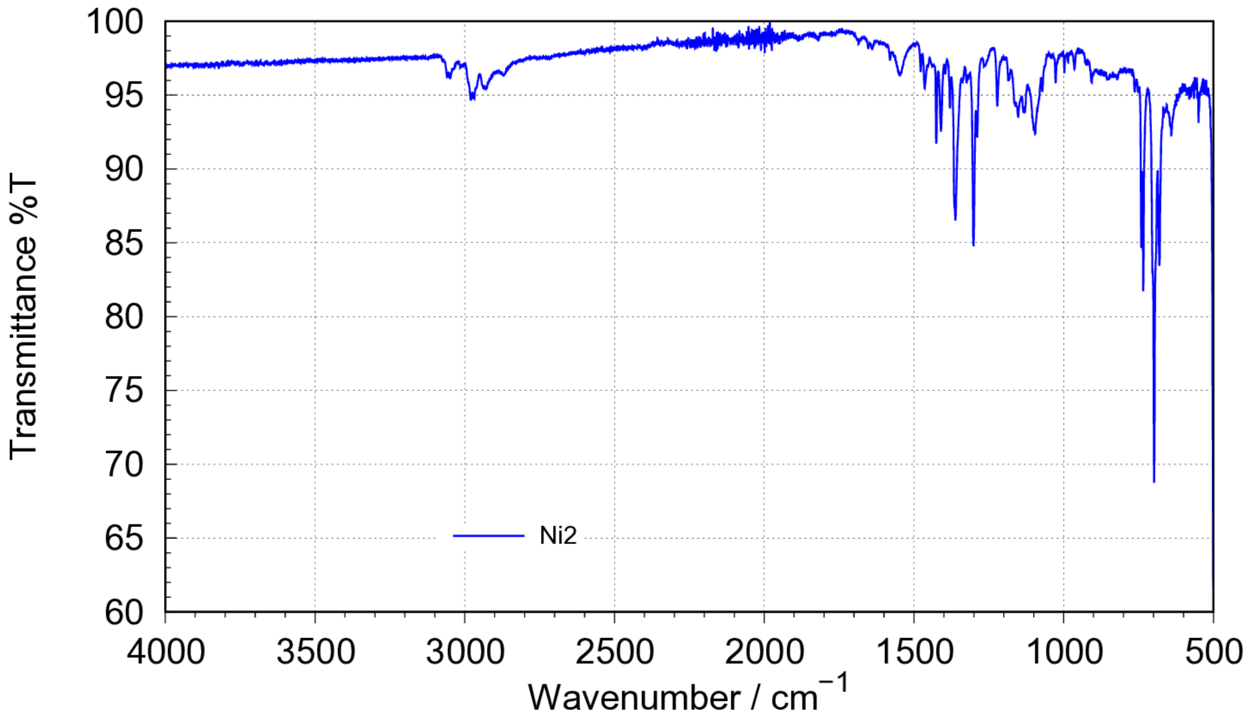

Supplementary Fig. 29. ATR-IR spectrum of **8** in the solid state.

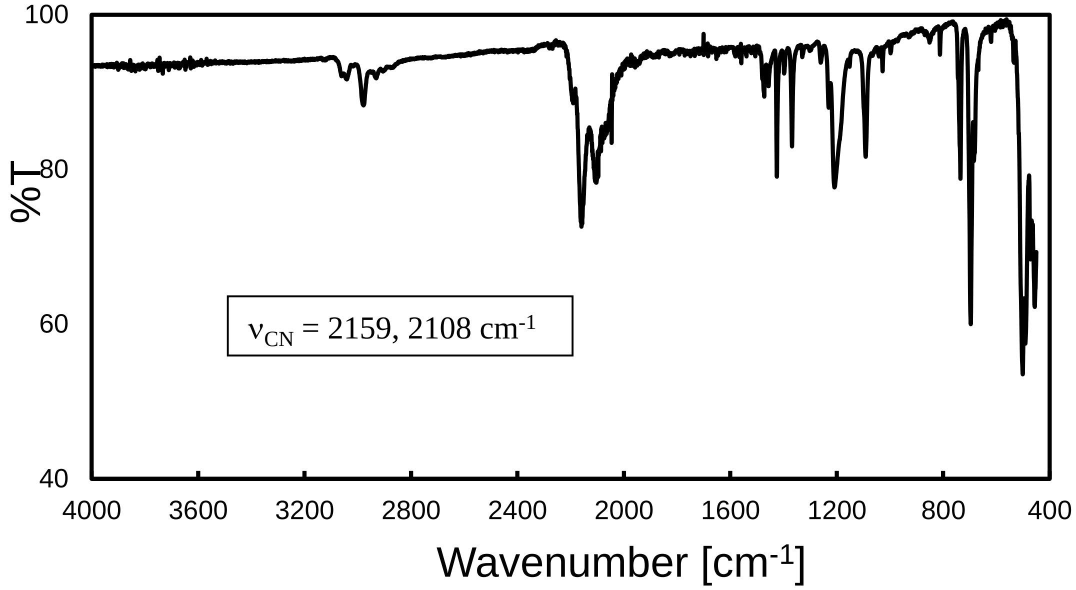

Supplementary Note 2

Computational Details All of the calculations were performed using the Gaussian 16 program.[4] Geometry optimization for complex **2**, **5a**, **5b**, **6**, **7** and **8** was carried out by using the DFT method with the B3PW91[5] functional, and the selected bond distances for these complexes as well as the calculated values of MBIs are summarized in Supplementary Figs. 30-43 and Table 1-9. Natural Bond Orbital (NBO) analyses were performed using the NBO 3.1 program implemented in Gaussian 16. The effective core potentials and the basis set by the Stuttgart–Dresden–Bonn group[6] and were used for Pt, Pd and Ni, and the 6-31G** basis sets[7] were used for C, N, Si, Cl and hydrogen atoms.

Supplementary Table 1. Actual bond distances, calculated bond distances and Wiberg bond index for **1a** and **1a**$_{\mathbf{opt}}$.

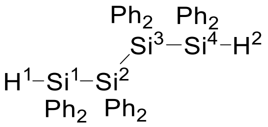

	Actual bond distances (Å)[8]	Estimated bond distances (Å)	Wiberg bond index
Si(1)-Si(2)	2.3592(4)	2.3801	0.9171
Si(2)-Si(3)	2.3661(6)	2.3952	0.9074
Si(3)-Si(4)	2.3592(4)	2.3801	0.9171
Si(1)-H(1)	1.38(2)	1.4951	0.9227
Si(4)-H(2)	1.38(2)	1.4951	0.9227

Supplementary Table 2. Actual bond distances, calculated bond distances and Wiberg bond index for **1b** and **1b**$_{\mathbf{opt}}$.

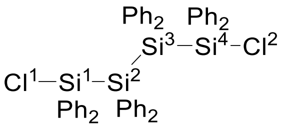

	Actual bond distances (Å)[9]	Estimated bond distances (Å)	Wiberg bond index
Si(1)-Si(2)	2.389(2)	2.4000	0.8946
Si(2)-Si(3)	2.374(2)	2.3961	0.9104
Si(3)-Si(4)	2.389(2)	2.4000	0.8946
Si(1)-Cl(1)	2.077(2)	2.1049	0.8223
Si(4)-Cl(2)	2.077(2)	2.1049	0.8223

Supplementary Table 3. Actual bond distances, calculated bond distances and Wiberg bond index for **2** and **2**$_{\mathbf{opt}}$.

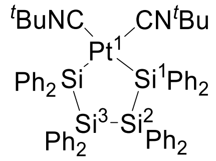

	Actual bond distances (Å)	Estimated bond distances (Å)	Wiberg bond index
Pt(1)-Si(1)	2.4249(12)	2.4391	0.6997
Si(1)-Si(2)	2.4238(17)	2.4004	0.8870
Si(2)-Si(3)	2.3108(15)	2.3709	0.9230

Supplementary Table 4. Actual bond distances, calculated bond distances and Wiberg bond index for **5a** and **5a**$_{\mathbf{opt}}$.

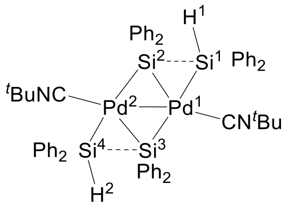

	Actual bond distances (Å)	Estimated bond distances (Å)	Wiberg bond index
Pd(1)-Pd(2)	2.7467(4)	2.8045	0.1842
Pd(1)-Si(1)	2.4050(5)	2.4519	0.4843
Pd(1)-Si(2)	2.2907(5)	2.3315	0.5816
Pd(1)-Si(3)	2.4209(5)	2.4508	0.4799
Si(1)-Si(2)	2.8324(4)	2.8592	0.3986
Si(1)-H(1)	1.344(15)	1.5028	0.8808

Supplementary Table 5. Actual bond distances, calculated bond distances and Wiberg bond index for **5b** and **5b$_{opt}$**.

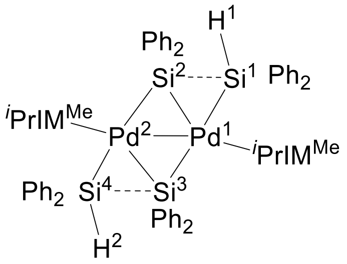

	Actual bond distances (Å)	Estimated bond distances (Å)	Wiberg bond index
Pd(1)-Pd(2)	2.8297(4)	2.8957	0.1602
Pd(1)-Si(1)	2.4487(7)	2.4754	0.4424
Pd(1)-Si(2)	2.2970(7)	2.3370	0.5288
Pd(1)-Si(3)	2.4086(7)	2.4360	0.4674
Pd(2)-Si(2)	2.4069(7)	2.4361	0.4673
Pd(2)-Si(3)	2.2955(7)	2.3370	0.5288
Pd(2)-Si(4)	2.4224(7)	2.4755	0.4424
Si(1)-Si(2)	2.8602(8)	2.8988	0.4046
Si(3)-Si(4)	2.9323(8)	2.8988	0.4046
Si(1)-H(1)	1.41(2)	1.5036	0.8734
Si(4)-H(2)	1.39(2)	1.5036	0.8734

Supplementary Table 6. Actual bond distances, calculated bond distances and Wiberg bond index for **6** and **6$_{opt}$**.

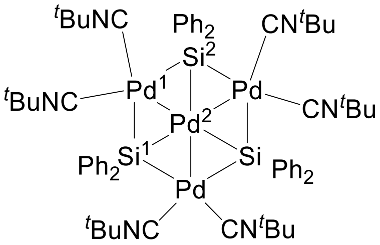

	Actual bond distances (Å)	Estimated bond distances (Å)	Wiberg bond index
Pd(1)-Pd(2)	2.7433(5)	2.7925	0.1920
Pd(1)-Si(1)	2.5305(8)	2.5825	0.4189
Pd(1)-Si(2)	2.5550(10)	2.5822	0.4191
Pd(2)-Si(1)	2.2760(8)	2.3009	0.5805

Supplementary Table 7. Actual bond distances, calculated bond distances and Wiberg bond index for **7** and **7opt**.

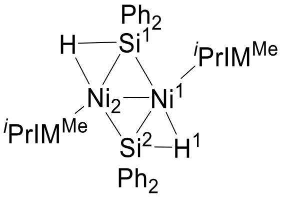

	Actual bond distances (Å)	Estimated bond distances (Å)	Wiberg bond index
Ni(1)-Ni(2)	2.5112(9)	2.5371	0.2649
Ni(1)-Si(1)	2.1940(9)	2.2051	0.6324
Ni(1)-Si(2)	2.2388(10)	2.2615	0.4490
Ni(1)-H(1)	1.59(3)	1.608	0.2975
Si(1)-H(1)	1.70(4)	1.679	0.5533

Supplementary Table 8. Actual bond distances, calculated bond distances and Wiberg bond index for **8** and **8opt**.

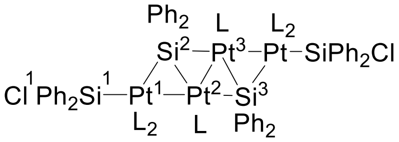

	Actual bond distances (Å)	Estimated bond distances (Å)	Wiberg bond index
Pt(1)-Pt(2)	2.7058(4)	2.78247	0.3147
Pt(2)-Pt(3)	2.7233(5)	2.80639	0.2268
Pt(1)-Si(1)	2.3496(11)	2.40294	0.7245
Pt(1)-Si(2)	2.8497(10)	2.92283	0.3107
Pt(2)-Si(2)	2.3775(9)	2.40850	0.5852
Pt(2)-Si(3)	2.3693(10)	2.41486	0.5674
Si(1)-Cl(1)	2.1529(17)	2.14701	0.7553

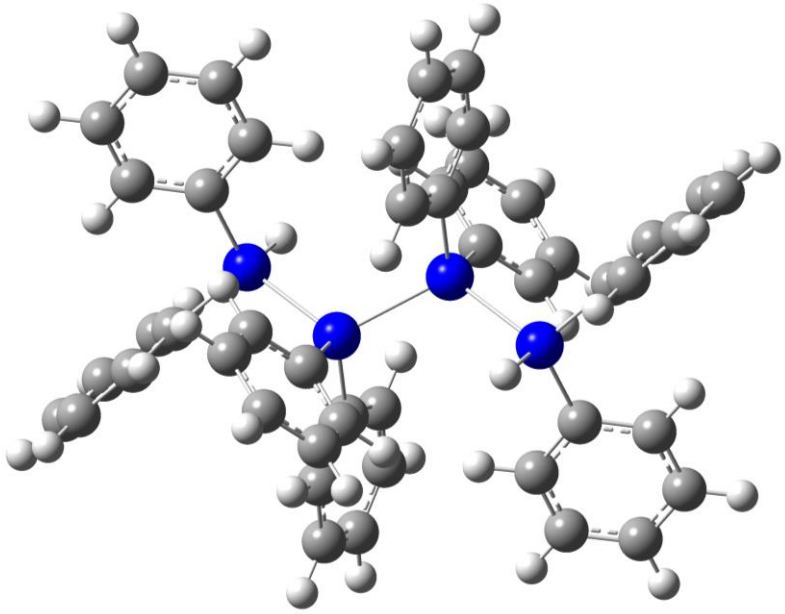

Supplementary Fig. 30. Optimized molecular structure of **1a** (**1a$_{opt}$**) with B3PW91 functional. The blue balls are silicon atoms, the grey balls are carbon atoms, and the white balls are hydrogen atoms.

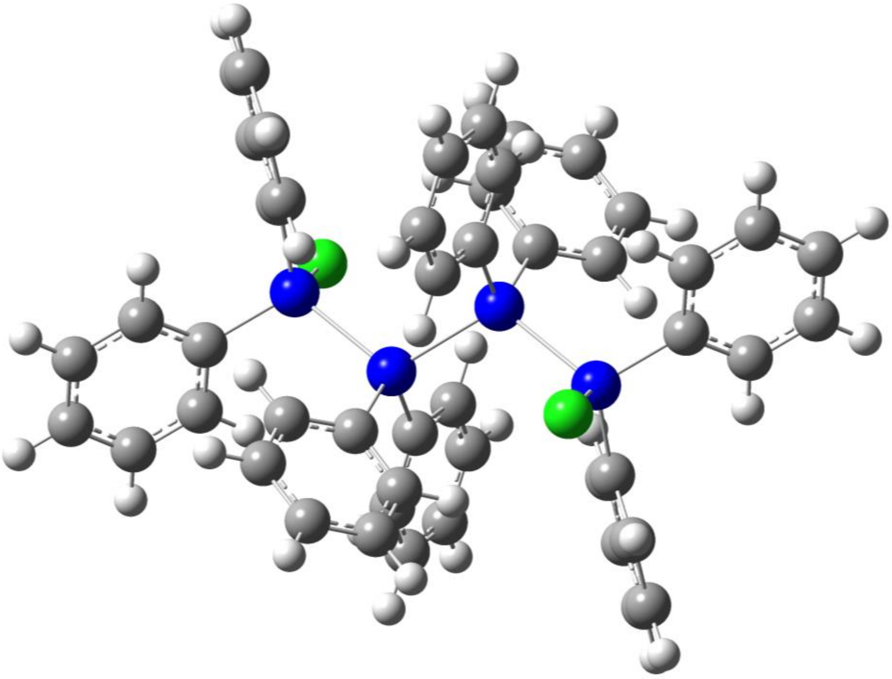

Supplementary Fig. 31. Optimized molecular structure of **1b** (**1b$_{opt}$**) with B3PW91 functional. The blue balls are silicon atoms, green balls are chlorine atoms, the grey balls are carbon atoms, and the white balls are hydrogen atoms.

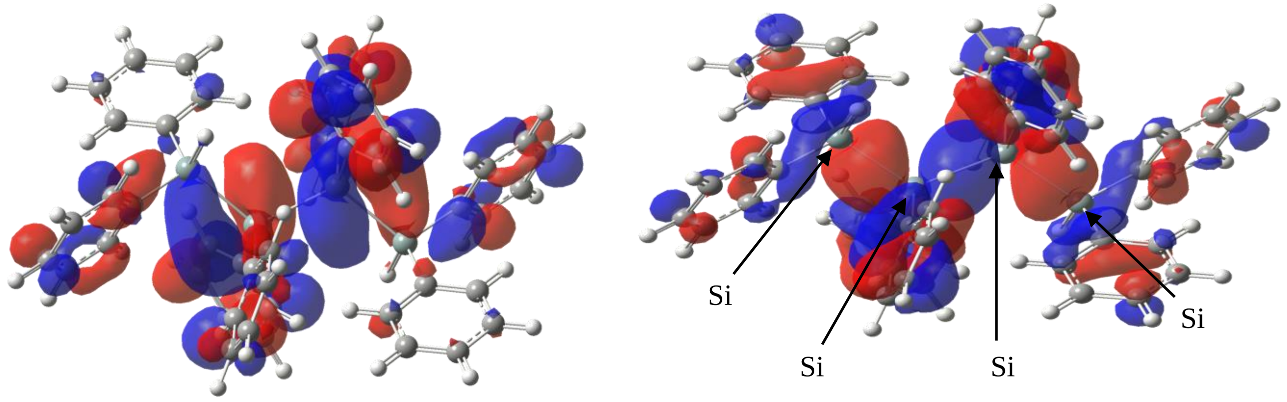

LUMO (-0.94 eV) HOMO (-5.82 eV)

Supplementary Fig. 32. Orbital figures (LUMO and HOMO) for $\mathbf{1a}_{\mathbf{opt}}$ depicted with the isovalue of 0.03 calculated by B3PW91 functional. Orbital figures of LUMO (left). Orbital figures of HOMO (right).

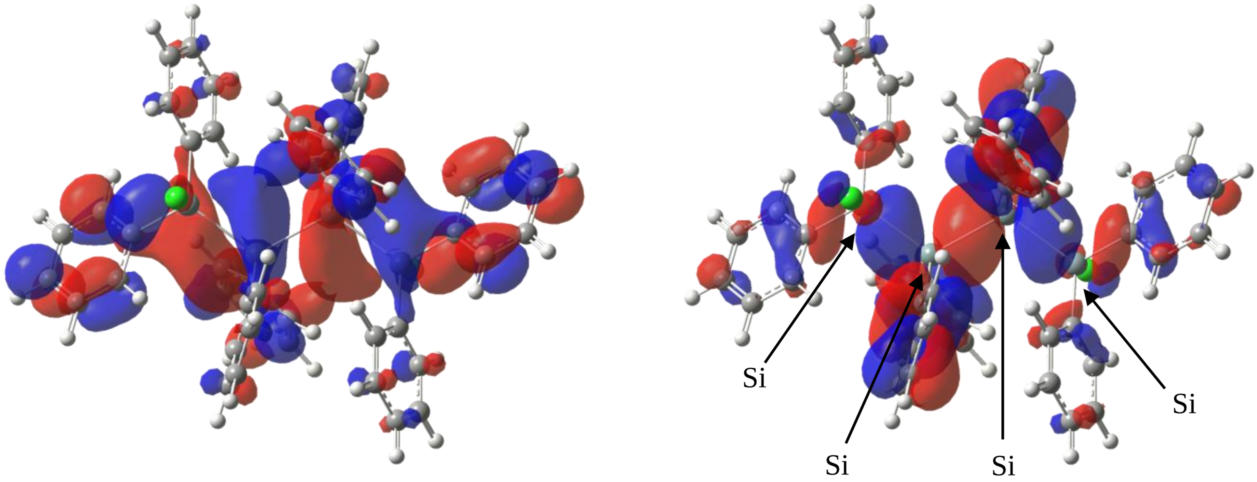

LUMO (-1.06 eV) HOMO (-6.02 eV)

Supplementary Fig. 33. Orbital figures (LUMO and HOMO) for $\mathbf{1b}_{\mathbf{opt}}$ depicted with the isovalue of 0.03 calculated by B3PW91 functional. Orbital figures of LUMO (left). Orbital figures of HOMO (right).

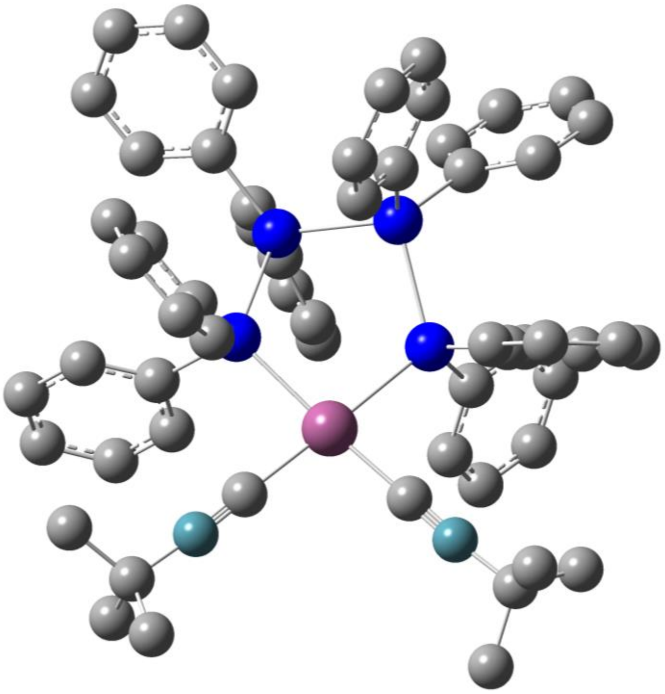

Supplementary Fig. 34. Optimized molecular structure of **2** (**2_{opt}**) with B3PW91 functional. The pale purple balls are platinum atoms, the blue balls are silicon atoms, the pale blue balls are nitrogen atoms, the grey balls are carbon atoms.

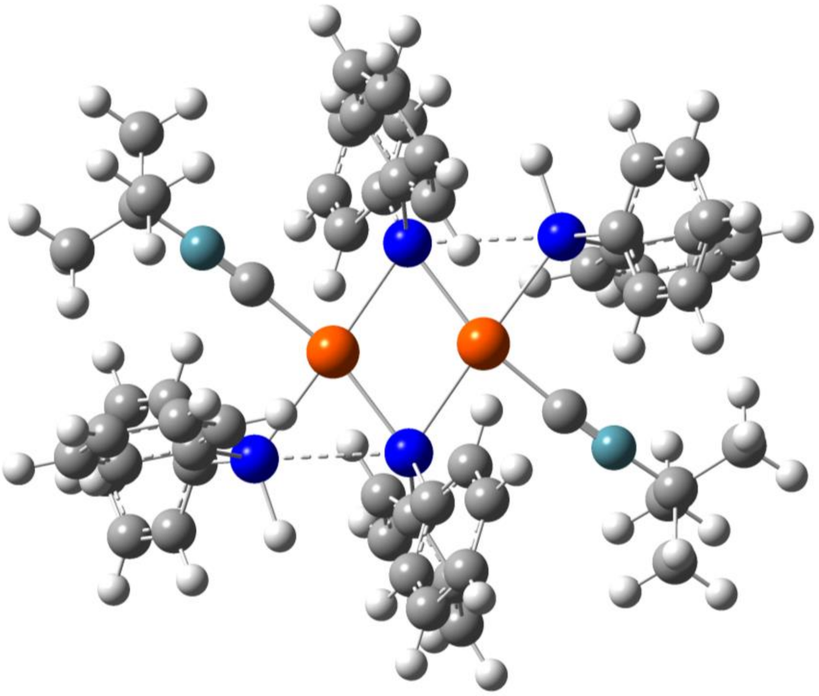

Supplementary Fig. 35. Optimized molecular structure of **5a** (**5a**$_{\mathbf{opt}}$) with B3PW91 functional. The orange balls are palladium atoms, the blue balls are silicon atoms, the pale blue balls are nitrogen atoms, the grey balls are carbon atoms, and the white balls are hydrogen atoms.

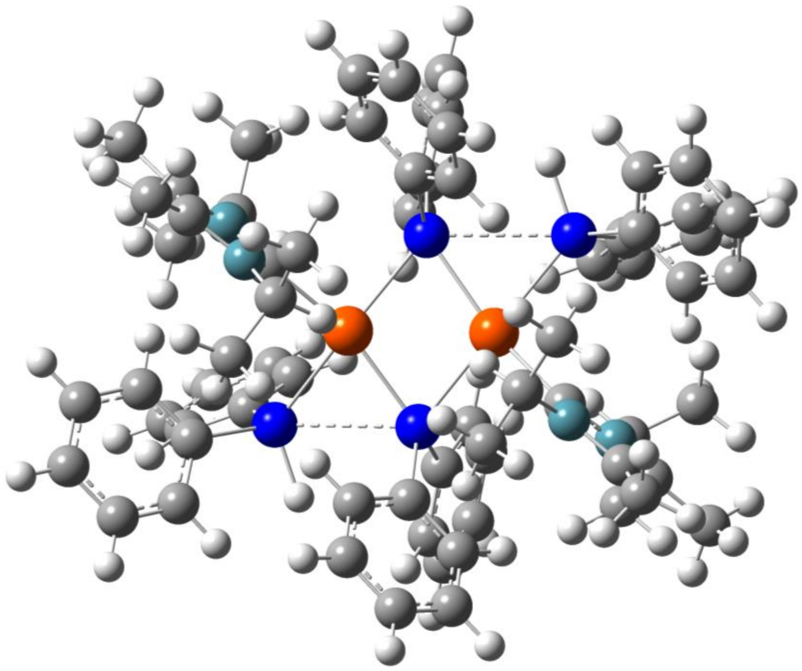

Supplementary Fig. 36. Optimized molecular structure of **5b** (**5b**$_{\mathbf{opt}}$) with B3PW91 functional. The orange balls are palladium atoms, the blue balls are silicon atoms, the pale blue balls are nitrogen atoms, the grey balls are carbon atoms, and the white balls are hydrogen atoms.

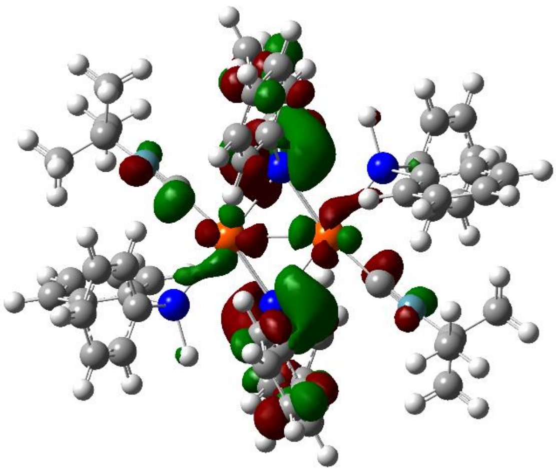

LUMO (-1.32 eV)

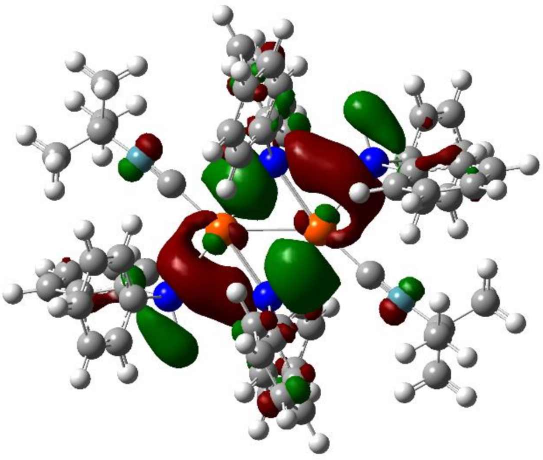

HOMO (-5.81 eV)

Supplementary Fig. 37. Orbital figures (LUMO and HOMO) for $\mathbf{5a}_{\mathbf{opt}}$ depicted with the isovalue of 0.03 calculated by B3PW91 functional. Orbital figures of LUMO (left). Orbital figures of HOMO (right).

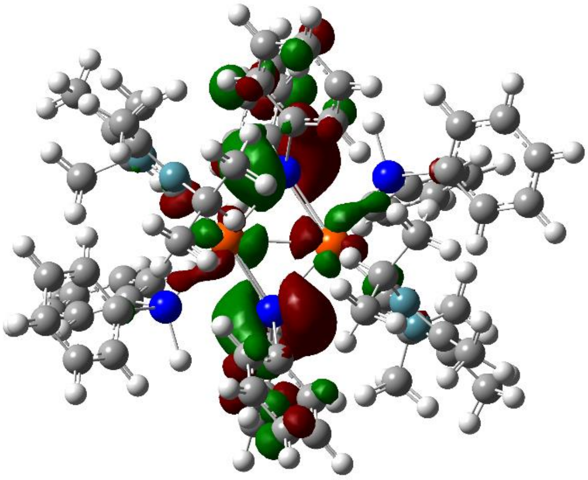

LUMO (-1.19 eV)

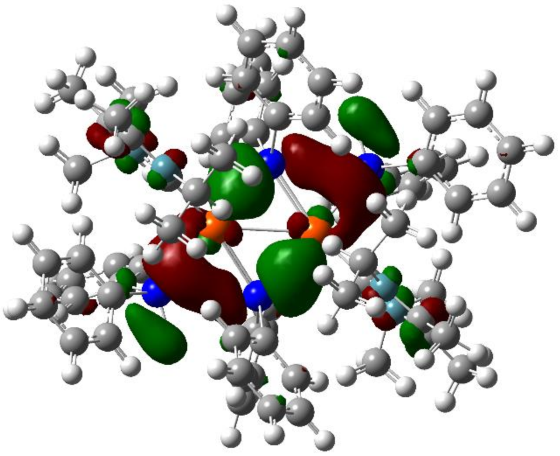

HOMO (-4.65 eV)

Supplementary Fig. 38. Orbital figures (LUMO and HOMO) for $\mathbf{5b}_{\mathbf{opt}}$ depicted with the isovalue of 0.03 calculated by B3PW91 functional. Orbital figures of LUMO (left). Orbital figures of HOMO (right).

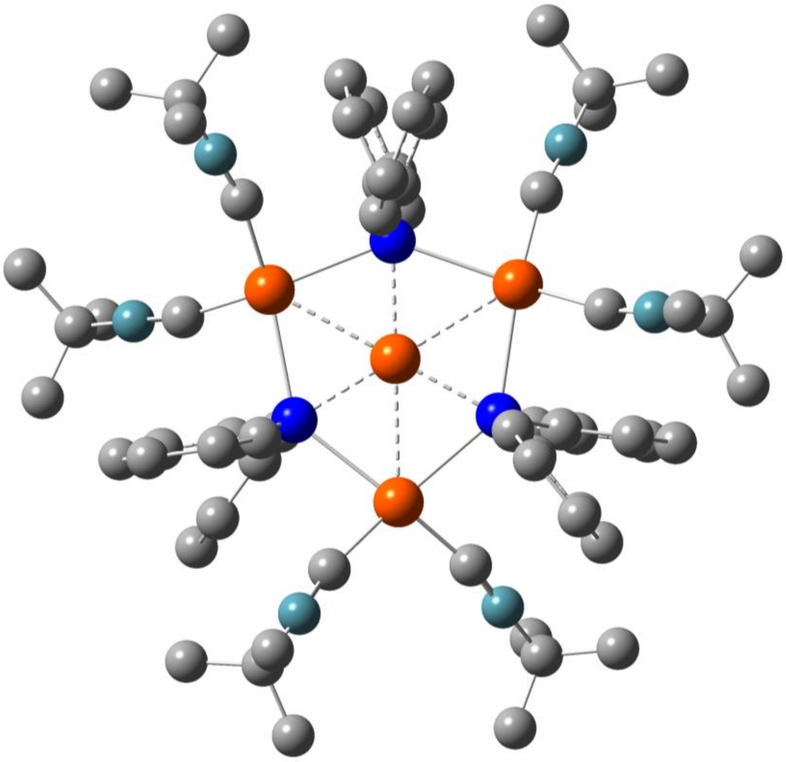

Supplementary Fig. 39. Optimized molecular structure of **6** (**6_{opt}**) with B3PW91 functional. The orange balls are palladium atoms, the blue balls are silicon atoms, the pale blue balls are nitrogen atoms, the grey balls are carbon atoms.

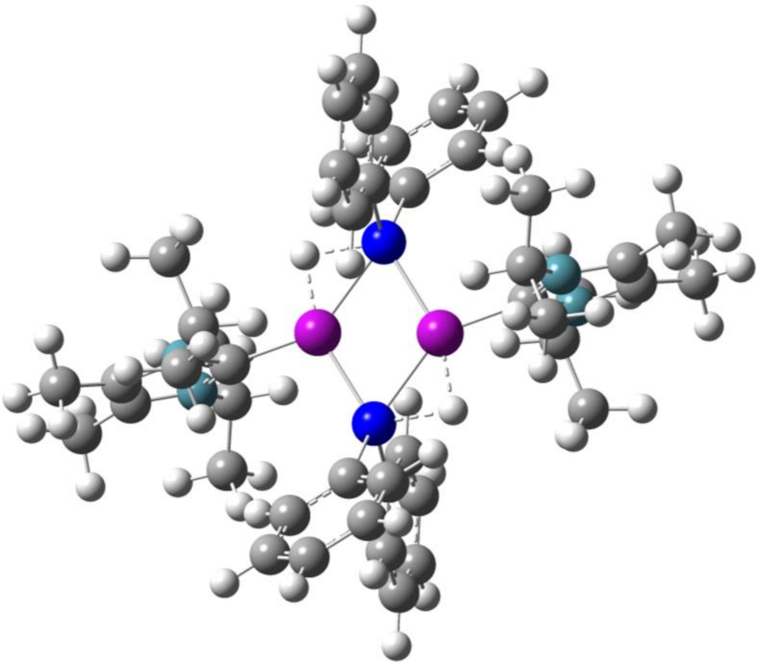

Supplementary Fig. 40. Optimized molecular structure of **7** ($\mathbf{7_{opt}}$) with B3PW91 functional. The purple balls are nickel atoms, the blue balls are silicon atoms, the pale blue balls are nitrogen atoms, the grey balls are carbon atoms, and the white balls are hydrogen atoms.

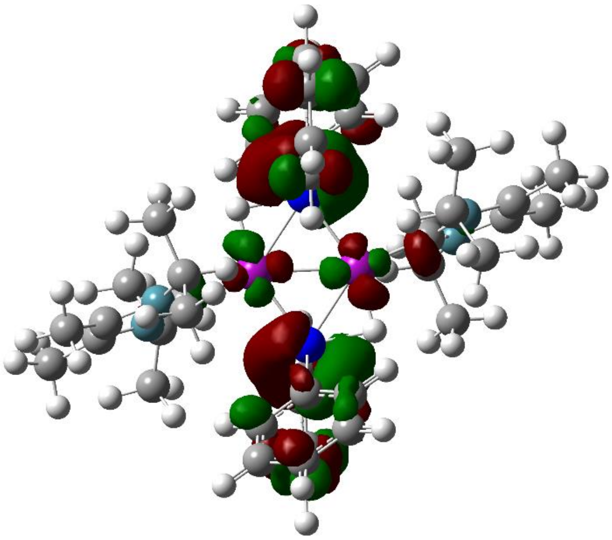

LUMO (-0.62 eV)

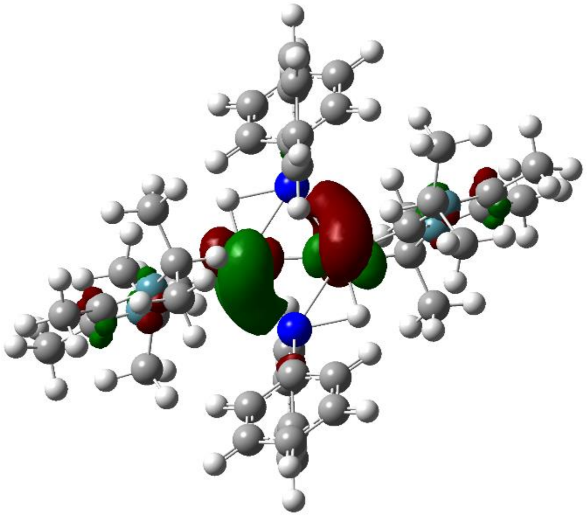

HOMO (-4.41 eV)

Supplementary Fig. 41. Orbital figures (LUMO and HOMO) for $\mathbf{7_{opt}}$ depicted with the isovalue of 0.03 calculated by B3PW91 functional. Orbital figures of LUMO (left). Orbital figures of HOMO (right).

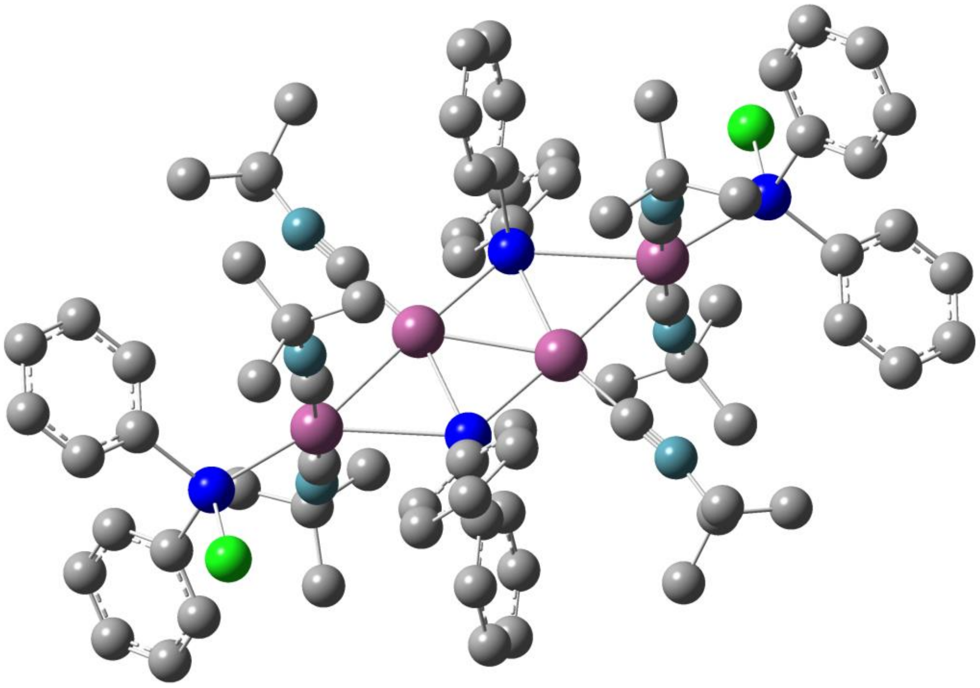

Supplementary Fig. 42. Optimized molecular structure of **8** ($\mathbf{8_{opt}}$) with B3PW91 functional. The pale purple balls are platinum atoms, the blue balls are silicon atoms, the pale blue balls are nitrogen atoms, the grey balls are carbon atoms.

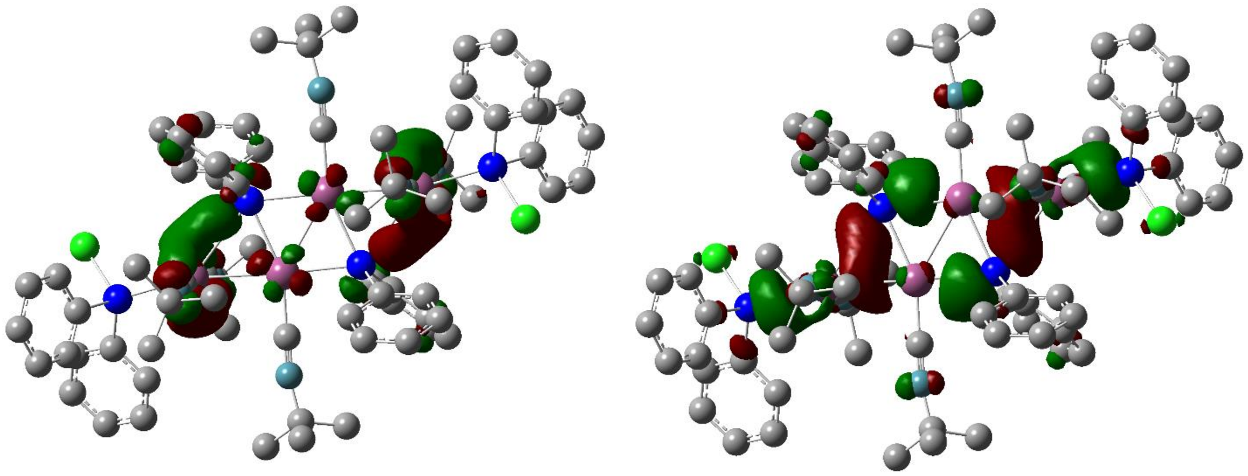

LUMO (-1.32 eV) HOMO (-4.16 eV)

Supplementary Fig. 43. Orbital figures (LUMO and HOMO) for $\mathbf{8_{opt}}$ depicted with the isovalue of 0.03 calculated by B3PW91 functional. Orbital figures of LUMO (left). Orbital figures of HOMO (right).

Supplementary Note 3

X-ray data collection and reduction X-ray crystallography for compound **2**, **3**, **4**, **6**, **7** and **8** was performed on a Rigaku Saturn CCD area detector with graphite monochromated Mo-Kα radiation (λ=0.71075 Å). Single crystals of **5a** and **5b** suitable for X-ray crystallography were analyzed by synchrotron radiation at beam line BL02B1 (λ=0.41340 Å for **5a**, 0.41350 Å for **5b**, 0.41330 Å for **7**) of Spring-8 (Hyogo, Japan) using PILATUS3 X CdTe 1M detector. The data were collected at 100(1) K for **5a, 5b**, **8**, 113(2)K for **2**, 123(1) K for **6, 3**, **4**, **7**, using ω scan in the θ range of 3.12≤ θ≤ 27.48 deg for **2**, 3.03≤ θ≤ 27.48 deg for **3**, 3.19≤ θ≤ 27.48 deg for **4**, 0.59≤ θ≤ 15.66 deg for **5a**, 0.47≤ θ≤ 15.64 deg for **5b**, 1.94≤ θ≤ 31.19 deg for **6**, 3.07≤ θ≤ 27.48 deg for **7** and 0.94≤ θ≤ 15.57 deg for **8**. The data obtained were processed using Crystal-Clear (Rigaku) on a Pentium computer, and were corrected for Lorentz and polarization effects. The structures were solved by direct methods[10], and expanded using Fourier techniques. Hydrogen atoms were refined using the riding model. The final cycle of full-matrix least-squares refinement on F^2 was based on 6025 observed reflections and 297 variable parameters for **2**, 15856 observed reflections and 732 variable parameters for **3**, 9593 observed reflections and 490 variable parameters for **4**, 6076 observed reflections and 305 variable parameters for **5a**, 17123 observed reflections and 1239 variable parameters for **5b**, 9541 observed reflections and 244 variable parameters for **6**, 4993 observed reflections and 254 variable parameters for **7**, and 9,323 observed reflections and 430 variable parameters for **8**. Neutral atom scattering factors were taken from International Tables for Crystallography (IT), Vol. C, Table 6.1.1.4[11]. Anomalous dispersion effects were included in Fcalc[12]; the values for Δf' and Δf" were those of Creagh and McAuley[13]. The values for the mass attenuation coefficients are those of Creagh and Hubbell[14]. All calculations were performed using the CrystalStructure[15] crystallographic software package except for refinement, which was performed using SHELXL Version 2017/1[16]. Details of final refinement as well as the bond lengths and angle are summarized in Supplementary Tables 9 – 16, and the numbering scheme employed is also shown in Supplementary Figs. 44-51, which were drawn with ORTEP at 50% probability ellipsoids. CCDC 2231760 (**2**), 2231758 (**3**), 2231757 (**4**), 2231762 (**5a**), 2231763 (**5b**), 2231761 (**6**), 2231756 (**7**) and 2231759 (**8**) contains the supplementary crystallographic data.

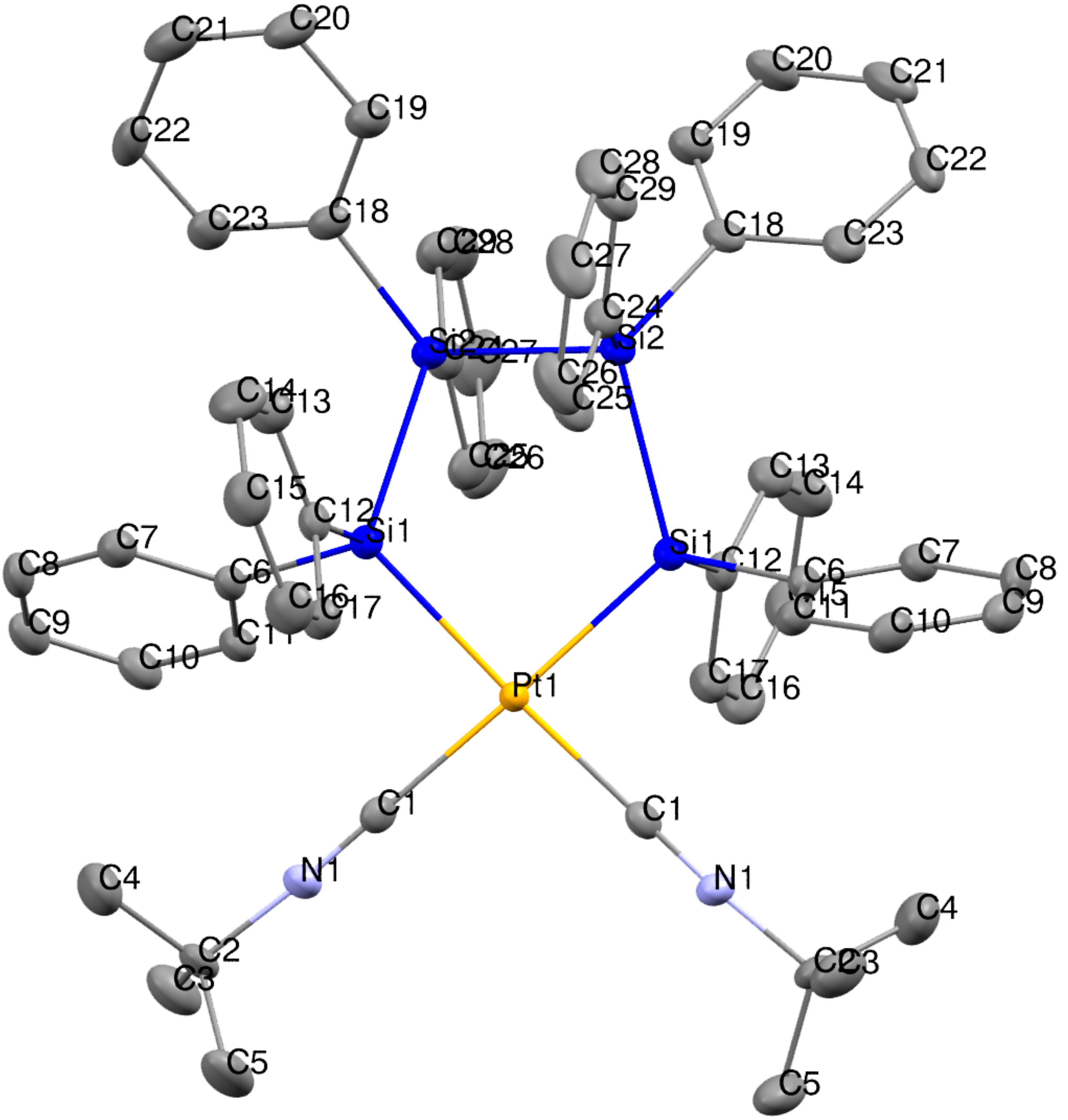

Supplementary Fig. 44. ORTEP drawing of **2** (50% probability of the thermal ellipsoids). Hydrogen atoms were omitted for clarity.

Supplementary Table 9. Crystal data and structure refinement for **2**.

Empirical Formula	$C_{58}H_{58}N_2PtSi_4$
Formula Weight	1090.54
Crystal Color, Habit	yello, platelet
Crystal Dimensions	0.150 X 0.050 X 0.020 mm
Crystal System	orthorhombic
Lattice Type	Primitive
Lattice Parameters	a = 14.903(6) Å
	b = 15.292(6) Å
	c = 23.077(9) Å
	V = 5259(4) Å^3
Space Group	Pbcn (#60)
Z value	4
D_{calc}	1.377 g/cm^3
F_{000}	2216.00
μ(MoKα)	27.869 cm^{-1}
Diffractometer	Saturn724
Radiation	MoKα (λ = 0.71075 Å)
	multi-layer mirror monochromated
Voltage, Current	50kV, 24mA
Temperature	-159.8^{o}C
Detector Aperture	72.8 x 72.8 mm
Data Images	720 exposures
ω oscillation Range (χ=45.0, ϕ=0.0)	-70.0 - 110.0^o
Exposure Rate	10.0 sec./^{o}
Detector Swing Angle	19.89^o
ω oscillation Range (χ=45.0, ϕ=90.0)	-70.0 - 110.0^o
Exposure Rate	10.0 sec./^{o}
Detector Swing Angle	19.89^o
Detector Position	44.58 mm
Pixel Size	0.141 mm
$2\theta_{max}$	56.3^o
No. of Reflections Measured	Total: 6025
	Unique: 6025 (R_{int} = 0.0729)
Corrections	Lorentz-polarization
	Absorption
	(trans. factors: 0.807 - 0.946)
Structure Solution	Direct Methods (SIR2008)

Refinement	Full-matrix least-squares on F^2
Function Minimized	Σ w $(Fo^2 - Fc^2)^2$
Least Squares Weights	w = 1/ [$\sigma^2(Fo^2) + (0.0366 \cdot P)^2$ + 1.4732 · P] where P = $(Max(Fo^2,0) + 2Fc^2)/3$
$2\theta_{max}$ cutoff	55.0°
Anomalous Dispersion	All non-hydrogen atoms
No. Observations (All reflections)	6025
No. Variables	297
Reflection/Parameter Ratio	20.29
Residuals: R1 (I>2.00σ(I))	0.0429
Residuals: R (All reflections)	0.0632
Residuals: wR2 (All reflections)	0.0933
Goodness of Fit Indicator	1.133
Max Shift/Error in Final Cycle	0.000
Maximum peak in Final Diff. Map	0.82 $e^-/Å^3$
Minimum peak in Final Diff. Map	-0.77 $e^-/Å^3$

Atomic coordinates and B_{iso}/B_{eq}

atom	x	y	z	B_{eq}	occ
Pt1	0.00000	0.24762(2)	0.25000	1.005(6)	1/2
Si1	0.02008(7)	0.13655(7)	0.32387(4)	1.230(17)	1
Si2	0.04815(7)	-0.01057(7)	0.28924(4)	1.282(17)	1
N1	0.0536(2)	0.3884(2)	0.34567(13)	1.63(6)	1
C1	0.0326(3)	0.3376(3)	0.31173(16)	1.44(6)	1
C2	0.0834(3)	0.4491(3)	0.39187(16)	1.82(7)	1
C3	0.1811(3)	0.4603(3)	0.3865(2)	3.01(9)	1
C4	0.0595(4)	0.4023(3)	0.44781(18)	3.37(10)	1
C5	0.0367(4)	0.5367(3)	0.3843(2)	3.25(10)	1
C6	0.1134(3)	0.1623(3)	0.37583(15)	1.38(6)	1
C7	0.1067(3)	0.1422(3)	0.43514(16)	1.75(7)	1
C8	0.1765(3)	0.1583(3)	0.47234(17)	2.01(8)	1
C9	0.2541(3)	0.1927(3)	0.45206(17)	2.01(7)	1
C10	0.2623(3)	0.2129(3)	0.39374(17)	1.95(7)	1
C11	0.1920(3)	0.1985(3)	0.35692(16)	1.55(7)	1
C12	-0.0841(3)	0.1354(3)	0.36948(15)	1.46(6)	1
C13	-0.1151(3)	0.0596(3)	0.39852(16)	1.92(7)	1
C14	-0.1931(3)	0.0617(3)	0.43008(18)	2.58(9)	1
C15	-0.2422(3)	0.1395(3)	0.43268(17)	2.41(8)	1
C16	-0.2123(3)	0.2152(3)	0.40420(18)	2.36(8)	1
C17	-0.1351(3)	0.2132(3)	0.37373(16)	1.78(7)	1
C18	0.0321(3)	-0.1016(3)	0.34497(16)	1.49(6)	1
C19	-0.0247(3)	-0.1738(3)	0.33709(18)	2.10(8)	1
C20	-0.0408(3)	-0.2354(3)	0.38066(19)	2.36(8)	1
C21	0.0007(3)	-0.2266(3)	0.4333(2)	2.55(9)	1
C22	0.0580(3)	-0.1563(3)	0.44276(16)	2.19(8)	1
C23	0.0734(3)	-0.0957(3)	0.39878(16)	1.81(7)	1
C24	0.1631(3)	-0.0198(3)	0.26070(16)	1.64(7)	1
C25	0.2080(3)	0.0543(3)	0.23828(17)	1.95(7)	1
C26	0.2915(3)	0.0456(3)	0.21529(19)	2.87(9)	1
C27	0.3311(3)	-0.0376(4)	0.21321(19)	3.01(10)	1
C28	0.2876(3)	-0.1132(3)	0.23463(18)	2.69(9)	1
C29	0.2047(3)	-0.1036(3)	0.25817(16)	2.04(8)	1

$B_{eq} = 8/3\ \pi^2(U_{11}(aa^*)^2 + U_{22}(bb^*)^2 + U_{33}(cc^*)^2 + 2U_{12}(aa^*bb^*)\cos\gamma + 2U_{13}(aa^*cc^*)\cos\beta + 2U_{23}(bb^*cc^*)\cos\alpha)$

Anisotropic displacement parameters

atom	U_{11}	U_{22}	U_{33}	U_{12}	U_{13}	U_{23}
Pt1	0.01264(11)	0.01249(10)	0.01305(11)	0.00000	-0.00097(8)	0.00000
Si1	0.0151(6)	0.0162(5)	0.0155(5)	0.0009(4)	0.0000(4)	-0.0001(4)
Si2	0.0153(6)	0.0151(5)	0.0183(5)	0.0016(4)	-0.0003(5)	0.0009(4)
N1	0.024(2)	0.0161(17)	0.0216(16)	0.0010(14)	-0.0002(15)	-0.0027(14)
C1	0.0126(19)	0.020(2)	0.022(2)	0.0011(15)	-0.0042(16)	0.0038(16)
C2	0.028(2)	0.017(2)	0.024(2)	0.0004(17)	-0.0024(18)	-0.0076(16)
C3	0.032(3)	0.035(3)	0.048(3)	-0.003(2)	-0.007(2)	-0.015(2)
C4	0.060(4)	0.039(3)	0.029(2)	-0.003(3)	-0.004(2)	-0.007(2)
C5	0.047(3)	0.028(3)	0.048(3)	0.010(2)	-0.015(3)	-0.014(2)
C6	0.018(2)	0.017(2)	0.0179(19)	0.0023(15)	-0.0054(16)	-0.0011(15)
C7	0.022(2)	0.021(2)	0.023(2)	0.0012(17)	-0.0007(17)	0.0003(17)
C8	0.030(3)	0.028(2)	0.018(2)	0.0030(19)	-0.0089(18)	-0.0018(17)
C9	0.021(2)	0.025(2)	0.030(2)	0.0032(18)	-0.0093(18)	-0.0063(18)
C10	0.019(2)	0.025(2)	0.031(2)	-0.0025(17)	-0.0029(18)	-0.0066(19)
C11	0.021(2)	0.018(2)	0.020(2)	-0.0018(16)	-0.0007(17)	-0.0022(16)
C12	0.017(2)	0.023(2)	0.0159(19)	-0.0010(16)	-0.0018(16)	-0.0010(16)
C13	0.021(2)	0.025(2)	0.027(2)	0.0032(17)	0.0037(18)	0.0017(18)
C14	0.030(3)	0.029(3)	0.039(3)	-0.001(2)	0.015(2)	0.009(2)
C15	0.022(2)	0.042(3)	0.028(2)	0.004(2)	0.0082(19)	-0.003(2)
C16	0.023(2)	0.038(3)	0.029(2)	0.007(2)	0.0087(19)	-0.003(2)
C17	0.024(2)	0.024(2)	0.019(2)	0.0026(18)	0.0039(18)	0.0017(17)
C18	0.017(2)	0.017(2)	0.023(2)	0.0047(16)	0.0063(17)	0.0032(15)
C19	0.028(2)	0.021(2)	0.031(2)	0.0007(18)	-0.0035(19)	0.0024(18)
C20	0.026(3)	0.023(2)	0.040(3)	-0.0000(18)	0.003(2)	0.0110(19)
C21	0.035(3)	0.026(2)	0.036(3)	0.011(2)	0.015(2)	0.0113(19)
C22	0.033(3)	0.032(3)	0.018(2)	0.015(2)	-0.0005(19)	0.0025(17)
C23	0.020(2)	0.022(2)	0.026(2)	0.0067(17)	0.0018(17)	0.0000(17)
C24	0.017(2)	0.024(2)	0.021(2)	0.0030(17)	0.0013(16)	-0.0014(16)
C25	0.015(2)	0.030(2)	0.028(2)	0.0047(18)	-0.0022(17)	0.0053(18)
C26	0.020(2)	0.053(3)	0.036(3)	0.003(2)	0.002(2)	0.017(2)
C27	0.023(3)	0.061(4)	0.031(2)	0.015(2)	0.011(2)	0.008(2)
C28	0.030(3)	0.038(3)	0.034(2)	0.018(2)	0.002(2)	-0.002(2)
C29	0.024(2)	0.026(2)	0.028(2)	0.0047(18)	-0.0046(18)	0.0027(18)

The general temperature factor expression: $\exp(-2\pi^2(a^{*2}U_{11}h^2 + b^{*2}U_{22}k^2 + c^{*2}U_{33}l^2 + 2a^*b^*U_{12}hk + 2a^*c^*U_{13}hl + 2b^*c^*U_{23}kl))$

Bond lengths (Å)

atom	atom	distance	atom	atom	distance
Pt1	Si1	2.4249(12)	Pt1	Si1[1]	2.4249(12)
Pt1	C1	2.039(4)	Pt1	C1[1]	2.039(4)
Si1	Si2	2.4238(17)	Si1	C6	1.878(4)
Si1	C12	1.875(4)	Si2	Si2[1]	2.3108(15)
Si2	C18	1.911(4)	Si2	C24	1.841(4)
N1	C1	1.147(5)	N1	C2	1.481(5)
C2	C3	1.472(6)	C2	C4	1.518(6)
C2	C5	1.520(6)	C6	C7	1.406(5)
C6	C11	1.368(5)	C7	C8	1.371(6)
C8	C9	1.355(6)	C9	C10	1.386(6)
C10	C11	1.367(6)	C12	C13	1.418(6)
C12	C17	1.414(6)	C13	C14	1.371(6)
C14	C15	1.398(7)	C15	C16	1.404(7)
C16	C17	1.349(6)	C18	C19	1.402(6)
C18	C23	1.389(5)	C19	C20	1.399(6)
C20	C21	1.370(7)	C21	C22	1.390(6)
C22	C23	1.394(6)	C24	C25	1.413(6)
C24	C29	1.425(6)	C25	C26	1.359(6)
C26	C27	1.403(7)	C27	C28	1.414(7)
C28	C29	1.358(6)			

Symmetry Operators:

(1) -X,Y,-Z+1/2

Bond angles (^{o})

atom	atom	atom	angle	atom	atom	atom	angle
Si1	Pt1	Si1[1]	91.08(5)	Si1	Pt1	C1	87.25(12)
Si1	Pt1	C1[1]	173.25(11)	Si1[1]	Pt1	C1	173.25(11)
Si1[1]	Pt1	C1[1]	87.25(12)	C1	Pt1	C1[1]	95.15(16)
Pt1	Si1	Si2	116.08(5)	Pt1	Si1	C6	113.16(13)
Pt1	Si1	C12	107.38(13)	Si2	Si1	C6	106.11(13)
Si2	Si1	C12	108.64(13)	C6	Si1	C12	104.84(17)
Si1	Si2	Si2[1]	98.70(5)	Si1	Si2	C18	115.65(13)
Si1	Si2	C24	110.45(14)	Si2[1]	Si2	C18	116.72(13)
Si2[1]	Si2	C24	107.32(13)	C18	Si2	C24	107.57(18)
C1	N1	C2	176.1(4)	Pt1	C1	N1	177.8(3)
N1	C2	C3	108.0(3)	N1	C2	C4	104.3(3)
N1	C2	C5	109.4(3)	C3	C2	C4	111.0(4)

C3	C2	C5	109.9(4)	C4	C2	C5	113.9(4)
Si1	C6	C7	121.6(3)	Si1	C6	C11	121.0(3)
C7	C6	C11	117.4(3)	C6	C7	C8	121.1(4)
C7	C8	C9	120.1(4)	C8	C9	C10	119.8(4)
C9	C10	C11	120.0(4)	C6	C11	C10	121.6(4)
Si1	C12	C13	122.9(3)	Si1	C12	C17	118.5(3)
C13	C12	C17	118.6(4)	C12	C13	C14	120.5(4)
C13	C14	C15	119.1(4)	C14	C15	C16	121.1(4)
C15	C16	C17	119.7(4)	C12	C17	C16	121.0(4)
Si2	C18	C19	124.2(3)	Si2	C18	C23	119.9(3)
C19	C18	C23	115.8(4)	C18	C19	C20	122.7(4)
C19	C20	C21	119.5(4)	C20	C21	C22	119.5(4)
C21	C22	C23	120.1(4)	C18	C23	C22	122.3(4)
Si2	C24	C25	120.7(3)	Si2	C24	C29	119.2(3)
C25	C24	C29	120.1(4)	C24	C25	C26	119.8(4)
C25	C26	C27	119.1(4)	C26	C27	C28	122.4(4)
C27	C28	C29	118.0(4)	C24	C29	C28	120.6(4)

Symmetry Operators:

(1) -X,Y,-Z+1/2

Torsion Angles(°)

(Those having bond angles > 160 or < 20 degrees are excluded.)

atom1	atom2	atom3	atom4	angle	atom1	atom2	atom3	atom4	angle
Si1	Pt1	$Si1^1$	$Si2^1$	14.64(5)	Si1	Pt1	$Si1^1$	$C6^1$	137.70(5)
Si1	Pt1	$Si1^1$	$C12^1$	-107.10(5)	$Si1^1$	Pt1	Si1	Si2	14.64(5)
$Si1^1$	Pt1	Si1	C6	137.70(5)	$Si1^1$	Pt1	Si1	C12	-107.10(5)
C1	Pt1	Si1	Si2	-158.81(12)	C1	Pt1	Si1	C6	-35.76(12)
C1	Pt1	Si1	C12	79.45(12)	$C1^1$	Pt1	$Si1^1$	$Si2^1$	-158.81(12)
$C1^1$	Pt1	$Si1^1$	$C6^1$	-35.76(12)	$C1^1$	Pt1	$Si1^1$	$C12^1$	79.45(12)
Pt1	Si1	Si2	$Si2^1$	-38.58(6)	Pt1	Si1	Si2	C18	-163.89(5)
Pt1	Si1	Si2	C24	73.66(6)	Pt1	Si1	C6	C7	142.6(2)
Pt1	Si1	C6	C11	-39.9(3)	Pt1	Si1	C12	C13	151.3(2)
Pt1	Si1	C12	C17	-26.9(3)	Si2	Si1	C6	C7	-89.0(3)
Si2	Si1	C6	C11	88.6(3)	C6	Si1	Si2	$Si2^1$	-165.25(12)
C6	Si1	Si2	C18	69.44(13)	C6	Si1	Si2	C24	-53.01(13)
Si2	Si1	C12	C13	25.0(3)	Si2	Si1	C12	C17	-153.1(2)
C12	Si1	Si2	$Si2^1$	82.49(13)	C12	Si1	Si2	C18	-42.82(14)
C12	Si1	Si2	C24	-165.27(12)	C6	Si1	C12	C13	-88.1(3)
C6	Si1	C12	C17	93.8(3)	C12	Si1	C6	C7	25.9(3)

C12	Si1	C6	C11	-156.6(3)	Si1	Si2	Si2^{1}	Si1^{1}	40.24(5)
Si1	Si2	Si2^{1}	C18^{1}	164.80(5)	Si1	Si2	Si2^{1}	C24^{1}	-74.47(6)
Si1	Si2	C18	C19	124.8(2)	Si1	Si2	C18	C23	-50.7(3)
Si1	Si2	C24	C25	-26.8(3)	Si1	Si2	C24	C29	157.1(2)
Si2^{1}	Si2	C18	C19	9.4(3)	Si2^{1}	Si2	C18	C23	-166.2(2)
C18	Si2	Si2^{1}	Si1^{1}	164.80(14)	C18	Si2	Si2^{1}	C18^{1}	-70.64(15)
C18	Si2	Si2^{1}	C24^{1}	50.09(15)	Si2^{1}	Si2	C24	C25	79.8(3)
Si2^{1}	Si2	C24	C29	-96.4(2)	C24	Si2	Si2^{1}	Si1^{1}	-74.47(15)
C24	Si2	Si2^{1}	C18^{1}	50.09(15)	C24	Si2	Si2^{1}	C24^{1}	170.82(14)
C18	Si2	C24	C25	-153.8(2)	C18	Si2	C24	C29	30.0(3)
C24	Si2	C18	C19	-111.2(3)	C24	Si2	C18	C23	73.2(3)
Si1	C6	C7	C8	177.5(2)	Si1	C6	C11	C10	-176.1(2)
C7	C6	C11	C10	1.5(5)	C11	C6	C7	C8	-0.1(5)
C6	C7	C8	C9	-1.1(6)	C7	C8	C9	C10	0.9(6)
C8	C9	C10	C11	0.4(6)	C9	C10	C11	C6	-1.7(6)
Si1	C12	C13	C14	-178.2(2)	Si1	C12	C17	C16	177.6(2)
C13	C12	C17	C16	-0.7(5)	C17	C12	C13	C14	-0.1(5)
C12	C13	C14	C15	0.8(6)	C13	C14	C15	C16	-0.8(6)
C14	C15	C16	C17	0.1(6)	C15	C16	C17	C12	0.7(6)
Si2	C18	C19	C20	-174.4(3)	Si2	C18	C23	C22	174.5(2)
C19	C18	C23	C22	-1.5(6)	C23	C18	C19	C20	1.3(6)
C18	C19	C20	C21	-0.8(6)	C19	C20	C21	C22	0.3(6)
C20	C21	C22	C23	-0.4(6)	C21	C22	C23	C18	1.0(6)
Si2	C24	C25	C26	-177.4(2)	Si2	C24	C29	C28	176.6(2)
C25	C24	C29	C28	0.4(5)	C29	C24	C25	C26	-1.2(5)
C24	C25	C26	C27	1.3(6)	C25	C26	C27	C28	-0.5(6)
C26	C27	C28	C29	-0.3(6)	C27	C28	C29	C24	0.3(6)

Symmetry Operators:

(1) -X,Y,-Z+1/2

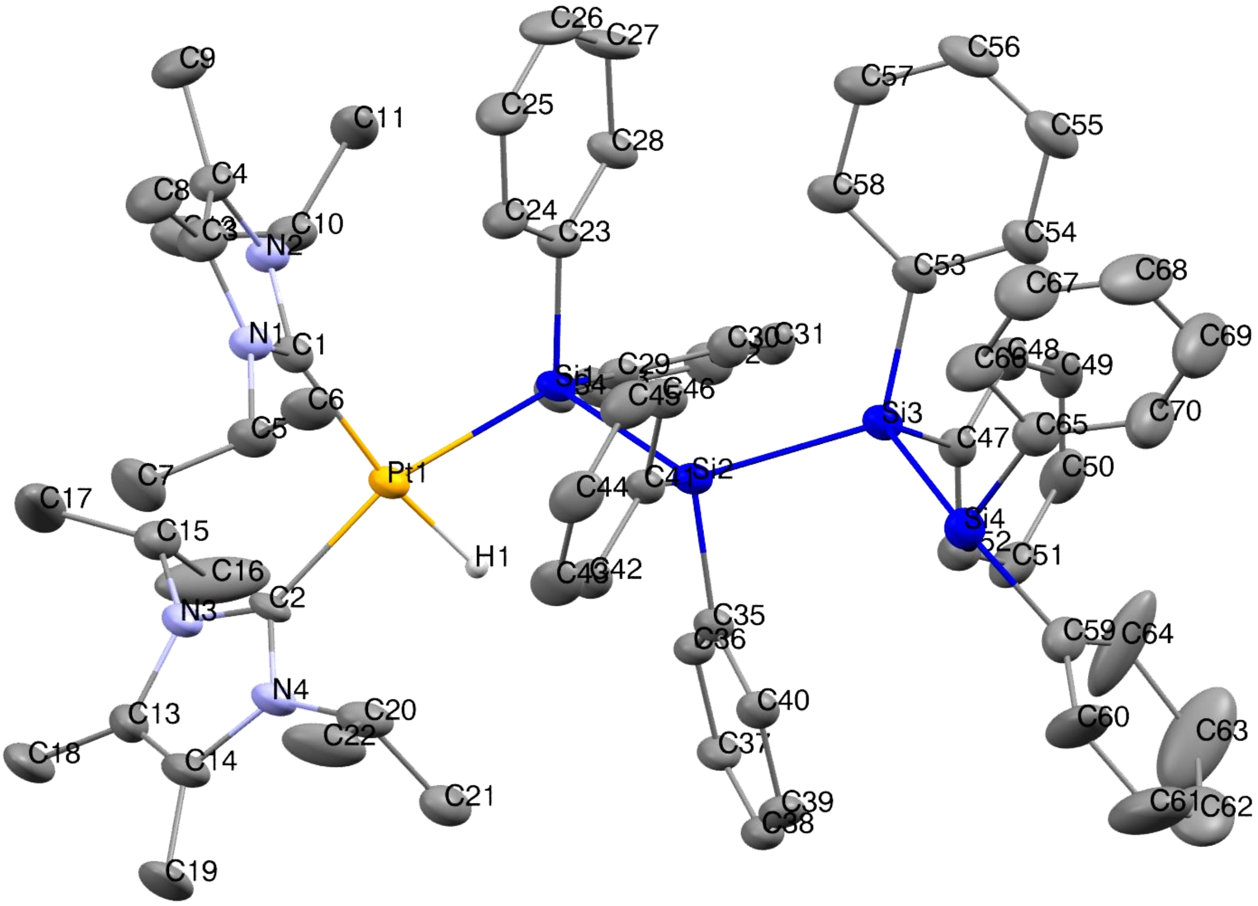

Supplementary Fig. 45. ORTEP drawing of **3** (30% probability of the thermal ellipsoids). Hydrogen atoms were omitted for clarity.

Supplementary Table 10. Crystal data and structure refinement for **3**.

Empirical Formula	$C_{60}H_{13}N_4PtSi_4$
Formula Weight	1097.22
Crystal Color, Habit	yellow, platelet
Crystal Dimensions	0.080 X 0.020 X 0.020 mm
Crystal System	triclinic
Lattice Type	Primitive
Lattice Parameters	a = 13.283(3) Å
	b = 17.520(3) Å
	c = 18.741(3) Å
	α = 61.917(8) ^{o}
	β = 69.067(10) ^{o}
	γ = 81.320(11) ^{o}
	V = 3593.0(12) $Å^3$
Space Group	P-1 (#2)
Z value	2
D_{calc}	1.014 g/cm^3
F_{000}	1070.00
μ(MoKα)	20.415 cm^{-1}
Diffractometer	Saturn724
Radiation	MoKα (λ = 0.71075 Å)
	multi-layer mirror monochromated
Voltage, Current	50kV, 24mA
Temperature	-149.8^{o}C
Detector Aperture	72.8 x 72.8 mm
Data Images	720 exposures
ω oscillation Range (χ=45.0, ϕ=0.0)	-70.0 - 110.0^{o}
Exposure Rate	48.0 sec./^{o}
Detector Swing Angle	19.92^{o}
ω oscillation Range (χ=45.0, ϕ=90.0)	-70.0 - 110.0^{o}
Exposure Rate	48.0 sec./^{o}
Detector Swing Angle	19.92^{o}
Detector Position	44.66 mm
Pixel Size	0.141 mm
$2\theta_{max}$	55.0^{o}
No. of Reflections Measured	Total: 29848
	Unique: 15856 (R_{int} = 0.0936)
Corrections	Lorentz-polarization

	Absorption
	(trans. factors: 0.789 - 0.960)
Structure Solution	Direct Methods (SHELXT Version 2014/5)
Refinement	Full-matrix least-squares on F^2
Function Minimized	Σ w $(Fo^2 - Fc^2)^2$
Least Squares Weights	$w = 1/ [\sigma^2(Fo^2) + (0.0794 \cdot P)^2$
	$+ 0.1973 \cdot P]$
	where $P = (Max(Fo^2,0) + 2Fc^2)/3$
$2\theta_{max}$ cutoff	55.0°
Anomalous Dispersion	All non-hydrogen atoms
No. Observations (All reflections)	15856
No. Variables	732
Reflection/Parameter Ratio	21.66
Residuals: R1 ($I>2.00\sigma(I)$)	0.0579
Residuals: R (All reflections)	0.0720
Residuals: wR2 (All reflections)	0.1625
Goodness of Fit Indicator	1.045
Max Shift/Error in Final Cycle	0.001
Maximum peak in Final Diff. Map	2.68 $e^-/Å^3$
Minimum peak in Final Diff. Map	-2.56 $e^-/Å^3$

Atomic coordinates and B_{iso}/B_{eq}

atom	x	y	z	B_{eq}
Pt1	0.27835(2)	0.31584(2)	0.22580(2)	2.669(6)
Si1	0.18452(11)	0.32327(9)	0.35408(9)	2.36(2)
Si2	0.28441(11)	0.25344(9)	0.45210(9)	2.42(2)
Si3	0.23153(12)	0.24971(10)	0.59036(9)	2.78(2)
Si4	0.34696(13)	0.15024(10)	0.66386(10)	3.13(3)
N1	0.1559(4)	0.1707(3)	0.2398(3)	2.91(8)
N2	0.0576(4)	0.2841(3)	0.2194(3)	3.03(8)
N3	0.4063(4)	0.3654(4)	0.0345(3)	3.78(10)
N4	0.4941(4)	0.2765(4)	0.1160(3)	3.69(9)
C1	0.1583(4)	0.2551(3)	0.2245(3)	2.81(9)
C2	0.3996(4)	0.3178(4)	0.1168(3)	2.89(9)
C3	0.0572(5)	0.1470(4)	0.2458(4)	4.09(12)
C4	-0.0064(5)	0.2184(4)	0.2326(4)	3.55(10)
C5	0.2545(5)	0.1187(4)	0.2415(4)	3.74(11)
C6	0.2358(5)	0.0340(4)	0.3254(4)	4.61(14)
C7	0.3012(7)	0.1034(6)	0.1638(5)	5.90(18)
C8	0.0265(6)	0.0578(5)	0.2669(5)	5.10(15)
C9	-0.1210(5)	0.2278(5)	0.2363(5)	4.93(14)
C10	0.0268(5)	0.3748(4)	0.1976(4)	3.60(11)
C11	-0.0778(5)	0.3851(6)	0.2596(5)	4.91(15)
C12	0.0235(6)	0.4216(5)	0.1049(5)	5.51(17)
C13	0.5081(6)	0.3534(7)	-0.0200(5)	5.58(19)
C14	0.5606(5)	0.2984(6)	0.0309(5)	5.22(16)
C15	0.3209(6)	0.4268(6)	0.0096(4)	5.03(16)
C16	0.3512(8)	0.5169(7)	-0.0076(9)	12.2(5)
C17	0.2880(8)	0.4261(14)	-0.0575(8)	15.9(8)
C18	0.5410(7)	0.3986(9)	-0.1156(5)	8.8(3)
C19	0.6724(6)	0.2654(8)	0.0061(6)	7.5(3)
C20	0.5209(5)	0.2219(4)	0.1953(4)	4.35(13)
C21	0.6009(5)	0.2687(5)	0.2036(4)	4.68(14)
C22	0.5552(6)	0.1307(5)	0.2056(7)	7.5(3)
C23	0.0488(4)	0.2648(3)	0.4199(3)	2.82(9)
C24	0.0361(5)	0.1834(4)	0.4292(4)	3.53(10)
C25	-0.0630(6)	0.1388(5)	0.4743(4)	4.60(14)
C26	-0.1513(5)	0.1759(5)	0.5103(4)	5.02(16)
C27	-0.1426(5)	0.2547(5)	0.5049(4)	4.96(16)
C28	-0.0438(5)	0.3005(4)	0.4602(4)	3.78(11)

C29	0.1609(4)	0.4374(3)	0.3437(3)	2.72(9)
C30	0.1336(5)	0.4566(4)	0.4122(4)	3.21(10)
C31	0.1217(5)	0.5406(4)	0.4033(4)	3.52(11)
C32	0.1334(5)	0.6089(4)	0.3235(4)	3.73(11)
C33	0.1575(5)	0.5919(4)	0.2539(4)	3.71(11)
C34	0.1727(4)	0.5086(3)	0.2638(3)	2.88(9)
C35	0.4220(4)	0.3076(4)	0.4036(3)	2.79(9)
C36	0.4358(4)	0.3952(4)	0.3438(4)	3.07(9)
C37	0.5336(5)	0.4401(4)	0.3125(4)	3.64(11)
C38	0.6170(5)	0.3972(5)	0.3409(4)	3.99(12)
C39	0.6057(5)	0.3103(5)	0.3997(4)	3.95(12)
C40	0.5104(4)	0.2661(4)	0.4305(4)	3.29(10)
C41	0.3044(4)	0.1354(3)	0.4751(3)	2.74(9)
C42	0.3973(5)	0.1063(4)	0.4305(4)	3.50(10)
C43	0.4104(6)	0.0191(4)	0.4492(5)	4.51(13)
C44	0.3327(6)	-0.0415(4)	0.5121(5)	4.77(14)
C45	0.2403(6)	-0.0152(4)	0.5561(5)	4.73(14)
C46	0.2249(5)	0.0720(4)	0.5388(4)	3.61(11)
C47	0.2447(4)	0.3608(4)	0.5801(3)	2.99(9)
C48	0.1543(5)	0.3992(4)	0.6167(4)	3.48(10)
C49	0.1611(6)	0.4815(5)	0.6086(4)	4.24(13)
C50	0.2559(6)	0.5281(5)	0.5620(5)	5.04(15)
C51	0.3461(6)	0.4921(5)	0.5246(5)	4.65(14)
C52	0.3411(5)	0.4102(4)	0.5321(4)	3.82(11)
C53	0.0912(5)	0.2079(4)	0.6676(4)	3.30(10)
C54	0.0611(5)	0.1989(5)	0.7515(4)	4.41(13)
C55	-0.0406(6)	0.1682(5)	0.8099(4)	5.36(17)
C56	-0.1154(6)	0.1448(5)	0.7868(4)	4.94(15)
C57	-0.0889(5)	0.1534(4)	0.7066(4)	4.11(12)
C58	0.0141(5)	0.1838(4)	0.6468(4)	3.63(11)
C59	0.4521(6)	0.1974(5)	0.6778(4)	4.42(13)
C60	0.5422(7)	0.1498(9)	0.6876(8)	8.9(3)
C61	0.6235(9)	0.1693(12)	0.7042(10)	11.4(4)
C62	0.6209(10)	0.2497(9)	0.6974(7)	8.4(3)
C63	0.5406(17)	0.3018(8)	0.6891(10)	13.6(6)
C64	0.4482(14)	0.2696(7)	0.6804(9)	12.4(5)
C65	0.2638(5)	0.0682(4)	0.7723(4)	4.19(12)
C66	0.2050(6)	0.0038(5)	0.7797(5)	5.16(16)
C67	0.1381(8)	-0.0531(6)	0.8588(6)	6.8(2)

C68	0.1258(8)	-0.0483(8)	0.9301(6)	8.9(3)
C69	0.1844(10)	0.0138(9)	0.9259(6)	10.2(4)
C70	0.2511(7)	0.0732(7)	0.8466(5)	6.9(2)

$B_{eq} = 8/3\ \pi^2(U_{11}(aa^*)^2 + U_{22}(bb^*)^2 + U_{33}(cc^*)^2 + 2U_{12}(aa^*bb^*)\cos\gamma + 2U_{13}(aa^*cc^*)\cos\beta + 2U_{23}(bb^*cc^*)\cos\alpha)$

Anisotropic displacement parameters

atom	U_{11}	U_{22}	U_{33}	U_{12}	U_{13}	U_{23}
Pt1	0.02859(12)	0.02834(12)	0.03489(14)	-0.00049(8)	-0.00343(9)	-0.01148(9)
Si1	0.0248(6)	0.0246(7)	0.0300(7)	0.0023(5)	-0.0037(5)	-0.0086(5)
Si2	0.0255(6)	0.0252(7)	0.0354(7)	0.0048(5)	-0.0084(6)	-0.0115(6)
Si3	0.0322(7)	0.0319(8)	0.0362(8)	0.0084(6)	-0.0104(6)	-0.0139(6)
Si4	0.0435(8)	0.0324(8)	0.0409(8)	0.0130(6)	-0.0196(7)	-0.0143(7)
N1	0.033(2)	0.028(2)	0.044(2)	0.0033(17)	-0.010(2)	-0.0137(19)
N2	0.031(2)	0.030(2)	0.050(3)	0.0045(17)	-0.011(2)	-0.018(2)
N3	0.032(2)	0.064(4)	0.045(3)	-0.009(2)	0.001(2)	-0.030(3)
N4	0.030(2)	0.053(3)	0.049(3)	0.001(2)	-0.000(2)	-0.026(2)
C1	0.036(3)	0.028(3)	0.038(3)	0.003(2)	-0.008(2)	-0.014(2)
C2	0.025(2)	0.036(3)	0.035(3)	-0.005(2)	0.007(2)	-0.016(2)
C3	0.049(3)	0.038(3)	0.069(4)	0.001(3)	-0.020(3)	-0.023(3)
C4	0.038(3)	0.038(3)	0.053(3)	-0.003(2)	-0.014(3)	-0.016(3)
C5	0.039(3)	0.039(3)	0.060(4)	0.010(2)	-0.013(3)	-0.024(3)
C6	0.045(4)	0.048(4)	0.070(4)	0.021(3)	-0.026(3)	-0.018(3)
C7	0.072(5)	0.074(6)	0.077(5)	0.026(4)	-0.014(4)	-0.047(4)
C8	0.061(4)	0.043(4)	0.090(5)	-0.004(3)	-0.029(4)	-0.026(4)
C9	0.045(4)	0.052(4)	0.093(5)	0.001(3)	-0.030(4)	-0.030(4)
C10	0.034(3)	0.034(3)	0.064(4)	0.005(2)	-0.015(3)	-0.020(3)
C11	0.046(4)	0.080(5)	0.076(5)	0.027(3)	-0.027(4)	-0.049(4)
C12	0.045(4)	0.050(4)	0.068(4)	0.005(3)	-0.011(3)	0.002(3)
C13	0.041(4)	0.132(8)	0.050(4)	-0.010(4)	-0.003(3)	-0.053(5)
C14	0.033(3)	0.100(6)	0.068(5)	-0.008(3)	0.004(3)	-0.052(5)
C15	0.042(4)	0.093(6)	0.043(3)	-0.005(4)	-0.011(3)	-0.021(4)
C16	0.062(6)	0.059(6)	0.215(14)	-0.010(5)	-0.043(7)	0.040(7)
C17	0.052(6)	0.49(3)	0.132(10)	0.061(11)	-0.043(6)	-0.203(16)
C18	0.043(4)	0.225(14)	0.053(5)	-0.003(6)	0.002(4)	-0.065(7)
C19	0.038(4)	0.168(11)	0.084(6)	0.021(5)	-0.003(4)	-0.079(7)
C20	0.036(3)	0.043(4)	0.062(4)	0.000(3)	-0.002(3)	-0.014(3)
C21	0.045(4)	0.064(5)	0.046(4)	0.001(3)	-0.007(3)	-0.012(3)
C22	0.050(4)	0.048(5)	0.138(8)	0.011(3)	0.003(5)	-0.030(5)

C23	0.030(3)	0.026(3)	0.035(3)	-0.0001(19)	-0.009(2)	-0.002(2)
C24	0.040(3)	0.036(3)	0.050(3)	-0.005(2)	-0.015(3)	-0.012(3)
C25	0.053(4)	0.048(4)	0.054(4)	-0.018(3)	-0.014(3)	-0.005(3)
C26	0.035(3)	0.051(4)	0.061(4)	-0.017(3)	-0.009(3)	0.010(3)
C27	0.024(3)	0.058(4)	0.058(4)	0.002(3)	0.004(3)	-0.002(3)
C28	0.037(3)	0.039(3)	0.040(3)	0.004(2)	-0.003(2)	-0.005(2)
C29	0.026(2)	0.033(3)	0.040(3)	0.0050(19)	-0.013(2)	-0.013(2)
C30	0.044(3)	0.030(3)	0.045(3)	0.010(2)	-0.020(3)	-0.014(2)
C31	0.044(3)	0.043(3)	0.058(4)	0.016(3)	-0.025(3)	-0.030(3)
C32	0.048(3)	0.029(3)	0.062(4)	0.009(2)	-0.016(3)	-0.022(3)
C33	0.044(3)	0.036(3)	0.040(3)	0.006(2)	-0.005(3)	-0.008(2)
C34	0.036(3)	0.028(3)	0.030(3)	0.004(2)	-0.003(2)	-0.007(2)
C35	0.030(3)	0.031(3)	0.041(3)	0.003(2)	-0.008(2)	-0.017(2)
C36	0.029(3)	0.041(3)	0.045(3)	-0.001(2)	-0.009(2)	-0.020(2)
C37	0.035(3)	0.050(4)	0.047(3)	-0.011(2)	-0.003(3)	-0.021(3)
C38	0.032(3)	0.069(5)	0.052(4)	-0.007(3)	-0.006(3)	-0.031(3)
C39	0.028(3)	0.063(4)	0.061(4)	0.003(3)	-0.014(3)	-0.030(3)
C40	0.036(3)	0.040(3)	0.047(3)	0.009(2)	-0.015(2)	-0.019(3)
C41	0.031(3)	0.031(3)	0.037(3)	0.007(2)	-0.012(2)	-0.013(2)
C42	0.040(3)	0.040(3)	0.054(3)	0.012(2)	-0.018(3)	-0.023(3)
C43	0.052(4)	0.041(4)	0.086(5)	0.025(3)	-0.030(4)	-0.037(4)
C44	0.070(4)	0.028(3)	0.092(5)	0.018(3)	-0.046(4)	-0.025(3)
C45	0.063(4)	0.031(3)	0.081(5)	0.003(3)	-0.037(4)	-0.012(3)
C46	0.041(3)	0.032(3)	0.057(4)	0.007(2)	-0.014(3)	-0.018(3)
C47	0.039(3)	0.036(3)	0.038(3)	0.010(2)	-0.016(2)	-0.017(2)
C48	0.046(3)	0.043(3)	0.050(3)	0.020(3)	-0.026(3)	-0.024(3)
C49	0.054(4)	0.059(4)	0.065(4)	0.031(3)	-0.032(3)	-0.041(4)
C50	0.076(5)	0.056(4)	0.093(5)	0.029(4)	-0.052(5)	-0.050(4)
C51	0.058(4)	0.055(4)	0.076(5)	-0.004(3)	-0.030(4)	-0.032(4)
C52	0.041(3)	0.052(4)	0.058(4)	0.003(3)	-0.015(3)	-0.031(3)
C53	0.037(3)	0.036(3)	0.040(3)	0.009(2)	-0.009(2)	-0.013(2)
C54	0.051(4)	0.063(5)	0.037(3)	0.013(3)	-0.012(3)	-0.015(3)
C55	0.060(4)	0.075(5)	0.034(3)	0.017(4)	-0.005(3)	-0.008(3)
C56	0.048(4)	0.045(4)	0.050(4)	-0.001(3)	0.009(3)	-0.004(3)
C57	0.041(3)	0.032(3)	0.063(4)	0.000(2)	-0.008(3)	-0.013(3)
C58	0.038(3)	0.030(3)	0.054(3)	0.008(2)	-0.011(3)	-0.011(3)
C59	0.059(4)	0.059(4)	0.038(3)	-0.009(3)	-0.018(3)	-0.008(3)
C60	0.044(5)	0.176(12)	0.181(11)	0.036(6)	-0.051(6)	-0.130(10)
C61	0.058(6)	0.182(16)	0.217(15)	0.008(8)	-0.052(8)	-0.105(13)

C62	0.106(9)	0.094(9)	0.085(7)	-0.057(7)	-0.002(6)	-0.017(6)
C63	0.30(2)	0.049(7)	0.205(15)	-0.018(10)	-0.182(16)	-0.008(8)
C64	0.289(18)	0.058(6)	0.227(14)	0.042(8)	-0.232(14)	-0.049(8)
C65	0.052(4)	0.048(4)	0.046(3)	0.006(3)	-0.023(3)	-0.007(3)
C66	0.064(4)	0.044(4)	0.069(4)	-0.008(3)	-0.029(4)	-0.003(3)
C67	0.087(6)	0.066(6)	0.066(5)	-0.019(5)	-0.028(5)	0.010(4)
C68	0.075(6)	0.130(10)	0.055(5)	-0.037(6)	-0.020(5)	0.029(5)
C69	0.124(9)	0.184(14)	0.047(5)	-0.062(9)	-0.032(5)	-0.007(6)
C70	0.090(6)	0.110(8)	0.046(4)	-0.031(5)	-0.026(4)	-0.012(4)

The general temperature factor expression: $\exp(-2\pi^2(a^{*2}U_{11}h^2 + b^{*2}U_{22}k^2 + c^{*2}U_{33}l^2 + 2a^*b^*U_{12}hk + 2a^*c^*U_{13}hl + 2b^*c^*U_{23}kl))$

Bond lengths (Å)

atom	atom	distance	atom	atom	distance
Pt1	Si1	2.3355(17)	Pt1	C1	2.060(7)
Pt1	C2	2.090(5)	Si1	Si2	2.402(2)
Si1	C23	1.897(5)	Si1	C29	1.903(6)
Si2	Si3	2.401(3)	Si2	C35	1.894(6)
Si2	C41	1.900(6)	Si3	Si4	2.393(2)
Si3	C47	1.892(7)	Si3	C53	1.896(5)
Si4	C59	1.874(10)	Si4	C65	1.882(5)
N1	C1	1.369(8)	N1	C3	1.386(10)
N1	C5	1.479(7)	N2	C1	1.376(7)
N2	C4	1.410(10)	N2	C10	1.474(8)
N3	C2	1.339(7)	N3	C13	1.431(9)
N3	C15	1.482(9)	N4	C2	1.350(7)
N4	C14	1.410(9)	N4	C20	1.480(9)
C3	C4	1.371(9)	C3	C8	1.503(12)
C4	C9	1.486(10)	C5	C6	1.540(8)
C5	C7	1.501(13)	C10	C11	1.511(9)
C10	C12	1.547(11)	C13	C14	1.315(11)
C13	C18	1.496(10)	C14	C19	1.503(11)
C15	C16	1.541(17)	C15	C17	1.48(2)
C20	C21	1.525(13)	C20	C22	1.533(12)
C23	C24	1.382(10)	C23	C28	1.422(8)
C24	C25	1.397(9)	C25	C26	1.368(10)
C26	C27	1.356(13)	C27	C28	1.403(9)
C29	C30	1.390(10)	C29	C34	1.398(6)
C30	C31	1.390(10)	C31	C32	1.377(8)

C32	C33	1.388(11)	C33	C34	1.375(9)
C35	C36	1.402(7)	C35	C40	1.408(8)
C36	C37	1.406(9)	C37	C38	1.367(9)
C38	C39	1.388(9)	C39	C40	1.374(9)
C41	C42	1.399(8)	C41	C46	1.406(7)
C42	C43	1.395(10)	C43	C44	1.366(8)
C44	C45	1.365(10)	C45	C46	1.403(10)
C47	C48	1.403(9)	C47	C52	1.407(8)
C48	C49	1.390(12)	C49	C50	1.366(10)
C50	C51	1.383(11)	C51	C52	1.383(12)
C53	C54	1.412(10)	C53	C58	1.396(11)
C54	C55	1.386(9)	C55	C56	1.393(14)
C56	C57	1.352(12)	C57	C58	1.406(7)
C59	C60	1.369(12)	C59	C64	1.282(17)
C60	C61	1.35(2)	C61	C62	1.35(3)
C62	C63	1.30(2)	C63	C64	1.51(3)
C65	C66	1.394(13)	C65	C70	1.386(14)
C66	C67	1.383(9)	C67	C68	1.330(18)
C68	C69	1.39(2)	C69	C70	1.399(11)
Pt1	H1	1.55(6)	Si4	H2	1.38(7)

Torsion Angles(⁰)

(Those having bond angles > 160 or < 20 degrees are excluded.)

atom1	atom2	atom3	atom4	angle	atom1	atom2	atom3	atom4	angle
Si1	Pt1	C1	N1	-107.2(4)	Si1	Pt1	C1	N2	63.5(4)
C1	Pt1	Si1	Si2	123.25(15)	C1	Pt1	Si1	C23	7.33(15)
C1	Pt1	Si1	C29	-118.03(16)	C1	Pt1	C2	N3	71.1(6)
C1	Pt1	C2	N4	-116.6(5)	C2	Pt1	C1	N1	68.7(4)
C2	Pt1	C1	N2	-120.6(4)	Pt1	Si1	Si2	Si3	175.89(6)
Pt1	Si1	Si2	C35	59.15(11)	Pt1	Si1	Si2	C41	-60.01(8)
Pt1	Si1	C23	C24	41.7(5)	Pt1	Si1	C23	C28	-137.1(4)
Pt1	Si1	C29	C30	-162.2(3)	Pt1	Si1	C29	C34	16.5(5)
Si2	Si1	C23	C24	-78.5(4)	Si2	Si1	C23	C28	102.7(4)
C23	Si1	Si2	Si3	-58.6(2)	C23	Si1	Si2	C35	-175.3(2)
C23	Si1	Si2	C41	65.5(2)	Si2	Si1	C29	C30	-41.0(4)
Si2	Si1	C29	C34	137.6(3)	C29	Si1	Si2	Si3	51.55(16)
C29	Si1	Si2	C35	-65.19(16)	C29	Si1	Si2	C41	175.65(14)
C23	Si1	C29	C30	66.4(5)	C23	Si1	C29	C34	-114.9(4)
C29	Si1	C23	C24	171.4(4)	C29	Si1	C23	C28	-7.4(5)

Si1	Si2	Si3	Si4	170.17(8)	Si1	Si2	Si3	C47	-69.17(12)
Si1	Si2	Si3	C53	53.85(13)	Si1	Si2	C35	C36	28.0(5)
Si1	Si2	C35	C40	-158.0(4)	Si1	Si2	C41	C42	95.1(5)
Si1	Si2	C41	C46	-84.9(5)	Si3	Si2	C35	C36	-103.6(5)
Si3	Si2	C35	C40	70.4(5)	C35	Si2	Si3	Si4	-67.97(18)
C35	Si2	Si3	C47	52.69(18)	C35	Si2	Si3	C53	175.71(17)
Si3	Si2	C41	C42	-129.9(4)	Si3	Si2	C41	C46	50.2(5)
C41	Si2	Si3	Si4	43.6(2)	C41	Si2	Si3	C47	164.26(19)
C41	Si2	Si3	C53	-72.7(2)	C35	Si2	C41	C42	-25.2(6)
C35	Si2	C41	C46	154.9(4)	C41	Si2	C35	C36	148.1(5)
C41	Si2	C35	C40	-37.8(6)	Si2	Si3	Si4	C59	107.77(11)
Si2	Si3	Si4	C65	-127.95(10)	Si2	Si3	C47	C48	121.7(4)
Si2	Si3	C47	C52	-54.3(5)	Si2	Si3	C53	C54	175.2(3)
Si2	Si3	C53	C58	-3.9(5)	Si4	Si3	C47	C48	-119.7(4)
Si4	Si3	C47	C52	64.3(5)	C47	Si3	Si4	C59	-13.2(2)
C47	Si3	Si4	C65	111.03(19)	Si4	Si3	C53	C54	57.0(4)
Si4	Si3	C53	C58	-122.2(4)	C53	Si3	Si4	C59	-126.2(3)
C53	Si3	Si4	C65	-2.0(3)	C47	Si3	C53	C54	-59.2(4)
C47	Si3	C53	C58	121.6(4)	C53	Si3	C47	C48	-8.3(5)
C53	Si3	C47	C52	175.7(4)	Si3	Si4	C59	C60	-155.0(3)
Si3	Si4	C59	C64	26.1(5)	Si3	Si4	C65	C66	72.2(5)
Si3	Si4	C65	C70	-102.4(5)	C59	Si4	C65	C66	-158.7(5)
C59	Si4	C65	C70	26.7(6)	C65	Si4	C59	C60	80.0(5)
C65	Si4	C59	C64	-99.0(5)	C1	N1	C3	C4	0.9(7)
C1	N1	C3	C8	-176.5(5)	C3	N1	C1	Pt1	171.6(5)
C3	N1	C1	N2	-1.1(6)	C1	N1	C5	C6	125.5(6)
C1	N1	C5	C7	-107.6(6)	C5	N1	C1	Pt1	-13.0(8)
C5	N1	C1	N2	174.3(5)	C3	N1	C5	C6	-59.9(9)
C3	N1	C5	C7	67.0(7)	C5	N1	C3	C4	-174.1(5)
C5	N1	C3	C8	8.5(10)	C1	N2	C4	C3	-0.3(6)
C1	N2	C4	C9	176.2(5)	C4	N2	C1	Pt1	-171.6(4)
C4	N2	C1	N1	0.8(6)	C1	N2	C10	C11	-128.8(6)
C1	N2	C10	C12	105.9(6)	C10	N2	C1	Pt1	11.8(8)
C10	N2	C1	N1	-175.8(5)	C4	N2	C10	C11	55.1(9)
C4	N2	C10	C12	-70.2(6)	C10	N2	C4	C3	176.2(5)
C10	N2	C4	C9	-7.4(9)	C2	N3	C13	C14	0.1(11)
C2	N3	C13	C18	-179.3(8)	C13	N3	C2	Pt1	173.8(7)
C13	N3	C2	N4	0.4(9)	C2	N3	C15	C16	92.5(7)
C2	N3	C15	C17	-136.4(6)	C15	N3	C2	Pt1	-0.5(11)

C15	N3	C2	N4	-173.9(7)	C13	N3	C15	C16	-80.8(11)
C13	N3	C15	C17	50.4(11)	C15	N3	C13	C14	174.0(8)
C15	N3	C13	C18	-5.4(16)	C2	N4	C14	C13	0.8(10)
C2	N4	C14	C19	179.0(8)	C14	N4	C2	Pt1	-174.5(6)
C14	N4	C2	N3	-0.7(9)	C2	N4	C20	C21	-104.0(7)
C2	N4	C20	C22	127.0(6)	C20	N4	C2	Pt1	1.8(10)
C20	N4	C2	N3	175.6(6)	C14	N4	C20	C21	71.6(10)
C14	N4	C20	C22	-57.4(10)	C20	N4	C14	C13	-175.2(7)
C20	N4	C14	C19	2.9(15)	N1	C3	C4	N2	-0.4(7)
N1	C3	C4	C9	-176.6(6)	C8	C3	C4	N2	177.0(7)
C8	C3	C4	C9	0.8(13)	N3	C13	C14	N4	-0.6(12)
N3	C13	C14	C19	-178.6(9)	C18	C13	C14	N4	178.8(12)
C18	C13	C14	C19	1(2)	Si1	C23	C24	C25	-177.6(4)
Si1	C23	C28	C27	177.3(4)	C24	C23	C28	C27	-1.6(9)
C28	C23	C24	C25	1.2(9)	C23	C24	C25	C26	0.6(11)
C24	C25	C26	C27	-2.2(12)	C25	C26	C27	C28	1.9(11)
C26	C27	C28	C23	0.0(11)	Si1	C29	C30	C31	176.7(4)
Si1	C29	C34	C33	-179.2(4)	C30	C29	C34	C33	-0.4(8)
C34	C29	C30	C31	-2.0(8)	C29	C30	C31	C32	2.4(9)
C30	C31	C32	C33	-0.4(9)	C31	C32	C33	C34	-1.9(9)
C32	C33	C34	C29	2.4(9)	Si2	C35	C36	C37	173.7(4)
Si2	C35	C40	C39	-173.5(5)	C36	C35	C40	C39	0.6(10)
C40	C35	C36	C37	-0.6(10)	C35	C36	C37	C38	0.2(11)
C36	C37	C38	C39	0.1(12)	C37	C38	C39	C40	-0.1(13)
C38	C39	C40	C35	-0.3(12)	Si2	C41	C42	C43	179.4(5)
Si2	C41	C46	C45	-179.5(5)	C42	C41	C46	C45	0.6(11)
C46	C41	C42	C43	-0.7(11)	C41	C42	C43	C44	-0.1(13)
C42	C43	C44	C45	0.9(14)	C43	C44	C45	C46	-1.0(15)
C44	C45	C46	C41	0.3(14)	Si3	C47	C48	C49	-178.6(4)
Si3	C47	C52	C51	178.4(4)	C48	C47	C52	C51	2.2(10)
C52	C47	C48	C49	-2.4(10)	C47	C48	C49	C50	2.2(11)
C48	C49	C50	C51	-1.7(13)	C49	C50	C51	C52	1.6(13)
C50	C51	C52	C47	-1.9(12)	Si3	C53	C54	C55	-179.7(4)
Si3	C53	C58	C57	-179.9(3)	C54	C53	C58	C57	0.9(8)
C58	C53	C54	C55	-0.5(9)	C53	C54	C55	C56	0.5(11)
C54	C55	C56	C57	-1.0(11)	C55	C56	C57	C58	1.5(10)
C56	C57	C58	C53	-1.5(9)	Si4	C59	C60	C61	-175.2(7)
Si4	C59	C64	C63	-177.9(6)	C60	C59	C64	C63	3.1(15)
C64	C59	C60	C61	3.9(14)	C59	C60	C61	C62	-10.4(18)

C60	C61	C62	C63	10.5(19)	C61	C62	C63	C64	-5(2)
C62	C63	C64	C59	-3(2)	Si4	C65	C66	C67	-175.7(4)
Si4	C65	C70	C69	176.8(5)	C66	C65	C70	C69	2.0(11)
C70	C65	C66	C67	-0.9(10)	C65	C66	C67	C68	1.0(12)
C66	C67	C68	C69	-2.2(14)	C67	C68	C69	C70	3.3(17)
C68	C69	C70	C65	-3.3(16)					

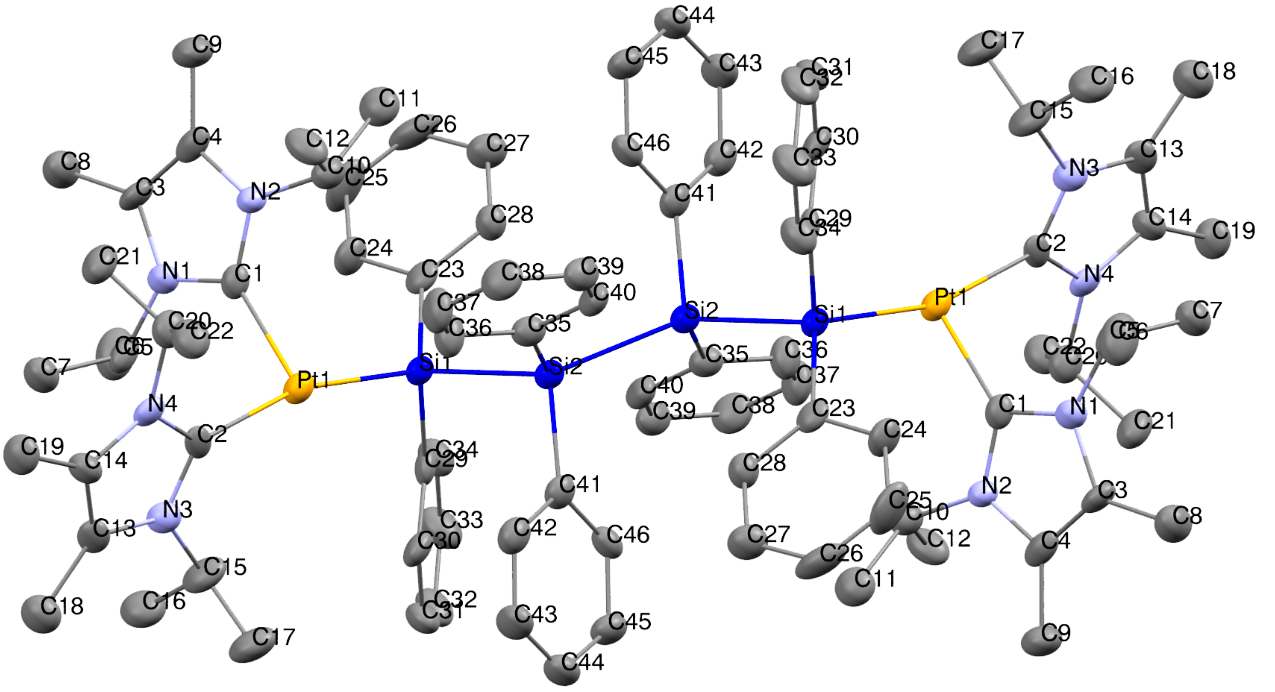

Supplementary Fig. 46. ORTEP drawing of **4** (50% probability of the thermal ellipsoids). Hydrogen atoms were omitted for clarity.

Supplementary Table 11. Crystal data and structure refinement for **4**.

Empirical Formula	$C_{92}H_{122}N_8Pt_2Si_4$
Formula Weight	1842.55
Crystal Color, Habit	yellow, platelet
Crystal Dimensions	0.100 X 0.050 X 0.020 mm
Crystal System	triclinic
Lattice Type	Primitive
Lattice Parameters	a = 12.5743(17) Å
	b = 12.631(3) Å
	c = 17.132(3) Å
	α = 89.617(13) ^{o}
	β = 69.219(10) ^{o}
	γ = 60.968(7) ^{o}
	V = 2178.3(8) $Å^3$
Space Group	P-1 (#2)
Z value	1
D_{calc}	1.404 g/cm^3
F_{000}	942.00
μ(MoKα)	32.984 cm^{-1}
Diffractometer	Saturn724
Radiation	MoKα (λ = 0.71075 Å)
	multi-layer mirror monochromated
Voltage, Current	50kV, 24mA
Temperature	-149.8^{o}C
Detector Aperture	72.8 x 72.8 mm
Data Images	720 exposures
ω oscillation Range (χ=45.0, ϕ=0.0)	-70.0 - 110.0^{o}
Exposure Rate	96.0 sec./^{o}
Detector Swing Angle	19.88^{o}
ω oscillation Range (χ=45.0, ϕ=90.0)	-70.0 - 110.0^{o}
Exposure Rate	96.0 sec./^{o}
Detector Swing Angle	19.88^{o}
Detector Position	44.66 mm
Pixel Size	0.141 mm
$2\theta_{max}$	54.9^{o}
No. of Reflections Measured	Total: 18232
	Unique: 9593 (R_{int} = 0.0929)
Corrections	Lorentz-polarization

	Absorption<br>(trans. factors: 0.748 - 0.936)
Structure Solution	Direct Methods (SHELXT Version 2014/5)
Refinement	Full-matrix least-squares on F^2
Function Minimized	Σ w $(Fo^2 - Fc^2)^2$
Least Squares Weights	w = 1/ [$\sigma^2(Fo^2)$ + $(0.0483 \cdot P)^2$<br>+ 0.0000 · P]<br>where P = $(Max(Fo^2,0) + 2Fc^2)/3$
$2\theta_{max}$ cutoff	54.9°
Anomalous Dispersion	All non-hydrogen atoms
No. Observations (All reflections)	9593
No. Variables	490
Reflection/Parameter Ratio	19.58
Residuals: R1 (I>2.00σ(I))	0.0569
Residuals: R (All reflections)	0.0807
Residuals: wR2 (All reflections)	0.1290
Goodness of Fit Indicator	1.018
Max Shift/Error in Final Cycle	0.001
Maximum peak in Final Diff. Map	3.24 $e^-/Å^3$
Minimum peak in Final Diff. Map	-1.97 $e^-/Å^3$

Atomic coordinates and B_{iso}/B_{eq}

atom	x	y	z	B_{eq}
Pt1	0.58438(3)	0.26082(2)	0.73458(2)	1.973(7)
Si1	0.45219(18)	0.43674(15)	0.69275(11)	1.69(3)
Si2	0.55885(18)	0.43683(15)	0.54187(11)	1.77(3)
N1	0.3888(6)	0.2054(5)	0.8688(4)	2.08(10)
N2	0.4049(6)	0.1544(5)	0.7434(3)	1.89(10)
N3	0.7618(6)	0.1733(5)	0.8331(4)	2.17(10)
N4	0.7856(5)	0.0128(5)	0.7688(3)	1.92(10)
C1	0.4491(7)	0.2050(5)	0.7848(4)	1.88(11)
C2	0.7217(6)	0.1390(6)	0.7800(4)	1.70(11)
C3	0.3064(7)	0.1563(6)	0.8791(5)	2.26(13)
C4	0.3157(7)	0.1248(6)	0.7993(5)	2.37(13)
C5	0.4086(8)	0.2545(6)	0.9363(4)	2.35(13)
C6	0.2792(9)	0.3599(7)	1.0050(5)	3.94(19)
C7	0.4890(8)	0.1529(7)	0.9745(5)	2.94(14)
C8	0.2230(9)	0.1464(8)	0.9624(5)	3.52(16)
C9	0.2513(9)	0.0678(7)	0.7743(5)	3.47(16)
C10	0.4426(7)	0.1416(6)	0.6494(4)	2.21(12)
C11	0.3219(8)	0.1996(6)	0.6267(5)	3.22(16)
C12	0.5417(8)	0.0080(6)	0.6021(5)	3.02(15)
C13	0.8524(6)	0.0697(6)	0.8560(4)	2.07(12)
C14	0.8664(7)	-0.0314(6)	0.8145(4)	2.33(12)
C15	0.7226(8)	0.3049(6)	0.8554(5)	3.16(16)
C16	0.6697(9)	0.3512(7)	0.9497(5)	3.58(17)
C17	0.8379(9)	0.3235(7)	0.8041(6)	3.85(18)
C18	0.9147(8)	0.0751(7)	0.9144(5)	3.22(15)
C19	0.9502(7)	-0.1642(6)	0.8156(5)	2.78(14)
C20	0.7680(7)	-0.0592(6)	0.7117(4)	2.44(13)
C21	0.6988(9)	-0.1250(7)	0.7601(5)	3.31(16)
C22	0.9001(8)	-0.1469(6)	0.6366(5)	2.92(15)
C23	0.2734(7)	0.4792(5)	0.7206(4)	2.11(12)
C24	0.1987(7)	0.4696(6)	0.7999(4)	2.50(14)
C25	0.0721(8)	0.4898(7)	0.8220(6)	4.0(2)
C26	0.0156(8)	0.5206(6)	0.7630(6)	3.69(19)
C27	0.0840(8)	0.5339(6)	0.6844(5)	2.88(14)
C28	0.2103(7)	0.5142(6)	0.6636(4)	2.28(12)
C29	0.4321(7)	0.5755(5)	0.7565(4)	1.99(12)
C30	0.5409(8)	0.5761(6)	0.7594(4)	2.51(14)

C31	0.5282(9)	0.6754(7)	0.8042(5)	2.96(14)
C32	0.4033(9)	0.7788(7)	0.8489(5)	3.24(16)
C33	0.2953(9)	0.7796(7)	0.8476(5)	3.48(16)
C34	0.3071(8)	0.6801(6)	0.8029(4)	2.67(14)
C35	0.6715(7)	0.2717(6)	0.4775(4)	2.13(12)
C36	0.7339(8)	0.1691(6)	0.5122(4)	2.73(14)
C37	0.8243(8)	0.0517(6)	0.4623(5)	3.06(15)
C38	0.8570(8)	0.0300(6)	0.3775(5)	3.12(16)
C39	0.7984(8)	0.1276(7)	0.3401(4)	2.68(14)
C40	0.7073(7)	0.2470(6)	0.3902(5)	2.48(13)
C41	0.6741(7)	0.4876(6)	0.5495(4)	2.10(12)
C42	0.7966(8)	0.4033(7)	0.5526(4)	2.61(14)
C43	0.8746(8)	0.4418(7)	0.5670(5)	3.06(15)
C44	0.8366(8)	0.5653(7)	0.5775(5)	2.87(14)
C45	0.7170(7)	0.6513(7)	0.5737(5)	2.63(14)
C46	0.6386(7)	0.6123(6)	0.5601(4)	2.14(12)

B_{eq} = 8/3 $\pi^2(U_{11}(aa^*)^2 + U_{22}(bb^*)^2 + U_{33}(cc^*)^2 + 2U_{12}(aa^*bb^*)\cos\gamma + 2U_{13}(aa^*cc^*)\cos\beta + 2U_{23}(bb^*cc^*)\cos\alpha)$

Anisotropic displacement parameters

atom	U_{11}	U_{22}	U_{33}	U_{12}	U_{13}	U_{23}
Pt1	0.02202(15)	0.01917(13)	0.02877(15)	-0.00974(10)	-0.00637(12)	0.00479(9)
Si1	0.0183(9)	0.0206(8)	0.0225(9)	-0.0100(7)	-0.0056(8)	0.0055(7)
Si2	0.0177(9)	0.0196(8)	0.0273(9)	-0.0083(7)	-0.0080(8)	0.0054(7)
N1	0.021(3)	0.023(3)	0.034(3)	-0.010(2)	-0.013(3)	0.007(2)
N2	0.026(3)	0.023(3)	0.027(3)	-0.015(2)	-0.011(3)	0.005(2)
N3	0.020(3)	0.020(3)	0.036(3)	-0.007(2)	-0.009(3)	0.002(2)
N4	0.017(3)	0.021(3)	0.028(3)	-0.011(2)	-0.002(2)	0.004(2)
C1	0.017(3)	0.017(3)	0.023(3)	-0.003(2)	-0.003(3)	0.003(2)
C2	0.009(3)	0.026(3)	0.019(3)	-0.005(3)	-0.001(3)	0.006(2)
C3	0.021(4)	0.025(3)	0.039(4)	-0.016(3)	-0.006(3)	0.009(3)
C4	0.014(3)	0.018(3)	0.048(4)	-0.005(3)	-0.007(3)	0.012(3)
C5	0.038(4)	0.026(3)	0.022(3)	-0.020(3)	-0.004(3)	0.005(3)
C6	0.049(6)	0.033(4)	0.040(4)	-0.010(4)	-0.005(4)	0.003(3)
C7	0.034(4)	0.043(4)	0.040(4)	-0.020(4)	-0.020(4)	0.013(3)
C8	0.042(5)	0.059(5)	0.041(4)	-0.032(4)	-0.018(4)	0.017(4)
C9	0.047(5)	0.045(5)	0.058(5)	-0.033(4)	-0.028(4)	0.016(4)
C10	0.025(4)	0.027(3)	0.029(4)	-0.014(3)	-0.007(3)	0.003(3)
C11	0.039(5)	0.027(4)	0.051(5)	-0.013(3)	-0.018(4)	0.006(3)

C12	0.037(5)	0.025(3)	0.042(4)	-0.005(3)	-0.021(4)	-0.007(3)
C13	0.009(3)	0.027(3)	0.032(4)	-0.004(3)	-0.005(3)	0.006(3)
C14	0.027(4)	0.030(4)	0.035(4)	-0.013(3)	-0.018(3)	0.007(3)
C15	0.033(4)	0.018(3)	0.064(5)	-0.013(3)	-0.014(4)	0.006(3)
C16	0.047(5)	0.032(4)	0.058(5)	-0.020(4)	-0.021(4)	0.002(4)
C17	0.045(5)	0.035(4)	0.072(6)	-0.028(4)	-0.018(5)	0.009(4)
C18	0.033(4)	0.037(4)	0.049(5)	-0.013(3)	-0.020(4)	0.008(4)
C19	0.024(4)	0.026(4)	0.041(4)	-0.004(3)	-0.011(3)	0.006(3)
C20	0.027(4)	0.028(4)	0.027(4)	-0.010(3)	-0.004(3)	0.002(3)
C21	0.055(6)	0.035(4)	0.043(4)	-0.029(4)	-0.018(4)	0.013(3)
C22	0.038(5)	0.025(3)	0.037(4)	-0.009(3)	-0.014(4)	-0.001(3)
C23	0.026(4)	0.012(3)	0.036(4)	-0.008(3)	-0.008(3)	0.004(3)
C24	0.022(4)	0.031(4)	0.030(4)	-0.012(3)	-0.001(3)	0.008(3)
C25	0.026(4)	0.040(4)	0.055(5)	-0.012(4)	0.007(4)	0.018(4)
C26	0.020(4)	0.025(4)	0.087(7)	-0.013(3)	-0.011(4)	0.009(4)
C27	0.024(4)	0.033(4)	0.045(4)	-0.012(3)	-0.011(4)	0.004(3)
C28	0.026(4)	0.026(3)	0.029(4)	-0.010(3)	-0.010(3)	0.007(3)
C29	0.030(4)	0.018(3)	0.021(3)	-0.009(3)	-0.009(3)	0.010(2)
C30	0.038(4)	0.026(3)	0.033(4)	-0.021(3)	-0.009(3)	0.012(3)
C31	0.050(5)	0.037(4)	0.043(4)	-0.026(4)	-0.033(4)	0.014(3)
C32	0.060(6)	0.033(4)	0.037(4)	-0.020(4)	-0.030(4)	0.002(3)
C33	0.047(5)	0.034(4)	0.041(4)	-0.009(4)	-0.024(4)	-0.004(3)
C34	0.032(4)	0.026(3)	0.035(4)	-0.006(3)	-0.017(3)	0.001(3)
C35	0.021(4)	0.025(3)	0.029(4)	-0.011(3)	-0.006(3)	0.001(3)
C36	0.037(4)	0.026(4)	0.027(4)	-0.009(3)	-0.009(3)	0.006(3)
C37	0.040(5)	0.025(4)	0.039(4)	-0.009(3)	-0.015(4)	0.013(3)
C38	0.034(5)	0.020(3)	0.049(5)	-0.007(3)	-0.010(4)	0.000(3)
C39	0.036(4)	0.044(4)	0.023(3)	-0.026(4)	-0.006(3)	0.004(3)
C40	0.033(4)	0.025(3)	0.045(4)	-0.018(3)	-0.021(4)	0.018(3)
C41	0.023(4)	0.028(3)	0.025(3)	-0.013(3)	-0.004(3)	0.006(3)
C42	0.033(4)	0.031(4)	0.030(4)	-0.017(3)	-0.007(3)	0.007(3)
C43	0.030(4)	0.037(4)	0.055(5)	-0.016(3)	-0.026(4)	0.017(4)
C44	0.032(4)	0.047(4)	0.042(4)	-0.024(4)	-0.021(4)	0.014(3)
C45	0.028(4)	0.036(4)	0.038(4)	-0.021(3)	-0.010(3)	0.008(3)
C46	0.022(4)	0.030(3)	0.026(3)	-0.009(3)	-0.013(3)	0.005(3)

The general temperature factor expression: $\exp(-2\pi^2(a^{*2}U_{11}h^2 + b^{*2}U_{22}k^2 + c^{*2}U_{33}l^2 + 2a^*b^*U_{12}hk + 2a^*c^*U_{13}hl + 2b^*c^*U_{23}kl))$

Bond lengths (Å)

atom	atom	distance	atom	atom	distance
Pt1	Si1	2.3352(18)	Pt1	C1	2.061(9)
Pt1	C2	2.077(7)	Si1	Si2	2.454(3)
Si1	C23	1.908(9)	Si1	C29	1.931(8)
Si2	Si2[1]	2.445(3)	Si2	C35	1.904(6)
Si2	C41	1.888(10)	N1	C1	1.367(8)
N1	C3	1.408(13)	N1	C5	1.464(12)
N2	C1	1.361(12)	N2	C4	1.398(10)
N2	C10	1.493(9)	N3	C2	1.347(12)
N3	C13	1.424(9)	N3	C15	1.494(10)
N4	C2	1.369(8)	N4	C14	1.403(11)
N4	C20	1.480(12)	C3	C4	1.376(12)
C3	C8	1.488(11)	C4	C9	1.474(16)
C5	C6	1.539(8)	C5	C7	1.515(11)
C10	C11	1.526(13)	C10	C12	1.522(8)
C13	C14	1.368(12)	C13	C18	1.493(14)
C14	C19	1.490(9)	C15	C16	1.505(12)
C15	C17	1.533(14)	C20	C21	1.522(14)
C20	C22	1.536(8)	C23	C24	1.396(10)
C23	C28	1.408(12)	C24	C25	1.386(14)
C25	C26	1.383(16)	C26	C27	1.376(12)
C27	C28	1.388(13)	C29	C30	1.390(15)
C29	C34	1.400(8)	C30	C31	1.384(14)
C31	C32	1.388(9)	C32	C33	1.362(18)
C33	C34	1.393(14)	C35	C36	1.409(10)
C35	C40	1.389(10)	C36	C37	1.384(8)
C37	C38	1.351(12)	C38	C39	1.383(11)
C39	C40	1.402(8)	C41	C42	1.405(11)
C41	C46	1.407(11)	C42	C43	1.378(16)
C43	C44	1.383(12)	C44	C45	1.389(11)
C45	C46	1.380(15)			

Symmetry Operators:

(1) -X+1,-Y+1,-Z+1

Bond angles (°)

atom	atom	atom	angle	atom	atom	atom	angle
Si1	Pt1	C1	98.11(19)	Si1	Pt1	C2	162.4(2)
C1	Pt1	C2	96.1(3)	Pt1	Si1	Si2	112.75(7)

Pt1	Si1	C23	117.0(2)	Pt1	Si1	C29	106.4(2)
Si2	Si1	C23	110.2(3)	Si2	Si1	C29	105.9(2)
C23	Si1	C29	103.4(3)	Si1	Si2	Si2[1]	124.63(9)
Si1	Si2	C35	110.1(2)	Si1	Si2	C41	99.3(2)
Si2[1]	Si2	C35	107.2(3)	Si2[1]	Si2	C41	108.4(2)
C35	Si2	C41	105.5(3)	C1	N1	C3	110.7(7)
C1	N1	C5	122.4(7)	C3	N1	C5	126.9(6)
C1	N2	C4	112.3(6)	C1	N2	C10	122.3(7)
C4	N2	C10	125.3(8)	C2	N3	C13	111.9(6)
C2	N3	C15	121.3(6)	C13	N3	C15	126.6(8)
C2	N4	C14	111.3(7)	C2	N4	C20	120.4(6)
C14	N4	C20	128.3(5)	Pt1	C1	N1	126.4(7)
Pt1	C1	N2	128.9(4)	N1	C1	N2	104.6(7)
Pt1	C2	N3	124.6(4)	Pt1	C2	N4	130.7(6)
N3	C2	N4	104.7(6)	N1	C3	C4	107.0(7)
N1	C3	C8	124.9(8)	C4	C3	C8	128.1(9)
N2	C4	C3	105.4(8)	N2	C4	C9	125.5(7)
C3	C4	C9	129.0(7)	N1	C5	C6	113.7(8)
N1	C5	C7	111.9(6)	C6	C5	C7	112.1(6)
N2	C10	C11	112.4(5)	N2	C10	C12	112.1(6)
C11	C10	C12	111.8(8)	N3	C13	C14	105.4(7)
N3	C13	C18	125.7(7)	C14	C13	C18	128.9(6)
N4	C14	C13	106.8(6)	N4	C14	C19	125.0(8)
C13	C14	C19	128.2(8)	N3	C15	C16	113.2(7)
N3	C15	C17	110.3(5)	C16	C15	C17	112.3(9)
N4	C20	C21	112.2(6)	N4	C20	C22	111.2(8)
C21	C20	C22	112.8(6)	Si1	C23	C24	120.3(6)
Si1	C23	C28	124.2(5)	C24	C23	C28	115.4(8)
C23	C24	C25	123.4(8)	C24	C25	C26	119.1(8)
C25	C26	C27	119.8(9)	C26	C27	C28	120.3(9)
C23	C28	C27	121.9(7)	Si1	C29	C30	121.7(4)
Si1	C29	C34	121.7(8)	C30	C29	C34	116.6(8)
C29	C30	C31	122.4(6)	C30	C31	C32	120.1(10)
C31	C32	C33	118.5(9)	C32	C33	C34	121.8(7)
C29	C34	C33	120.6(9)	Si2	C35	C36	123.1(5)
Si2	C35	C40	121.1(5)	C36	C35	C40	115.5(5)
C35	C36	C37	121.8(7)	C36	C37	C38	121.4(7)
C37	C38	C39	119.1(6)	C38	C39	C40	119.9(7)
C35	C40	C39	122.2(7)	Si2	C41	C42	121.4(6)

Si2	C41	C46	122.6(6)	C42	C41	C46	115.7(9)
C41	C42	C43	121.6(7)	C42	C43	C44	121.3(8)
C43	C44	C45	118.8(10)	C44	C45	C46	119.6(8)
C41	C46	C45	122.9(7)				

Symmetry Operators:

(1) -X+1,-Y+1,-Z+1

Torsion Angles(^{o})

(Those having bond angles > 160 or < 20 degrees are excluded.)

atom1	atom2	atom3	atom4	angle	atom1	atom2	atom3	atom4	angle
Si1	Pt1	C1	N1	-111.9(4)	Si1	Pt1	C1	N2	71.8(4)
C1	Pt1	Si1	Si2	-125.9(2)	C1	Pt1	Si1	C23	3.5(2)
C1	Pt1	Si1	C29	118.4(2)	C1	Pt1	C2	N3	-120.1(5)
C1	Pt1	C2	N4	56.3(5)	C2	Pt1	C1	N1	57.7(4)
C2	Pt1	C1	N2	-118.5(5)	Pt1	Si1	Si2	Si2[1]	160.71(10)
Pt1	Si1	Si2	C35	31.22(17)	Pt1	Si1	Si2	C41	-79.18(12)
Pt1	Si1	C23	C24	41.1(5)	Pt1	Si1	C23	C28	-134.9(4)
Pt1	Si1	C29	C30	50.6(5)	Pt1	Si1	C29	C34	-128.8(4)
Si2	Si1	C23	C24	171.7(4)	Si2	Si1	C23	C28	-4.3(5)
C23	Si1	Si2	Si2[1]	27.9(3)	C23	Si1	Si2	C35	-101.6(2)
C23	Si1	Si2	C41	148.0(2)	Si2	Si1	C29	C30	-69.6(5)
Si2	Si1	C29	C34	111.1(4)	C29	Si1	Si2	Si2[1]	-83.3(3)
C29	Si1	Si2	C35	147.2(3)	C29	Si1	Si2	C41	36.8(3)
C23	Si1	C29	C30	174.4(5)	C23	Si1	C29	C34	-4.9(5)
C29	Si1	C23	C24	-75.4(5)	C29	Si1	C23	C28	108.6(5)
Si1	Si2	Si2[1]	Si1[1]	180.00(12)	Si1	Si2	Si2[1]	C35[1]	49.36(19)
Si1	Si2	Si2[1]	C41[1]	-64.14(15)	Si1	Si2	C35	C36	-28.3(7)
Si1	Si2	C35	C40	158.0(5)	Si1	Si2	C41	C42	81.6(4)
Si1	Si2	C41	C46	-92.6(4)	Si2[1]	Si2	C35	C36	-166.7(6)
Si2[1]	Si2	C35	C40	19.7(7)	C35	Si2	Si2[1]	Si1[1]	-49.4(3)
C35	Si2	Si2[1]	C35[1]	-180.0(3)	C35	Si2	Si2[1]	C41[1]	66.5(3)
Si2[1]	Si2	C41	C42	-147.0(4)	Si2[1]	Si2	C41	C46	38.8(5)
C41	Si2	Si2[1]	Si1[1]	64.1(2)	C41	Si2	Si2[1]	C35[1]	-66.5(2)
C41	Si2	Si2[1]	C41[1]	-180.00(18)	C35	Si2	C41	C42	-32.3(5)
C35	Si2	C41	C46	153.4(4)	C41	Si2	C35	C36	77.9(7)
C41	Si2	C35	C40	-95.7(7)	C1	N1	C3	C4	-0.1(6)
C1	N1	C3	C8	-178.1(5)	C3	N1	C1	Pt1	-177.6(4)
C3	N1	C1	N2	-0.7(6)	C1	N1	C5	C6	121.6(6)
C1	N1	C5	C7	-110.1(6)	C5	N1	C1	Pt1	3.8(7)

C5	N1	C1	N2	-179.2(4)	C3	N1	C5	C6	-56.7(8)
C3	N1	C5	C7	71.6(7)	C5	N1	C3	C4	178.4(5)
C5	N1	C3	C8	0.3(9)	C1	N2	C4	C3	-1.3(6)
C1	N2	C4	C9	-179.4(5)	C4	N2	C1	Pt1	178.1(4)
C4	N2	C1	N1	1.2(6)	C1	N2	C10	C11	-126.2(6)
C1	N2	C10	C12	106.9(7)	C10	N2	C1	Pt1	-5.8(8)
C10	N2	C1	N1	177.4(4)	C4	N2	C10	C11	49.4(8)
C4	N2	C10	C12	-77.5(8)	C10	N2	C4	C3	-177.3(4)
C10	N2	C4	C9	4.6(8)	C2	N3	C13	C14	-0.3(7)
C2	N3	C13	C18	-179.3(5)	C13	N3	C2	Pt1	177.6(5)
C13	N3	C2	N4	0.5(7)	C2	N3	C15	C16	131.7(7)
C2	N3	C15	C17	-101.6(8)	C15	N3	C2	Pt1	-8.2(9)
C15	N3	C2	N4	174.7(5)	C13	N3	C15	C16	-55.0(9)
C13	N3	C15	C17	71.7(8)	C15	N3	C13	C14	-174.1(5)
C15	N3	C13	C18	6.9(10)	C2	N4	C14	C13	0.3(7)
C2	N4	C14	C19	-179.5(5)	C14	N4	C2	Pt1	-177.4(5)
C14	N4	C2	N3	-0.5(7)	C2	N4	C20	C21	-115.1(6)
C2	N4	C20	C22	117.5(6)	C20	N4	C2	Pt1	4.5(9)
C20	N4	C2	N3	-178.6(5)	C14	N4	C20	C21	67.2(8)
C14	N4	C20	C22	-60.2(9)	C20	N4	C14	C13	178.3(6)
C20	N4	C14	C19	-1.6(11)	N1	C3	C4	N2	0.8(6)
N1	C3	C4	C9	178.8(5)	C8	C3	C4	N2	178.8(6)
C8	C3	C4	C9	-3.2(10)	N3	C13	C14	N4	-0.0(7)
N3	C13	C14	C19	179.8(6)	C18	C13	C14	N4	179.0(6)
C18	C13	C14	C19	-1.2(12)	Si1	C23	C24	C25	-174.9(4)
Si1	C23	C28	C27	174.3(4)	C24	C23	C28	C27	-1.8(9)
C28	C23	C24	C25	1.4(10)	C23	C24	C25	C26	0.3(11)
C24	C25	C26	C27	-1.7(11)	C25	C26	C27	C28	1.3(11)
C26	C27	C28	C23	0.5(11)	Si1	C29	C30	C31	179.4(4)
Si1	C29	C34	C33	-179.2(4)	C30	C29	C34	C33	1.4(10)
C34	C29	C30	C31	-1.3(10)	C29	C30	C31	C32	0.6(11)
C30	C31	C32	C33	0.1(11)	C31	C32	C33	C34	0.1(12)
C32	C33	C34	C29	-0.8(12)	Si2	C35	C36	C37	-174.3(6)
Si2	C35	C40	C39	174.7(6)	C36	C35	C40	C39	0.6(14)
C40	C35	C36	C37	-0.3(14)	C35	C36	C37	C38	0.1(16)
C36	C37	C38	C39	-0.1(16)	C37	C38	C39	C40	0.4(15)
C38	C39	C40	C35	-0.7(15)	Si2	C41	C42	C43	-173.2(4)
Si2	C41	C46	C45	173.8(4)	C42	C41	C46	C45	-0.8(9)
C46	C41	C42	C43	1.4(9)	C41	C42	C43	C44	-1.2(10)

C42	C43	C44	C45	0.2(11)	C43	C44	C45	C46	0.4(10)
C44	C45	C46	C41	-0.2(10)					

Symmetry Operators:

(1) -X+1,-Y+1,-Z+1

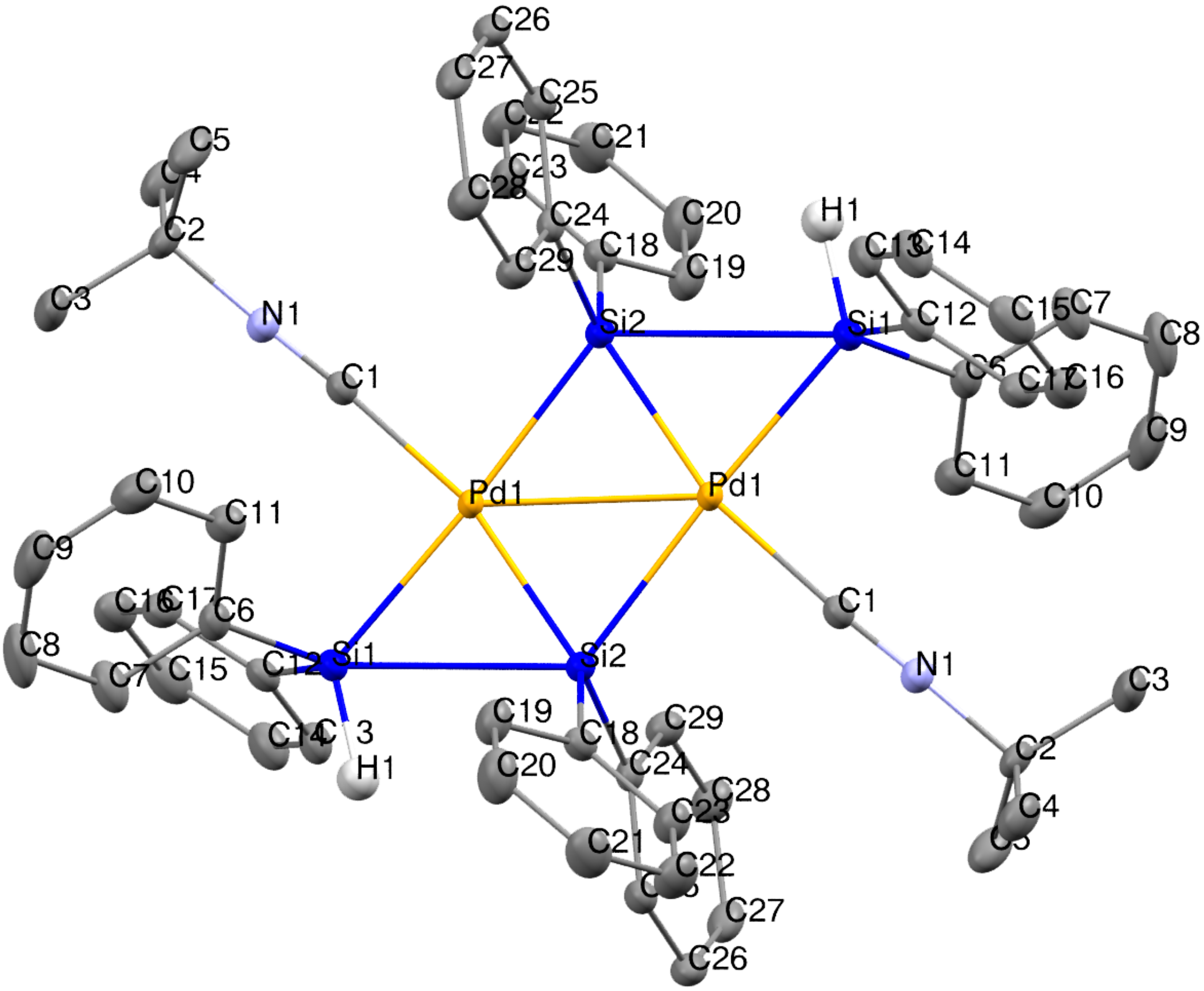

Supplementary Fig. 47. ORTEP drawing of **5a** (50% probability of the thermal ellipsoids). Hydrogen atoms were omitted for clarity.

Supplementary Table 12. Crystal data and structure refinement for **5a**.

Empirical Formula	$C_{58}H_{60}N_2Pd_2Si_4$
Formula Weight	1110.27
Crystal Color, Habit	yellow, plate
Crystal Dimensions	0.100 X 0.050 X 0.050 mm
Crystal System	monoclinic
Lattice Type	Primitive
Lattice Parameters	a = 10.2968(4) Å
	b = 20.0349(9) Å
	c = 13.3501(6) Å
	β = 104.181(7) ^{o}
	V = 2670.1(2) $Å^3$
Space Group	$P2_1/n$ (#14)
Z value	2
D_{calc}	1.381 g/cm^3
F_{000}	1140.00
μ(unknown radiation, λ = 0.41340Å)	9.680 cm^{-1}
Diffractometer	R-AXIS IV
unknown Radiation (λ = 0.41340Å)	
	monochromated
Voltage, Current	8kV, 100mA
Temperature	-173.0^{o}C
Detector Aperture	300.0 x 300.0 mm
Data Images	1440 exposures
ω oscillation Range (χ=45.0, ϕ=0.0)	0.0 - 180.0^{o}
Exposure Rate	120.0 sec./^{o}
ω oscillation Range (χ=45.0, ϕ=90.0)	0.0 - 180.0^{o}
Exposure Rate	120.0 sec./^{o}
ω oscillation Range (χ=45.0, ϕ=180.0)	0.0 - 180.0^{o}
Exposure Rate	120.0 sec./^{o}
ω oscillation Range (χ=45.0, ϕ=270.0)	0.0 - 180.0^{o}
Exposure Rate	120.0 sec./^{o}
Detector Position	130.00 mm
Pixel Size	0.172 mm
$2\theta_{max}$	31.1^{o}
No. of Reflections Measured	Total: 75994
	Unique: 6076 (R_{int} = 0.0367)
Corrections	Lorentz-polarization

	Absorption
	(trans. factors: 0.835 - 1.000)
Structure Solution	Direct Methods (SHELXT Version 2014/5)
Refinement	Full-matrix least-squares on F^2
Function Minimized	Σ w $(Fo^2 - Fc^2)^2$
Least Squares Weights	w = 1/ [$\sigma^2(Fo^2) + (0.0153 \cdot P)^2$
	+ 1.6564 · P]
	where P = $(Max(Fo^2,0) + 2Fc^2)/3$
$2\theta_{max}$ cutoff	31.1°
Anomalous Dispersion	All non-hydrogen atoms
No. Observations (All reflections)	6076
No. Variables	305
Reflection/Parameter Ratio	19.92
Residuals: R1 (I>2.00σ(I))	0.0167
Residuals: R (All reflections)	0.0175
Residuals: wR2 (All reflections)	0.0417
Goodness of Fit Indicator	0.963
Max Shift/Error in Final Cycle	0.004
Maximum peak in Final Diff. Map	0.31 $e^-/Å^3$
Minimum peak in Final Diff. Map	-0.43 $e^-/Å^3$

Atomic coordinates and B_{iso}/B_{eq}

atom	x	y	z	B_{eq}
Pd1	0.41331(2)	0.49712(2)	0.40386(2)	1.005(2)
Si1	0.29642(3)	0.40406(2)	0.30891(2)	1.185(5)
Si2	0.48151(3)	0.40501(2)	0.50468(2)	1.080(5)
N1	0.25975(10)	0.59708(5)	0.23408(7)	1.471(15)
C1	0.32255(11)	0.56197(6)	0.29523(8)	1.356(17)
C2	0.17089(12)	0.64324(6)	0.16434(9)	1.523(17)
C3	0.06020(14)	0.60212(7)	0.09447(11)	2.46(2)
C4	0.25398(13)	0.68003(7)	0.10183(10)	2.12(2)
C5	0.11557(14)	0.69078(7)	0.23267(10)	2.29(2)
C6	0.11077(11)	0.42355(6)	0.28070(9)	1.451(17)
C7	0.01956(13)	0.38522(7)	0.20873(10)	2.17(2)
C8	-0.11716(13)	0.39866(8)	0.18477(10)	2.67(3)
C9	-0.16630(13)	0.45058(8)	0.23251(11)	2.46(2)
C10	-0.07890(14)	0.48929(7)	0.30454(12)	2.28(2)
C11	0.05833(12)	0.47581(6)	0.32825(10)	1.772(18)
C12	0.34906(11)	0.40322(6)	0.18289(9)	1.383(17)
C13	0.46402(12)	0.36861(6)	0.17457(9)	1.688(18)
C14	0.50796(12)	0.37046(7)	0.08408(10)	2.00(2)
C15	0.43879(14)	0.40742(7)	0.00011(10)	2.15(2)
C16	0.32415(14)	0.44203(6)	0.00656(10)	2.07(2)
C17	0.28000(12)	0.43961(6)	0.09693(9)	1.700(18)
C18	0.37802(11)	0.35632(6)	0.57718(8)	1.329(17)
C19	0.23945(12)	0.36456(7)	0.55828(9)	1.822(19)
C20	0.16478(13)	0.33031(8)	0.61578(10)	2.31(2)
C21	0.22681(13)	0.28736(7)	0.69353(10)	2.28(2)
C22	0.36460(14)	0.27815(7)	0.71408(10)	2.18(2)
C23	0.43864(12)	0.31224(6)	0.65668(9)	1.746(18)
C24	0.61701(11)	0.34923(6)	0.47957(8)	1.254(16)
C25	0.62178(11)	0.27994(6)	0.49391(9)	1.498(17)
C26	0.73172(12)	0.24266(6)	0.48384(9)	1.747(18)
C27	0.83856(12)	0.27393(6)	0.45756(9)	1.799(19)
C28	0.83456(12)	0.34218(6)	0.43890(10)	1.846(19)
C29	0.72514(12)	0.37931(6)	0.45033(9)	1.574(17)

$B_{eq} = 8/3\ \pi^2(U_{11}(aa^*)^2 + U_{22}(bb^*)^2 + U_{33}(cc^*)^2 + 2U_{12}(aa^*bb^*)\cos\gamma + 2U_{13}(aa^*cc^*)\cos\beta + 2U_{23}(bb^*cc^*)\cos\alpha)$

Anisotropic displacement parameters

atom	U_{11}	U_{22}	U_{33}	U_{12}	U_{13}	U_{23}
Pd1	0.01227(5)	0.01190(5)	0.01224(5)	0.00002(3)	-0.00040(3)	0.00081(3)
Si1	0.01473(14)	0.01432(14)	0.01470(14)	-0.00110(11)	0.00116(11)	-0.00073(11)
Si2	0.01338(13)	0.01255(14)	0.01401(14)	-0.00030(10)	0.00123(11)	0.00122(10)
N1	0.0190(5)	0.0179(5)	0.0176(5)	0.0011(4)	0.0016(4)	0.0015(4)
C1	0.0175(5)	0.0162(5)	0.0163(5)	-0.0013(4)	0.0011(4)	-0.0011(4)
C2	0.0204(5)	0.0190(5)	0.0166(5)	0.0053(4)	0.0008(4)	0.0048(4)
C3	0.0285(7)	0.0266(7)	0.0301(7)	0.0006(5)	-0.0088(5)	0.0058(5)
C4	0.0267(6)	0.0319(7)	0.0222(6)	0.0057(5)	0.0063(5)	0.0096(5)
C5	0.0321(7)	0.0323(7)	0.0231(6)	0.0144(6)	0.0078(5)	0.0039(5)
C6	0.0166(5)	0.0209(5)	0.0164(5)	-0.0019(4)	0.0017(4)	0.0027(4)
C7	0.0216(6)	0.0379(7)	0.0223(6)	-0.0073(5)	0.0041(5)	-0.0060(5)
C8	0.0195(6)	0.0570(9)	0.0223(6)	-0.0112(6)	-0.0004(5)	-0.0011(6)
C9	0.0151(5)	0.0474(8)	0.0296(7)	0.0004(5)	0.0027(5)	0.0176(6)
C10	0.0240(6)	0.0259(6)	0.0391(8)	0.0045(5)	0.0124(6)	0.0094(6)
C11	0.0195(6)	0.0204(6)	0.0270(6)	-0.0009(5)	0.0048(5)	0.0020(5)
C12	0.0183(5)	0.0158(5)	0.0176(5)	-0.0040(4)	0.0029(4)	-0.0029(4)
C13	0.0173(5)	0.0248(6)	0.0202(6)	-0.0016(4)	0.0011(4)	-0.0028(5)
C14	0.0171(5)	0.0323(7)	0.0276(6)	-0.0048(5)	0.0074(5)	-0.0076(5)
C15	0.0296(7)	0.0314(7)	0.0237(6)	-0.0112(5)	0.0124(5)	-0.0045(5)
C16	0.0344(7)	0.0230(6)	0.0207(6)	-0.0038(5)	0.0056(5)	0.0036(5)
C17	0.0250(6)	0.0179(5)	0.0215(6)	0.0005(4)	0.0052(5)	-0.0003(4)
C18	0.0182(5)	0.0159(5)	0.0159(5)	-0.0025(4)	0.0032(4)	-0.0005(4)
C19	0.0191(6)	0.0292(6)	0.0204(6)	0.0019(5)	0.0038(5)	0.0044(5)
C20	0.0184(6)	0.0434(8)	0.0262(6)	-0.0029(5)	0.0060(5)	0.0039(6)
C21	0.0273(6)	0.0347(7)	0.0266(6)	-0.0085(5)	0.0104(5)	0.0056(5)
C22	0.0289(6)	0.0280(6)	0.0260(6)	-0.0004(5)	0.0066(5)	0.0106(5)
C23	0.0189(5)	0.0244(6)	0.0221(6)	-0.0004(5)	0.0032(5)	0.0046(5)
C24	0.0156(5)	0.0165(5)	0.0136(5)	0.0010(4)	-0.0001(4)	-0.0004(4)
C25	0.0171(5)	0.0166(5)	0.0209(5)	-0.0011(4)	0.0003(4)	-0.0008(4)
C26	0.0233(6)	0.0163(5)	0.0240(6)	0.0038(4)	0.0005(5)	-0.0012(4)
C27	0.0206(6)	0.0262(6)	0.0209(6)	0.0082(5)	0.0038(5)	0.0002(5)
C28	0.0203(6)	0.0273(6)	0.0239(6)	0.0030(5)	0.0079(5)	0.0045(5)
C29	0.0211(6)	0.0182(5)	0.0208(5)	0.0018(4)	0.0058(4)	0.0031(4)

The general temperature factor expression: $\exp(-2\pi^2(a^{*2}U_{11}h^2 + b^{*2}U_{22}k^2 + c^{*2}U_{33}l^2 + 2a^*b^*U_{12}hk + 2a^*c^*U_{13}hl + 2b^*c^*U_{23}kl))$

Bond lengths (Å)

atom	atom	distance	atom	atom	distance
Pd1	Pd1[1]	2.7467(4)	Pd1	Si1	2.4050(5)
Pd1	Si2	2.2907(5)	Pd1	Si2[1]	2.4209(5)
Pd1	C1	2.0001(11)	Si1	Si2	2.8324(4)
Si1	C6	1.8963(12)	Si1	C12	1.8898(13)
Si2	C18	1.8786(13)	Si2	C24	1.8807(13)
N1	C1	1.1492(14)	N1	C2	1.4635(14)
C2	C3	1.5254(17)	C2	C4	1.524(2)
C2	C5	1.523(2)	C6	C7	1.3974(16)
C6	C11	1.3995(18)	C7	C8	1.3914(19)
C8	C9	1.379(2)	C9	C10	1.3828(19)
C10	C11	1.3967(19)	C12	C13	1.3996(17)
C12	C17	1.3980(16)	C13	C14	1.3904(19)
C14	C15	1.3860(18)	C15	C16	1.390(2)
C16	C17	1.390(2)	C18	C19	1.3966(17)
C18	C23	1.4041(16)	C19	C20	1.393(2)
C20	C21	1.3788(19)	C21	C22	1.3898(19)
C22	C23	1.386(2)	C24	C25	1.4006(17)
C24	C29	1.4029(18)	C25	C26	1.3900(18)
C26	C27	1.3838(18)	C27	C28	1.3887(17)
C28	C29	1.3894(18)	Si1	H1	1.344(15)

Symmetry Operators:

(1) -X+1,-Y+1,-Z+1

Bond angles (°)

atom	atom	atom	angle	atom	atom	atom	angle
Pd1[1]	Pd1	Si1	130.686(19)	Pd1[1]	Pd1	Si2	56.579(13)
Pd1[1]	Pd1	Si2[1]	52.162(12)	Pd1[1]	Pd1	C1	137.02(4)
Si1	Pd1	Si2	74.154(17)	Si1	Pd1	Si2[1]	176.399(17)
Si1	Pd1	C1	92.11(3)	Si2	Pd1	Si2[1]	108.741(15)
Si2	Pd1	C1	166.14(4)	Si2[1]	Pd1	C1	84.92(4)
Pd1	Si1	Si2	51.077(12)	Pd1	Si1	C6	107.16(4)
Pd1	Si1	C12	105.10(4)	Si2	Si1	C6	126.84(4)
Si2	Si1	C12	123.14(3)	C6	Si1	C12	108.67(5)
Pd1	Si2	Pd1[1]	71.259(16)	Pd1	Si2	Si1	54.768(12)
Pd1	Si2	C18	126.17(4)	Pd1	Si2	C24	120.53(4)
Pd1[1]	Si2	Si1	125.985(18)	Pd1[1]	Si2	C18	113.63(4)
Pd1[1]	Si2	C24	108.15(4)	Si1	Si2	C18	97.67(3)

Si1	Si2	C24	101.09(3)	C18	Si2	C24	108.81(6)
C1	N1	C2	173.70(13)	Pd1	C1	N1	173.87(11)
N1	C2	C3	107.74(10)	N1	C2	C4	107.86(10)
N1	C2	C5	106.35(10)	C3	C2	C4	111.23(11)
C3	C2	C5	111.78(11)	C4	C2	C5	111.60(11)
Si1	C6	C7	119.89(10)	Si1	C6	C11	123.10(8)
C7	C6	C11	117.00(11)	C6	C7	C8	121.56(13)
C7	C8	C9	120.32(12)	C8	C9	C10	119.63(12)
C9	C10	C11	119.94(14)	C6	C11	C10	121.55(11)
Si1	C12	C13	120.51(8)	Si1	C12	C17	121.80(9)
C13	C12	C17	117.60(12)	C12	C13	C14	121.15(11)
C13	C14	C15	120.32(12)	C14	C15	C16	119.50(13)
C15	C16	C17	119.97(11)	C12	C17	C16	121.45(12)
Si2	C18	C19	121.93(9)	Si2	C18	C23	120.85(9)
C19	C18	C23	117.15(11)	C18	C19	C20	121.31(11)
C19	C20	C21	120.39(12)	C20	C21	C22	119.58(13)
C21	C22	C23	119.91(12)	C18	C23	C22	121.67(11)
Si2	C24	C25	124.56(9)	Si2	C24	C29	117.93(9)
C25	C24	C29	117.40(11)	C24	C25	C26	121.43(11)
C25	C26	C27	119.88(11)	C26	C27	C28	120.05(12)
C27	C28	C29	119.78(12)	C24	C29	C28	121.38(11)

Symmetry Operators:

(1) -X+1,-Y+1,-Z+1

Torsion Angles(°)

(Those having bond angles > 160 or < 20 degrees are excluded.)

atom1	atom2	atom3	atom4	angle	atom1	atom2	atom3	atom4	angle
Pd1[1]	Pd1	Si1	Si2	-2.469(14)	Pd1[1]	Pd1	Si1	C6	121.393(18)
Pd1[1]	Pd1	Si1	C12	-123.123(17)	Si1	Pd1	Pd1[1]	Si1[1]	180.000(15)
Si1	Pd1	Pd1[1]	Si2[1]	-177.154(19)	Si1	Pd1	Pd1[1]	Si2	2.846(16)
Si1	Pd1	Pd1[1]	C1[1]	6.39(3)	Pd1[1]	Pd1	Si2	Pd1[1]	0.0
Pd1[1]	Pd1	Si2	Si1	177.757(12)	Pd1[1]	Pd1	Si2	C18	106.04(2)
Pd1[1]	Pd1	Si2	C24	-100.469(19)	Si2	Pd1	Pd1[1]	Si1[1]	177.154(19)
Si2	Pd1	Pd1[1]	Si2[1]	180.000(15)	Si2	Pd1	Pd1[1]	Si2	0.0
Si2	Pd1	Pd1[1]	C1[1]	3.545(19)	Pd1[1]	Pd1	Si2[1]	Pd1[1]	-0.0
Pd1[1]	Pd1	Si2[1]	Si1[1]	2.264(12)	Pd1[1]	Pd1	Si2[1]	C18[1]	122.12(2)
Pd1[1]	Pd1	Si2[1]	C24[1]	-116.958(18)	Si2[1]	Pd1	Pd1[1]	Si1[1]	-2.846(15)
Si2[1]	Pd1	Pd1[1]	Si2[1]	-0.0	Si2[1]	Pd1	Pd1[1]	Si2	-180.000(15)
Si2[1]	Pd1	Pd1[1]	C1[1]	-176.45(2)	C1	Pd1	Pd1[1]	Si1[1]	-6.39(6)

C1	Pd1	Pd1[1]	Si2[1]	-3.55(5)	C1	Pd1	Pd1[1]	Si2	176.45(6)
C1	Pd1	Pd1[1]	C1[1]	-180.00(5)	Si1	Pd1	Si2	Pd1[1]	-177.757(13)
Si1	Pd1	Si2	Si1	0.0	Si1	Pd1	Si2	C18	-71.72(2)
Si1	Pd1	Si2	C24	81.774(18)	Si2	Pd1	Si1	Si2	0.0
Si2	Pd1	Si1	C6	123.863(17)	Si2	Pd1	Si1	C12	-120.654(17)
C1	Pd1	Si1	Si2	-178.11(4)	C1	Pd1	Si1	C6	-54.25(4)
C1	Pd1	Si1	C12	61.23(4)	Si2	Pd1	Si2[1]	Pd1[1]	-0.000(14)
Si2	Pd1	Si2[1]	Si1[1]	2.26(2)	Si2	Pd1	Si2[1]	C18[1]	122.123(17)
Si2	Pd1	Si2[1]	C24[1]	-116.958(17)	Si2[1]	Pd1	Si2	Pd1[1]	0.000(13)
Si2[1]	Pd1	Si2	Si1	177.757(15)	Si2[1]	Pd1	Si2	C18	106.04(2)
Si2[1]	Pd1	Si2	C24	-100.47(2)	C1	Pd1	Si2[1]	Pd1[1]	177.57(4)
C1	Pd1	Si2[1]	Si1[1]	179.84(4)	C1	Pd1	Si2[1]	C18[1]	-60.30(4)
C1	Pd1	Si2[1]	C24[1]	60.62(4)	Pd1	Si1	Si2	Pd1	0.0
Pd1	Si1	Si2	Pd1[1]	2.625(15)	Pd1	Si1	Si2	C18	129.332(19)
Pd1	Si1	Si2	C24	-119.690(18)	Pd1	Si1	C6	C7	166.85(7)
Pd1	Si1	C6	C11	-12.55(10)	Pd1	Si1	C12	C13	87.93(8)
Pd1	Si1	C12	C17	-88.56(8)	Si2	Si1	C6	C7	-139.33(6)
Si2	Si1	C6	C11	41.28(11)	C6	Si1	Si2	Pd1	-82.46(5)
C6	Si1	Si2	Pd1[1]	-79.84(5)	C6	Si1	Si2	C18	46.87(5)
C6	Si1	Si2	C24	157.85(5)	Si2	Si1	C12	C13	34.87(10)
Si2	Si1	C12	C17	-141.62(6)	C12	Si1	Si2	Pd1	82.69(5)
C12	Si1	Si2	Pd1[1]	85.31(5)	C12	Si1	Si2	C18	-147.98(5)
C12	Si1	Si2	C24	-37.00(5)	C6	Si1	C12	C13	-157.64(8)
C6	Si1	C12	C17	25.88(10)	C12	Si1	C6	C7	53.77(10)
C12	Si1	C6	C11	-125.63(9)	Pd1	Si2	C18	C19	13.55(10)
Pd1	Si2	C18	C23	-163.28(5)	Pd1	Si2	C24	C25	-145.71(6)
Pd1	Si2	C24	C29	38.27(8)	Pd1[1]	Si2	C18	C19	96.97(7)
Pd1[1]	Si2	C18	C23	-79.86(8)	Pd1[1]	Si2	C24	C25	135.78(6)
Pd1[1]	Si2	C24	C29	-40.24(7)	Si1	Si2	C18	C19	-37.95(8)
Si1	Si2	C18	C23	145.22(7)	Si1	Si2	C24	C25	-90.25(7)
Si1	Si2	C24	C29	93.74(6)	C18	Si2	C24	C25	11.91(9)
C18	Si2	C24	C29	-164.11(6)	C24	Si2	C18	C19	-142.48(8)
C24	Si2	C18	C23	40.68(9)	Si1	C6	C7	C8	-178.96(8)
Si1	C6	C11	C10	179.12(8)	C7	C6	C11	C10	-0.29(18)
C11	C6	C7	C8	0.47(19)	C6	C7	C8	C9	-0.3(2)
C7	C8	C9	C10	-0.1(2)	C8	C9	C10	C11	0.2(2)
C9	C10	C11	C6	-0.0(2)	Si1	C12	C13	C14	-176.54(7)
Si1	C12	C17	C16	175.99(7)	C13	C12	C17	C16	-0.59(16)
C17	C12	C13	C14	0.08(16)	C12	C13	C14	C15	0.59(18)

C13	C14	C15	C16	-0.76(19)	C14	C15	C16	C17	0.25(18)
C15	C16	C17	C12	0.43(18)	Si2	C18	C19	C20	-177.11(8)
Si2	C18	C23	C22	177.08(7)	C19	C18	C23	C22	0.10(17)
C23	C18	C19	C20	-0.17(17)	C18	C19	C20	C21	0.3(2)
C19	C20	C21	C22	-0.3(2)	C20	C21	C22	C23	0.2(2)
C21	C22	C23	C18	-0.12(19)	Si2	C24	C25	C26	-173.42(7)
Si2	C24	C29	C28	174.50(7)	C25	C24	C29	C28	-1.81(15)
C29	C24	C25	C26	2.62(15)	C24	C25	C26	C27	-1.00(16)
C25	C26	C27	C28	-1.52(16)	C26	C27	C28	C29	2.31(17)
C27	C28	C29	C24	-0.61(17)					

Symmetry Operators:

(1) -X+1,-Y+1,-Z+1

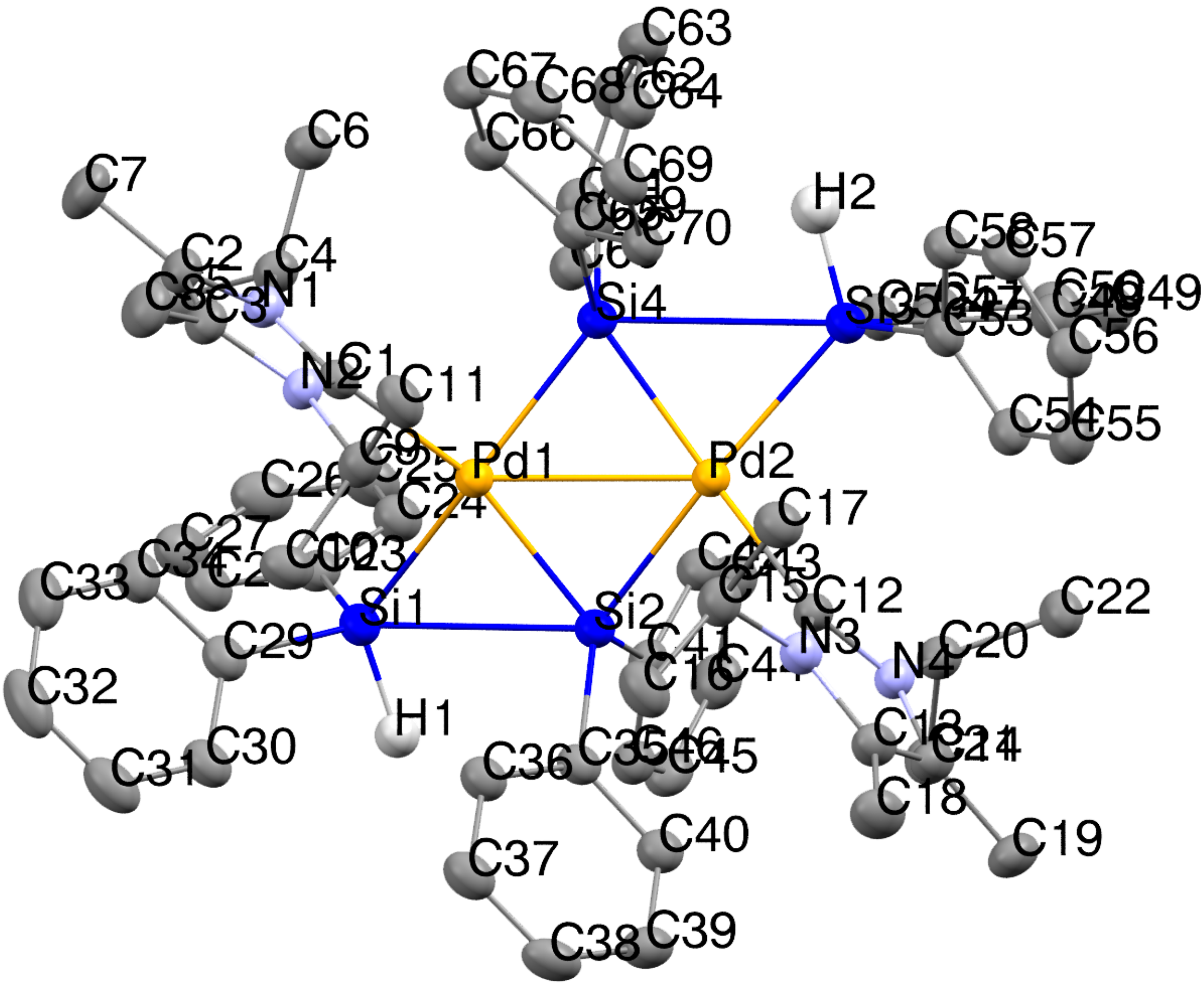

Supplementary Fig. 48. ORTEP drawing of **5b** (50% probability of the thermal ellipsoids). Hydrogen atoms were omitted for clarity.

Supplementary Table 13. Crystal data and structure refinement for **5b**.

Empirical Formula	$C_{84}H_{98}N_4Pd_2Si_4$
Formula Weight	1488.87
Crystal Color, Habit	yellow, block
Crystal Dimensions	0.020 X 0.020 X 0.020 mm
Crystal System	monoclinic
Lattice Type	Primitive
Lattice Parameters	a = 20.3309(8) Å
	b = 14.7044(6) Å
	c = 25.0642(10) Å
	β = 90.216(6) ^{o}
	V = 7493.0(5) Å^3
Space Group	$P2_1/n$ (#14)
Z value	4
D_{calc}	1.320 g/cm^3
F_{000}	3112.00
μ(unknown radiation, λ = 0.41350Å)	6.820 cm^{-1}
Diffractometer	R-AXIS IV
unknown Radiation (λ = 0.41350Å)	
	monochromated
Voltage, Current	8kV, 100mA
Temperature	-173.0^{o}C
Detector Aperture	300.0 x 300.0 mm
Data Images	1440 exposures
ω oscillation Range (χ=45.0, ϕ=0.0)	0.0 - 180.0^{o}
Exposure Rate	360.0 sec./^{o}
ω oscillation Range (χ=45.0, ϕ=90.0)	0.0 - 180.0^{o}
Exposure Rate	360.0 sec./^{o}
ω oscillation Range (χ=45.0, ϕ=180.0)	0.0 - 180.0^{o}
Exposure Rate	360.0 sec./^{o}
ω oscillation Range (χ=45.0, ϕ=270.0)	0.0 - 180.0^{o}
Exposure Rate	360.0 sec./^{o}
Detector Position	130.00 mm
Pixel Size	0.172 mm
$2\theta_{max}$	31.2^{o}
No. of Reflections Measured	Total: 218345
	Unique: 17123 (R_{int} = 0.1033)
Corrections	Lorentz-polarization

	Absorption
	(trans. factors: 0.664 - 1.000)
Structure Solution	Direct Methods (SHELXT Version 2014/5)
Refinement	Full-matrix least-squares on F^2
Function Minimized	Σ w $(Fo^2 - Fc^2)^2$
Least Squares Weights	w = 1/ [$\sigma^2(Fo^2)$ + $(0.0400 \cdot P)^2$
	+ 0.0000 · P]
	where P = $(Max(Fo^2,0) + 2Fc^2)/3$
$2\theta_{max}$ cutoff	31.1^{o}
Anomalous Dispersion	All non-hydrogen atoms
No. Observations (All reflections)	17123
No. Variables	1239
Reflection/Parameter Ratio	13.82
Residuals: R1 (I>2.00σ(I))	0.0299
Residuals: R (All reflections)	0.0409
Residuals: wR2 (All reflections)	0.0738
Goodness of Fit Indicator	0.978
Max Shift/Error in Final Cycle	0.003
Maximum peak in Final Diff. Map	0.45 $e^{-}/Å^3$
Minimum peak in Final Diff. Map	-1.03 $e^{-}/Å^3$

Atomic coordinates and B_{iso}/B_{eq}

atom	x	y	z	B_{eq}
Pd1	0.50381(2)	0.42444(2)	0.71583(2)	1.762(3)
Pd2	0.49306(2)	0.23298(2)	0.70850(2)	1.753(3)
Si1	0.60337(3)	0.51815(4)	0.71649(2)	2.033(10)
Si2	0.58968(3)	0.32649(4)	0.70044(2)	1.866(9)
Si3	0.41109(3)	0.12893(4)	0.74280(2)	1.970(10)
Si4	0.41297(3)	0.32829(4)	0.74052(2)	1.842(9)
N1	0.41286(8)	0.59616(12)	0.74091(7)	2.01(3)
N2	0.41057(8)	0.55166(12)	0.65901(7)	1.98(3)
N3	0.53769(8)	0.14453(12)	0.60645(7)	2.12(3)
N4	0.59427(8)	0.08067(12)	0.66916(7)	2.09(3)
C1	0.43649(9)	0.53108(14)	0.70773(8)	1.91(3)
C2	0.37222(10)	0.65725(14)	0.71330(9)	2.28(3)
C3	0.37093(10)	0.62915(15)	0.66185(9)	2.30(3)
C4	0.42849(10)	0.59648(15)	0.79890(8)	2.26(3)
C5	0.46138(13)	0.68419(18)	0.81753(11)	2.92(4)
C6	0.36804(11)	0.57465(18)	0.83198(10)	2.59(4)
C7	0.33832(14)	0.73757(19)	0.73705(11)	3.24(5)
C8	0.33342(14)	0.66944(19)	0.61638(11)	3.10(4)
C9	0.42665(10)	0.49801(15)	0.61096(8)	2.24(3)
C10	0.47039(12)	0.55128(18)	0.57258(9)	2.67(4)
C11	0.36585(12)	0.46042(18)	0.58321(10)	2.73(4)
C12	0.54695(9)	0.14359(14)	0.66045(8)	1.96(3)
C13	0.57958(10)	0.08236(15)	0.58161(9)	2.43(4)
C14	0.61535(10)	0.04245(15)	0.62051(9)	2.38(4)
C15	0.48853(11)	0.20523(16)	0.58073(9)	2.38(3)
C16	0.51592(13)	0.25816(19)	0.53303(10)	3.07(4)
C17	0.42604(11)	0.15430(18)	0.56525(10)	2.66(4)
C18	0.58546(14)	0.0701(2)	0.52288(10)	3.13(4)
C19	0.67153(12)	-0.02258(18)	0.61495(11)	3.01(4)
C20	0.61604(10)	0.05359(15)	0.72348(9)	2.31(3)
C21	0.69001(11)	0.06515(18)	0.73230(11)	2.76(4)
C22	0.59344(12)	-0.04239(16)	0.73707(11)	2.67(4)
C23	0.61613(10)	0.56387(15)	0.78690(9)	2.25(3)
C24	0.60937(11)	0.50658(17)	0.83095(9)	2.69(4)
C25	0.62038(12)	0.5372(2)	0.88272(10)	3.26(4)
C26	0.63907(12)	0.6262(2)	0.89140(10)	3.33(5)
C27	0.64733(12)	0.68415(19)	0.84864(11)	3.28(5)

C28	0.63601(11)	0.65343(16)	0.79698(10)	2.71(4)
C29	0.59703(10)	0.61920(15)	0.66960(9)	2.31(3)
C30	0.63586(11)	0.62609(17)	0.62385(9)	2.66(4)
C31	0.62799(12)	0.69670(19)	0.58731(10)	3.27(5)
C32	0.58266(14)	0.76470(19)	0.59697(11)	3.69(5)
C33	0.54432(13)	0.76138(17)	0.64261(11)	3.35(5)
C34	0.55082(11)	0.68918(15)	0.67768(10)	2.69(4)
C35	0.63534(9)	0.32901(14)	0.63495(8)	2.06(3)
C36	0.62262(10)	0.39964(15)	0.59868(9)	2.34(3)
C37	0.65354(11)	0.40368(17)	0.54950(9)	2.72(4)
C38	0.69709(11)	0.33599(18)	0.53466(9)	2.87(4)
C39	0.71003(11)	0.26480(17)	0.56927(9)	2.71(4)
C40	0.67996(10)	0.26206(16)	0.61923(9)	2.37(3)
C41	0.64682(9)	0.29403(14)	0.75652(9)	2.07(3)
C42	0.61725(10)	0.26032(15)	0.80322(9)	2.32(3)
C43	0.65347(12)	0.24104(17)	0.84871(10)	2.87(4)
C44	0.72090(12)	0.25489(17)	0.84853(10)	3.03(4)
C45	0.75161(11)	0.28627(16)	0.80308(10)	2.76(4)
C46	0.71507(10)	0.30662(15)	0.75746(9)	2.34(3)
C47	0.44782(9)	0.06887(14)	0.80321(8)	2.08(3)
C48	0.44705(10)	-0.02580(15)	0.81011(9)	2.39(4)
C49	0.47215(11)	-0.06602(16)	0.85632(10)	2.78(4)
C50	0.49814(11)	-0.01263(17)	0.89692(10)	2.76(4)
C51	0.49969(11)	0.08107(17)	0.89089(10)	2.72(4)
C52	0.47482(10)	0.12066(16)	0.84468(9)	2.43(3)
C53	0.38244(10)	0.04113(14)	0.69206(8)	2.16(3)
C54	0.42517(11)	-0.01578(15)	0.66321(9)	2.42(4)
C55	0.40224(11)	-0.07259(16)	0.62298(9)	2.60(4)
C56	0.33584(11)	-0.07362(16)	0.60960(9)	2.61(4)
C57	0.29270(11)	-0.01901(16)	0.63725(9)	2.68(4)
C58	0.31568(10)	0.03677(15)	0.67826(9)	2.41(4)
C59	0.39834(10)	0.34004(14)	0.81462(8)	2.05(3)
C60	0.45100(10)	0.36731(15)	0.84701(9)	2.31(3)
C61	0.44643(12)	0.36975(16)	0.90225(9)	2.69(4)
C62	0.38829(12)	0.34334(16)	0.92645(10)	2.83(4)
C63	0.33529(11)	0.31577(16)	0.89565(9)	2.74(4)
C64	0.34009(11)	0.31453(15)	0.84036(9)	2.27(3)
C65	0.33071(9)	0.33945(14)	0.70520(8)	2.05(3)
C66	0.28784(10)	0.41122(15)	0.71658(9)	2.21(3)

C67	0.22862(10)	0.42167(16)	0.68915(9)	2.52(4)
C68	0.21068(10)	0.35958(17)	0.65008(9)	2.54(4)
C69	0.25243(10)	0.28760(16)	0.63800(9)	2.39(4)
C70	0.31187(10)	0.27811(15)	0.66518(8)	2.12(3)
C71	0.64654(14)	0.3564(2)	1.00928(11)	3.90(5)
C72	0.64140(17)	0.2629(2)	1.00882(12)	4.49(6)
C73	0.58254(18)	0.2207(2)	1.01913(13)	4.85(6)
C74	0.52774(17)	0.2704(3)	1.03019(13)	4.91(7)
C75	0.53182(16)	0.3660(3)	1.03148(12)	4.69(6)
C76	0.59169(16)	0.4077(2)	1.02068(11)	3.93(5)
C77	0.7114(2)	0.4024(4)	0.9982(2)	6.62(10)
C78	0.82573(11)	0.46484(17)	0.56159(9)	2.76(4)
C79	0.86049(11)	0.39533(17)	0.53684(10)	2.94(4)
C80	0.86580(12)	0.39126(19)	0.48192(11)	3.34(5)
C81	0.83550(13)	0.4567(2)	0.45030(11)	3.62(5)
C82	0.80080(12)	0.5256(2)	0.47454(11)	3.53(5)
C83	0.79613(11)	0.53032(18)	0.52967(10)	3.06(4)
C84	0.81839(16)	0.4682(2)	0.62117(11)	3.79(5)

$B_{eq} = 8/3\ \pi^2(U_{11}(aa^*)^2 + U_{22}(bb^*)^2 + U_{33}(cc^*)^2 + 2U_{12}(aa^*bb^*)\cos\gamma + 2U_{13}(aa^*cc^*)\cos\beta + 2U_{23}(bb^*cc^*)\cos\alpha)$

Anisotropic displacement parameters

atom	U_{11}	U_{22}	U_{33}	U_{12}	U_{13}	U_{23}
Pd1	0.02135(7)	0.02197(7)	0.02363(7)	0.00007(5)	-0.00112(5)	-0.00011(6)
Pd2	0.02100(7)	0.02207(7)	0.02353(8)	0.00020(5)	-0.00047(5)	-0.00040(6)
Si1	0.0245(3)	0.0254(3)	0.0273(3)	-0.0020(2)	-0.0020(2)	0.0000(2)
Si2	0.0214(2)	0.0246(3)	0.0249(3)	-0.0003(2)	-0.0004(2)	0.0005(2)
Si3	0.0246(3)	0.0245(3)	0.0257(3)	-0.0012(2)	-0.0002(2)	0.0004(2)
Si4	0.0216(3)	0.0241(3)	0.0243(3)	0.0000(2)	-0.0003(2)	-0.0003(2)
N1	0.0248(8)	0.0256(9)	0.0258(9)	0.0019(7)	-0.0015(7)	-0.0008(7)
N2	0.0264(8)	0.0243(9)	0.0245(9)	0.0018(7)	-0.0011(7)	-0.0008(7)
N3	0.0290(9)	0.0274(9)	0.0243(9)	-0.0009(7)	0.0004(7)	-0.0023(7)
N4	0.0256(8)	0.0244(9)	0.0293(9)	0.0007(7)	0.0009(7)	-0.0018(7)
C1	0.0228(9)	0.0249(10)	0.0250(10)	-0.0030(8)	-0.0006(7)	0.0004(8)
C2	0.0277(10)	0.0256(11)	0.0332(11)	0.0043(8)	-0.0024(8)	0.0005(9)
C3	0.0271(10)	0.0279(11)	0.0322(11)	0.0042(8)	-0.0027(8)	0.0015(9)
C4	0.0292(10)	0.0315(11)	0.0250(10)	0.0024(8)	-0.0015(8)	-0.0033(8)
C5	0.0375(13)	0.0384(14)	0.0351(13)	-0.0062(11)	-0.0007(10)	-0.0090(11)
C6	0.0332(11)	0.0341(12)	0.0311(12)	0.0020(10)	0.0025(9)	-0.0019(10)

C7	0.0484(14)	0.0345(13)	0.0403(14)	0.0171(12)	-0.0014(12)	-0.0029(11)
C8	0.0432(14)	0.0376(14)	0.0367(13)	0.0108(11)	-0.0092(11)	0.0033(11)
C9	0.0331(11)	0.0293(11)	0.0227(10)	0.0037(9)	-0.0028(8)	-0.0004(8)
C10	0.0305(11)	0.0433(14)	0.0277(11)	-0.0053(10)	-0.0004(9)	-0.0023(10)
C11	0.0411(12)	0.0343(13)	0.0285(12)	-0.0096(10)	-0.0043(10)	0.0028(10)
C12	0.0233(9)	0.0241(10)	0.0271(10)	-0.0005(8)	0.0014(8)	-0.0022(8)
C13	0.0325(11)	0.0300(11)	0.0299(11)	-0.0026(9)	0.0036(9)	-0.0066(9)
C14	0.0295(10)	0.0280(11)	0.0331(11)	-0.0010(8)	0.0055(9)	-0.0070(9)
C15	0.0355(11)	0.0297(11)	0.0251(11)	0.0013(9)	-0.0048(9)	-0.0004(9)
C16	0.0429(13)	0.0403(14)	0.0334(13)	-0.0101(11)	-0.0075(10)	0.0064(11)
C17	0.0321(11)	0.0377(13)	0.0314(12)	-0.0016(10)	-0.0015(9)	0.0008(10)
C18	0.0439(14)	0.0442(15)	0.0309(12)	-0.0038(12)	0.0036(10)	-0.0094(11)
C19	0.0330(12)	0.0372(14)	0.0442(15)	0.0068(10)	0.0052(10)	-0.0085(12)
C20	0.0282(10)	0.0311(11)	0.0285(11)	0.0026(8)	-0.0012(8)	-0.0003(9)
C21	0.0286(11)	0.0343(13)	0.0417(14)	0.0018(9)	-0.0044(10)	0.0015(11)
C22	0.0327(11)	0.0307(12)	0.0380(13)	0.0015(9)	-0.0018(10)	0.0045(10)
C23	0.0233(9)	0.0330(12)	0.0293(11)	0.0004(8)	-0.0024(8)	-0.0023(9)
C24	0.0331(11)	0.0360(13)	0.0330(12)	-0.0042(10)	-0.0008(9)	-0.0010(10)
C25	0.0408(13)	0.0524(16)	0.0307(12)	-0.0074(11)	-0.0013(10)	0.0009(11)
C26	0.0378(12)	0.0568(17)	0.0320(13)	-0.0078(11)	-0.0033(10)	-0.0092(12)
C27	0.0395(13)	0.0430(15)	0.0420(14)	-0.0102(11)	-0.0022(10)	-0.0104(11)
C28	0.0345(11)	0.0333(12)	0.0352(12)	-0.0071(9)	-0.0025(9)	-0.0013(10)
C29	0.0268(10)	0.0302(11)	0.0308(11)	-0.0066(8)	-0.0047(8)	0.0002(9)
C30	0.0304(11)	0.0364(13)	0.0343(12)	-0.0082(9)	-0.0037(9)	0.0041(10)
C31	0.0403(13)	0.0497(15)	0.0344(13)	-0.0178(11)	-0.0082(10)	0.0089(11)
C32	0.0517(15)	0.0404(14)	0.0479(15)	-0.0169(12)	-0.0229(12)	0.0170(12)
C33	0.0432(13)	0.0315(13)	0.0523(16)	-0.0036(11)	-0.0155(12)	0.0043(11)
C34	0.0348(11)	0.0297(12)	0.0376(13)	-0.0041(9)	-0.0077(10)	-0.0007(9)
C35	0.0222(9)	0.0299(11)	0.0262(10)	-0.0043(8)	-0.0024(8)	-0.0006(8)
C36	0.0285(10)	0.0328(12)	0.0274(11)	-0.0043(9)	-0.0024(8)	0.0002(9)
C37	0.0334(11)	0.0414(14)	0.0286(11)	-0.0083(10)	-0.0034(9)	0.0046(10)
C38	0.0313(11)	0.0530(16)	0.0250(11)	-0.0104(10)	0.0033(9)	-0.0034(10)
C39	0.0287(10)	0.0407(13)	0.0335(12)	-0.0001(9)	0.0029(9)	-0.0053(10)
C40	0.0273(10)	0.0332(12)	0.0296(11)	-0.0021(9)	-0.0013(8)	-0.0001(9)
C41	0.0239(9)	0.0229(10)	0.0318(11)	0.0022(8)	-0.0019(8)	-0.0023(8)
C42	0.0254(10)	0.0302(11)	0.0326(11)	0.0024(8)	-0.0010(8)	0.0027(9)
C43	0.0385(12)	0.0393(13)	0.0312(12)	0.0058(10)	-0.0012(10)	0.0039(10)
C44	0.0392(12)	0.0409(14)	0.0350(13)	0.0069(10)	-0.0130(10)	0.0008(10)
C45	0.0268(11)	0.0365(13)	0.0416(13)	0.0024(9)	-0.0070(9)	-0.0053(10)

C46	0.0269(10)	0.0286(11)	0.0333(12)	-0.0004(8)	-0.0020(9)	-0.0032(9)
C47	0.0222(9)	0.0290(11)	0.0281(10)	0.0002(8)	0.0014(8)	0.0009(8)
C48	0.0296(10)	0.0283(11)	0.0327(12)	-0.0002(8)	0.0002(9)	0.0001(9)
C49	0.0342(11)	0.0284(12)	0.0428(13)	0.0020(9)	0.0001(10)	0.0074(10)
C50	0.0290(11)	0.0398(13)	0.0361(12)	0.0015(9)	-0.0043(9)	0.0097(10)
C51	0.0336(11)	0.0358(12)	0.0340(12)	-0.0030(9)	-0.0047(9)	-0.0009(10)
C52	0.0326(11)	0.0278(11)	0.0317(11)	-0.0025(9)	-0.0015(9)	0.0016(9)
C53	0.0280(10)	0.0255(10)	0.0286(11)	-0.0016(8)	-0.0011(8)	0.0037(8)
C54	0.0296(10)	0.0286(11)	0.0335(12)	0.0008(9)	-0.0046(9)	0.0016(9)
C55	0.0357(11)	0.0306(12)	0.0323(12)	0.0024(9)	-0.0010(9)	-0.0028(9)
C56	0.0358(11)	0.0308(12)	0.0324(12)	-0.0044(9)	-0.0029(9)	-0.0037(9)
C57	0.0283(11)	0.0361(12)	0.0374(12)	-0.0059(9)	-0.0029(9)	-0.0012(10)
C58	0.0272(10)	0.0293(11)	0.0352(12)	-0.0024(9)	0.0011(9)	-0.0008(9)
C59	0.0269(10)	0.0221(10)	0.0288(11)	0.0022(8)	-0.0007(8)	-0.0014(8)
C60	0.0297(10)	0.0283(11)	0.0297(11)	0.0012(8)	-0.0025(8)	0.0003(9)
C61	0.0393(12)	0.0323(12)	0.0306(11)	-0.0008(10)	-0.0063(9)	-0.0008(9)
C62	0.0467(13)	0.0350(13)	0.0258(11)	0.0025(10)	-0.0012(10)	0.0005(9)
C63	0.0334(11)	0.0377(13)	0.0331(12)	0.0010(9)	0.0049(9)	0.0019(10)
C64	0.0276(10)	0.0310(12)	0.0276(11)	0.0007(8)	0.0005(8)	-0.0000(9)
C65	0.0240(9)	0.0283(11)	0.0254(10)	-0.0017(8)	0.0008(8)	0.0043(8)
C66	0.0276(10)	0.0298(11)	0.0265(10)	-0.0002(8)	0.0009(8)	-0.0004(9)
C67	0.0264(10)	0.0346(12)	0.0348(12)	0.0037(9)	-0.0010(9)	0.0049(10)
C68	0.0236(10)	0.0425(13)	0.0303(11)	-0.0017(9)	-0.0037(8)	0.0084(10)
C69	0.0300(10)	0.0337(12)	0.0270(11)	-0.0071(9)	-0.0037(8)	0.0026(9)
C70	0.0259(9)	0.0272(11)	0.0274(10)	-0.0012(8)	-0.0014(8)	0.0013(8)
C71	0.0576(16)	0.0532(17)	0.0373(14)	-0.0028(13)	0.0007(12)	0.0036(12)
C72	0.070(2)	0.0540(18)	0.0467(17)	0.0044(16)	0.0015(14)	0.0010(14)
C73	0.079(2)	0.0518(19)	0.0537(19)	-0.0097(17)	-0.0109(16)	0.0026(15)
C74	0.0542(18)	0.086(3)	0.0462(17)	-0.0242(18)	-0.0117(14)	0.0176(16)
C75	0.0523(17)	0.087(3)	0.0384(15)	0.0122(17)	-0.0028(13)	0.0062(15)
C76	0.0689(19)	0.0485(17)	0.0320(13)	0.0001(14)	-0.0053(12)	0.0006(12)
C77	0.061(2)	0.093(4)	0.098(3)	-0.012(2)	0.003(2)	0.022(3)
C78	0.0316(11)	0.0397(13)	0.0336(12)	-0.0060(9)	-0.0021(9)	0.0016(10)
C79	0.0334(11)	0.0368(13)	0.0413(13)	-0.0037(10)	-0.0034(10)	0.0032(11)
C80	0.0367(12)	0.0433(14)	0.0469(15)	-0.0071(11)	0.0060(11)	-0.0066(12)
C81	0.0435(14)	0.0595(17)	0.0343(13)	-0.0156(12)	0.0004(11)	0.0020(12)
C82	0.0364(13)	0.0564(17)	0.0413(14)	-0.0058(12)	-0.0039(11)	0.0134(13)
C83	0.0311(11)	0.0412(14)	0.0440(14)	-0.0024(10)	0.0030(10)	0.0052(11)
C84	0.0540(17)	0.0536(18)	0.0362(14)	-0.0039(14)	-0.0002(12)	0.0012(13)

The general temperature factor expression: $\exp(-2\pi^2(a^{*2}U_{11}h^2 + b^{*2}U_{22}k^2 + c^{*2}U_{33}l^2 + 2a^*b^*U_{12}hk + 2a^*c^*U_{13}hl + 2b^*c^*U_{23}kl))$

Bond lengths (Å)

atom	atom	distance	atom	atom	distance
Pd1	Pd2	2.8297(4)	Pd1	Si1	2.4487(7)
Pd1	Si2	2.2970(7)	Pd1	Si4	2.4086(7)
Pd1	C1	2.091(2)	Pd2	Si2	2.4069(7)
Pd2	Si3	2.4224(7)	Pd2	Si4	2.2955(7)
Pd2	C12	2.095(2)	Si1	C23	1.905(2)
Si1	C29	1.899(2)	Si2	C35	1.889(2)
Si2	C41	1.882(2)	Si3	C47	1.903(2)
Si3	C53	1.902(2)	Si4	C59	1.890(2)
Si4	C65	1.896(2)	N1	C1	1.357(3)
N1	C2	1.402(3)	N1	C4	1.487(3)
N2	C1	1.362(3)	N2	C3	1.398(3)
N2	C9	1.477(3)	N3	C12	1.366(3)
N3	C13	1.397(3)	N3	C15	1.485(3)
N4	C12	1.352(3)	N4	C14	1.411(3)
N4	C20	1.484(3)	C2	C3	1.354(3)
C2	C7	1.492(3)	C3	C8	1.492(4)
C4	C5	1.525(3)	C4	C6	1.519(3)
C9	C10	1.528(3)	C9	C11	1.520(3)
C13	C14	1.349(3)	C13	C18	1.488(3)
C14	C19	1.497(3)	C15	C16	1.533(3)
C15	C17	1.524(3)	C20	C21	1.529(3)
C20	C22	1.523(3)	C23	C24	1.396(3)
C23	C28	1.400(3)	C24	C25	1.391(3)
C25	C26	1.380(4)	C26	C27	1.380(4)
C27	C28	1.390(4)	C29	C30	1.398(3)
C29	C34	1.409(3)	C30	C31	1.393(4)
C31	C32	1.382(4)	C32	C33	1.388(4)
C33	C34	1.384(3)	C35	C36	1.404(3)
C35	C40	1.396(3)	C36	C37	1.387(3)
C37	C38	1.384(3)	C38	C39	1.384(3)
C39	C40	1.396(3)	C41	C42	1.408(3)
C41	C46	1.400(3)	C42	C43	1.384(3)
C43	C44	1.386(3)	C44	C45	1.381(3)
C45	C46	1.394(3)	C47	C48	1.403(3)

C47	C52	1.399(3)	C48	C49	1.395(3)
C49	C50	1.388(3)	C50	C51	1.386(4)
C51	C52	1.390(3)	C53	C54	1.408(3)
C53	C58	1.401(3)	C54	C55	1.389(3)
C55	C56	1.390(3)	C56	C57	1.378(3)
C57	C58	1.394(3)	C59	C60	1.400(3)
C59	C64	1.402(3)	C60	C61	1.388(3)
C61	C62	1.386(3)	C62	C63	1.384(3)
C63	C64	1.390(3)	C65	C66	1.399(3)
C65	C70	1.401(3)	C66	C67	1.393(3)
C67	C68	1.387(3)	C68	C69	1.391(3)
C69	C70	1.392(3)	C71	C72	1.378(4)
C71	C76	1.377(4)	C71	C77	1.510(6)
C72	C73	1.373(5)	C73	C74	1.362(5)
C74	C75	1.408(6)	C75	C76	1.390(5)
C78	C79	1.390(3)	C78	C83	1.388(3)
C78	C84	1.502(4)	C79	C80	1.382(4)
C80	C81	1.389(4)	C81	C82	1.377(4)
C82	C83	1.387(4)	Si1	H1	1.41(2)
Si3	H2	1.39(2)			

Bond angles (^{o})

atom	atom	atom	angle	atom	atom	atom	angle
Pd2	Pd1	Si1	128.61(2)	Pd2	Pd1	Si2	54.817(18)
Pd2	Pd1	Si4	51.212(18)	Pd2	Pd1	C1	133.59(6)
Si1	Pd1	Si2	74.05(2)	Si1	Pd1	Si4	164.41(3)
Si1	Pd1	C1	96.86(6)	Si2	Pd1	Si4	105.02(2)
Si2	Pd1	C1	162.11(6)	Si4	Pd1	C1	87.87(6)
Pd1	Pd2	Si2	51.260(17)	Pd1	Pd2	Si3	131.18(2)
Pd1	Pd2	Si4	54.872(18)	Pd1	Pd2	C12	128.40(6)
Si2	Pd2	Si3	162.44(3)	Si2	Pd2	Si4	105.12(2)
Si2	Pd2	C12	83.19(6)	Si3	Pd2	Si4	76.80(2)
Si3	Pd2	C12	99.77(6)	Si4	Pd2	C12	163.19(6)
Pd1	Si1	C23	108.33(7)	Pd1	Si1	C29	112.46(7)
C23	Si1	C29	107.81(10)	Pd1	Si2	Pd2	73.92(2)
Pd1	Si2	C35	120.66(7)	Pd1	Si2	C41	120.07(7)
Pd2	Si2	C35	119.21(7)	Pd2	Si2	C41	107.11(7)
C35	Si2	C41	110.49(9)	Pd2	Si3	C47	107.88(6)
Pd2	Si3	C53	113.63(7)	C47	Si3	C53	109.59(9)

Pd1	Si4	Pd2	73.92(2)	Pd1	Si4	C59	108.81(7)
Pd1	Si4	C65	120.33(7)	Pd2	Si4	C59	120.92(7)
Pd2	Si4	C65	121.00(7)	C59	Si4	C65	108.00(9)
C1	N1	C2	111.03(17)	C1	N1	C4	121.77(17)
C2	N1	C4	127.17(17)	C1	N2	C3	110.90(17)
C1	N2	C9	121.74(17)	C3	N2	C9	127.32(17)
C12	N3	C13	110.67(17)	C12	N3	C15	121.75(17)
C13	N3	C15	127.59(18)	C12	N4	C14	110.57(17)
C12	N4	C20	122.77(17)	C14	N4	C20	126.54(17)
Pd1	C1	N1	134.63(15)	Pd1	C1	N2	120.30(14)
N1	C1	N2	104.84(17)	N1	C2	C3	106.49(18)
N1	C2	C7	125.6(2)	C3	C2	C7	127.9(2)
N2	C3	C2	106.75(18)	N2	C3	C8	125.3(2)
C2	C3	C8	127.9(2)	N1	C4	C5	113.19(18)
N1	C4	C6	111.25(17)	C5	C4	C6	111.48(19)
N2	C9	C10	111.74(18)	N2	C9	C11	112.65(17)
C10	C9	C11	111.86(18)	Pd2	C12	N3	119.53(14)
Pd2	C12	N4	135.23(15)	N3	C12	N4	105.21(17)
N3	C13	C14	106.89(19)	N3	C13	C18	124.8(2)
C14	C13	C18	128.1(2)	N4	C14	C13	106.67(18)
N4	C14	C19	124.7(2)	C13	C14	C19	128.3(2)
N3	C15	C16	113.44(19)	N3	C15	C17	111.98(19)
C16	C15	C17	110.85(19)	N4	C20	C21	113.11(18)
N4	C20	C22	111.38(18)	C21	C20	C22	111.60(19)
Si1	C23	C24	120.41(17)	Si1	C23	C28	122.49(17)
C24	C23	C28	117.0(2)	C23	C24	C25	121.8(2)
C24	C25	C26	119.8(2)	C25	C26	C27	119.8(2)
C26	C27	C28	120.2(2)	C23	C28	C27	121.4(2)
Si1	C29	C30	121.81(17)	Si1	C29	C34	121.77(17)
C30	C29	C34	116.4(2)	C29	C30	C31	122.0(2)
C30	C31	C32	119.9(2)	C31	C32	C33	119.7(2)
C32	C33	C34	119.9(2)	C29	C34	C33	122.1(2)
Si2	C35	C36	119.16(15)	Si2	C35	C40	123.59(16)
C36	C35	C40	117.21(19)	C35	C36	C37	121.6(2)
C36	C37	C38	120.0(2)	C37	C38	C39	119.7(2)
C38	C39	C40	120.1(2)	C35	C40	C39	121.3(2)
Si2	C41	C42	116.48(14)	Si2	C41	C46	126.02(17)
C42	C41	C46	117.31(19)	C41	C42	C43	121.96(19)
C42	C43	C44	119.4(2)	C43	C44	C45	120.1(2)

C44	C45	C46	120.5(2)	C41	C46	C45	120.7(2)
Si3	C47	C48	123.65(15)	Si3	C47	C52	119.35(16)
C48	C47	C52	116.94(19)	C47	C48	C49	121.2(2)
C48	C49	C50	120.4(2)	C49	C50	C51	119.4(2)
C50	C51	C52	119.9(2)	C47	C52	C51	122.1(2)
Si3	C53	C54	123.97(16)	Si3	C53	C58	119.35(16)
C54	C53	C58	116.43(19)	C53	C54	C55	121.6(2)
C54	C55	C56	120.3(2)	C55	C56	C57	119.5(2)
C56	C57	C58	120.1(2)	C53	C58	C57	122.0(2)
Si4	C59	C60	118.25(15)	Si4	C59	C64	124.34(16)
C60	C59	C64	117.09(19)	C59	C60	C61	122.1(2)
C60	C61	C62	119.3(2)	C61	C62	C63	120.1(2)
C62	C63	C64	120.2(2)	C59	C64	C63	121.2(2)
Si4	C65	C66	121.28(15)	Si4	C65	C70	121.11(15)
C66	C65	C70	117.56(18)	C65	C66	C67	121.4(2)
C66	C67	C68	120.0(2)	C67	C68	C69	119.71(19)
C68	C69	C70	119.9(2)	C65	C70	C69	121.3(2)
C72	C71	C76	119.1(3)	C72	C71	C77	120.8(3)
C76	C71	C77	120.1(3)	C71	C72	C73	121.0(3)
C72	C73	C74	120.7(3)	C73	C74	C75	119.5(3)
C74	C75	C76	119.2(3)	C71	C76	C75	120.6(3)
C79	C78	C83	118.2(2)	C79	C78	C84	121.4(2)
C83	C78	C84	120.4(2)	C78	C79	C80	121.2(2)
C79	C80	C81	120.1(2)	C80	C81	C82	119.0(3)
C81	C82	C83	120.9(3)	C78	C83	C82	120.6(2)

Torsion Angles(^{o})

(Those having bond angles > 160 or < 20 degrees are excluded.)

atom1	atom2	atom3	atom4	angle	atom1	atom2	atom3	atom4	angle
Pd2	Pd1	Si1	C23	-107.67(3)	Pd2	Pd1	Si1	C29	133.27(3)
Si1	Pd1	Pd2	Si2	-6.73(3)	Si1	Pd1	Pd2	Si3	150.51(3)
Si1	Pd1	Pd2	Si4	159.99(3)	Si1	Pd1	Pd2	C12	-40.64(4)
Pd2	Pd1	Si2	Pd2	0.000(12)	Pd2	Pd1	Si2	C35	114.81(4)
Pd2	Pd1	Si2	C41	-100.77(3)	Si2	Pd1	Pd2	Si2	0.000(15)
Si2	Pd1	Pd2	Si3	157.24(3)	Si2	Pd1	Pd2	Si4	166.71(3)
Si2	Pd1	Pd2	C12	-33.91(3)	Pd2	Pd1	Si4	Pd2	0.000(13)
Pd2	Pd1	Si4	C59	117.82(3)	Pd2	Pd1	Si4	C65	-116.90(3)
Si4	Pd1	Pd2	Si2	-166.71(3)	Si4	Pd1	Pd2	Si3	-9.47(3)
Si4	Pd1	Pd2	Si4	-0.000(18)	Si4	Pd1	Pd2	C12	159.37(3)

Pd2	Pd1	C1	N1	116.45(15)	Pd2	Pd1	C1	N2	-69.93(15)
C1	Pd1	Pd2	Si2	159.47(8)	C1	Pd1	Pd2	Si3	-43.29(9)
C1	Pd1	Pd2	Si4	-33.82(8)	C1	Pd1	Pd2	C12	125.56(8)
Si1	Pd1	Si2	Pd2	174.54(2)	Si1	Pd1	Si2	C35	-70.66(3)
Si1	Pd1	Si2	C41	73.77(3)	Si2	Pd1	Si1	C23	-113.39(3)
Si2	Pd1	Si1	C29	127.56(3)	Si1	Pd1	C1	N1	-74.37(17)
Si1	Pd1	C1	N2	99.25(13)	C1	Pd1	Si1	C23	82.35(6)
C1	Pd1	Si1	C29	-36.71(6)	Si2	Pd1	Si4	Pd2	-11.21(2)
Si2	Pd1	Si4	C59	106.61(3)	Si2	Pd1	Si4	C65	-128.11(3)
Si4	Pd1	Si2	Pd2	10.69(2)	Si4	Pd1	Si2	C35	125.49(3)
Si4	Pd1	Si2	C41	-90.08(4)	Si4	Pd1	C1	N1	90.72(17)
Si4	Pd1	C1	N2	-95.66(13)	C1	Pd1	Si4	Pd2	156.21(6)
C1	Pd1	Si4	C59	-85.96(6)	C1	Pd1	Si4	C65	39.32(6)
Pd1	Pd2	Si2	Pd1	0.000(13)	Pd1	Pd2	Si2	C35	-116.54(4)
Pd1	Pd2	Si2	C41	117.19(3)	Pd1	Pd2	Si3	C47	-104.38(3)
Pd1	Pd2	Si3	C53	133.91(3)	Pd1	Pd2	Si4	Pd1	-0.000(12)
Pd1	Pd2	Si4	C59	-102.62(4)	Pd1	Pd2	Si4	C65	116.10(4)
Pd1	Pd2	C12	N3	-69.25(14)	Pd1	Pd2	C12	N4	108.45(17)
Si2	Pd2	Si4	Pd1	10.70(2)	Si2	Pd2	Si4	C59	-91.92(4)
Si2	Pd2	Si4	C65	126.80(3)	Si4	Pd2	Si2	Pd1	-11.23(2)
Si4	Pd2	Si2	C35	-127.77(3)	Si4	Pd2	Si2	C41	105.96(3)
Si2	Pd2	C12	N3	-95.25(13)	Si2	Pd2	C12	N4	82.46(17)
C12	Pd2	Si2	Pd1	153.87(6)	C12	Pd2	Si2	C35	37.33(6)
C12	Pd2	Si2	C41	-88.94(6)	Si3	Pd2	Si4	Pd1	172.69(2)
Si3	Pd2	Si4	C59	70.07(3)	Si3	Pd2	Si4	C65	-71.21(3)
Si4	Pd2	Si3	C47	-112.33(3)	Si4	Pd2	Si3	C53	125.96(3)
Si3	Pd2	C12	N3	102.25(12)	Si3	Pd2	C12	N4	-80.05(17)
C12	Pd2	Si3	C47	84.47(6)	C12	Pd2	Si3	C53	-37.24(6)
Pd1	Si1	C23	C24	45.16(15)	Pd1	Si1	C23	C28	-138.49(12)
Pd1	Si1	C29	C30	-112.70(13)	Pd1	Si1	C29	C34	64.29(16)
C23	Si1	C29	C30	127.94(15)	C23	Si1	C29	C34	-55.07(17)
C29	Si1	C23	C24	167.11(13)	C29	Si1	C23	C28	-16.54(17)
Pd1	Si2	C35	C36	8.99(16)	Pd1	Si2	C35	C40	-168.53(10)
Pd1	Si2	C41	C42	52.69(15)	Pd1	Si2	C41	C46	-122.25(14)
Pd2	Si2	C35	C36	96.84(13)	Pd2	Si2	C35	C40	-80.68(14)
Pd2	Si2	C41	C42	-28.30(15)	Pd2	Si2	C41	C46	156.76(13)
C35	Si2	C41	C42	-159.60(13)	C35	Si2	C41	C46	25.46(19)
C41	Si2	C35	C36	-138.50(13)	C41	Si2	C35	C40	43.98(17)
Pd2	Si3	C47	C48	-129.92(12)	Pd2	Si3	C47	C52	53.00(14)

Pd2	Si3	C53	C54	52.98(16)	Pd2	Si3	C53	C58	-121.06(12)
C47	Si3	C53	C54	-67.77(16)	C47	Si3	C53	C58	118.18(14)
C53	Si3	C47	C48	-5.74(17)	C53	Si3	C47	C52	177.18(12)
Pd1	Si4	C59	C60	-24.75(15)	Pd1	Si4	C59	C64	162.00(12)
Pd1	Si4	C65	C66	-76.38(15)	Pd1	Si4	C65	C70	100.98(13)
Pd2	Si4	C59	C60	57.37(16)	Pd2	Si4	C59	C64	-115.88(13)
Pd2	Si4	C65	C66	-165.07(10)	Pd2	Si4	C65	C70	12.30(17)
C59	Si4	C65	C66	49.28(16)	C59	Si4	C65	C70	-133.36(14)
C65	Si4	C59	C60	-156.94(13)	C65	Si4	C59	C64	29.80(18)
C1	N1	C2	C3	-0.1(2)	C1	N1	C2	C7	-179.49(16)
C2	N1	C1	Pd1	174.41(16)	C2	N1	C1	N2	0.1(2)
C1	N1	C4	C5	122.58(18)	C1	N1	C4	C6	-110.97(19)
C4	N1	C1	Pd1	-7.4(3)	C4	N1	C1	N2	178.34(15)
C2	N1	C4	C5	-59.5(2)	C2	N1	C4	C6	67.0(2)
C4	N1	C2	C3	-178.23(16)	C4	N1	C2	C7	2.4(3)
C1	N2	C3	C2	-0.0(2)	C1	N2	C3	C8	-178.26(16)
C3	N2	C1	Pd1	-175.36(13)	C3	N2	C1	N1	-0.1(2)
C1	N2	C9	C10	-109.15(19)	C1	N2	C9	C11	123.92(18)
C9	N2	C1	Pd1	2.4(2)	C9	N2	C1	N1	177.75(15)
C3	N2	C9	C10	68.3(2)	C3	N2	C9	C11	-58.7(3)
C9	N2	C3	C2	-177.67(16)	C9	N2	C3	C8	4.1(3)
C12	N3	C13	C14	-0.1(2)	C12	N3	C13	C18	-175.91(16)
C13	N3	C12	Pd2	178.04(14)	C13	N3	C12	N4	-0.3(2)
C12	N3	C15	C16	131.75(18)	C12	N3	C15	C17	-101.9(2)
C15	N3	C12	Pd2	-2.2(2)	C15	N3	C12	N4	179.51(16)
C13	N3	C15	C16	-48.5(3)	C13	N3	C15	C17	77.9(2)
C15	N3	C13	C14	-179.88(17)	C15	N3	C13	C18	4.3(3)
C12	N4	C14	C13	-0.6(2)	C12	N4	C14	C19	173.79(16)
C14	N4	C12	Pd2	-177.37(16)	C14	N4	C12	N3	0.6(2)
C12	N4	C20	C21	-124.13(18)	C12	N4	C20	C22	109.23(19)
C20	N4	C12	Pd2	6.4(3)	C20	N4	C12	N3	-175.66(15)
C14	N4	C20	C21	60.3(2)	C14	N4	C20	C22	-66.4(2)
C20	N4	C14	C13	175.40(16)	C20	N4	C14	C19	-10.2(3)
N1	C2	C3	N2	0.1(2)	N1	C2	C3	C8	178.26(17)
C7	C2	C3	N2	179.4(2)	C7	C2	C3	C8	-2.4(4)
N3	C13	C14	N4	0.4(2)	N3	C13	C14	C19	-173.72(17)
C18	C13	C14	N4	176.1(2)	C18	C13	C14	C19	1.9(4)
Si1	C23	C24	C25	177.95(13)	Si1	C23	C28	C27	-177.58(13)
C24	C23	C28	C27	-1.1(3)	C28	C23	C24	C25	1.4(3)

C23	C24	C25	C26	-0.6(3)	C24	C25	C26	C27	-0.6(4)
C25	C26	C27	C28	0.9(4)	C26	C27	C28	C23	0.0(3)
Si1	C29	C30	C31	175.20(14)	Si1	C29	C34	C33	-177.63(14)
C30	C29	C34	C33	-0.5(3)	C34	C29	C30	C31	-1.9(3)
C29	C30	C31	C32	2.8(4)	C30	C31	C32	C33	-1.2(4)
C31	C32	C33	C34	-1.2(4)	C32	C33	C34	C29	2.1(4)
Si2	C35	C36	C37	-178.44(13)	Si2	C35	C40	C39	176.84(13)
C36	C35	C40	C39	-0.7(3)	C40	C35	C36	C37	-0.8(3)
C35	C36	C37	C38	1.4(3)	C36	C37	C38	C39	-0.6(3)
C37	C38	C39	C40	-0.9(3)	C38	C39	C40	C35	1.6(3)
Si2	C41	C42	C43	-174.71(14)	Si2	C41	C46	C45	174.92(13)
C42	C41	C46	C45	0.0(3)	C46	C41	C42	C43	0.7(3)
C41	C42	C43	C44	-0.2(3)	C42	C43	C44	C45	-1.0(4)
C43	C44	C45	C46	1.7(4)	C44	C45	C46	C41	-1.2(3)
Si3	C47	C48	C49	-177.15(12)	Si3	C47	C52	C51	177.04(12)
C48	C47	C52	C51	-0.2(3)	C52	C47	C48	C49	-0.0(3)
C47	C48	C49	C50	0.5(3)	C48	C49	C50	C51	-0.8(3)
C49	C50	C51	C52	0.6(3)	C50	C51	C52	C47	-0.0(3)
Si3	C53	C54	C55	-173.73(13)	Si3	C53	C58	C57	172.85(13)
C54	C53	C58	C57	-1.6(3)	C58	C53	C54	C55	0.5(3)
C53	C54	C55	C56	0.9(3)	C54	C55	C56	C57	-1.2(3)
C55	C56	C57	C58	0.0(3)	C56	C57	C58	C53	1.4(3)
Si4	C59	C60	C61	-174.03(13)	Si4	C59	C64	C63	172.87(13)
C60	C59	C64	C63	-0.5(3)	C64	C59	C60	C61	-0.3(3)
C59	C60	C61	C62	0.8(3)	C60	C61	C62	C63	-0.6(3)
C61	C62	C63	C64	-0.1(3)	C62	C63	C64	C59	0.6(3)
Si4	C65	C66	C67	177.33(13)	Si4	C65	C70	C69	-178.02(12)
C66	C65	C70	C69	-0.6(3)	C70	C65	C66	C67	-0.1(3)
C65	C66	C67	C68	0.8(3)	C66	C67	C68	C69	-0.8(3)
C67	C68	C69	C70	0.1(3)	C68	C69	C70	C65	0.6(3)
C72	C71	C76	C75	-0.1(4)	C76	C71	C72	C73	0.5(4)
C77	C71	C72	C73	179.7(3)	C77	C71	C76	C75	-179.4(3)
C71	C72	C73	C74	-0.1(5)	C72	C73	C74	C75	-0.6(5)
C73	C74	C75	C76	0.9(4)	C74	C75	C76	C71	-0.5(4)
C79	C78	C83	C82	0.6(3)	C83	C78	C79	C80	0.2(3)
C84	C78	C79	C80	178.4(2)	C84	C78	C83	C82	-177.7(2)
C78	C79	C80	C81	-0.7(4)	C79	C80	C81	C82	0.5(4)
C80	C81	C82	C83	0.3(4)	C81	C82	C83	C78	-0.8(4)

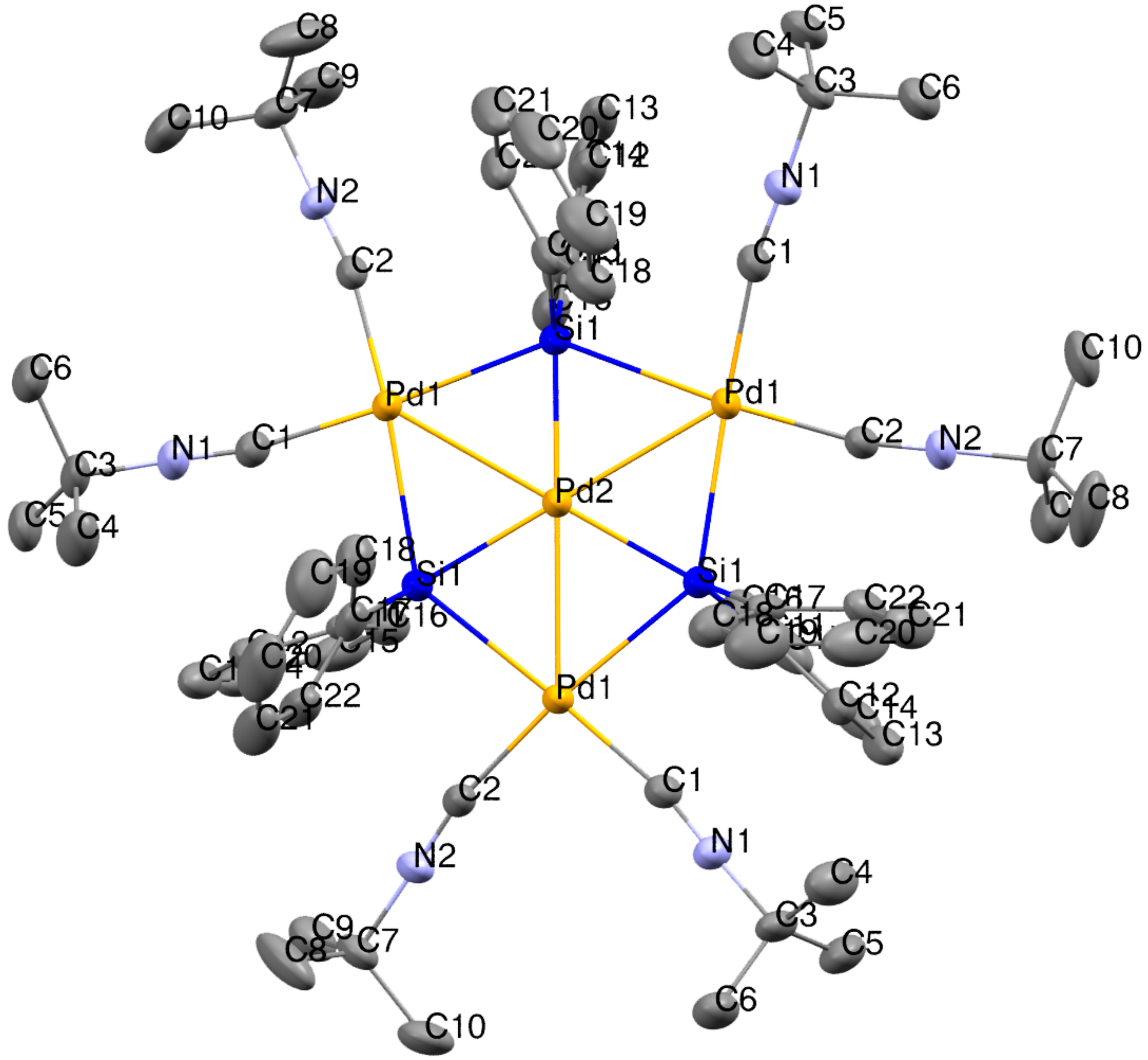

Supplementary Fig. 49. ORTEP drawing of **6** (50% probability of the thermal ellipsoids). Hydrogen atoms were omitted for clarity.

Supplementary Table 14. Crystal data and structure refinement for **6**.

Empirical Formula	$C_{66}H_{84}N_6Pd_4Si_3$
Formula Weight	1471.29
Crystal Color, Habit	yellow, block
Crystal Dimensions	0.100 X 0.080 X 0.060 mm
Crystal System	trigonal
Lattice Type	Primitive
Lattice Parameters	a = 20.19000 Å
	c = 26.16590 Å
	V = 9237.16992 Å^3
Space Group	P-3c1 (#165)
Z value	4
D_{calc}	1.058 g/cm^3
F_{000}	2992.00
μ(MoKα)	8.363 cm^{-1}
Diffractometer	Saturn724
Radiation	MoKα (λ = 0.71075 Å)
	graphite monochromated
Voltage, Current	50kV, 24mA
Temperature	-150.0^{o}C
Detector Aperture	72.8 x 72.8 mm
Data Images	720 exposures
ω oscillation Range (χ=45.0, ϕ=0.0)	-70.0 - 110.0^o
Exposure Rate	10.0 sec./^{o}
Detector Swing Angle	20.00^o
ω oscillation Range (χ=45.0, ϕ=90.0)	-70.0 - 110.0^o
Exposure Rate	10.0 sec./^{o}
Detector Swing Angle	20.00^o
Detector Position	44.61 mm
Pixel Size	0.035 mm
$2\theta_{max}$	62.4^o
No. of Reflections Measured	Total: 83711
	Unique: 9541 (R_{int} = 0.0706)
Corrections	Lorentz-polarization
	Absorption
	(trans. factors: 0.819 - 0.951)
Structure Solution	Direct Methods (SHELXT Version 2014/5)
Refinement	Full-matrix least-squares on F^2

Function Minimized	Σ w $(Fo^2 - Fc^2)^2$
Least Squares Weights	w = 1/ [$\sigma^2(Fo^2)$ + $(0.0434 \cdot P)^2$ + 0.6768 · P] where P = $(Max(Fo^2,0) + 2Fc^2)/3$
$2\theta_{max}$ cutoff	62.4°
Anomalous Dispersion	All non-hydrogen atoms
No. Observations (All reflections)	9541
No. Variables	244
Reflection/Parameter Ratio	39.10
Residuals: R1 (I>2.00σ(I))	0.0428
Residuals: R (All reflections)	0.0771
Residuals: wR2 (All reflections)	0.0891
Goodness of Fit Indicator	1.012
Max Shift/Error in Final Cycle	0.004
Maximum peak in Final Diff. Map	1.33 $e^-/Å^3$
Minimum peak in Final Diff. Map	-0.49 $e^-/Å^3$

Atomic coordinates and B_{iso}/B_{eq}

atom	x	y	z	B_{eq}	occ
Pd1	0.56462(2)	0.38548(2)	0.60461(2)	2.077(5)	1
Pd2	0.66667	0.33333	0.60664(2)	2.114(6)	1/3
Si1	0.70923(4)	0.46114(4)	0.60747(3)	2.130(11)	1
N1	0.38874(12)	0.31046(12)	0.62878(9)	2.89(4)	1
N2	0.56752(11)	0.53820(12)	0.57544(8)	2.81(4)	1
C1	0.45143(14)	0.33107(14)	0.61794(10)	2.62(4)	1
C2	0.57423(13)	0.48712(13)	0.58732(10)	2.56(4)	1
C3	0.31027(14)	0.28224(16)	0.64419(11)	3.28(5)	1
C4	0.29495(18)	0.22955(19)	0.68961(13)	4.77(7)	1
C5	0.25912(16)	0.23967(18)	0.59899(13)	4.38(6)	1
C6	0.30241(16)	0.35073(17)	0.65896(13)	4.18(6)	1
C7	0.56022(16)	0.60439(14)	0.56217(11)	3.34(5)	1
C8	0.5958(3)	0.66168(19)	0.60513(14)	6.12(9)	1
C9	0.59888(19)	0.63453(17)	0.51132(13)	4.56(7)	1
C10	0.47432(19)	0.57618(19)	0.55809(15)	5.20(8)	1
C11	0.73733(13)	0.52643(13)	0.54978(10)	2.46(4)	1
C12	0.78156(14)	0.60660(14)	0.55138(12)	3.32(5)	1
C13	0.80205(16)	0.64998(18)	0.50709(14)	4.32(7)	1
C14	0.78028(16)	0.6165(2)	0.46058(14)	4.39(7)	1
C15	0.73549(17)	0.5370(2)	0.45725(12)	4.31(7)	1
C16	0.71508(15)	0.49372(16)	0.50146(11)	3.23(5)	1
C17	0.72599(13)	0.52074(14)	0.66777(10)	2.76(4)	1
C18	0.75098(17)	0.50158(16)	0.71200(11)	3.63(5)	1
C19	0.7625(2)	0.5416(2)	0.75734(12)	5.16(8)	1
C20	0.74910(19)	0.6017(2)	0.75928(15)	5.57(9)	1
C21	0.72383(18)	0.62181(19)	0.71639(14)	4.91(7)	1
C22	0.71252(15)	0.58236(16)	0.67169(12)	3.63(5)	1

$B_{eq} = 8/3\ \pi^2(U_{11}(aa^*)^2 + U_{22}(bb^*)^2 + U_{33}(cc^*)^2 + 2U_{12}(aa^*bb^*)\cos\gamma + 2U_{13}(aa^*cc^*)\cos\beta + 2U_{23}(bb^*cc^*)\cos\alpha)$

Anisotropic displacement parameters

atom	U_{11}	U_{22}	U_{33}	U_{12}	U_{13}	U_{23}
Pd1	0.02058(9)	0.02111(9)	0.03883(11)	0.01162(7)	0.00051(7)	0.00027(7)
Pd2	0.01879(9)	0.01879(9)	0.04273(18)	0.00940(5)	0.00000	0.00000
Si1	0.0217(3)	0.0206(3)	0.0390(4)	0.0108(3)	0.0008(3)	-0.0016(3)
N1	0.0256(11)	0.0346(11)	0.0520(13)	0.0167(9)	0.0026(10)	0.0055(10)
N2	0.0318(11)	0.0284(11)	0.0527(13)	0.0195(9)	-0.0044(10)	-0.0031(10)

C1	0.0297(13)	0.0300(13)	0.0435(14)	0.0176(11)	-0.0009(11)	0.0006(11)
C2	0.0223(12)	0.0259(12)	0.0490(15)	0.0120(10)	-0.0005(10)	-0.0038(11)
C3	0.0248(13)	0.0411(15)	0.0644(18)	0.0206(12)	0.0085(12)	0.0116(13)
C4	0.0460(18)	0.065(2)	0.076(2)	0.0324(17)	0.0192(16)	0.0278(18)
C5	0.0284(14)	0.0536(19)	0.087(2)	0.0221(14)	-0.0055(15)	-0.0017(17)
C6	0.0355(15)	0.0485(18)	0.082(2)	0.0263(14)	0.0061(15)	0.0019(16)
C7	0.0464(16)	0.0276(13)	0.0617(18)	0.0249(12)	-0.0083(14)	-0.0024(12)
C8	0.124(3)	0.0444(19)	0.083(3)	0.056(2)	-0.043(2)	-0.0206(17)
C9	0.061(2)	0.0393(17)	0.080(2)	0.0303(16)	0.0028(17)	0.0118(15)
C10	0.062(2)	0.057(2)	0.103(3)	0.0478(18)	0.0003(19)	0.0045(19)
C11	0.0226(11)	0.0304(12)	0.0465(14)	0.0179(11)	0.0033(10)	0.0057(11)
C12	0.0299(13)	0.0309(13)	0.0685(19)	0.0176(12)	0.0039(13)	0.0087(13)
C13	0.0333(15)	0.0426(16)	0.091(3)	0.0212(14)	0.0124(16)	0.0280(17)
C14	0.0359(16)	0.068(2)	0.074(2)	0.0338(16)	0.0161(15)	0.0373(18)
C15	0.0449(18)	0.090(3)	0.0461(17)	0.0465(19)	0.0056(14)	0.0106(17)
C16	0.0362(14)	0.0431(15)	0.0492(16)	0.0242(13)	0.0056(12)	0.0066(13)
C17	0.0209(12)	0.0293(13)	0.0476(15)	0.0074(10)	0.0045(11)	-0.0069(11)
C18	0.0444(16)	0.0382(15)	0.0445(16)	0.0124(13)	0.0032(13)	-0.0014(12)
C19	0.064(2)	0.067(2)	0.0410(17)	0.0153(19)	0.0030(16)	-0.0072(16)
C20	0.052(2)	0.068(2)	0.066(2)	0.0103(18)	0.0144(18)	-0.0328(19)
C21	0.0438(18)	0.054(2)	0.085(3)	0.0217(16)	0.0043(17)	-0.0333(19)
C22	0.0305(14)	0.0436(16)	0.0655(19)	0.0197(13)	-0.0009(13)	-0.0177(14)

The general temperature factor expression: $\exp(-2\pi^2(a^{*2}U_{11}h^2 + b^{*2}U_{22}k^2 + c^{*2}U_{33}l^2 + 2a^*b^*U_{12}hk + 2a^*c^*U_{13}hl + 2b^*c^*U_{23}kl))$

Bond lengths (Å)

atom	atom	distance	atom	atom	distance
Pd1	Pd2	2.7433(5)	Pd1	Si1	2.5305(8)
Pd1	Si1[1]	2.5550(10)	Pd1	C1	2.010(3)
Pd1	C2	2.014(3)	Pd2	Si1	2.2760(8)
Pd2	Si1[1]	2.2760(8)	Pd2	Si1[2]	2.2760(8)
Si1	C11	1.895(3)	Si1	C17	1.909(3)
N1	C1	1.153(4)	N1	C3	1.447(4)
N2	C2	1.148(4)	N2	C7	1.458(4)
C3	C4	1.520(5)	C3	C5	1.522(4)
C3	C6	1.519(5)	C7	C8	1.512(4)
C7	C9	1.508(4)	C7	C10	1.535(5)
C11	C12	1.405(3)	C11	C16	1.393(4)
C12	C13	1.385(5)	C13	C14	1.354(5)

C14	C15	1.397(5)	C15	C16	1.383(4)
C17	C18	1.392(4)	C17	C22	1.404(5)
C18	C19	1.388(5)	C19	C20	1.371(7)
C20	C21	1.375(6)	C21	C22	1.368(5)

Symmetry Operators:

(1) -Y+1,X-Y,Z (2) -X+Y+1,-X+1,Z

Bond angles (^o)

atom	atom	atom	angle	atom	atom	atom	angle
Pd2	Pd1	Si1	50.94(2)	Pd2	Pd1	$Si1^1$	50.728(17)
Pd2	Pd1	C1	130.82(9)	Pd2	Pd1	C2	133.41(7)
Si1	Pd1	$Si1^1$	101.63(3)	Si1	Pd1	C1	167.98(8)
Si1	Pd1	C2	84.10(7)	$Si1^1$	Pd1	C1	80.96(9)
$Si1^1$	Pd1	C2	167.56(8)	C1	Pd1	C2	95.73(12)
Pd1	Pd2	$Pd1^1$	119.963(9)	Pd1	Pd2	$Pd1^2$	119.963(9)
Pd1	Pd2	Si1	59.69(2)	Pd1	Pd2	$Si1^1$	60.35(2)
Pd1	Pd2	$Si1^2$	179.35(3)	$Pd1^1$	Pd2	$Pd1^2$	119.963(9)
$Pd1^1$	Pd2	Si1	179.35(3)	$Pd1^1$	Pd2	$Si1^1$	59.69(2)
$Pd1^1$	Pd2	$Si1^2$	60.35(2)	$Pd1^2$	Pd2	Si1	60.35(2)
$Pd1^2$	Pd2	$Si1^1$	179.35(3)	$Pd1^2$	Pd2	$Si1^2$	59.69(2)
Si1	Pd2	$Si1^1$	119.99(3)	Si1	Pd2	$Si1^2$	119.99(3)
$Si1^1$	Pd2	$Si1^2$	119.99(3)	Pd1	Si1	$Pd1^2$	138.18(4)
Pd1	Si1	Pd2	69.372(19)	Pd1	Si1	C11	104.49(8)
Pd1	Si1	C17	101.09(8)	$Pd1^2$	Si1	Pd2	68.92(3)
$Pd1^2$	Si1	C11	97.50(10)	$Pd1^2$	Si1	C17	104.84(10)
Pd2	Si1	C11	126.38(9)	Pd2	Si1	C17	124.75(9)
C11	Si1	C17	108.84(12)	C1	N1	C3	177.3(3)
C2	N2	C7	177.9(2)	Pd1	C1	N1	169.4(3)
Pd1	C2	N2	169.00(19)	N1	C3	C4	107.0(3)
N1	C3	C5	107.8(2)	N1	C3	C6	107.7(2)
C4	C3	C5	111.7(2)	C4	C3	C6	111.2(3)
C5	C3	C6	111.2(3)	N2	C7	C8	107.3(3)
N2	C7	C9	108.1(3)	N2	C7	C10	106.8(2)
C8	C7	C9	113.3(2)	C8	C7	C10	110.6(3)
C9	C7	C10	110.4(3)	Si1	C11	C12	125.1(2)
Si1	C11	C16	118.68(18)	C12	C11	C16	116.2(2)
C11	C12	C13	121.4(3)	C12	C13	C14	121.1(3)
C13	C14	C15	119.5(3)	C14	C15	C16	119.4(3)
C11	C16	C15	122.4(3)	Si1	C17	C18	119.1(2)

Si1	C17	C22	124.6(2)	C18	C17	C22	116.3(3)
C17	C18	C19	121.8(4)	C18	C19	C20	119.8(4)
C19	C20	C21	119.8(4)	C20	C21	C22	120.3(4)
C17	C22	C21	121.9(3)				

Symmetry Operators:

(1) -Y+1,X-Y,Z (2) -X+Y+1,-X+1,Z

Torsion Angles(^{o})

(Those having bond angles > 160 or < 20 degrees are excluded.)

atom1	atom2	atom3	atom4	angle	atom1	atom2	atom3	atom4	angle
Pd2	Pd1	Si1	$Pd1^1$	4.47(4)	Pd2	Pd1	Si1	Pd2	-0.000(14)
Pd2	Pd1	Si1	C11	123.87(4)	Pd2	Pd1	Si1	C17	-123.15(4)
Si1	Pd1	Pd2	$Pd1^2$	-179.36(4)	Si1	Pd1	Pd2	$Pd1^1$	-3.20(3)
Si1	Pd1	Pd2	Si1	0.00(2)	Si1	Pd1	Pd2	$Si1^2$	177.46(3)
Pd2	Pd1	$Si1^2$	$Pd1^2$	-4.48(4)	Pd2	Pd1	$Si1^2$	Pd2	0.000(14)
Pd2	Pd1	$Si1^2$	$C11^2$	-126.19(4)	Pd2	Pd1	$Si1^2$	$C17^2$	121.99(4)
$Si1^2$	Pd1	Pd2	$Pd1^2$	3.18(3)	$Si1^2$	Pd1	Pd2	$Pd1^1$	179.34(4)
$Si1^2$	Pd1	Pd2	Si1	-177.46(3)	$Si1^2$	Pd1	Pd2	$Si1^2$	0.00(2)
C1	Pd1	Pd2	$Pd1^2$	16.17(11)	C1	Pd1	Pd2	$Pd1^1$	-167.68(10)
C1	Pd1	Pd2	Si1	-164.47(10)	C1	Pd1	Pd2	$Si1^2$	12.98(10)
C2	Pd1	Pd2	$Pd1^2$	-161.16(10)	C2	Pd1	Pd2	$Pd1^1$	15.00(11)
C2	Pd1	Pd2	Si1	18.20(11)	C2	Pd1	Pd2	$Si1^2$	-164.34(11)
Si1	Pd1	$Si1^2$	$Pd1^2$	-6.50(6)	Si1	Pd1	$Si1^2$	Pd2	-2.02(3)
Si1	Pd1	$Si1^2$	$C11^2$	-128.20(3)	Si1	Pd1	$Si1^2$	$C17^2$	119.97(3)
$Si1^2$	Pd1	Si1	$Pd1^1$	6.48(6)	$Si1^2$	Pd1	Si1	Pd2	2.01(3)
$Si1^2$	Pd1	Si1	C11	125.88(4)	$Si1^2$	Pd1	Si1	C17	-121.14(4)
C2	Pd1	Si1	$Pd1^1$	-162.35(9)	C2	Pd1	Si1	Pd2	-166.82(8)
C2	Pd1	Si1	C11	-42.94(8)	C2	Pd1	Si1	C17	70.04(8)
C1	Pd1	$Si1^2$	$Pd1^2$	-174.57(9)	C1	Pd1	$Si1^2$	Pd2	-170.09(8)
C1	Pd1	$Si1^2$	$C11^2$	63.73(8)	C1	Pd1	$Si1^2$	$C17^2$	-48.10(8)
Pd1	Pd2	Si1	Pd1	-0.000(13)	Pd1	Pd2	Si1	$Pd1^1$	-176.81(3)
Pd1	Pd2	Si1	C11	-93.13(5)	Pd1	Pd2	Si1	C17	89.37(5)
Pd1	Pd2	$Si1^2$	$Pd1^2$	176.81(3)	Pd1	Pd2	$Si1^2$	Pd1	0.000(13)
Pd1	Pd2	$Si1^2$	$C11^2$	83.67(5)	Pd1	Pd2	$Si1^2$	$C17^2$	-93.83(5)
$Pd1^2$	Pd2	$Si1^2$	$Pd1^2$	0.000(13)	$Pd1^2$	Pd2	$Si1^2$	Pd1	-176.81(3)
$Pd1^2$	Pd2	$Si1^2$	$C11^2$	-93.13(5)	$Pd1^2$	Pd2	$Si1^2$	$C17^2$	89.37(5)
$Pd1^2$	Pd2	$Si1^1$	$Pd1^1$	176.81(3)	$Pd1^2$	Pd2	$Si1^1$	$Pd1^2$	0.000(13)
$Pd1^2$	Pd2	$Si1^1$	$C11^1$	83.67(5)	$Pd1^2$	Pd2	$Si1^1$	$C17^1$	-93.82(5)
$Pd1^1$	Pd2	Si1	Pd1	176.81(3)	$Pd1^1$	Pd2	Si1	$Pd1^1$	0.000(13)

Pd1^{1}	Pd2	Si1	C11	83.67(5)	Pd1^{1}	Pd2	Si1	C17	-93.82(5)
Pd1^{1}	Pd2	Si1^{1}	Pd1^{1}	-0.000(13)	Pd1^{1}	Pd2	Si1^{1}	Pd1^{2}	-176.81(3)
Pd1^{1}	Pd2	Si1^{1}	C11^{1}	-93.13(5)	Pd1^{1}	Pd2	Si1^{1}	C17^{1}	89.37(5)
Si1	Pd2	Si1^{2}	Pd1^{2}	179.34(4)	Si1	Pd2	Si1^{2}	Pd1	2.53(4)
Si1	Pd2	Si1^{2}	C11^{2}	86.21(6)	Si1	Pd2	Si1^{2}	C17^{2}	-91.29(6)
Si1^{2}	Pd2	Si1	Pd1	-2.55(4)	Si1^{2}	Pd2	Si1	Pd1^{1}	-179.36(4)
Si1^{2}	Pd2	Si1	C11	-95.69(6)	Si1^{2}	Pd2	Si1	C17	86.82(6)
Si1	Pd2	Si1^{1}	Pd1^{1}	-2.55(4)	Si1	Pd2	Si1^{1}	Pd1^{2}	-179.36(4)
Si1	Pd2	Si1^{1}	C11^{1}	-95.69(6)	Si1	Pd2	Si1^{1}	C17^{1}	86.82(6)
Si1^{1}	Pd2	Si1	Pd1	179.34(4)	Si1^{1}	Pd2	Si1	Pd1^{1}	2.53(4)
Si1^{1}	Pd2	Si1	C11	86.21(6)	Si1^{1}	Pd2	Si1	C17	-91.29(6)
Si1^{2}	Pd2	Si1^{1}	Pd1^{1}	179.34(4)	Si1^{2}	Pd2	Si1^{1}	Pd1^{2}	2.53(4)
Si1^{2}	Pd2	Si1^{1}	C11^{1}	86.21(6)	Si1^{2}	Pd2	Si1^{1}	C17^{1}	-91.29(6)
Si1^{1}	Pd2	Si1^{2}	Pd1^{2}	-2.55(4)	Si1^{1}	Pd2	Si1^{2}	Pd1	-179.36(4)
Si1^{1}	Pd2	Si1^{2}	C11^{2}	-95.69(6)	Si1^{1}	Pd2	Si1^{2}	C17^{2}	86.82(6)
Pd1	Si1	C11	C12	122.8(2)	Pd1	Si1	C11	C16	-59.2(2)
Pd1	Si1	C17	C18	105.72(14)	Pd1	Si1	C17	C22	-72.11(15)
Pd1^{1}	Si1	C11	C12	-93.0(2)	Pd1^{1}	Si1	C11	C16	84.89(19)
Pd1^{1}	Si1	C17	C18	-41.16(15)	Pd1^{1}	Si1	C17	C22	141.01(14)
Pd2	Si1	C11	C12	-162.34(16)	Pd2	Si1	C11	C16	15.6(3)
Pd2	Si1	C17	C18	33.24(19)	Pd2	Si1	C17	C22	-144.60(12)
C11	Si1	C17	C18	-144.64(15)	C11	Si1	C17	C22	37.5(2)
C17	Si1	C11	C12	15.5(3)	C17	Si1	C11	C16	-166.58(18)
Si1	C11	C12	C13	177.45(19)	Si1	C11	C16	C15	-177.6(2)
C12	C11	C16	C15	0.5(5)	C16	C11	C12	C13	-0.5(4)
C11	C12	C13	C14	-0.2(5)	C12	C13	C14	C15	1.0(6)
C13	C14	C15	C16	-1.0(6)	C14	C15	C16	C11	0.2(6)
Si1	C17	C18	C19	-178.52(15)	Si1	C17	C22	C21	178.34(14)
C18	C17	C22	C21	0.5(3)	C22	C17	C18	C19	-0.5(3)
C17	C18	C19	C20	0.1(4)	C18	C19	C20	C21	0.4(4)
C19	C20	C21	C22	-0.4(4)	C20	C21	C22	C17	0.0(4)

Symmetry Operators:

(1) -X+Y+1,-X+1,Z (2) -Y+1,X-Y,Z

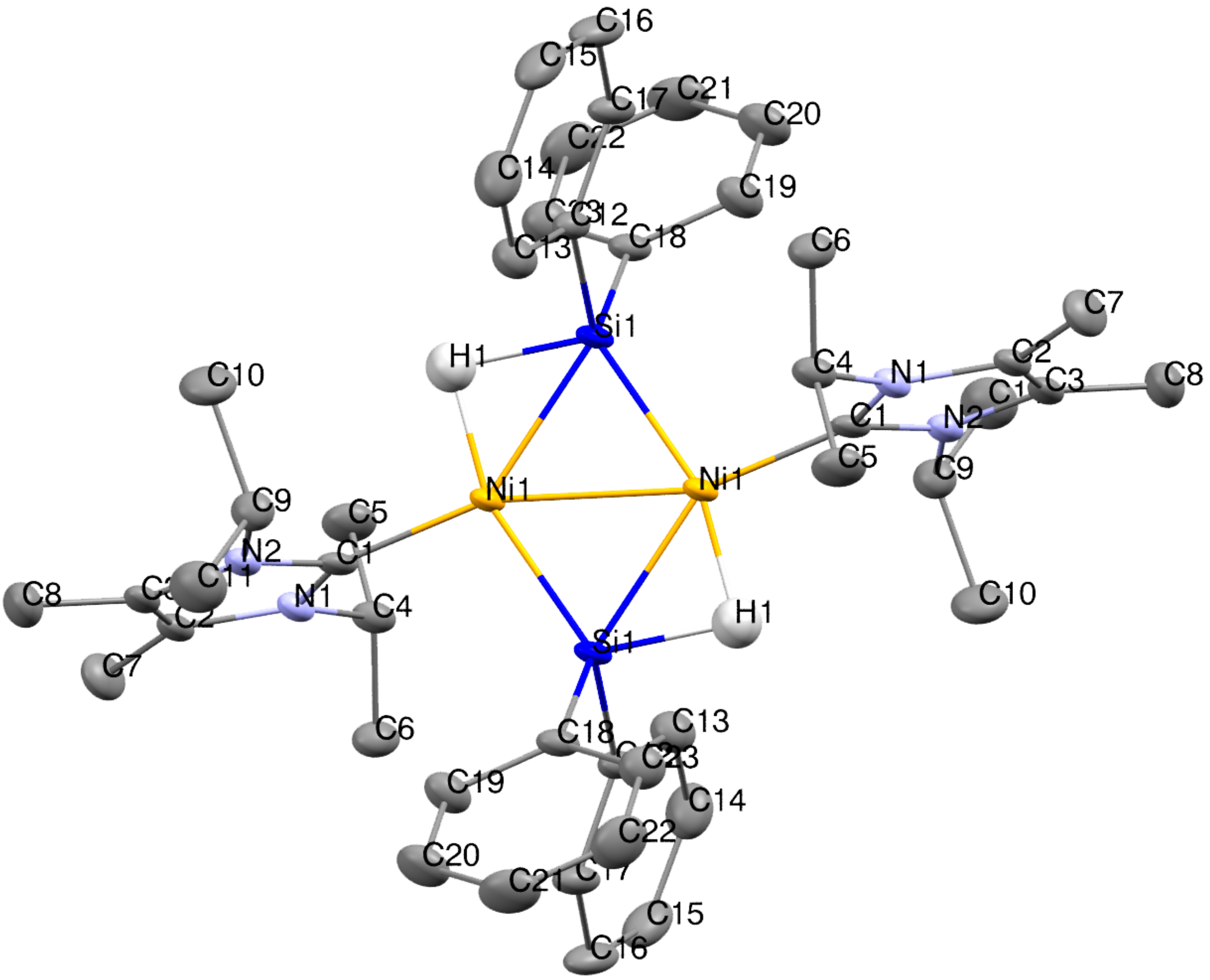

Supplementary Fig. 50. ORTEP drawing of **7** (50% probability of the thermal ellipsoids). Hydrogen atoms were omitted for clarity.

Supplementary Table 15. Crystal data and structure refinement for **7**.

Empirical Formula	$C_{46}H_{62}N_4Ni_2Si_2$
Formula Weight	844.59
Crystal Color, Habit	red, block
Crystal Dimensions	0.150 X 0.100 X 0.100 mm
Crystal System	triclinic
Lattice Type	Primitive
Lattice Parameters	a = 10.346(4) Å
	b = 10.728(4) Å
	c = 11.238(4) Å
	α = 100.255(3) ^{o}
	β = 95.690(4) ^{o}
	γ = 110.268(5) ^{o}
	V = 1133.8(7) $Å^3$
Space Group	P-1 (#2)
Z value	1
D_{calc}	1.237 g/cm^3
F_{000}	450.00
μ(MoKα)	9.180 cm^{-1}
Diffractometer	Saturn724
Radiation	MoKα (λ = 0.71075 Å)
	multi-layer mirror monochromated
Voltage, Current	50kV, 24mA
Temperature	-149.8^{o}C
Detector Aperture	72.8 x 72.8 mm
Data Images	720 exposures
ω oscillation Range (χ=45.0, ϕ=0.0)	-70.0 - 110.0^{o}
Exposure Rate	10.0 sec./^{o}
Detector Swing Angle	19.90^{o}
ω oscillation Range (χ=45.0, ϕ=90.0)	-70.0 - 110.0^{o}
Exposure Rate	10.0 sec./^{o}
Detector Swing Angle	19.90^{o}
Detector Position	44.61 mm
Pixel Size	0.141 mm
$2\theta_{max}$	55.0^{o}
No. of Reflections Measured	Total: 9338
	Unique: 4993 (R_{int} = 0.1208)
Corrections	Lorentz-polarization

	Absorption
	(trans. factors: 0.736 - 0.912)
Structure Solution	Direct Methods (SHELXT Version 2014/5)
Refinement	Full-matrix least-squares on F^2
Function Minimized	Σ w $(Fo^2 - Fc^2)^2$
Least Squares Weights	w = 1/ [$\sigma^2(Fo^2)$ + $(0.0861 \cdot P)^2$
	+ 0.7865 · P]
	where P = (Max(Fo^2,0) + $2Fc^2$)/3
$2\theta_{max}$ cutoff	55.0^o
Anomalous Dispersion	All non-hydrogen atoms
No. Observations (All reflections)	4993
No. Variables	254
Reflection/Parameter Ratio	19.66
Residuals: R1 (I>2.00σ(I))	0.0532
Residuals: R (All reflections)	0.0571
Residuals: wR2 (All reflections)	0.1380
Goodness of Fit Indicator	0.938
Max Shift/Error in Final Cycle	0.001
Maximum peak in Final Diff. Map	1.49 $e^-/Å^3$
Minimum peak in Final Diff. Map	-1.30 $e^-/Å^3$

Atomic coordinates and B_{iso}/B_{eq}

atom	x	y	z	B_{eq}
Ni1	0.38853(3)	0.48788(3)	0.54059(2)	1.137(10)
Si1	0.59397(6)	0.55738(6)	0.65816(5)	1.213(12)
N1	0.19929(19)	0.3873(2)	0.70885(16)	1.36(3)
N2	0.20749(19)	0.5846(2)	0.68580(17)	1.37(3)
C1	0.2652(2)	0.4905(2)	0.65414(18)	1.28(3)
C2	0.0977(2)	0.4159(3)	0.77022(19)	1.57(4)
C3	0.1025(2)	0.5394(3)	0.75593(19)	1.57(4)
C4	0.2314(3)	0.2629(2)	0.6904(2)	1.61(4)
C5	0.1140(3)	0.1434(3)	0.6026(3)	2.44(4)
C6	0.2745(3)	0.2293(3)	0.8100(2)	2.01(4)
C7	0.0086(3)	0.3262(3)	0.8416(3)	2.48(5)
C8	0.0161(3)	0.6168(3)	0.8030(3)	2.50(5)
C9	0.2461(3)	0.7076(3)	0.6347(2)	1.85(4)
C10	0.1364(3)	0.6892(3)	0.5249(3)	2.81(5)
C11	0.2795(3)	0.8393(3)	0.7305(3)	2.98(5)
C12	0.6192(2)	0.4401(2)	0.75864(19)	1.43(3)
C13	0.6066(3)	0.3073(3)	0.7046(2)	2.15(4)
C14	0.6215(3)	0.2173(3)	0.7758(3)	2.74(5)
C15	0.6466(3)	0.2580(3)	0.9030(3)	2.70(5)
C16	0.6588(3)	0.3880(3)	0.9586(2)	2.52(5)
C17	0.6461(2)	0.4784(3)	0.8875(2)	1.83(4)
C18	0.6726(2)	0.7348(2)	0.76238(19)	1.48(3)
C19	0.6010(3)	0.7785(3)	0.8498(2)	2.09(4)
C20	0.6614(3)	0.9044(3)	0.9329(3)	2.48(5)
C21	0.7965(3)	0.9893(3)	0.9309(3)	2.78(5)
C22	0.8693(3)	0.9493(3)	0.8445(3)	2.79(5)
C23	0.8075(3)	0.8246(3)	0.7607(2)	2.05(4)

$B_{eq} = 8/3\ \pi^2(U_{11}(aa^*)^2 + U_{22}(bb^*)^2 + U_{33}(cc^*)^2 + 2U_{12}(aa^*bb^*)\cos\gamma + 2U_{13}(aa^*cc^*)\cos\beta + 2U_{23}(bb^*cc^*)\cos\alpha)$

Anisotropic displacement parameters

atom	U_{11}	U_{22}	U_{33}	U_{12}	U_{13}	U_{23}
Ni1	0.01453(18)	0.01730(18)	0.00721(16)	0.00127(12)	0.00116(10)	0.00238(11)
Si1	0.0166(3)	0.0176(3)	0.0070(3)	0.0011(2)	0.0006(2)	0.0022(2)
N1	0.0173(9)	0.0189(9)	0.0111(8)	0.0014(7)	0.0031(7)	0.0030(7)
N2	0.0166(9)	0.0201(9)	0.0114(8)	0.0034(7)	0.0006(6)	0.0014(7)
C1	0.0158(10)	0.0177(10)	0.0084(9)	-0.0005(8)	-0.0018(7)	0.0019(8)

C2	0.0166(10)	0.0273(12)	0.0100(9)	0.0026(9)	0.0014(7)	0.0018(9)
C3	0.0156(10)	0.0270(12)	0.0103(9)	0.0030(9)	0.0001(7)	-0.0011(9)
C4	0.0244(11)	0.0180(11)	0.0145(10)	0.0036(9)	0.0005(8)	0.0027(8)
C5	0.0313(13)	0.0252(13)	0.0246(12)	0.0022(11)	-0.0050(10)	-0.0011(10)
C6	0.0254(12)	0.0266(12)	0.0213(11)	0.0046(10)	0.0010(9)	0.0102(10)
C7	0.0263(13)	0.0378(15)	0.0275(13)	0.0037(11)	0.0135(10)	0.0129(12)
C8	0.0292(13)	0.0396(16)	0.0267(13)	0.0155(12)	0.0075(10)	0.0021(12)
C9	0.0270(12)	0.0206(11)	0.0232(11)	0.0089(10)	0.0034(9)	0.0066(10)
C10	0.0415(16)	0.0395(16)	0.0275(13)	0.0150(13)	-0.0003(11)	0.0159(12)
C11	0.0458(17)	0.0234(13)	0.0385(16)	0.0109(13)	0.0019(13)	0.0004(12)
C12	0.0147(10)	0.0229(11)	0.0132(10)	0.0025(9)	0.0003(7)	0.0057(8)
C13	0.0288(13)	0.0280(13)	0.0238(12)	0.0080(11)	0.0057(10)	0.0077(10)
C14	0.0344(14)	0.0290(14)	0.0471(17)	0.0144(12)	0.0113(12)	0.0166(13)
C15	0.0243(13)	0.0409(16)	0.0429(16)	0.0094(12)	0.0044(11)	0.0293(13)
C16	0.0252(13)	0.0425(16)	0.0223(12)	0.0012(12)	-0.0027(9)	0.0196(12)
C17	0.0204(11)	0.0261(12)	0.0150(10)	-0.0011(9)	-0.0024(8)	0.0077(9)
C18	0.0218(11)	0.0183(11)	0.0115(9)	0.0024(9)	-0.0007(8)	0.0039(8)
C19	0.0296(13)	0.0233(12)	0.0206(11)	0.0029(10)	0.0065(9)	0.0028(10)
C20	0.0430(15)	0.0250(13)	0.0215(12)	0.0105(12)	0.0037(11)	-0.0015(10)
C21	0.0449(16)	0.0179(12)	0.0310(14)	0.0056(12)	-0.0077(12)	-0.0040(11)
C22	0.0279(14)	0.0190(12)	0.0479(17)	-0.0013(11)	-0.0027(12)	0.0054(12)
C23	0.0263(12)	0.0199(12)	0.0272(12)	0.0034(10)	0.0031(9)	0.0057(10)

The general temperature factor expression: $\exp(-2\pi^2(a^{*2}U_{11}h^2 + b^{*2}U_{22}k^2 + c^{*2}U_{33}l^2 + 2a^*b^*U_{12}hk + 2a^*c^*U_{13}hl + 2b^*c^*U_{23}kl))$

Bond lengths (Å)

atom	atom	distance	atom	atom	distance
Ni1	Ni1[1]	2.5112(9)	Ni1	Si1	2.1940(9)
Ni1	Si1[1]	2.2388(10)	Ni1	C1	1.895(2)
Si1	C12	1.898(3)	Si1	C18	1.898(2)
N1	C1	1.368(3)	N1	C2	1.402(3)
N1	C4	1.469(4)	N2	C1	1.354(4)
N2	C3	1.406(3)	N2	C9	1.477(4)
C2	C3	1.348(4)	C2	C7	1.492(4)
C3	C8	1.490(5)	C4	C5	1.525(3)
C4	C6	1.516(4)	C9	C10	1.526(4)
C9	C11	1.524(4)	C12	C13	1.401(4)
C12	C17	1.404(3)	C13	C14	1.396(5)
C14	C15	1.389(5)	C15	C16	1.380(5)

C16	C17	1.392(5)	C18	C19	1.397(4)
C18	C23	1.399(3)	C19	C20	1.392(3)
C20	C21	1.384(4)	C21	C22	1.384(5)
C22	C23	1.387(4)	Ni1	H1	1.59(3)
Si1	H1[1]	1.70(4)			

Symmetry Operators:

(1) -X+1,-Y+1,-Z+1

Bond angles (^o)

atom	atom	atom	angle	atom	atom	atom	angle
Ni1[1]	Ni1	Si1	56.34(3)	Ni1[1]	Ni1	Si1[1]	54.656(19)
Ni1[1]	Ni1	C1	159.88(6)	Si1	Ni1	Si1[1]	110.99(3)
Si1	Ni1	C1	103.55(7)	Si1[1]	Ni1	C1	145.46(6)
Ni1	Si1	Ni1[1]	69.01(3)	Ni1	Si1	C12	116.24(6)
Ni1	Si1	C18	120.72(9)	Ni1[1]	Si1	C12	119.72(8)
Ni1[1]	Si1	C18	123.72(8)	C12	Si1	C18	104.80(10)
C1	N1	C2	110.9(2)	C1	N1	C4	120.1(2)
C2	N1	C4	128.8(2)	C1	N2	C3	111.4(2)
C1	N2	C9	119.8(2)	C3	N2	C9	128.4(2)
Ni1	C1	N1	126.8(2)	Ni1	C1	N2	128.15(18)
N1	C1	N2	104.4(2)	N1	C2	C3	106.8(2)
N1	C2	C7	124.9(3)	C3	C2	C7	128.2(3)
N2	C3	C2	106.4(2)	N2	C3	C8	125.1(3)
C2	C3	C8	128.5(2)	N1	C4	C5	111.6(2)
N1	C4	C6	112.98(19)	C5	C4	C6	113.2(2)
N2	C9	C10	110.64(19)	N2	C9	C11	113.5(2)
C10	C9	C11	113.0(3)	Si1	C12	C13	119.89(18)
Si1	C12	C17	122.9(2)	C13	C12	C17	117.2(3)
C12	C13	C14	121.4(2)	C13	C14	C15	119.9(3)
C14	C15	C16	119.8(3)	C15	C16	C17	120.2(3)
C12	C17	C16	121.4(3)	Si1	C18	C19	121.16(16)
Si1	C18	C23	121.8(2)	C19	C18	C23	116.9(2)
C18	C19	C20	121.8(2)	C19	C20	C21	119.8(3)
C20	C21	C22	119.6(2)	C21	C22	C23	120.2(2)
C18	C23	C22	121.6(3)				

Symmetry Operators:

(1) -X+1,-Y+1,-Z+1

Torsion Angles(^{o})

(Those having bond angles > 160 or < 20 degrees are excluded.)

atom1	atom2	atom3	atom4	angle	atom1	atom2	atom3	atom4	angle
Ni1^{1}	Ni1	Si1	Ni1^{1}	0.0	Ni1^{1}	Ni1	Si1	C12	-113.76(4)
Ni1^{1}	Ni1	Si1	C18	117.62(4)	Si1	Ni1	Ni1^{1}	Si1^{1}	180.00(3)
Si1	Ni1	Ni1^{1}	Si1	0.00(2)	Si1	Ni1	Ni1^{1}	C1^{1}	-179.25(6)
Ni1^{1}	Ni1	Si1^{1}	Ni1^{1}	-0.0	Ni1^{1}	Ni1	Si1^{1}	C12^{1}	-109.04(3)
Ni1^{1}	Ni1	Si1^{1}	C18^{1}	113.69(5)	Si1^{1}	Ni1	Ni1^{1}	Si1^{1}	0.00(2)
Si1^{1}	Ni1	Ni1^{1}	Si1	-180.00(3)	Si1^{1}	Ni1	Ni1^{1}	C1^{1}	0.75(5)
Ni1^{1}	Ni1	C1	N1	-87.2(2)	Ni1^{1}	Ni1	C1	N2	103.0(3)
C1	Ni1	Ni1^{1}	Si1^{1}	179.3(2)	C1	Ni1	Ni1^{1}	Si1	-0.7(2)
C1	Ni1	Ni1^{1}	C1^{1}	-180.0(2)	Si1	Ni1	Si1^{1}	Ni1^{1}	-0.00(3)
Si1	Ni1	Si1^{1}	C12^{1}	-109.04(4)	Si1	Ni1	Si1^{1}	C18^{1}	113.69(4)
Si1^{1}	Ni1	Si1	Ni1^{1}	0.00(3)	Si1^{1}	Ni1	Si1	C12	-113.76(4)
Si1^{1}	Ni1	Si1	C18	117.62(4)	Si1	Ni1	C1	N1	-87.83(14)
Si1	Ni1	C1	N2	102.38(14)	C1	Ni1	Si1	Ni1^{1}	179.74(8)
C1	Ni1	Si1	C12	65.98(9)	C1	Ni1	Si1	C18	-62.64(9)
Si1^{1}	Ni1	C1	N1	91.7(2)	Si1^{1}	Ni1	C1	N2	-78.05(18)
C1	Ni1	Si1^{1}	Ni1^{1}	-179.55(14)	C1	Ni1	Si1^{1}	C12^{1}	71.41(14)
C1	Ni1	Si1^{1}	C18^{1}	-65.86(15)	Ni1	Si1	C12	C13	60.53(17)
Ni1	Si1	C12	C17	-117.38(13)	Ni1	Si1	C18	C19	56.6(2)
Ni1	Si1	C18	C23	-127.38(16)	Ni1^{1}	Si1	C12	C13	-19.18(18)
Ni1^{1}	Si1	C12	C17	162.90(12)	Ni1^{1}	Si1	C18	C19	140.60(14)
Ni1^{1}	Si1	C18	C23	-43.4(2)	C12	Si1	C18	C19	-77.0(2)
C12	Si1	C18	C23	99.07(19)	C18	Si1	C12	C13	-163.47(15)
C18	Si1	C12	C17	18.62(19)	C1	N1	C2	C3	-1.3(2)
C1	N1	C2	C7	-178.72(15)	C2	N1	C1	Ni1	-169.56(14)
C2	N1	C1	N2	2.16(19)	C1	N1	C4	C5	-105.21(19)
C1	N1	C4	C6	125.92(18)	C4	N1	C1	Ni1	5.8(3)
C4	N1	C1	N2	177.52(14)	C2	N1	C4	C5	69.2(3)
C2	N1	C4	C6	-59.6(2)	C4	N1	C2	C3	-176.19(16)
C4	N1	C2	C7	6.4(3)	C1	N2	C3	C2	1.4(2)
C1	N2	C3	C8	-178.51(15)	C3	N2	C1	Ni1	169.37(14)
C3	N2	C1	N1	-2.20(19)	C1	N2	C9	C10	98.0(2)
C1	N2	C9	C11	-133.67(19)	C9	N2	C1	Ni1	-3.8(3)
C9	N2	C1	N1	-175.33(15)	C3	N2	C9	C10	-73.8(3)
C3	N2	C9	C11	54.5(3)	C9	N2	C3	C2	173.83(16)
C9	N2	C3	C8	-6.1(3)	N1	C2	C3	N2	-0.0(2)
N1	C2	C3	C8	179.91(16)	C7	C2	C3	N2	177.21(18)

C7	C2	C3	C8	-2.8(3)	Si1	C12	C13	C14	-178.62(15)
Si1	C12	C17	C16	177.51(14)	C13	C12	C17	C16	-0.5(3)
C17	C12	C13	C14	-0.6(3)	C12	C13	C14	C15	1.3(4)
C13	C14	C15	C16	-1.0(4)	C14	C15	C16	C17	-0.1(4)
C15	C16	C17	C12	0.8(4)	Si1	C18	C19	C20	175.18(18)
Si1	C18	C23	C22	-174.10(18)	C19	C18	C23	C22	2.1(4)
C23	C18	C19	C20	-1.0(4)	C18	C19	C20	C21	-0.6(5)
C19	C20	C21	C22	1.2(5)	C20	C21	C22	C23	-0.2(5)
C21	C22	C23	C18	-1.5(5)					

Symmetry Operators:

(1) -X+1,-Y+1,-Z+1

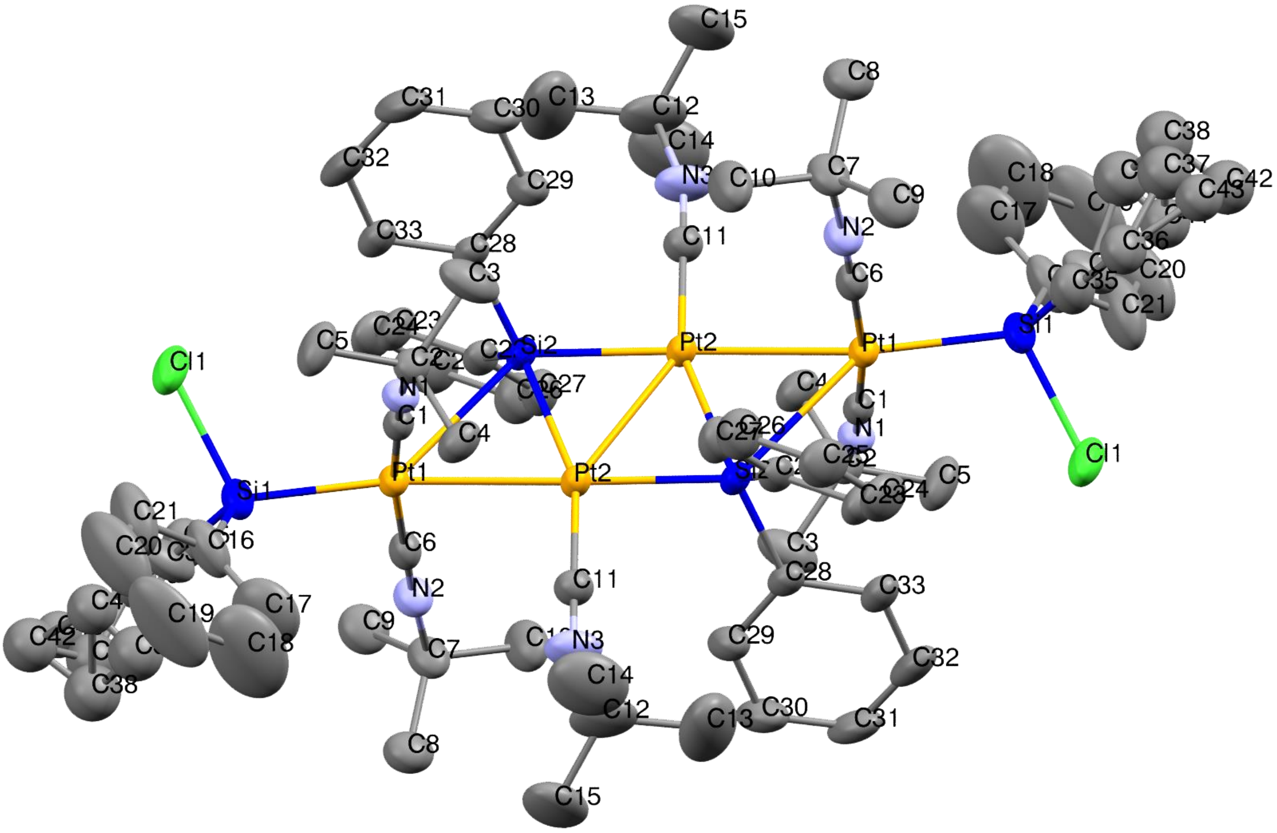

Supplementary Fig. 51. ORTEP drawing of **8** (50% probability of the thermal ellipsoids). Hydrogen atoms were omitted for clarity. Two phenyl groups were found to be disordered. The site occupancy factor for the carbons derived from the methyl groups on the disordered Ph groups was defined as follows: 0.5 for C37, C38, C39, C41, C42 and C43, respectively.

Supplementary Table 16. Crystal data and structure refinement for **8**.

Empirical Formula	$C_{78}H_{94}Cl_2N_6Pt_4Si_4$
Formula Weight	2079.25
Crystal Color, Habit	red, platelet
Crystal Dimensions	0.040 X 0.020 X 0.010 mm
Crystal System	monoclinic
Lattice Type	Primitive
Lattice Parameters	a = 15.9918(10) Å
	b = 12.5337(8) Å
	c = 20.6258(12) Å
	β = 90.331(6) ^{o}
	V = 4134.1(4) Å^3
Space Group	$P2_1/n$ (#14)
Z value	2
D_{calc}	1.670 g/cm^3
F_{000}	2012.00
Diffractometer	R-AXIS IV
Radiation (λ = 0.41330Å)	monochromated
Voltage, Current	8kV, 100mA
Temperature	-173.0^{o}C
Detector Aperture	300.0 x 300.0 mm
Data Images	1080 exposures
ω oscillation Range (χ=45.0, φ=0.0)	0.0 - 180.0^{o}
Exposure Rate	120.0 sec./^{o}
Detector Swing Angle	0.00^{o}
ω oscillation Range (χ=45.0, φ=90.0)	0.0 - 180.0^{o}
Exposure Rate	120.0 sec./^{o}
ω oscillation Range (χ=45.0, φ=180.0)	0.0 - 180.0^{o}
Exposure Rate	120.0 sec./^{o}
Detector Position	130.00 mm
Pixel Size	0.172 mm
$2\theta_{max}$	31.1^{o}
No. of Reflections Measured	Total: 88644
	Unique: 9323 (R_{int} = 0.0594)
Corrections	Lorentz-polarization
	Absorption
	(trans. factors: 0.825 - 1.000)
Structure Solution	Direct Methods (SHELXT Version 2014/5)

Refinement	Full-matrix least-squares on F^2
Function Minimized	Σ w $(Fo^2 - Fc^2)^2$
Least Squares Weights	w = 1/ [$\sigma^2(Fo^2)$ + $(0.0267 \cdot P)^2$
	+ 10.8739 · P]
	where P = (Max(Fo^2,0) + $2Fc^2$)/3
$2\theta_{max}$ cutoff	31.1^o
Anomalous Dispersion	All non-hydrogen atoms
No. Observations (All reflections)	9323
No. Variables	430
Reflection/Parameter Ratio	21.68
Residuals: R1 (I>2.00σ(I))	0.0259
Residuals: R (All reflections)	0.0270
Residuals: wR2 (All reflections)	0.0650
Goodness of Fit Indicator	1.025
Max Shift/Error in Final Cycle	0.003
Maximum peak in Final Diff. Map	2.14 $e^-/Å^3$
Minimum peak in Final Diff. Map	-1.45 $e^-/Å^3$

Atomic coordinates and B_{iso}/B_{eq}

atom	x	y	z	B_{eq}	
Pt1	0.69804(2)	0.36099(2)	0.48647(2)	1.887(3)	1
Pt2	0.58392(2)	0.51829(2)	0.50157(2)	1.923(3)	1
Cl1	0.78607(9)	0.13034(10)	0.39808(7)	4.90(3)	1
Si1	0.81731(6)	0.25881(9)	0.46313(5)	2.651(17)	1
Si2	0.52009(6)	0.34949(7)	0.48352(5)	2.061(14)	1
N1	0.70305(19)	0.4392(3)	0.34449(14)	2.37(5)	1
N2	0.6989(2)	0.3337(3)	0.63549(15)	2.83(5)	1
N3	0.7204(3)	0.6909(3)	0.50657(19)	4.00(7)	1
C1	0.6983(2)	0.4073(3)	0.39690(17)	2.19(5)	1
C2	0.7072(2)	0.4902(3)	0.28099(16)	2.21(5)	1
C3	0.6202(3)	0.4879(5)	0.2516(3)	4.56(11)	1
C4	0.7360(3)	0.6051(3)	0.2921(2)	3.28(7)	1
C5	0.7710(3)	0.4285(4)	0.2405(2)	3.81(8)	1
C6	0.6968(2)	0.3400(3)	0.57987(18)	2.44(5)	1
C7	0.7007(3)	0.3328(4)	0.70611(18)	3.14(7)	1
C8	0.7795(3)	0.3923(4)	0.7268(2)	4.03(8)	1
C9	0.7035(3)	0.2160(4)	0.7272(2)	3.98(8)	1
C10	0.6207(3)	0.3867(5)	0.7297(2)	4.47(10)	1
C11	0.6701(2)	0.6256(3)	0.50637(19)	2.75(6)	1
C12	0.7607(4)	0.7957(5)	0.5054(3)	5.34(12)	1
C13	0.6941(6)	0.8772(6)	0.5296(6)	9.6(3)	1
C14	0.7793(6)	0.8217(7)	0.4374(4)	8.1(2)	1
C15	0.8348(4)	0.7903(6)	0.5516(4)	7.09(18)	1
C16	0.8997(3)	0.3357(5)	0.4186(2)	4.24(10)	1
C17	0.9109(4)	0.4425(7)	0.4332(4)	7.25(18)	1
C18	0.9710(6)	0.5042(9)	0.4013(7)	11.5(4)	1
C19	1.0205(5)	0.4525(11)	0.3524(5)	10.1(4)	1
C20	1.0051(5)	0.3500(11)	0.3350(4)	9.1(3)	1
C21	0.9476(4)	0.2916(7)	0.3677(3)	6.53(17)	1
C22	0.5113(2)	0.2413(3)	0.54769(18)	2.48(6)	1
C23	0.5399(3)	0.1363(3)	0.5419(2)	3.56(8)	1
C24	0.5295(4)	0.0625(3)	0.5927(3)	4.29(9)	1
C25	0.4918(3)	0.0921(4)	0.6491(3)	4.07(9)	1
C26	0.4628(3)	0.1953(4)	0.6560(2)	3.92(8)	1
C27	0.4725(3)	0.2681(3)	0.6062(2)	3.12(7)	1
C28	0.5129(2)	0.2882(3)	0.39926(18)	2.50(6)	1
C29	0.4513(3)	0.3243(4)	0.3558(2)	3.27(7)	1

C30	0.4466(3)	0.2870(5)	0.2926(2)	4.41(10)	1
C31	0.5039(3)	0.2127(4)	0.2703(2)	4.36(10)	1
C32	0.5649(3)	0.1745(4)	0.3118(2)	3.95(9)	1
C33	0.5696(3)	0.2114(3)	0.3756(2)	3.02(7)	1
C34	0.8714(3)	0.1813(4)	0.5299(3)	4.47(10)	1
C35	0.8213(3)	0.1249(4)	0.5769(3)	4.37(9)	1
C36	0.8563(3)	0.0637(4)	0.6252(3)	4.39(9)	1
C37	0.9368(8)	0.0933(12)	0.6374(7)	5.4(2)	1/2
C38	0.9790(9)	0.1587(12)	0.6115(7)	6.0(3)	1/2
C39	0.9512(9)	0.2193(11)	0.5579(7)	5.8(2)	1/2
C41	0.9532(7)	0.1564(10)	0.5344(6)	4.8(2)	1/2
C42	0.9900(8)	0.0766(11)	0.5861(6)	5.5(2)	1/2
C43	0.9412(7)	0.0434(10)	0.6284(5)	4.15(18)	1/2

$B_{eq} = 8/3\ \pi^2(U_{11}(aa^*)^2 + U_{22}(bb^*)^2 + U_{33}(cc^*)^2 + 2U_{12}(aa^*bb^*)\cos\gamma + 2U_{13}(aa^*cc^*)\cos\beta + 2U_{23}(bb^*cc^*)\cos\alpha)$

Anisotropic displacement parameters

atom	U_{11}	U_{22}	U_{33}	U_{12}	U_{13}	U_{23}
Pt1	0.02431(7)	0.02574(7)	0.02169(7)	0.00127(4)	0.00259(5)	-0.00151(4)
Pt2	0.02351(7)	0.02231(7)	0.02728(7)	-0.00066(4)	0.00347(5)	-0.00280(4)
Cl1	0.0738(8)	0.0525(7)	0.0597(7)	0.0299(6)	-0.0124(6)	-0.0232(6)
Si1	0.0315(5)	0.0405(5)	0.0287(5)	0.0115(4)	-0.0006(4)	0.0020(4)
Si2	0.0281(4)	0.0205(4)	0.0298(5)	0.0001(3)	0.0026(4)	-0.0042(3)
N1	0.0304(14)	0.0356(16)	0.0241(14)	-0.0011(12)	-0.0006(11)	0.0013(12)
N2	0.0379(16)	0.0439(18)	0.0258(15)	0.0017(14)	0.0032(12)	0.0042(13)
N3	0.053(2)	0.051(2)	0.047(2)	-0.0273(18)	-0.0030(17)	0.0023(17)
C1	0.0247(15)	0.0269(16)	0.0316(17)	0.0014(12)	0.0011(12)	-0.0043(13)
C2	0.0331(17)	0.0283(16)	0.0227(15)	-0.0009(13)	0.0004(13)	0.0031(12)
C3	0.048(3)	0.072(3)	0.054(3)	-0.016(2)	-0.019(2)	0.027(2)
C4	0.056(2)	0.0302(18)	0.039(2)	-0.0035(17)	0.0056(18)	-0.0041(16)
C5	0.071(3)	0.036(2)	0.039(2)	0.010(2)	0.020(2)	-0.0001(17)
C6	0.0268(16)	0.0321(17)	0.0338(18)	0.0029(13)	0.0042(13)	0.0005(14)
C7	0.039(2)	0.057(2)	0.0229(16)	0.0039(18)	0.0018(14)	0.0044(16)
C8	0.053(3)	0.059(3)	0.041(2)	-0.004(2)	-0.0074(19)	0.001(2)
C9	0.055(3)	0.061(3)	0.036(2)	-0.003(2)	0.0055(18)	0.0160(19)
C10	0.051(3)	0.079(3)	0.040(2)	0.012(2)	0.011(2)	-0.007(2)
C11	0.0335(18)	0.0345(18)	0.0364(19)	-0.0046(14)	0.0009(15)	-0.0008(14)
C12	0.076(4)	0.061(3)	0.066(3)	-0.044(3)	-0.004(3)	-0.001(3)
C13	0.095(6)	0.057(4)	0.211(12)	-0.010(4)	0.012(6)	-0.033(5)

C14	0.102(6)	0.093(5)	0.113(6)	-0.051(5)	0.002(5)	0.038(5)
C15	0.060(4)	0.077(4)	0.132(6)	-0.029(3)	-0.025(4)	-0.007(4)
C16	0.032(2)	0.078(3)	0.051(3)	0.013(2)	0.0104(18)	0.026(2)
C17	0.061(4)	0.108(6)	0.106(5)	-0.025(4)	0.029(4)	0.029(5)
C18	0.092(6)	0.132(8)	0.213(13)	-0.032(6)	0.047(7)	0.073(8)
C19	0.051(4)	0.190(11)	0.144(8)	-0.016(5)	0.035(4)	0.099(8)
C20	0.056(4)	0.201(11)	0.089(5)	0.014(5)	0.037(4)	0.059(6)
C21	0.059(3)	0.121(6)	0.069(4)	0.034(4)	0.027(3)	0.024(4)
C22	0.0318(17)	0.0223(15)	0.0402(19)	-0.0040(13)	0.0007(14)	-0.0010(13)
C23	0.059(3)	0.0266(19)	0.049(2)	0.0066(17)	-0.003(2)	-0.0072(16)
C24	0.071(3)	0.0229(19)	0.070(3)	0.0007(19)	-0.007(2)	0.0029(19)
C25	0.064(3)	0.032(2)	0.059(3)	-0.0085(19)	0.002(2)	0.0118(19)
C26	0.053(3)	0.045(2)	0.051(2)	-0.0029(19)	0.013(2)	0.009(2)
C27	0.041(2)	0.0292(18)	0.048(2)	0.0008(15)	0.0116(17)	0.0049(16)
C28	0.0290(16)	0.0305(17)	0.0355(18)	-0.0072(13)	0.0045(14)	-0.0094(14)
C29	0.0321(18)	0.054(2)	0.038(2)	-0.0044(17)	0.0001(15)	-0.0130(18)
C30	0.045(2)	0.079(3)	0.043(2)	-0.011(2)	-0.0062(19)	-0.021(2)
C31	0.056(3)	0.066(3)	0.044(2)	-0.021(2)	0.007(2)	-0.029(2)
C32	0.053(2)	0.041(2)	0.057(3)	-0.0160(19)	0.019(2)	-0.027(2)
C33	0.0378(19)	0.0302(18)	0.047(2)	-0.0059(15)	0.0070(16)	-0.0124(16)
C34	0.046(2)	0.060(3)	0.063(3)	-0.006(2)	-0.024(2)	0.022(2)
C35	0.054(3)	0.045(2)	0.067(3)	0.003(2)	0.002(2)	0.004(2)
C36	0.059(3)	0.052(3)	0.056(3)	0.006(2)	-0.003(2)	0.003(2)

The general temperature factor expression: $\exp(-2\pi^2(a^{*2}U_{11}h^2 + b^{*2}U_{22}k^2 + c^{*2}U_{33}l^2 + 2a^*b^*U_{12}hk + 2a^*c^*U_{13}hl + 2b^*c^*U_{23}kl))$

Bond lengths (Å)

atom	atom	distance	atom	atom	distance
Pt1	Pt2	2.7058(4)	Pt1	Si1	2.3496(11)
Pt1	Si2	2.8497(10)	Pt1	C1	1.936(4)
Pt1	C6	1.945(4)	Pt2	Pt2[1]	2.7233(5)
Pt2	Si2	2.3775(9)	Pt2	Si2[1]	2.3693(10)
Pt2	C11	1.928(4)	Cl1	Si1	2.1529(17)
Si1	C16	1.876(5)	Si1	C34	1.890(5)
Si2	C22	1.901(4)	Si2	C28	1.903(4)
N1	C1	1.156(5)	N1	C2	1.459(4)
N2	C6	1.150(5)	N2	C7	1.457(5)
N3	C11	1.148(6)	N3	C12	1.463(7)
C2	C3	1.515(6)	C2	C4	1.528(5)

C2	C5	1.532(6)	C7	C8	1.524(7)
C7	C9	1.527(7)	C7	C10	1.528(7)
C12	C13	1.561(11)	C12	C14	1.472(11)
C12	C15	1.518(10)	C16	C17	1.384(10)
C16	C21	1.416(8)	C17	C18	1.400(14)
C18	C19	1.440(16)	C19	C20	1.356(19)
C20	C21	1.357(12)	C22	C23	1.399(5)
C22	C27	1.401(6)	C23	C24	1.408(7)
C24	C25	1.364(8)	C25	C26	1.382(6)
C26	C27	1.384(6)	C28	C29	1.402(5)
C28	C33	1.412(5)	C29	C30	1.386(6)
C30	C31	1.386(7)	C31	C32	1.379(7)
C32	C33	1.397(6)	C34	C35	1.446(8)
C34	C39	1.477(15)	C34	C41	1.347(13)
C35	C36	1.374(8)	C36	C37	1.363(14)
C36	C43	1.383(12)	C37	C38	1.19(2)
C37	C42	1.376(19)	C37	C43	0.656(19)
C38	C39	1.41(2)	C38	C41	1.642(19)
C38	C42	1.17(2)	C38	C43	1.606(19)
C39	C41	0.926(19)	C39	C42	1.979(19)
C41	C42	1.575(18)	C42	C43	1.245(17)

Symmetry Operators:

(1) -X+1,-Y+1,-Z+1

Bond angles (⁰)

atom	atom	atom	angle	atom	atom	atom	angle
Pt2	Pt1	Si1	165.84(3)	Pt2	Pt1	Si2	50.59(2)
Pt2	Pt1	C1	84.07(10)	Pt2	Pt1	C6	88.51(11)
Si1	Pt1	Si2	141.24(3)	Si1	Pt1	C1	87.80(10)
Si1	Pt1	C6	98.16(11)	Si2	Pt1	C1	90.13(10)
Si2	Pt1	C6	89.93(11)	C1	Pt1	C6	170.33(15)
Pt1	Pt2	$Pt2^1$	122.677(13)	Pt1	Pt2	Si2	67.84(2)
Pt1	Pt2	$Si2^1$	177.55(3)	Pt1	Pt2	C11	91.83(12)
$Pt2^1$	Pt2	Si2	54.85(2)	$Pt2^1$	Pt2	$Si2^1$	55.13(2)
$Pt2^1$	Pt2	C11	145.36(12)	Si2	Pt2	$Si2^1$	109.98(3)
Si2	Pt2	C11	159.25(12)	$Si2^1$	Pt2	C11	90.42(12)
Pt1	Si1	Cl1	110.47(6)	Pt1	Si1	C16	113.18(18)
Pt1	Si1	C34	119.97(17)	Cl1	Si1	C16	103.89(18)
Cl1	Si1	C34	99.95(17)	C16	Si1	C34	107.5(2)

Pt1	Si2	Pt2	61.57(2)	Pt1	Si2	Pt2[1]	131.57(4)
Pt1	Si2	C22	95.68(11)	Pt1	Si2	C28	95.47(11)
Pt2	Si2	Pt2[1]	70.02(3)	Pt2	Si2	C22	124.00(12)
Pt2	Si2	C28	121.74(12)	Pt2[1]	Si2	C22	110.73(12)
Pt2[1]	Si2	C28	111.21(12)	C22	Si2	C28	110.09(16)
C1	N1	C2	174.2(3)	C6	N2	C7	176.4(4)
C11	N3	C12	161.5(5)	Pt1	C1	N1	175.3(3)
N1	C2	C3	107.7(3)	N1	C2	C4	107.0(3)
N1	C2	C5	107.5(3)	C3	C2	C4	110.7(3)
C3	C2	C5	112.7(3)	C4	C2	C5	110.9(3)
Pt1	C6	N2	175.6(3)	N2	C7	C8	106.7(3)
N2	C7	C9	107.0(3)	N2	C7	C10	107.7(3)
C8	C7	C9	111.5(4)	C8	C7	C10	112.8(4)
C9	C7	C10	110.9(4)	Pt2	C11	N3	177.0(4)
N3	C12	C13	106.3(5)	N3	C12	C14	107.8(5)
N3	C12	C15	106.9(5)	C13	C12	C14	107.6(7)
C13	C12	C15	111.1(6)	C14	C12	C15	116.5(6)
Si1	C16	C17	118.8(4)	Si1	C16	C21	123.2(5)
C17	C16	C21	117.9(6)	C16	C17	C18	121.4(8)
C17	C18	C19	117.5(10)	C18	C19	C20	120.8(9)
C19	C20	C21	120.1(9)	C16	C21	C20	122.0(8)
Si2	C22	C23	125.9(3)	Si2	C22	C27	117.6(3)
C23	C22	C27	116.5(4)	C22	C23	C24	120.9(4)
C23	C24	C25	120.7(4)	C24	C25	C26	119.5(4)
C25	C26	C27	120.1(4)	C22	C27	C26	122.2(4)
Si2	C28	C29	119.5(3)	Si2	C28	C33	123.7(3)
C29	C28	C33	116.8(4)	C28	C29	C30	121.7(4)
C29	C30	C31	120.4(4)	C30	C31	C32	119.6(4)
C31	C32	C33	120.2(4)	C28	C33	C32	121.3(4)
Si1	C34	C35	119.1(4)	Si1	C34	C39	120.7(6)
Si1	C34	C41	127.5(6)	C35	C34	C39	112.0(7)
C35	C34	C41	112.5(7)	C39	C34	C41	38.0(8)
C34	C35	C36	122.3(5)	C35	C36	C37	111.2(7)
C35	C36	C43	122.2(7)	C37	C36	C43	27.6(8)
C36	C37	C38	130.0(13)	C36	C37	C42	113.9(11)
C36	C37	C43	77.9(16)	C38	C37	C42	53.6(11)
C38	C37	C43	118(2)	C42	C37	C43	64.6(15)
C37	C38	C39	123.0(14)	C37	C38	C41	106.3(12)
C37	C38	C42	71.4(13)	C37	C38	C43	21.1(9)

C39	C38	C41	34.3(8)	C39	C38	C42	99.8(13)
C39	C38	C43	122.4(12)	C41	C38	C42	65.7(10)
C41	C38	C43	95.8(10)	C42	C38	C43	50.4(9)
C34	C39	C38	113.7(11)	C34	C39	C41	63.4(11)
C34	C39	C42	95.3(8)	C38	C39	C41	86.7(14)
C38	C39	C42	35.6(8)	C41	C39	C42	51.3(11)
C34	C41	C38	107.6(9)	C34	C41	C39	78.6(12)
C34	C41	C42	123.6(10)	C38	C41	C39	59.1(12)
C38	C41	C42	42.5(8)	C39	C41	C42	101.4(13)
C37	C42	C38	55.1(11)	C37	C42	C39	83.9(10)
C37	C42	C41	101.2(11)	C37	C42	C43	28.4(9)
C38	C42	C39	44.6(9)	C38	C42	C41	71.8(11)
C38	C42	C43	83.4(12)	C39	C42	C41	27.3(6)
C39	C42	C43	108.1(11)	C41	C42	C43	117.0(11)
C36	C43	C37	74.5(15)	C36	C43	C38	101.3(9)
C36	C43	C42	121.6(11)	C37	C43	C38	40.9(15)
C37	C43	C42	87.0(17)	C38	C43	C42	46.3(9)

Symmetry Operators:

(1) -X+1,-Y+1,-Z+1

Torsion Angles(^{o})

(Those having bond angles > 160 or < 20 degrees are excluded.)

Pt2	Pt1	Si2	Pt2	0.000(11)	Pt2	Pt1	Si2	$Pt2^{1}$	-1.51(4)
Pt2	Pt1	Si2	C22	-125.82(5)	Pt2	Pt1	Si2	C28	123.30(4)
Si2	Pt1	Pt2	$Pt2^{1}$	1.17(3)	Si2	Pt1	Pt2	Si2	-0.00(3)
Si2	Pt1	Pt2	C11	-175.69(3)	C1	Pt1	Pt2	$Pt2^{1}$	96.23(10)
C1	Pt1	Pt2	Si2	95.07(10)	C1	Pt1	Pt2	C11	-80.62(10)
C6	Pt1	Pt2	$Pt2^{1}$	-89.97(11)	C6	Pt1	Pt2	Si2	-91.14(11)
C6	Pt1	Pt2	C11	93.18(11)	Si1	Pt1	Si2	Pt2	-168.86(5)
Si1	Pt1	Si2	$Pt2^{1}$	-170.37(5)	Si1	Pt1	Si2	C22	65.32(7)
Si1	Pt1	Si2	C28	-45.55(7)	Si2	Pt1	Si1	Cl1	19.78(8)
Si2	Pt1	Si1	C16	135.80(5)	Si2	Pt1	Si1	C34	-95.57(7)
C1	Pt1	Si1	Cl1	-67.68(11)	C1	Pt1	Si1	C16	48.33(11)
C1	Pt1	Si1	C34	176.96(11)	C6	Pt1	Si1	Cl1	119.96(11)
C6	Pt1	Si1	C16	-124.03(12)	C6	Pt1	Si1	C34	4.61(12)
C1	Pt1	Si2	Pt2	-82.21(10)	C1	Pt1	Si2	$Pt2^{1}$	-83.72(11)
C1	Pt1	Si2	C22	151.97(11)	C1	Pt1	Si2	C28	41.10(10)
C6	Pt1	Si2	Pt2	88.13(11)	C6	Pt1	Si2	$Pt2^{1}$	86.62(12)
C6	Pt1	Si2	C22	-37.69(11)	C6	Pt1	Si2	C28	-148.57(11)

Pt1	Pt2	Pt2^1	Pt1^1	180.000(18)	Pt1	Pt2	Pt2^1	Si2^1	178.68(2)
Pt1	Pt2	Pt2^1	Si2	-1.323(18)	Pt1	Pt2	Pt2^1	C11^1	5.54(5)
Pt1	Pt2	Si2	Pt1	0.0	Pt1	Pt2	Si2	Pt2^1	178.80(3)
Pt1	Pt2	Si2	C22	76.73(5)	Pt1	Pt2	Si2	C28	-78.03(5)
Pt2^1	Pt2	Si2	Pt1	-178.80(3)	Pt2^1	Pt2	Si2	Pt2^1	0.000(11)
Pt2^1	Pt2	Si2	C22	-102.07(6)	Pt2^1	Pt2	Si2	C28	103.17(6)
Si2	Pt2	Pt2^1	Pt1^1	-178.68(4)	Si2	Pt2	Pt2^1	Si2^1	180.00(3)
Si2	Pt2	Pt2^1	Si2	0.00(3)	Si2	Pt2	Pt2^1	C11^1	6.86(5)
Pt2^1	Pt2	Si2^1	Pt1^1	-1.41(4)	Pt2^1	Pt2	Si2^1	Pt2^1	-0.000(10)
Pt2^1	Pt2	Si2^1	C22^1	-119.91(5)	Pt2^1	Pt2	Si2^1	C28^1	117.34(5)
Si2^1	Pt2	Pt2^1	Pt1^1	1.32(3)	Si2^1	Pt2	Pt2^1	Si2^1	-0.00(3)
Si2^1	Pt2	Pt2^1	Si2	-180.00(3)	Si2^1	Pt2	Pt2^1	C11^1	-173.14(5)
C11	Pt2	Pt2^1	Pt1^1	-5.5(2)	C11	Pt2	Pt2^1	Si2^1	-6.9(2)
C11	Pt2	Pt2^1	Si2	173.1(2)	C11	Pt2	Pt2^1	C11^1	180.0(2)
Si2	Pt2	Si2^1	Pt1^1	-1.41(7)	Si2	Pt2	Si2^1	Pt2^1	-0.00(4)
Si2	Pt2	Si2^1	C22^1	-119.91(5)	Si2	Pt2	Si2^1	C28^1	117.34(5)
Si2^1	Pt2	Si2	Pt1	-178.80(3)	Si2^1	Pt2	Si2	Pt2^1	0.00(4)
Si2^1	Pt2	Si2	C22	-102.07(6)	Si2^1	Pt2	Si2	C28	103.17(6)
C11	Pt2	Si2	Pt1	12.2(3)	C11	Pt2	Si2	Pt2^1	-169.0(3)
C11	Pt2	Si2	C22	89.0(3)	C11	Pt2	Si2	C28	-65.8(3)
C11	Pt2	Si2^1	Pt1^1	174.69(13)	C11	Pt2	Si2^1	Pt2^1	176.11(12)
C11	Pt2	Si2^1	C22^1	56.19(12)	C11	Pt2	Si2^1	C28^1	-66.55(12)
Pt1	Si1	C16	C17	35.4(4)	Pt1	Si1	C16	C21	-140.3(3)
Pt1	Si1	C34	C35	41.4(4)	Pt1	Si1	C34	C39	-104.7(3)
Pt1	Si1	C34	C41	-150.3(3)	Cl1	Si1	C16	C17	155.3(3)
Cl1	Si1	C16	C21	-20.5(4)	Cl1	Si1	C34	C35	-79.4(3)
Cl1	Si1	C34	C39	134.5(3)	Cl1	Si1	C34	C41	89.0(4)
C16	Si1	C34	C35	172.5(3)	C16	Si1	C34	C39	26.4(4)
C16	Si1	C34	C41	-19.1(5)	C34	Si1	C16	C17	-99.4(4)
C34	Si1	C16	C21	84.9(4)	Pt1	Si2	C22	C23	-65.4(3)
Pt1	Si2	C22	C27	114.9(2)	Pt1	Si2	C28	C29	-138.4(2)
Pt1	Si2	C28	C33	37.7(3)	Pt2	Si2	C22	C23	-124.8(2)
Pt2	Si2	C22	C27	55.6(3)	Pt2	Si2	C28	C29	-78.6(3)
Pt2	Si2	C28	C33	97.5(3)	Pt2^1	Si2	C22	C23	155.9(2)
Pt2^1	Si2	C22	C27	-23.7(3)	Pt2^1	Si2	C28	C29	0.4(3)
Pt2^1	Si2	C28	C33	176.5(2)	C22	Si2	C28	C29	123.5(2)
C22	Si2	C28	C33	-60.4(3)	C28	Si2	C22	C23	32.5(3)
C28	Si2	C22	C27	-147.1(2)	Si1	C16	C17	C18	-179.5(4)
Si1	C16	C21	C20	178.1(4)	C17	C16	C21	C20	2.3(8)

C21	C16	C17	C18	-3.5(9)	C16	C17	C18	C19	0.2(13)
C17	C18	C19	C20	4.6(15)	C18	C19	C20	C21	-6.0(14)
C19	C20	C21	C16	2.5(12)	Si2	C22	C23	C24	-179.7(3)
Si2	C22	C27	C26	179.9(2)	C23	C22	C27	C26	0.3(6)
C27	C22	C23	C24	-0.0(6)	C22	C23	C24	C25	-0.4(7)
C23	C24	C25	C26	0.5(8)	C24	C25	C26	C27	-0.3(7)
C25	C26	C27	C22	-0.1(7)	Si2	C28	C29	C30	175.9(3)
Si2	C28	C33	C32	-175.4(2)	C29	C28	C33	C32	0.7(5)
C33	C28	C29	C30	-0.4(6)	C28	C29	C30	C31	-0.4(7)
C29	C30	C31	C32	1.0(7)	C30	C31	C32	C33	-0.7(7)
C31	C32	C33	C28	-0.2(6)	Si1	C34	C35	C36	177.5(3)
Si1	C34	C39	C38	173.9(6)	Si1	C34	C39	C41	-113.0(7)
Si1	C34	C39	C42	-154.5(4)	Si1	C34	C41	C38	145.7(5)
Si1	C34	C41	C39	93.5(8)	Si1	C34	C41	C42	-170.2(5)
C35	C34	C39	C38	25.6(12)	C35	C34	C39	C41	98.7(8)
C35	C34	C39	C42	57.2(7)	C39	C34	C35	C36	-33.7(9)
C35	C34	C41	C38	-45.4(9)	C35	C34	C41	C39	-97.5(8)
C35	C34	C41	C42	-1.2(12)	C41	C34	C35	C36	7.5(9)
C39	C34	C41	C38	52.1(10)	C39	C34	C41	C39	-0.0(9)
C39	C34	C41	C42	96.3(14)	C41	C34	C39	C38	-73.1(13)
C41	C34	C39	C41	0.0(8)	C41	C34	C39	C42	-41.4(10)
C34	C35	C36	C37	22.2(7)	C34	C35	C36	C43	-6.3(8)
C35	C36	C37	C38	-2.9(17)	C35	C36	C37	C42	-64.5(12)
C35	C36	C37	C43	-119.4(11)	C35	C36	C43	C37	73.6(11)
C35	C36	C43	C38	42.4(9)	C35	C36	C43	C42	-2.7(14)
C37	C36	C43	C37	0.0(12)	C37	C36	C43	C38	-31.2(14)
C37	C36	C43	C42	-76.4(17)	C43	C36	C37	C38	117(2)
C43	C36	C37	C42	55.0(14)	C43	C36	C37	C43	0.0(10)
C36	C37	C38	C39	-2(3)	C36	C37	C38	C41	-36(2)
C36	C37	C38	C42	-92.1(16)	C36	C37	C38	C43	-98(2)
C36	C37	C42	C38	123.2(13)	C36	C37	C42	C39	86.2(11)
C36	C37	C42	C41	65.0(13)	C36	C37	C42	C43	-62.4(12)
C36	C37	C43	C36	0.0(2)	C36	C37	C43	C38	129.1(16)
C36	C37	C43	C42	124.0(7)	C38	C37	C42	C38	0.0(9)
C38	C37	C42	C39	-37.0(10)	C38	C37	C42	C41	-58.3(11)
C38	C37	C42	C43	174.4(18)	C42	C37	C38	C39	89.8(15)
C42	C37	C38	C41	56.5(10)	C42	C37	C38	C42	-0.0(7)
C42	C37	C38	C43	-5.7(18)	C38	C37	C43	C36	-129.1(18)
C38	C37	C43	C38	0.0(8)	C38	C37	C43	C42	-5.1(18)

C43	C37	C38	C39	96(2)	C43	C37	C38	C41	62(2)
C43	C37	C38	C42	6(2)	C43	C37	C38	C43	-0.0(11)
C42	C37	C43	C36	-124.0(9)	C42	C37	C43	C38	5.1(17)
C42	C37	C43	C42	0.0(6)	C43	C37	C42	C38	-174.4(19)
C43	C37	C42	C39	148.6(16)	C43	C37	C42	C41	127.4(16)
C43	C37	C42	C43	-0.0(11)	C37	C38	C39	C34	-10(2)
C37	C38	C39	C41	-69.2(18)	C37	C38	C39	C42	-74.1(16)
C37	C38	C41	C34	60.7(14)	C37	C38	C41	C39	125.2(14)
C37	C38	C41	C42	-60.1(11)	C37	C38	C42	C37	0.0(7)
C37	C38	C42	C39	121.6(13)	C37	C38	C42	C41	118.6(10)
C37	C38	C42	C43	-2.7(9)	C37	C38	C43	C36	50(2)
C37	C38	C43	C37	-0.0(18)	C37	C38	C43	C42	173(2)
C39	C38	C41	C34	-64.5(13)	C39	C38	C41	C39	-0.0(10)
C39	C38	C41	C42	174.7(15)	C41	C38	C39	C34	59.0(11)
C41	C38	C39	C41	-0.0(7)	C41	C38	C39	C42	-4.9(14)
C39	C38	C42	C37	-121.6(12)	C39	C38	C42	C39	0.0(6)
C39	C38	C42	C41	-3.0(8)	C39	C38	C42	C43	-124.3(11)
C42	C38	C39	C34	63.9(15)	C42	C38	C39	C41	4.9(14)
C42	C38	C39	C42	0.0(7)	C39	C38	C43	C36	-48.6(15)
C39	C38	C43	C37	-98.2(17)	C39	C38	C43	C42	74.7(13)
C43	C38	C39	C34	15.0(18)	C43	C38	C39	C41	-44.0(15)
C43	C38	C39	C42	-48.9(10)	C41	C38	C42	C37	-118.6(9)
C41	C38	C42	C39	3.0(8)	C41	C38	C42	C41	0.0(4)
C41	C38	C42	C43	-121.3(9)	C42	C38	C41	C34	120.8(12)
C42	C38	C41	C39	-174.7(14)	C42	C38	C41	C42	0.0(7)
C41	C38	C43	C36	-71.7(9)	C41	C38	C43	C37	-121.4(15)
C41	C38	C43	C42	51.5(7)	C43	C38	C41	C34	79.4(10)
C43	C38	C41	C39	143.9(11)	C43	C38	C41	C42	-41.4(6)
C42	C38	C43	C36	-123.3(12)	C42	C38	C43	C37	-172.9(19)
C42	C38	C43	C42	-0.0(8)	C43	C38	C42	C37	2.7(7)
C43	C38	C42	C39	124.3(10)	C43	C38	C42	C41	121.3(9)
C43	C38	C42	C43	-0.0(5)	C34	C39	C41	C34	0.0(2)
C34	C39	C41	C38	-118.7(8)	C34	C39	C41	C42	-122.3(9)
C34	C39	C42	C37	-79.7(8)	C34	C39	C42	C38	-124.3(10)
C34	C39	C42	C41	49.4(7)	C34	C39	C42	C43	-64.6(10)
C38	C39	C41	C34	118.7(9)	C38	C39	C41	C38	0.0(6)
C38	C39	C41	C42	-3.7(10)	C38	C39	C42	C37	44.6(11)
C38	C39	C42	C38	0.0(10)	C38	C39	C42	C41	173.7(18)
C38	C39	C42	C43	59.7(13)	C41	C39	C42	C37	-129.1(14)

C41	C39	C42	C38	-173.7(17)	C41	C39	C42	C41	0.0(9)
C41	C39	C42	C43	-114.0(15)	C42	C39	C41	C34	122.3(10)
C42	C39	C41	C38	3.7(10)	C42	C39	C41	C42	-0.0(5)
C34	C41	C42	C37	-32.0(14)	C34	C41	C42	C38	-79.2(11)
C34	C41	C42	C39	-83.9(12)	C34	C41	C42	C43	-6.9(17)
C38	C41	C42	C37	47.2(9)	C38	C41	C42	C38	0.0(7)
C38	C41	C42	C39	-4.7(12)	C38	C41	C42	C43	72.3(12)
C39	C41	C42	C37	51.9(14)	C39	C41	C42	C38	4.7(13)
C39	C41	C42	C39	0.0(9)	C39	C41	C42	C43	77.0(16)
C37	C42	C43	C36	69.7(16)	C37	C42	C43	C37	-0.0(12)
C37	C42	C43	C38	-4.6(16)	C38	C42	C43	C36	74.3(14)
C38	C42	C43	C37	4.6(12)	C38	C42	C43	C38	-0.0(7)
C39	C42	C43	C36	36.7(15)	C39	C42	C43	C37	-33.0(13)
C39	C42	C43	C38	-37.6(6)	C41	C42	C43	C36	8.6(18)
C41	C42	C43	C37	-61.0(14)	C41	C42	C43	C38	-65.7(10)

Symmetry Operators:

(1) -X+1,-Y+1,-Z+1

Supplementary References

(1) Suginome, M.; Oike, H.; Park, S. -S.; Ito, Y. Reactions of Si–Si σ-Bonds with Bis(t-alkyl isocyanide)palladium(0) Complexes. Synthesis and Reactions of Cyclic Bis(organosilyl)palladium Complexex, *Bull. Chem. Soc. Jpn.,* **69**, 289-299 (1996).

(2) Tacke, R.; Heemann, J.; Penka, M.; Richter, I.; Wagner, B. Z. Improved Synthesis of $HOPh_2Si\text{-}SiPh_2OH$ and Crystal Structure Analyses of $HOPh_2Si\text{-}SiPh_2OH$ and $HOPh_2Si\text{-}SiPh_2\text{-}O\text{-}Ph_2Si\text{-}SiPh_2OH{\cdot}1/2C_6H_6$, *Naturforsch*., **57b**, 731-735 (2002).

(3) Ansell, M. B.; Roberts, D. E.; Cloke, F. G. N.; Navarro, O.; Spencer, J. Synthesis of an $[(NHC)_2Pd(SiMe_3)_2]$ Complex and Catalytic *cis*-Bis(silyl)ations of Alkynes with Unactivated Disilanes, *Angew. Chem. Int. Ed.* **54**, 5578-5582 (2015).

(4) Frisch, M. J.; Trucks, G. W.; Schlegel, H. B.; Scuseria, G. E.; Robb, M. A.; Cheeseman, J. R.; Scalmani, G.; Barone, V.; Mennucci, B.; Petersson, G. A.; Nakatsuji, H.; Caricato, M.; Li, X.; Hratchian, H. P.; Izmaylov, A. F.; Bloino, J.; Zheng, G.; Sonnenberg, J. L.; Hada, M.; Ehara, M.; Toyota, K.; Fukuda, R.; Hasegawa, J.; Ishida, M.; Nakajima, T.; Honda, Y.; Kitao, O.; Nakai, H.; Vreven, T.; Montgomery, J. A. Jr.; Peralta, J. E.; Ogliaro, F.; Bearpark, M.; Heyd, J. J.; Brothers, E.; Kudin, K. N.; Staroverov, V. N.; Keith, T.; Kobayashi, R.; Normand, J.; Raghavachari, K;. Rendell, A.; Burant, J. C.; Iyengar, S. S.; Tomasi, J.; Cossi, M.; Rega, N.; Millam, J. M.; Klene, M.; Knox, J. E.; Cross, J. B.; Bakken, V.; Adamo, C.; Jaramillo, J;. Gomperts, R.; Stratmann, R. E.; Yazyev, O.; Austin, A. J.; Cammi, R.; Pomelli, C.; Ochterski, J. W.; Martin, R. L.; Morokuma, K.; Zakrzewski, V. G.; Voth, G. A.; Salvador, P.; Dannenberg, J. J.; Dapprich, S.; Daniels, A. D.; Farkas, O.; Foresman, J. B.; Ortiz, J. V.; Cioslowski, J.; Fox, D. J. *Gaussian 09, Revision C.01;* Gaussian, Inc., Wallingford, CT, 2010.

(5) Perdew, J. P.; Chevary, J. A.; Vosko, S. H.; Jackson, K. A.; Pederson, M. R.; Singh, D. J.; Fiolhais, C. Erratum: Atoms, molecules, solids, and surfaces: Applications of the generalized gradient approximation for exchange and correlation, *Phys. Rev. B,* **48**, 4978 (1993).

(6) Andrae, D.; Häußermann, U.; Dolg, M.; Stoll, H.; Preuß, H. Energy-adjusted *ab initio* pseudopotentials for the second and third row transition elements, *Theor. Chim. Acta,* 77, 123-14 (1990).

(7) (a) Gordon, M. S. The isomers of silacyclopropane, *Chem. Phys. Lett.,* **76**, 163−168 (1980); (b) Hariharan P. C.; Pople, J. A. Accuracy of AH*n* equilibrium geometries by single determinant molecular orbital theory, *Mol. Phys.,* **27**, 209−214 (1974); (c) Hariharan P. C.; Pople, J. A. The influence of polarization functions on molecular orbital hydrogenation energies, *Theor. Chem. Acc.,* **28**, 213−222 (1973); (d) Hehre, W. J.; Ditchfield, R.; Pople, J. A. Self-Consistent Molecular Orbital Methods. XII. Further Extensions of Gaussian-Type Basis Sets for Use in Molecular Orbital Studies of Organic Molecules, *J. Chem. Phys.,* **56**, 2257−2261 (1972); (e) Ditchfield, R.; Hehre W. J.; Pople, J. A. Self-Consistent Molecular-Orbital Methods. IX. An Extended Gaussian-Type Basis for Molecular-Orbital Studies of Organic Molecules, *J. Chem. Phys.,* **54**, 724−728 (1971).

(8) Pribanic, B.; Trincado, M.; Eiler, F.; Vogt, M.; Comas-Vives, A.; Grützmacher, H. Hydrogenolysis of Polysilanes Catalyzed by Low-Valent Nickel Complexes, *Angew. Chem. Int. Ed.* **59**, 15603-15609 (2020).

(9) Korshak, V. V.; Ovchinnikov, Yu. E.; Dement'ev, V. V.; Shklover, V. E.; Struchkov, Yu. T.; Frunze, T. M.; *Dokl. Akad. Nauk SSSR*. **293**, 140-143 (1987).

(10) SIR2008: Burla, M. C.; Caliandro, R.; Camalli, M.; Carrozzini, B.; Cascarano, G. L.; De Caro, L.; Giacovazzo, C.; Polidori, G.; Siliqi, D.; Spagna, R. *IL MILIONE*: a suite of computer programs for crystal structure solution of proteins, *J. Appl. Cryst.,* **40**, 609-613 (2007).

(11) International Tables for Crystallography, Vol. C; (Ed. Wilson, A. J. C.) Kluwer Academic Publishers, Dordrecht, Netherlands, Table 6.1.1.4, 1992, pp. 572.

(12) Ibers, J. A.; Hamilton, W. C. Dispersion corrections and crystal structure refinements, *Acta Cryst*., **17**, 781-782 (1964).

(13) Creagh, D. C.; McAuley, W. J. in *International Tables for Crystallography, Vol C*; (Ed. Wilson, A. J. C.), Kluwer Academic Publishers, Dordrecht, Netherlands, Table 4.2.6.8, 1992, pages 219-222.

(14) Creagh, D. C.; Hubbell, J. H. in *International Tables for Crystallography*, *Vol C*; (Ed. Wilson, A. J. C.), Kluwer Academic Publishers, Boston, Table 4.2.4.3, 1992, pages 200-206.

(15) CrystalStructure 4.2.5: Crystal Structure Analysis Package, Rigaku Corporation (2000-2017). Tokyo 196-8666, Japan.

(16) SHELXL Version 2017/1: Sheldrick, G. M. A short history of SHELX, *Acta Cryst*., **A64**, 112-122 (2008).
