# Supplementary material for: Dinuclear and tetranuclear group 10 metal complexes constructed from linear tetrasilane comprising both Si-H and Si-Si moieties
Source: Commun Chem. 2023 May 15;6:93. doi: 10.1038/s42004-023-00892-8 (PMC10185686; doi:10.1038/s42004-023-00892-8)
Supplement: Supplementary file 2 — Description of Additional Supplementary Files [file 42004_2023_892_MOESM2_ESM.pdf]

Description of Additional Supplementary Files

File Name: Supplementary Data 1
Description: crystallographic information file (cif) of **2**.

File Name: Supplementary Data 2
Description: cif check of **2**.

File Name: Supplementary Data 3
Description: crystallographic information file (cif) of **3**.

File Name: Supplementary Data 4
Description: cif check of **3**.

File Name: Supplementary Data 5
Description: crystallographic information file (cif) of **4**.

File Name: Supplementary Data 6
Description: cif check of **4**.

File Name: Supplementary Data 7
Description: crystallographic information file (cif) of **5a**.

File Name: Supplementary Data 8
Description: cif check of **5a**.

File Name: Supplementary Data 9
Description: crystallographic information file (cif) of **5b**.

File Name: Supplementary Data 10
Description: cif check of **5b**.

File Name: Supplementary Data 11
Description: crystallographic information file (cif) of **6**.

File Name: Supplementary Data 12
Description: cif check of **6**.

File Name: Supplementary Data 13
Description: crystallographic information file (cif) of **7**.

File Name: Supplementary Data 14

Description: cif check of **7**.

File Name: Supplementary Data 15

Description: crystallographic information file (cif) of **8**.

File Name: Supplementary Data 16

Description: cif check of **8**.

File Name: Supplementary Data 17

Description: atomic coordinates of $\mathbf{1a}_{\mathbf{opt}}$.

File Name: Supplementary Data 18

Description: atomic coordinates of $\mathbf{1b}_{\mathbf{opt}}$.

File Name: Supplementary Data 19

Description: atomic coordinates of $\mathbf{2}_{\mathbf{opt}}$.

File Name: Supplementary Data 20

Description: atomic coordinates of $\mathbf{5a}_{\mathbf{opt}}$.

File Name: Supplementary Data 21

Description: atomic coordinates of $\mathbf{5b}_{\mathbf{opt}}$.

File Name: Supplementary Data 22

Description: atomic coordinates of $\mathbf{6}_{\mathbf{opt}}$.

File Name: Supplementary Data 23

Description: atomic coordinates of $\mathbf{7}_{\mathbf{opt}}$.

File Name: Supplementary Data 24

Description: atomic coordinates of $\mathbf{8}_{\mathbf{opt}}$.
