# Supplementary material for: Dinuclear and tetranuclear group 10 metal complexes constructed from linear tetrasilane comprising both Si-H and Si-Si moieties
Source: Commun Chem. 2023 May 15;6:93. doi: 10.1038/s42004-023-00892-8 (PMC10185686; doi:10.1038/s42004-023-00892-8)
Supplement: Supplementary file 4 — Supplementary Data 2 [file 42004_2023_892_MOESM4_ESM.pdf]

checkCIF (basic structural check) running

Checking for embedded fcf data in CIF ...
Found embedded fcf data in CIF. Extracting fcf data from uploaded CIF, please wait . . . . .

checkCIF/PLATON (basic structural check)

Structure factors have been supplied for datablock(s) PtSi4

THIS REPORT IS FOR GUIDANCE ONLY. IF USED AS PART OF A REVIEW PROCEDURE FOR PUBLICATION, IT SHOULD NOT REPLACE THE EXPERTISE OF AN EXPERIENCED CRYSTALLOGRAPHIC REFEREE.

No syntax errors found. CIF dictionary
Please wait while processing .... Interpreting this report

Structure factor report

Datablock: PtSi4

Bond precision:	C-C = 0.0060 A		Wavelength=0.71075
Cell:	a=14.903(6)	b=15.292(6)	c=23.077(9)
	alpha=90	beta=90	gamma=90
Temperature:	113 K		

	Calculated	Reported
Volume	5259(4)	5259(4)
Space group	P b c n	P b c n
Hall group	-P 2n 2ab	-P 2n 2ab
Moiety formula	C58 H58 N2 Pt Si4 [+ solvent]	C58 H58 N2 Pt Si4
Sum formula	C58 H58 N2 Pt Si4 [+ solvent]	C58 H58 N2 Pt Si4
Mr	1090.50	1090.54
Dx,g cm-3	1.377	1.377
Z	4	4
Mu (mm-1)	2.798	2.787
F000	2216.0	2216.0
F000'	2211.13	
h,k,lmax	19,20,30	19,19,29
Nref	6455	6025
Tmin,Tmax	0.846,0.946	0.807,0.946
Tmin'	0.658	

Correction method= # Reported T Limits: Tmin=0.807 Tmax=0.946 AbsCorr = MULTI-SCAN

Data completeness= 0.933 Theta(max)= 28.153

R(reflections)= 0.0429(4578) wR2(reflections)= 0.0933(6025)

S = 1.133 Npar= 297

The following ALERTS were generated. Each ALERT has the format
test-name_ALERT_alert-type_alert-level.
Click on the hyperlinks for more details of the test.

Alert level C

PLAT906_ALERT_3_C Large K Value in the Analysis of Variance ...... 15.573 Check
PLAT906_ALERT_3_C Large K Value in the Analysis of Variance ...... 3.149 Check
PLAT910_ALERT_3_C Missing # of FCF Reflection(s) Below Theta(Min). 8 Note
PLAT911_ALERT_3_C Missing FCF Refl Between Thmin & STh/L= 0.600 2 Report

Alert level G

CHEMS02_ALERT_1_G Please check that you have entered the correct
_publ_requested_category classification of your compound;
FI or CI or EI for inorganic; FM or CM or EM for metal-organic;
FO or CO or EO for organic.
From the CIF: _publ_requested_category CHOOSE FI FM FO CI CM CO or A
From the CIF: _chemical_formula_sum :C58 H58 N2 Pt1 Si4
PLAT232_ALERT_2_G Hirshfeld Test Diff (M-X) Pt1 --Si1 . 6.4 s.u.
PLAT232_ALERT_2_G Hirshfeld Test Diff (M-X) Pt1 --C1 . 5.5 s.u.
PLAT605_ALERT_4_G Largest Solvent Accessible VOID in the Structure 41 A**3
PLAT793_ALERT_4_G Model has Chirality at Si1 (Centro SPGR) R Verify
PLAT793_ALERT_4_G Model has Chirality at Si2 (Centro SPGR) S Verify
PLAT869_ALERT_4_G ALERTS Related to the Use of SQUEEZE Suppressed ! Info
PLAT912_ALERT_4_G Missing # of FCF Reflections Above STh/L= 0.600 386 Note
PLAT978_ALERT_2_G Number C-C Bonds with Positive Residual Density. 3 Info

0 **ALERT level A** = Most likely a serious problem - resolve or explain
0 **ALERT level B** = A potentially serious problem, consider carefully
4 **ALERT level C** = Check. Ensure it is not caused by an omission or oversight
9 **ALERT level G** = General information/check it is not something unexpected

1 ALERT type 1 CIF construction/syntax error, inconsistent or missing data
3 ALERT type 2 Indicator that the structure model may be wrong or deficient
4 ALERT type 3 Indicator that the structure quality may be low
5 ALERT type 4 Improvement, methodology, query or suggestion
0 ALERT type 5 Informative message, check

It is advisable to attempt to resolve as many as possible of the alerts in all categories. Often the minor alerts point to easily fixed oversights, errors and omissions in your CIF or refinement strategy, so attention to these fine details can be worthwhile. In order to resolve some of the more serious problems it may be necessary to carry out additional measurements or structure refinements. However, the purpose of your study may justify the reported deviations and the more serious of these should normally be commented upon in the discussion or experimental section of a paper or in the "special_details" fields of the CIF. checkCIF was carefully designed to identify outliers and unusual parameters, but every test has its limitations and alerts that are not important in a particular case may appear. Conversely, the absence of alerts does not guarantee there are no aspects of the results needing attention. It is up to the individual to critically assess their own results and, if necessary, seek expert advice.

Publication of your CIF in IUCr journals

A basic structural check has been run on your CIF. These basic checks will be run on all CIFs submitted for publication in IUCr journals (*Acta Crystallographica*, *Journal of Applied Crystallography*, *Journal of Synchrotron Radiation*); however, if you intend to submit to *Acta Crystallographica Section C* or *E* or *IUCrData*, you should make sure that full publication checks are run on the final version of your CIF prior to submission.

Publication of your CIF in other journals

Please refer to the *Notes for Authors* of the relevant journal for any special instructions relating to CIF submission.

PLATON version of 18/05/2022; check.def file version of 17/05/2022

Datablock PtSi4 - ellipsoid plot

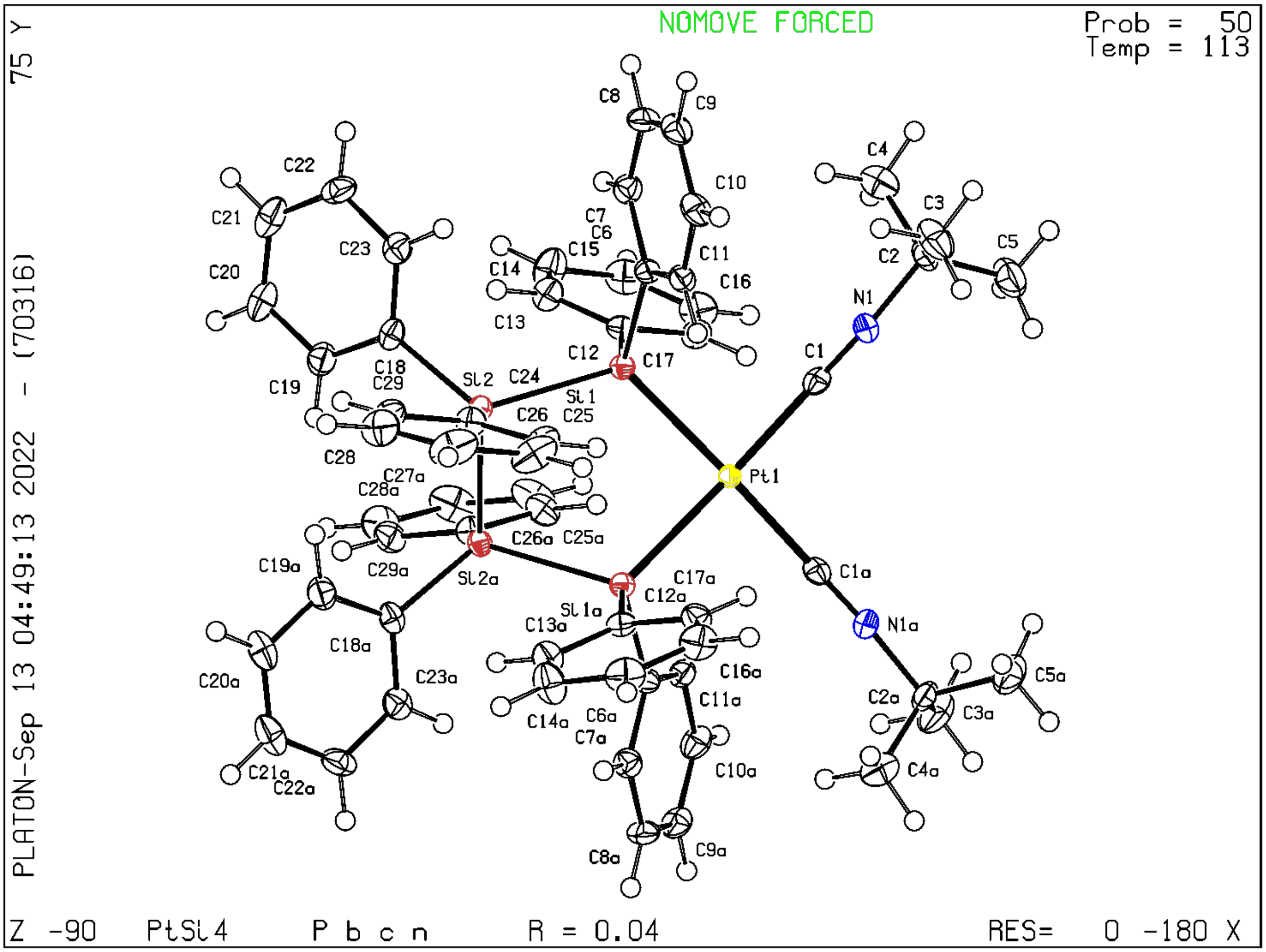

Download CIF editor (publCIF) from the IUCr
Download CIF editor (enCIFer) from the CCDC
Test a new CIF entry
