# Supplementary material for: Dinuclear and tetranuclear group 10 metal complexes constructed from linear tetrasilane comprising both Si-H and Si-Si moieties
Source: Commun Chem. 2023 May 15;6:93. doi: 10.1038/s42004-023-00892-8 (PMC10185686; doi:10.1038/s42004-023-00892-8)
Supplement: Supplementary file 6 — Supplementary Data 4 [file 42004_2023_892_MOESM6_ESM.pdf]

checkCIF (basic structural check) running

Checking for embedded fcf data in CIF ...
Found embedded fcf data in CIF. Extracting fcf data from uploaded CIF, please wait . . . . .

checkCIF/PLATON (basic structural check)

Structure factors have been supplied for datablock(s) Pt-HNHCSi4

THIS REPORT IS FOR GUIDANCE ONLY. IF USED AS PART OF A REVIEW PROCEDURE FOR PUBLICATION, IT SHOULD NOT REPLACE THE EXPERTISE OF AN EXPERIENCED CRYSTALLOGRAPHIC REFEREE.

No syntax errors found. CIF dictionary
Please wait while processing .... Interpreting this report

Structure factor report

Datablock: Pt-HNHCSi4

Bond precision:	C-C = 0.0123 A		Wavelength=0.71075
Cell:	a=13.283(3)	b=17.520(3)	c=18.741(3)
	alpha=61.917(8)	beta=69.067(10)	gamma=81.320(11)
Temperature:	123 K		

	Calculated	Reported
Volume	3593.1(12)	3593.0(12)
Space group	P -1	P -1
Hall group	-P 1	-P 1
Moiety formula	C70 H82 N4 Pt Si4 [+ solvent]	C60 H13 N4 Pt Si4
Sum formula	C70 H82 N4 Pt Si4 [+ solvent]	C60 H13 N4 Pt Si4
Mr	1286.84	1097.22
Dx,g cm-3	1.189	1.014
Z	2	2
Mu (mm-1)	2.058	2.041
F000	1328.0	1070.0
F000'	1325.60	
h,k,lmax	17,22,24	17,22,24
Nref	16532	15856
Tmin,Tmax	0.952,0.960	0.789,0.960
Tmin'	0.849	

Correction method= # Reported T Limits: Tmin=0.789 Tmax=0.960 AbsCorr = MULTI-SCAN

Data completeness= 0.959 Theta(max)= 27.497

R(reflections)= 0.0579(12714) wR2(reflections)= 0.1625(15856)

S = 1.045 Npar= 732

The following ALERTS were generated. Each ALERT has the format
test-name_ALERT_alert-type_alert-level.
Click on the hyperlinks for more details of the test.

Alert level B

PLAT242_ALERT_2_B Low 'MainMol' Ueq as Compared to Neighbors of C15 Check
PLAT242_ALERT_2_B Low 'MainMol' Ueq as Compared to Neighbors of C62 Check
PLAT910_ALERT_3_B Missing # of FCF Reflection(s) Below Theta(Min). 22 Note
PLAT972_ALERT_2_B Check Calcd Resid. Dens. 0.68Ang From Pt1 -2.92 eA-3
PLAT972_ALERT_2_B Check Calcd Resid. Dens. 0.83Ang From Pt1 -2.63 eA-3
PLAT973_ALERT_2_B Check Calcd Positive Resid. Density on Pt1 1.66 eA-3

Alert level C

PLAT155_ALERT_4_C The Triclinic Unitcell is NOT Reduced .... Please Do !
PLAT213_ALERT_2_C Atom C17 has ADP max/min Ratio ..... 3.4 prolat
PLAT213_ALERT_2_C Atom C64 has ADP max/min Ratio ..... 3.3 prolat
PLAT220_ALERT_2_C NonSolvent Resd 1 C Ueq(max)/Ueq(min) Range 5.8 Ratio
PLAT222_ALERT_3_C NonSolvent Resd 1 H Uiso(max)/Uiso(min) Range 10.0 Ratio
PLAT234_ALERT_4_C Large Hirshfeld Difference C15 --C16 . 0.20 Ang.
And 3 other PLAT234 Alerts
Less ...

PLAT234_ALERT_4_C Large Hirshfeld Difference C61 --C62 . 0.24 Ang.
PLAT234_ALERT_4_C Large Hirshfeld Difference C62 --C63 . 0.21 Ang.
PLAT234_ALERT_4_C Large Hirshfeld Difference C67 --C68 . 0.18 Ang.

PLAT241_ALERT_2_C High 'MainMol' Ueq as Compared to Neighbors of C61 Check
And 3 other PLAT241 Alerts
More ...
PLAT242_ALERT_2_C Low 'MainMol' Ueq as Compared to Neighbors of C59 Check
PLAT245_ALERT_2_C U(iso) H1 Smaller than U(eq) Pt1 by 0.019 Ang**2
PLAT332_ALERT_2_C Large Phenyl C-C Range C59 -C64 . 0.23 Ang.
PLAT342_ALERT_3_C Low Bond Precision on C-C Bonds .... 0.01232 Ang.
PLAT420_ALERT_2_C D-H Bond Without Acceptor Si4 --H2 . Please Check
PLAT911_ALERT_3_C Missing FCF Refl Between Thmin & STh/L= 0.600 224 Report
PLAT971_ALERT_2_C Check Calcd Resid. Dens. 0.86Ang From C6 2.14 eA-3
And 2 other PLAT971 Alerts
Less ...

PLAT971_ALERT_2_C Check Calcd Resid. Dens. 1.04Ang From Pt1 1.55 eA-3
PLAT971_ALERT_2_C Check Calcd Resid. Dens. 1.35Ang From Pt1 1.53 eA-3

PLAT972_ALERT_2_C Check Calcd Resid. Dens. 0.85Ang From Pt1 -1.69 eA-3
And 3 other PLAT972 Alerts
Less ...

PLAT972_ALERT_2_C Check Calcd Resid. Dens. 0.77Ang From Pt1 -1.64 eA-3
PLAT972_ALERT_2_C Check Calcd Resid. Dens. 0.81Ang From Pt1 -1.60 eA-3
PLAT972_ALERT_2_C Check Calcd Resid. Dens. 0.73Ang From Pt1 -1.59 eA-3

Alert level G

FORMU01_ALERT_2_G There is a discrepancy between the atom counts in the
_chemical_formula_sum and the formula from the _atom_site* data.
Atom count from _chemical_formula_sum:C60 H13 N4 Pt1 Si4
Atom count from the _atom_site data: C70 H82 N4 Pt1 Si4
CELLZ01_ALERT_1_G Difference between formula and atom_site contents detected.
CELLZ01_ALERT_1_G ALERT: Large difference may be due to a
symmetry error - see SYMMG tests
From the CIF: _cell_formula_units_Z 2
From the CIF: _chemical_formula_sum C60 H13 N4 Pt Si4
TEST: Compare cell contents of formula and atom_site data

atom	Z*formula	cif sites	diff
C	120.00	140.00	-20.00
H	26.00	164.00	-138.00
N	8.00	8.00	0.00
Pt	2.00	2.00	0.00
Si	8.00	8.00	0.00

CHEMS02_ALERT_1_G Please check that you have entered the correct

_publ_requested_category classification of your compound;
FI or CI or EI for inorganic; FM or CM or EM for metal-organic;
FO or CO or EO for organic.
From the CIF: _publ_requested_category CHOOSE FI FM FO CI CM CO or A
From the CIF: _chemical_formula_sum :C60 H13 N4 Pt1 Si4

PLAT041_ALERT_1_G Calc. and Reported SumFormula Strings Differ Please Check
PLAT042_ALERT_1_G Calc. and Reported MoietyFormula Strings Differ Please Check
PLAT232_ALERT_2_G Hirshfeld Test Diff (M-X) Pt1 --Si1 . 5.3 s.u.
PLAT606_ALERT_4_G Solvent Accessible VOID(S) in Structure .... ! Info
PLAT793_ALERT_4_G Model has Chirality at Si4 (Centro SPGR) S Verify
PLAT869_ALERT_4_G ALERTS Related to the Use of SQUEEZE Suppressed ! Info
PLAT912_ALERT_4_G Missing # of FCF Reflections Above STh/L= 0.600 430 Note
PLAT933_ALERT_2_G Number of HKL-OMIT Records in Embedded .res File 12 Note
PLAT941_ALERT_3_G Average HKL Measurement Multiplicity .... 1.9 Low
PLAT978_ALERT_2_G Number C-C Bonds with Positive Residual Density. 0 Info

---

0 **ALERT level A** = Most likely a serious problem - resolve or explain
6 **ALERT level B** = A potentially serious problem, consider carefully
26 **ALERT level C** = Check. Ensure it is not caused by an omission or oversight
14 **ALERT level G** = General information/check it is not something unexpected

5 ALERT type 1 CIF construction/syntax error, inconsistent or missing data
27 ALERT type 2 Indicator that the structure model may be wrong or deficient
5 ALERT type 3 Indicator that the structure quality may be low
9 ALERT type 4 Improvement, methodology, query or suggestion
0 ALERT type 5 Informative message, check

It is advisable to attempt to resolve as many as possible of the alerts in all categories. Often the minor alerts point to easily fixed oversights, errors and omissions in your CIF or refinement strategy, so attention to these fine details can be worthwhile. In order to resolve some of the more serious problems it may be necessary to carry out additional measurements or structure refinements. However, the purpose of your study may justify the reported deviations and the more serious of these should normally be commented upon in the discussion or experimental section of a paper or in the "special_details" fields of the CIF. checkCIF was carefully designed to identify outliers and unusual parameters, but every test has its limitations and alerts that are not important in a particular case may appear. Conversely, the absence of alerts does not guarantee there are no aspects of the results needing attention. It is up to the individual to critically assess their own results and, if necessary, seek expert advice.

Publication of your CIF in IUCr journals

A basic structural check has been run on your CIF. These basic checks will be run on all CIFs submitted for publication in IUCr journals (*Acta Crystallographica*, *Journal of Applied Crystallography*, *Journal of Synchrotron Radiation*); however, if you intend to submit to *Acta Crystallographica Section C* or *E* or *IUCrData*, you should make sure that full publication checks are run on the final version of your CIF prior to submission.

Publication of your CIF in other journals

Please refer to the *Notes for Authors* of the relevant journal for any special instructions relating to CIF submission.

PLATON version of 18/05/2022; check.def file version of 17/05/2022

Datablock Pt-HNHCSi4 - ellipsoid plot

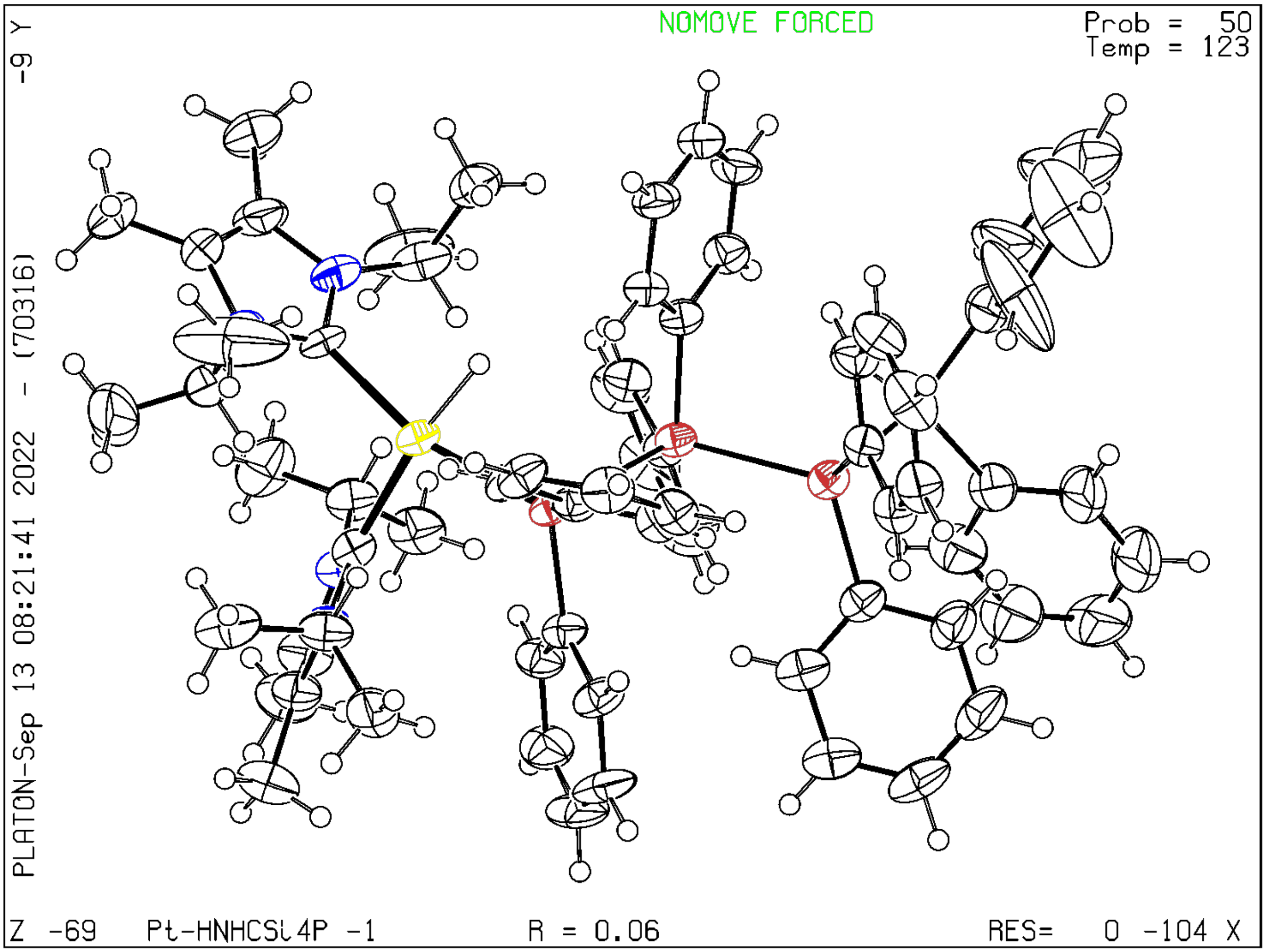

Download CIF editor (publCIF) from the IUCr
Download CIF editor (enCIFer) from the CCDC
Test a new CIF entry
