# Supplementary material for: Dinuclear and tetranuclear group 10 metal complexes constructed from linear tetrasilane comprising both Si-H and Si-Si moieties
Source: Commun Chem. 2023 May 15;6:93. doi: 10.1038/s42004-023-00892-8 (PMC10185686; doi:10.1038/s42004-023-00892-8)
Supplement: Supplementary file 8 — Supplementary Data 6 [file 42004_2023_892_MOESM8_ESM.pdf]

checkCIF (basic structural check) running

Checking for embedded fcf data in CIF ...
Found embedded fcf data in CIF. Extracting fcf data from uploaded CIF, please wait . . . .

checkCIF/PLATON (basic structural check)

Structure factors have been supplied for datablock(s) PtH2NHCSi4

THIS REPORT IS FOR GUIDANCE ONLY. IF USED AS PART OF A REVIEW PROCEDURE FOR PUBLICATION, IT SHOULD NOT REPLACE THE EXPERTISE OF AN EXPERIENCED CRYSTALLOGRAPHIC REFEREE.

No syntax errors found. CIF dictionary
Please wait while processing .... Interpreting this report

Structure factor report

Datablock: PtH2NHCSi4

Bond precision:	C-C = 0.0122 A		Wavelength=0.71075
Cell:	a=12.5743(17)	b=12.631(3)	c=17.132(3)
	alpha=89.617(13)	beta=69.219(10)	gamma=60.968(7)
Temperature:	123 K		

	Calculated	Reported
Volume	2178.3(8)	2178.3(8)
Space group	P -1	P -1
Hall group	-P 1	-P 1
Moiety formula	C92 H120 N8 Pt2 Si4	C92 H122 N8 Pt2 Si4
Sum formula	C92 H120 N8 Pt2 Si4	C92 H122 N8 Pt2 Si4
Mr	1840.48	1842.55
Dx,g cm-3	1.403	1.404
Z	1	1
Mu (mm-1)	3.311	3.298
F000	940.0	942.0
F000'	937.21	
h,k,lmax	16,16,22	16,16,22
Nref	9992	9593
Tmin,Tmax	0.820,0.936	0.748,0.936
Tmin'	0.719	

Correction method= # Reported T Limits: Tmin=0.748 Tmax=0.936 AbsCorr = MULTI-SCAN

Data completeness= 0.960 Theta(max)= 27.473

R(reflections)= 0.0569(7101) wR2(reflections)= 0.1290(9593)

S = 1.018 Npar= 490

The following ALERTS were generated. Each ALERT has the format
test-name_ALERT_alert-type_alert-level.
Click on the hyperlinks for more details of the test.

Alert level B

PLAT910_ALERT_3_B Missing # of FCF Reflection(s) Below Theta(Min). 14 Note
PLAT973_ALERT_2_B Check Calcd Positive Resid. Density on Pt1 1.86 eA-3

Alert level C

PLAT029_ALERT_3_C _diffrn_measured_fraction_theta_full value Low . 0.979 Why?
PLAT041_ALERT_1_C Calc. and Reported SumFormula Strings Differ Please Check

PLAT042_ALERT_1_C Calc. and Reported MoietyFormula Strings Differ Please Check
PLAT043_ALERT_1_C Calculated and Reported Mol. Weight Differ by .. 2.07 Check
PLAT068_ALERT_1_C Reported F000 Differs from Calcd (or Missing)... Please Check
PLAT342_ALERT_3_C Low Bond Precision on C-C Bonds .... 0.01218 Ang.
PLAT911_ALERT_3_C Missing FCF Refl Between Thmin & STh/L= 0.600 155 Report
PLAT971_ALERT_2_C Check Calcd Resid. Dens. 1.28Ang From C4 2.47 eA-3
And 4 other PLAT971 Alerts
Less ...

PLAT971_ALERT_2_C Check Calcd Resid. Dens. 0.98Ang From Pt1 2.21 eA-3
PLAT971_ALERT_2_C Check Calcd Resid. Dens. 0.82Ang From C2 1.63 eA-3
PLAT971_ALERT_2_C Check Calcd Resid. Dens. 1.22Ang From Si2 1.62 eA-3
PLAT971_ALERT_2_C Check Calcd Resid. Dens. 0.87Ang From Pt1 1.57 eA-3

PLAT972_ALERT_2_C Check Calcd Resid. Dens. 0.91Ang From Pt1 -1.77 eA-3
And 4 other PLAT972 Alerts
Less ...

PLAT972_ALERT_2_C Check Calcd Resid. Dens. 0.94Ang From Pt1 -1.68 eA-3
PLAT972_ALERT_2_C Check Calcd Resid. Dens. 0.97Ang From Pt1 -1.66 eA-3
PLAT972_ALERT_2_C Check Calcd Resid. Dens. 0.81Ang From Pt1 -1.58 eA-3
PLAT972_ALERT_2_C Check Calcd Resid. Dens. 0.82Ang From Pt1 -1.51 eA-3

Alert level G

FORMU01_ALERT_2_G There is a discrepancy between the atom counts in the
_chemical_formula_sum and the formula from the _atom_site* data.
Atom count from _chemical_formula_sum:C92 H122 N8 Pt2 Si4
Atom count from the _atom_site data: C92 H120 N8 Pt2 Si4
CELLZ01_ALERT_1_G Difference between formula and atom_site contents detected.
CELLZ01_ALERT_1_G WARNING: H atoms missing from atom site list. Is this intentional?
From the CIF: _cell_formula_units_Z 1
From the CIF: _chemical_formula_sum C92 H122 N8 Pt2 Si4
TEST: Compare cell contents of formula and atom_site data

atom	Z*formula	cif sites	diff
C	92.00	92.00	0.00
H	122.00	120.00	2.00
N	8.00	8.00	0.00
Pt	2.00	2.00	0.00
Si	4.00	4.00	0.00

CHEMS02_ALERT_1_G Please check that you have entered the correct
_publ_requested_category classification of your compound;
FI or CI or EI for inorganic; FM or CM or EM for metal-organic;
FO or CO or EO for organic.
From the CIF: _publ_requested_category CHOOSE FI FM FO CI CM CO or A
From the CIF: _chemical_formula_sum :C92 H122 N8 Pt2 Si4
PLAT912_ALERT_4_G Missing # of FCF Reflections Above STh/L= 0.600 231 Note
PLAT933_ALERT_2_G Number of HKL-OMIT Records in Embedded .res File 10 Note
PLAT941_ALERT_3_G Average HKL Measurement Multiplicity .... 1.9 Low
PLAT978_ALERT_2_G Number C-C Bonds with Positive Residual Density. 0 Info

0 **ALERT level A** = Most likely a serious problem - resolve or explain
2 **ALERT level B** = A potentially serious problem, consider carefully
17 **ALERT level C** = Check. Ensure it is not caused by an omission or oversight
8 **ALERT level G** = General information/check it is not something unexpected

7 ALERT type 1 CIF construction/syntax error, inconsistent or missing data
14 ALERT type 2 Indicator that the structure model may be wrong or deficient
5 ALERT type 3 Indicator that the structure quality may be low
1 ALERT type 4 Improvement, methodology, query or suggestion
0 ALERT type 5 Informative message, check

It is advisable to attempt to resolve as many as possible of the alerts in all categories. Often the minor alerts point to easily fixed oversights, errors and omissions in your CIF or refinement strategy, so attention to these fine details can be worthwhile. In order to resolve some of the more serious problems it may be necessary to carry out additional measurements or structure refinements. However, the purpose of your study may justify the reported deviations and the more serious of these should normally be commented upon in the discussion

or experimental section of a paper or in the "special_details" fields of the CIF. checkCIF was carefully designed to identify outliers and unusual parameters, but every test has its limitations and alerts that are not important in a particular case may appear. Conversely, the absence of alerts does not guarantee there are no aspects of the results needing attention. It is up to the individual to critically assess their own results and, if necessary, seek expert advice.

Publication of your CIF in IUCr journals

A basic structural check has been run on your CIF. These basic checks will be run on all CIFs submitted for publication in IUCr journals (*Acta Crystallographica*, *Journal of Applied Crystallography*, *Journal of Synchrotron Radiation*); however, if you intend to submit to *Acta Crystallographica Section C* or *E* or *IUCrData*, you should make sure that full publication checks are run on the final version of your CIF prior to submission.

Publication of your CIF in other journals

Please refer to the *Notes for Authors* of the relevant journal for any special instructions relating to CIF submission.

PLATON version of 18/05/2022; check.def file version of 17/05/2022

Datablock PtH2NHCSi4 - ellipsoid plot

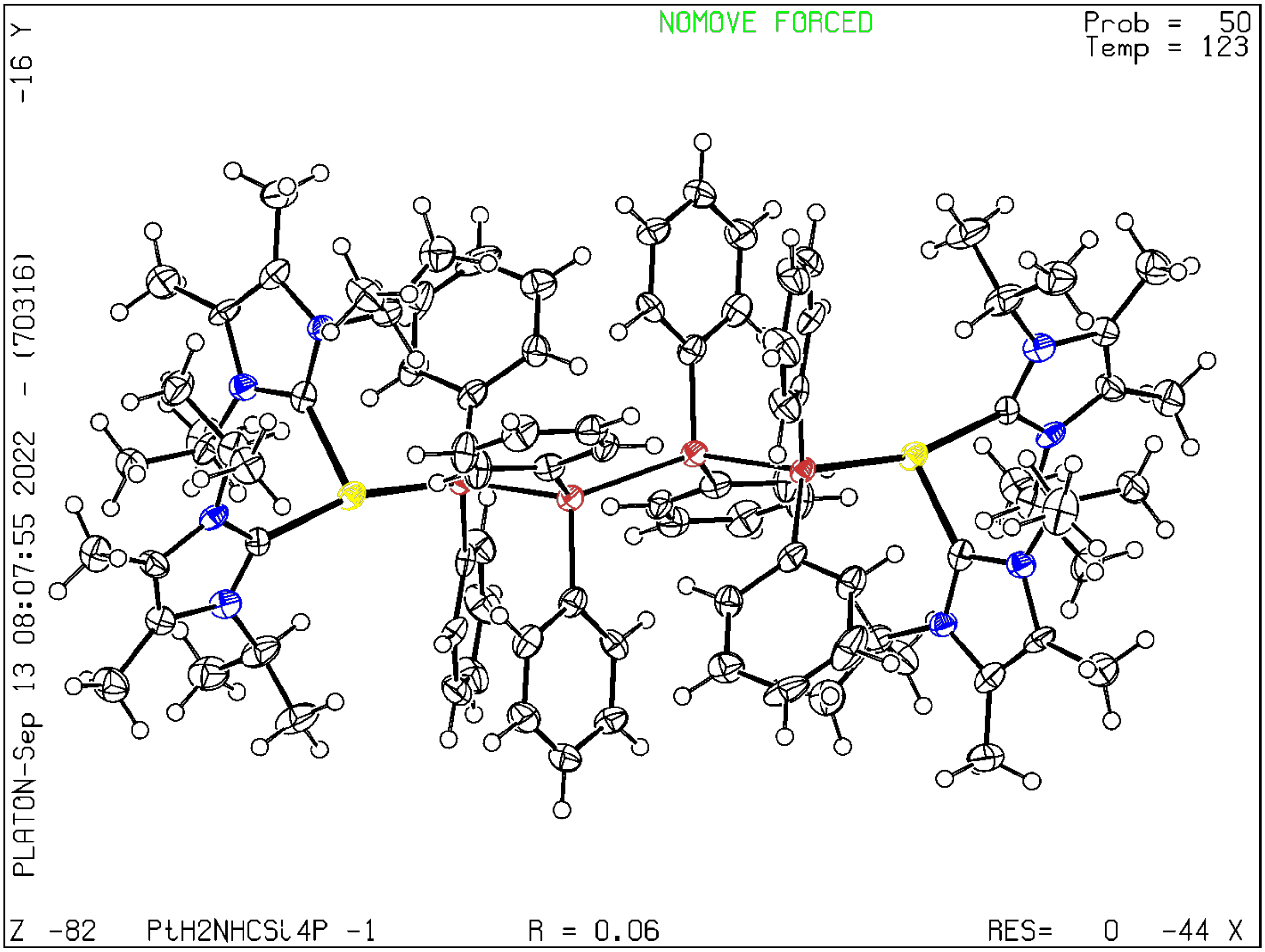

Download CIF editor (publCIF) from the IUCr
Download CIF editor (enCIFer) from the CCDC
Test a new CIF entry
