# Supplementary material for: Dinuclear and tetranuclear group 10 metal complexes constructed from linear tetrasilane comprising both Si-H and Si-Si moieties
Source: Commun Chem. 2023 May 15;6:93. doi: 10.1038/s42004-023-00892-8 (PMC10185686; doi:10.1038/s42004-023-00892-8)
Supplement: Supplementary file 14 — Supplementary Data 12 [file 42004_2023_892_MOESM14_ESM.pdf]

checkCIF (basic structural check) running

Checking for embedded fcf data in CIF ...
Found embedded fcf data in CIF. Extracting fcf data from uploaded CIF, please wait . . . .

checkCIF/PLATON (basic structural check)

Structure factors have been supplied for datablock(s) Pd4SiPh23CNtBu6

THIS REPORT IS FOR GUIDANCE ONLY. IF USED AS PART OF A REVIEW PROCEDURE FOR PUBLICATION, IT SHOULD NOT REPLACE THE EXPERTISE OF AN EXPERIENCED CRYSTALLOGRAPHIC REFEREE.

No syntax errors found. CIF dictionary
Please wait while processing .... Interpreting this report

Structure factor report

Datablock: Pd4SiPh23CNtBu6

Bond precision:	C-C = 0.0045 A		Wavelength=0.71075
Cell:	a=20.19000	b=20.19000	c=26.16590
	alpha=90	beta=90	gamma=120
Temperature:	123 K		

	Calculated	Reported
Volume	9237.170	9237
Space group	P -3 c 1	P -3 c 1
Hall group	-P 3 2"c	-P 3 2"c
Moiety formula	C66 H84 N6 Pd4 Si3 [+ solvent]	C66 H84 N6 Pd4 Si3
Sum formula	C66 H84 N6 Pd4 Si3 [+ solvent]	C66 H84 N6 Pd4 Si3
Mr	1471.26	1471.29
Dx,g cm-3	1.058	1.058
Z	4	4
Mu (mm-1)	0.836	0.836
F000	2992.0	2992.0
F000'	2977.69	
h,k,lmax	29,29,38	28,29,37
Nref	9993	9541
Tmin,Tmax	0.923,0.951	0.819,0.951
Tmin'	0.920	

Correction method= # Reported T Limits: Tmin=0.819 Tmax=0.951 AbsCorr = MULTI-SCAN

Data completeness= 0.955 Theta(max)= 31.187

R(reflections)= 0.0428(6545) wR2(reflections)= 0.0891(9541)

S = 1.012 Npar= 244

The following ALERTS were generated. Each ALERT has the format
test-name_ALERT_alert-type_alert-level.
Click on the hyperlinks for more details of the test.

Alert level C

PLAT094_ALERT_2_C Ratio of Maximum / Minimum Residual Density .... 2.71 Report
PLAT141_ALERT_4_C s.u. on a - Axis Small or Missing .... 0.00000 Ang.
PLAT143_ALERT_4_C s.u. on c - Axis Small or Missing .... 0.00000 Ang.
PLAT151_ALERT_1_C No s.u. (esd) Given on Volume .... Please Do !
PLAT230_ALERT_2_C Hirshfeld Test Diff for C14 --C15 . 6.3 s.u.
PLAT242_ALERT_2_C Low 'MainMol' Ueq as Compared to Neighbors of C7 Check

PLAT911_ALERT_3_C Missing FCF Refl Between Thmin & STh/L= 0.600 58 Report

Alert level G

CHEMS02_ALERT_1_G Please check that you have entered the correct
_publ_requested_category classification of your compound;
FI or CI or EI for inorganic; FM or CM or EM for metal-organic;
FO or CO or EO for organic.
From the CIF: _publ_requested_category CHOOSE FI FM FO CI CM CO or A
From the CIF: _chemical_formula_sum :C66 H84 N6 Pd4 Si3
PLAT232_ALERT_2_G Hirshfeld Test Diff (M-X) Pd1 --C1 . 7.2 s.u.
PLAT232_ALERT_2_G Hirshfeld Test Diff (M-X) Pd1 --C2 . 6.5 s.u.
PLAT606_ALERT_4_G Solvent Accessible VOID(S) in Structure .... ! Info
PLAT869_ALERT_4_G ALERTS Related to the Use of SQUEEZE Suppressed ! Info
PLAT910_ALERT_3_G Missing # of FCF Reflection(s) Below Theta(Min). 2 Note
PLAT912_ALERT_4_G Missing # of FCF Reflections Above STh/L= 0.600 386 Note
PLAT913_ALERT_3_G Missing # of Very Strong Reflections in FCF .... 2 Note
PLAT933_ALERT_2_G Number of HKL-OMIT Records in Embedded .res File 57 Note
PLAT978_ALERT_2_G Number C-C Bonds with Positive Residual Density. 4 Info

0 **ALERT level A** = Most likely a serious problem - resolve or explain
0 **ALERT level B** = A potentially serious problem, consider carefully
7 **ALERT level C** = Check. Ensure it is not caused by an omission or oversight
10 **ALERT level G** = General information/check it is not something unexpected

2 ALERT type 1 CIF construction/syntax error, inconsistent or missing data
7 ALERT type 2 Indicator that the structure model may be wrong or deficient
3 ALERT type 3 Indicator that the structure quality may be low
5 ALERT type 4 Improvement, methodology, query or suggestion
0 ALERT type 5 Informative message, check

It is advisable to attempt to resolve as many as possible of the alerts in all categories. Often the minor alerts point to easily fixed oversights, errors and omissions in your CIF or refinement strategy, so attention to these fine details can be worthwhile. In order to resolve some of the more serious problems it may be necessary to carry out additional measurements or structure refinements. However, the purpose of your study may justify the reported deviations and the more serious of these should normally be commented upon in the discussion or experimental section of a paper or in the "special_details" fields of the CIF. checkCIF was carefully designed to identify outliers and unusual parameters, but every test has its limitations and alerts that are not important in a particular case may appear. Conversely, the absence of alerts does not guarantee there are no aspects of the results needing attention. It is up to the individual to critically assess their own results and, if necessary, seek expert advice.

Publication of your CIF in IUCr journals

A basic structural check has been run on your CIF. These basic checks will be run on all CIFs submitted for publication in IUCr journals (*Acta Crystallographica*, *Journal of Applied Crystallography*, *Journal of Synchrotron Radiation*); however, if you intend to submit to *Acta Crystallographica Section C* or *E* or *IUCrData*, you should make sure that full publication checks are run on the final version of your CIF prior to submission.

Publication of your CIF in other journals

Please refer to the *Notes for Authors* of the relevant journal for any special instructions relating to CIF submission.

PLATON version of 18/05/2022; check.def file version of 17/05/2022

Datablock Pd4SiPh23CNtBu6 - ellipsoid plot

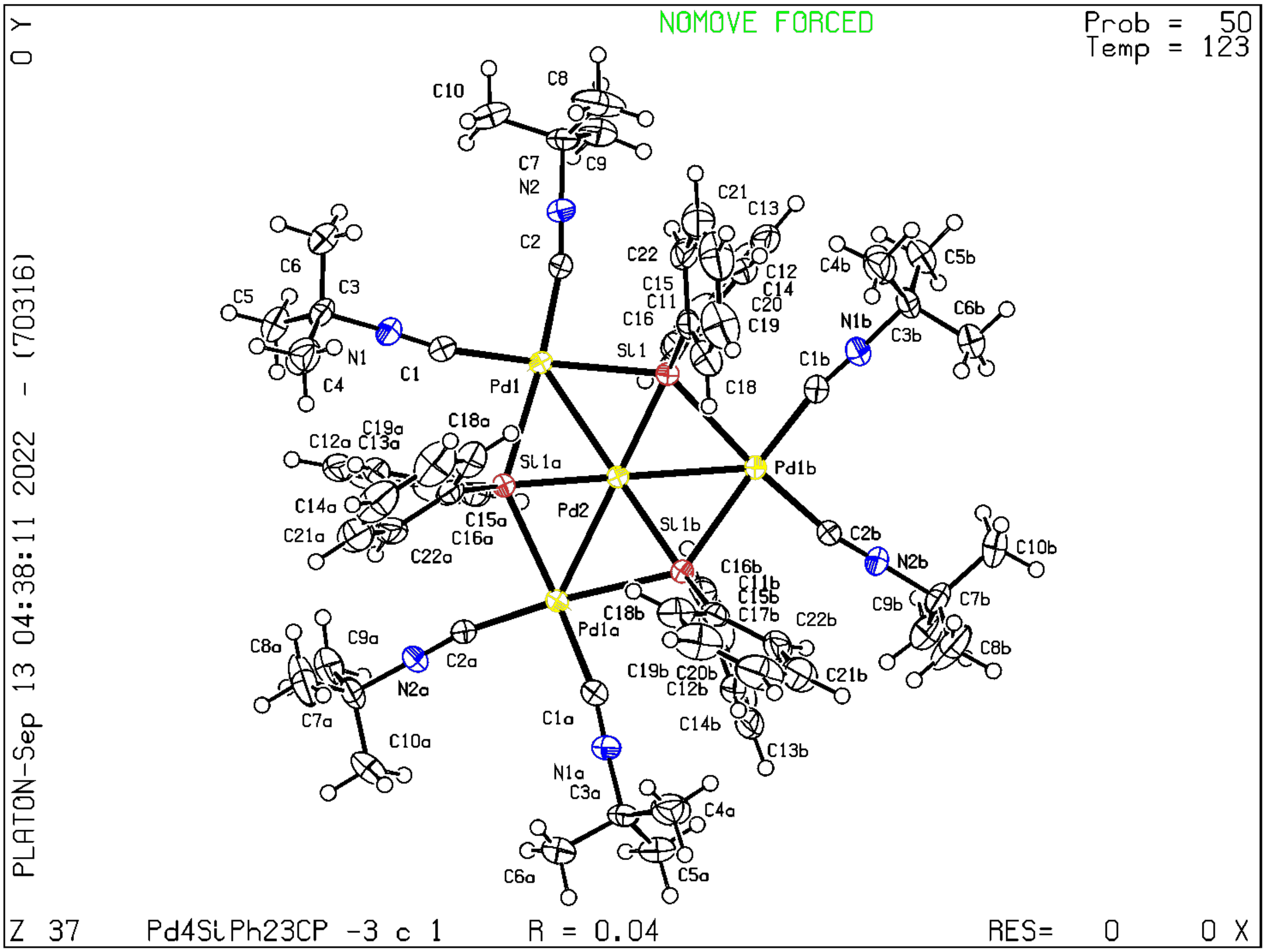

Download CIF editor (publCIF) from the IUCr
Download CIF editor (enCIFer) from the CCDC
Test a new CIF entry
