# Supplementary material for: Dinuclear and tetranuclear group 10 metal complexes constructed from linear tetrasilane comprising both Si-H and Si-Si moieties
Source: Commun Chem. 2023 May 15;6:93. doi: 10.1038/s42004-023-00892-8 (PMC10185686; doi:10.1038/s42004-023-00892-8)
Supplement: Supplementary file 16 — Supplementary Data 14 [file 42004_2023_892_MOESM16_ESM.pdf]

checkCIF (basic structural check) running

Checking for embedded fcf data in CIF ...
Found embedded fcf data in CIF. Extracting fcf data from uploaded CIF, please wait . .

checkCIF/PLATON (basic structural check)

Structure factors have been supplied for datablock(s) NI2NHC

THIS REPORT IS FOR GUIDANCE ONLY. IF USED AS PART OF A REVIEW PROCEDURE FOR PUBLICATION, IT SHOULD NOT REPLACE THE EXPERTISE OF AN EXPERIENCED CRYSTALLOGRAPHIC REFEREE.

No syntax errors found. CIF dictionary
Please wait while processing .... Interpreting this report

Structure factor report

Datablock: NI2NHC

Bond precision:	C-C = 0.0041 A		Wavelength=0.71075
Cell:	a=10.346(4)	b=10.728(4)	c=11.238(4)
	alpha=100.255(3)	beta=95.690(4)	gamma=110.268(5)
Temperature:	123 K		

	Calculated	Reported
Volume	1133.8(7)	1133.8(7)
Space group	P -1	P -1
Hall group	-P 1	-P 1
Moiety formula	C46 H62 N4 Ni2 Si2	C46 H62 N4 Ni2 Si2
Sum formula	C46 H62 N4 Ni2 Si2	C46 H62 N4 Ni2 Si2
Mr	844.56	844.59
Dx,g cm-3	1.237	1.237
Z	1	1
Mu (mm-1)	0.918	0.918
F000	450.0	450.0
F000'	450.93	
h,k,lmax	13,13,14	13,13,14
Nref	5211	4993
Tmin,Tmax	0.896,0.912	0.736,0.912
Tmin'	0.871	

Correction method= # Reported T Limits: Tmin=0.736 Tmax=0.912 AbsCorr = MULTI-SCAN

Data completeness= 0.958 Theta(max)= 27.500

R(reflections)= 0.0532(4545) wR2(reflections)= 0.1380(4993)

S = 0.938 Npar= 254

The following ALERTS were generated. Each ALERT has the format
test-name_ALERT_alert-type_alert-level.
Click on the hyperlinks for more details of the test.

Alert level C

RINTA01_ALERT_3_C The value of Rint is greater than 0.12
Rint given 0.121
PLAT029_ALERT_3_C _diffrn_measured_fraction_theta_full value Low . 0.977 Why?
PLAT601_ALERT_2_C Unit Cell Contains Solvent Accessible VOIDS of . 79 Ang**3
PLAT910_ALERT_3_C Missing # of FCF Reflection(s) Below Theta(Min). 7 Note
PLAT911_ALERT_3_C Missing FCF Refl Between Thmin & STh/L= 0.600 89 Report

PLAT913_ALERT_3_C Missing # of Very Strong Reflections in FCF .... 7 Note
PLAT971_ALERT_2_C Check Calcd Resid. Dens. 0.83Ang From Ni1 1.54 eA-3

Alert level G

CHEMS02_ALERT_1_G Please check that you have entered the correct
_publ_requested_category classification of your compound;
FI or CI or EI for inorganic; FM or CM or EM for metal-organic;
FO or CO or EO for organic.
From the CIF: _publ_requested_category CHOOSE FI FM FO CI CM CO or A
From the CIF: _chemical_formula_sum :C46 H62 N4 Ni2 Si2
PLAT020_ALERT_3_G The Value of Rint is Greater Than 0.12 .... 0.121 Report
PLAT232_ALERT_2_G Hirshfeld Test Diff (M-X) Ni1 --C1 . 5.4 s.u.
PLAT303_ALERT_2_G Full Occupancy Atom H1 with # Connections 2.00 Check
PLAT912_ALERT_4_G Missing # of FCF Reflections Above STh/L= 0.600 122 Note
PLAT941_ALERT_3_G Average HKL Measurement Multiplicity .... 1.9 Low
PLAT978_ALERT_2_G Number C-C Bonds with Positive Residual Density. 5 Info

0 **ALERT level A** = Most likely a serious problem - resolve or explain
0 **ALERT level B** = A potentially serious problem, consider carefully
7 **ALERT level C** = Check. Ensure it is not caused by an omission or oversight
7 **ALERT level G** = General information/check it is not something unexpected

1 ALERT type 1 CIF construction/syntax error, inconsistent or missing data
5 ALERT type 2 Indicator that the structure model may be wrong or deficient
7 ALERT type 3 Indicator that the structure quality may be low
1 ALERT type 4 Improvement, methodology, query or suggestion
0 ALERT type 5 Informative message, check

It is advisable to attempt to resolve as many as possible of the alerts in all categories. Often the minor alerts point to easily fixed oversights, errors and omissions in your CIF or refinement strategy, so attention to these fine details can be worthwhile. In order to resolve some of the more serious problems it may be necessary to carry out additional measurements or structure refinements. However, the purpose of your study may justify the reported deviations and the more serious of these should normally be commented upon in the discussion or experimental section of a paper or in the "special_details" fields of the CIF. checkCIF was carefully designed to identify outliers and unusual parameters, but every test has its limitations and alerts that are not important in a particular case may appear. Conversely, the absence of alerts does not guarantee there are no aspects of the results needing attention. It is up to the individual to critically assess their own results and, if necessary, seek expert advice.

Publication of your CIF in IUCr journals

A basic structural check has been run on your CIF. These basic checks will be run on all CIFs submitted for publication in IUCr journals (*Acta Crystallographica*, *Journal of Applied Crystallography*, *Journal of Synchrotron Radiation*); however, if you intend to submit to *Acta Crystallographica Section C* or *E* or *IUCrData*, you should make sure that full publication checks are run on the final version of your CIF prior to submission.

Publication of your CIF in other journals

Please refer to the *Notes for Authors* of the relevant journal for any special instructions relating to CIF submission.

PLATON version of 18/05/2022; check.def file version of 17/05/2022

Datablock NI2NHC - ellipsoid plot

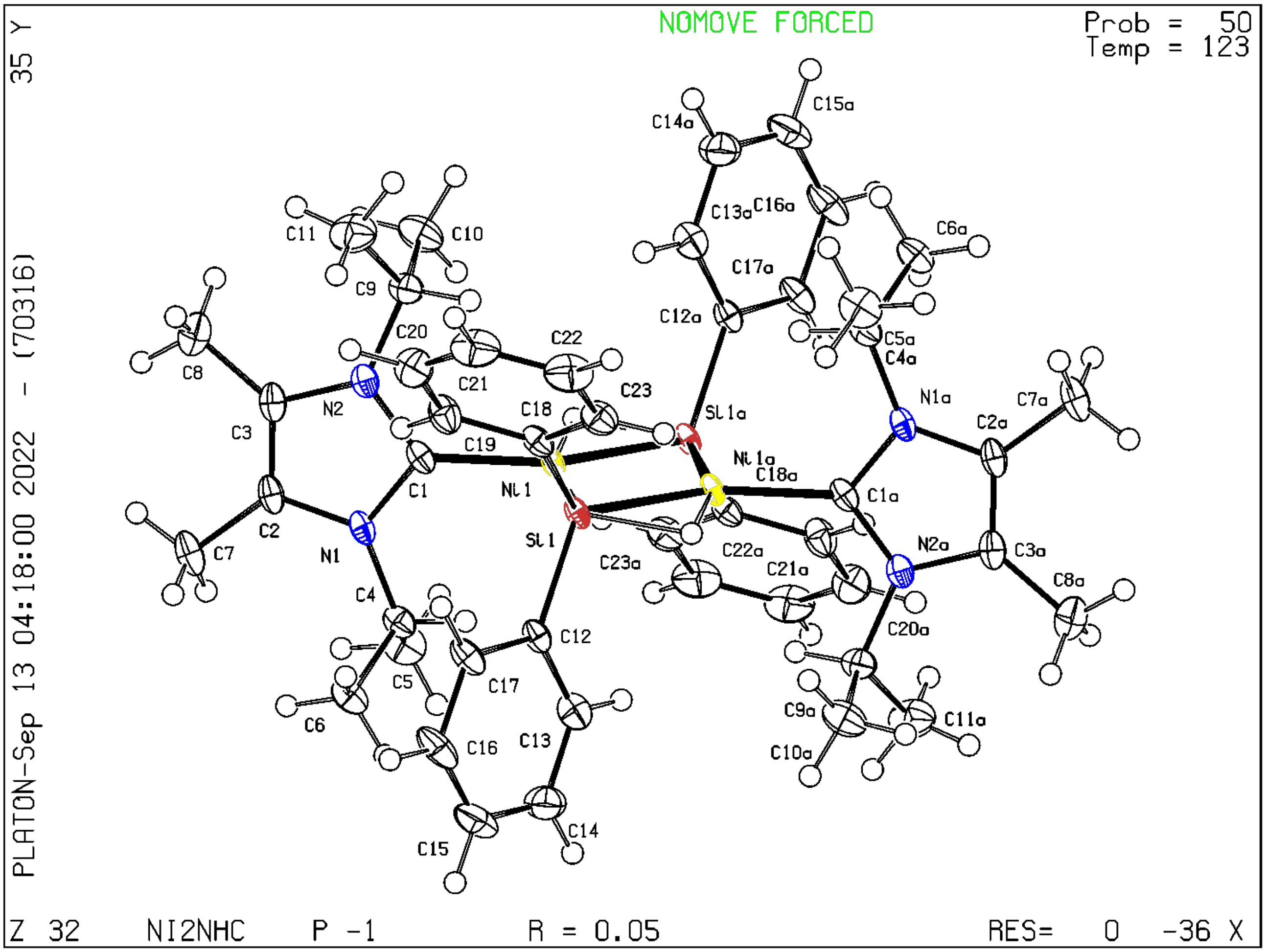

Download CIF editor (publCIF) from the IUCr
Download CIF editor (enCIFer) from the CCDC
Test a new CIF entry
