# Supplementary material for: Dinuclear and tetranuclear group 10 metal complexes constructed from linear tetrasilane comprising both Si-H and Si-Si moieties
Source: Commun Chem. 2023 May 15;6:93. doi: 10.1038/s42004-023-00892-8 (PMC10185686; doi:10.1038/s42004-023-00892-8)
Supplement: Supplementary file 18 — Supplementary Data 16 [file 42004_2023_892_MOESM18_ESM.pdf]

checkCIF (basic structural check) running

Checking for embedded fcf data in CIF ...
Found embedded fcf data in CIF. Extracting fcf data from uploaded CIF, please wait . . . .

checkCIF/PLATON (basic structural check)

Structure factors have been supplied for datablock(s) Pt4Si4Cl2

THIS REPORT IS FOR GUIDANCE ONLY. IF USED AS PART OF A REVIEW PROCEDURE FOR PUBLICATION, IT SHOULD NOT REPLACE THE EXPERTISE OF AN EXPERIENCED CRYSTALLOGRAPHIC REFEREE.

No syntax errors found. CIF dictionary
Please wait while processing .... Interpreting this report

Structure factor report

Datablock: Pt4Si4Cl2

Bond precision:	C-C = 0.0082 A		Wavelength=0.41330
Cell:	a=15.9918(10)	b=12.5337(8)	c=20.6258(12)
	alpha=90	beta=90.331(6)	gamma=90
Temperature:	100 K		

	Calculated	Reported
Volume	4134.1(4)	4134.1(4)
Space group	P 21/n	P 1 21/n 1
Hall group	-P 2yn	-P 2yn
Moiety formula	C78 H84 Cl2 N6 Pt4 Si4	C78 H94 Cl2 N6 Pt4 Si4
Sum formula	C78 H84 Cl2 N6 Pt4 Si4	C78 H94 Cl2 N6 Pt4 Si4
Mr	2069.09	2079.25
Dx,g cm-3	1.662	1.670
Z	2	2
Mu (mm-1)	1.690	0.000
F000	1992.0	2012.0
F000'	1988.22	
h,k,lmax	20,16,26	20,16,26
Nref	9482	9323
Tmin,Tmax		0.825,1.000
Tmin'		

Correction method= # Reported T Limits: Tmin=0.825 Tmax=1.000 AbsCorr = MULTI-SCAN

Data completeness= 0.983 Theta(max)= 15.563

R(reflections)= 0.0259(8951) wR2(reflections)= 0.0650(9323)

S = 1.025 Npar= 430

The following ALERTS were generated. Each ALERT has the format
test-name_ALERT_alert-type_alert-level.
Click on the hyperlinks for more details of the test.

Alert level C

PLAT041_ALERT_1_C Calc. and Reported SumFormula Strings Differ Please Check
PLAT042_ALERT_1_C Calc. and Reported MoietyFormula Strings Differ Please Check
PLAT043_ALERT_1_C Calculated and Reported Mol. Weight Differ by .. 10.16 Check
PLAT213_ALERT_2_C Atom C19 has ADP max/min Ratio ..... 3.7 prolat
PLAT220_ALERT_2_C NonSolvent Resd 1 C Ueq(max)/Ueq(min) Range 5.3 Ratio
PLAT234_ALERT_4_C Large Hirshfeld Difference C12 --C13 . 0.17 Ang.
PLAT234_ALERT_4_C Large Hirshfeld Difference C12 --C14 . 0.16 Ang.
PLAT241_ALERT_2_C High 'MainMol' Ueq as Compared to Neighbors of C18 Check
PLAT242_ALERT_2_C Low 'MainMol' Ueq as Compared to Neighbors of C2 Check
And 2 other PLAT242 Alerts
Less ...

PLAT242_ALERT_2_C Low 'MainMol' Ueq as Compared to Neighbors of C12 Check
PLAT242_ALERT_2_C Low 'MainMol' Ueq as Compared to Neighbors of C16 Check

PLAT329_ALERT_4_C Carbon Atom Hybridisation Unclear for .... C39 Check
PLAT342_ALERT_3_C Low Bond Precision on C-C Bonds .... 0.00821 Ang.
PLAT911_ALERT_3_C Missing FCF Refl Between Thmin & STh/L= 0.600 143 Report
PLAT971_ALERT_2_C Check Calcd Resid. Dens. 1.32Ang From C42 2.12 eA-3
And 3 other PLAT971 Alerts
Less ...

PLAT971_ALERT_2_C Check Calcd Resid. Dens. 0.82Ang From Cl1 2.02 eA-3
PLAT971_ALERT_2_C Check Calcd Resid. Dens. 1.31Ang From C41 1.84 eA-3
PLAT971_ALERT_2_C Check Calcd Resid. Dens. 1.04Ang From C34 1.55 eA-3

PLAT977_ALERT_2_C Check Negative Difference Density on H21 . -0.32 eA-3

Alert level G

FORMU01_ALERT_2_G There is a discrepancy between the atom counts in the
_chemical_formula_sum and the formula from the _atom_site* data.
Atom count from _chemical_formula_sum:C78 H94 Cl2 N6 Pt4 Si4
Atom count from the _atom_site data: C78 H84 Cl2 N6 Pt4 Si4
ABSMU01_ALERT_1_G Calculation of _exptl_absorpt_correction_mu
not performed for this radiation type.
CELLZ01_ALERT_1_G Difference between formula and atom_site contents detected.
CELLZ01_ALERT_1_G WARNING: H atoms missing from atom site list. Is this intentional?
From the CIF: _cell_formula_units_Z 2
From the CIF: _chemical_formula_sum C78 H94 Cl2 N6 Pt4 Si4
TEST: Compare cell contents of formula and atom_site data

atom	Z*formula	cif sites	diff
C	156.00	156.00	0.00
H	188.00	168.00	20.00
Cl	4.00	4.00	0.00
N	12.00	12.00	0.00
Pt	8.00	8.00	0.00
Si	8.00	8.00	0.00

CHEMS02_ALERT_1_G Please check that you have entered the correct
_publ_requested_category classification of your compound;
FI or CI or EI for inorganic; FM or CM or EM for metal-organic;
FO or CO or EO for organic.

From the CIF: _publ_requested_category CHOOSE FI FM FO CI CM CO or A
From the CIF: _chemical_formula_sum :C78 H94 Cl2 N6 Pt4 Si4
PLAT083_ALERT_2_G SHELXL Second Parameter in WGHT Unusually Large 10.87 Why ?
PLAT092_ALERT_4_G Check: Wavelength Given is not Cu,Ga,Mo,Ag,In Ka 0.41330 Ang.
PLAT232_ALERT_2_G Hirshfeld Test Diff (M-X) Pt1 --Si2 . 9.2 s.u.
And 2 other PLAT232 Alerts
More ...
PLAT300_ALERT_4_G Atom Site Occupancy of C37 Constrained at 0.5 Check
And 5 other PLAT300 Alerts
More ...
PLAT301_ALERT_3_G Main Residue Disorder ....(Resd 1) 6% Note
PLAT343_ALERT_2_G Unusual sp? Angle Range in Main Residue for C35 Check
PLAT773_ALERT_2_G Check long C-C Bond in CIF: C39 --C42 1.98 Ang.
PLAT779_ALERT_4_G Suspect or Irrelevant (Bond) Angle(s) in CIF ... 38.00 Deg.
C41 -C34 -C39 1_555 1_555 1_555 ...... # 170 Check
And 9 other PLAT779 Alerts
More ...
PLAT793_ALERT_4_G Model has Chirality at Si1 (Centro SPGR) S Verify
PLAT910_ALERT_3_G Missing # of FCF Reflection(s) Below Theta(Min). 2 Note
PLAT912_ALERT_4_G Missing # of FCF Reflections Above STh/L= 0.600 18 Note
PLAT933_ALERT_2_G Number of HKL-OMIT Records in Embedded .res File 148 Note
PLAT978_ALERT_2_G Number C-C Bonds with Positive Residual Density. 4 Info
PLAT981_ALERT_1_G No non-zero f" Anomalous Scattering Values Found Please Check
PLAT986_ALERT_1_G No non-zero f' Anomalous Scattering Values Found Please Check

---

0 **ALERT level A** = Most likely a serious problem - resolve or explain
0 **ALERT level B** = A potentially serious problem, consider carefully
19 **ALERT level C** = Check. Ensure it is not caused by an omission or oversight
36 **ALERT level G** = General information/check it is not something unexpected

9 ALERT type 1 CIF construction/syntax error, inconsistent or missing data
20 ALERT type 2 Indicator that the structure model may be wrong or deficient
4 ALERT type 3 Indicator that the structure quality may be low
22 ALERT type 4 Improvement, methodology, query or suggestion
0 ALERT type 5 Informative message, check

It is advisable to attempt to resolve as many as possible of the alerts in all categories. Often the minor alerts point to easily fixed oversights, errors and omissions in your CIF or refinement strategy, so attention to these fine details can be worthwhile. In order to resolve some of the more serious problems it may be necessary to carry out additional measurements or structure refinements. However, the purpose of your study may justify the reported deviations and the more serious of these should normally be commented upon in the discussion or experimental section of a paper or in the "special_details" fields of the CIF. checkCIF was carefully designed to identify outliers and unusual parameters, but every test has its limitations and alerts that are not important in a particular case may appear. Conversely, the absence of alerts does not guarantee there are no aspects of the results needing attention. It is up to the individual to critically assess their own results and, if necessary, seek expert advice.

Publication of your CIF in IUCr journals

A basic structural check has been run on your CIF. These basic checks will be run on all CIFs submitted for publication in IUCr journals (*Acta Crystallographica*, *Journal of Applied Crystallography*, *Journal of Synchrotron Radiation*); however, if you intend to submit to *Acta Crystallographica Section C* or *E* or *IUCrData*, you should make sure that full publication checks are run on the final version of your CIF prior to submission.

Publication of your CIF in other journals

Please refer to the *Notes for Authors* of the relevant journal for any special instructions relating to CIF submission.

PLATON version of 12/09/2022; check.def file version of 09/08/2022

Datablock Pt4Si4Cl2 - ellipsoid plot

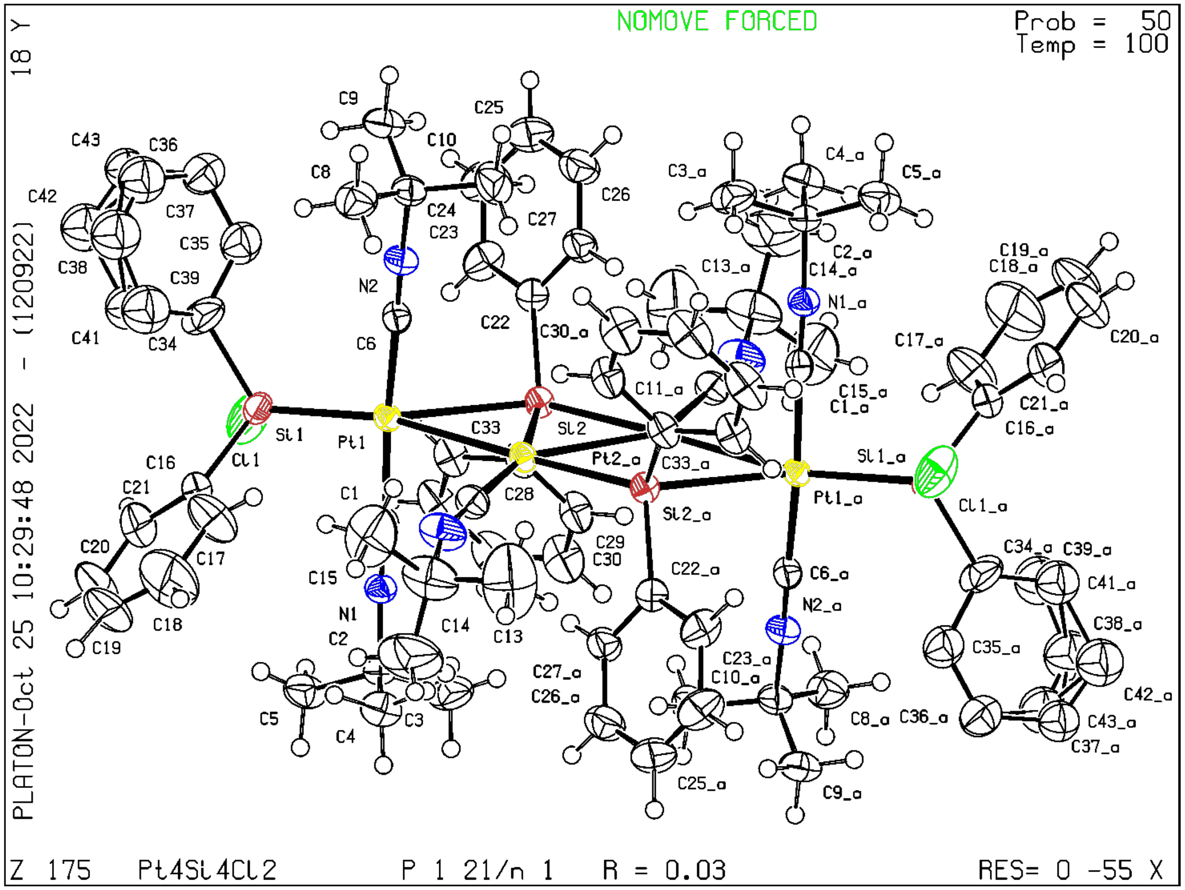

Download CIF editor (publCIF) from the IUCr
Download CIF editor (enCIFer) from the CCDC
Test a new CIF entry
