# Supplementary material for: Dinuclear and tetranuclear group 10 metal complexes constructed from linear tetrasilane comprising both Si-H and Si-Si moieties
Source: Commun Chem. 2023 May 15;6:93. doi: 10.1038/s42004-023-00892-8 (PMC10185686; doi:10.1038/s42004-023-00892-8)
Supplement: Supplementary file 19 — Supplementary Data 17 [file 42004_2023_892_MOESM19_ESM.pdf]

The DFT-optimized Geometry for disilane **1a**$_{\mathbf{opt}}$ (in XYZ format)

| | | | |
|---|---|---|---|
| Si | -1.058299 | -0.414699 | 0.377100 |
| Si | -2.907200 | 0.265699 | -0.958400 |
| C | -1.092799 | -2.306999 | 0.193700 |
| C | -0.727799 | -2.902099 | -1.027900 |
| C | -0.800299 | -4.281900 | -1.209599 |
| C | -1.241400 | -5.102299 | -0.172000 |
| C | -1.615599 | -4.532300 | 1.043800 |
| C | -1.546000 | -3.150800 | 1.221800 |
| C | -1.306000 | 0.054899 | 2.201099 |
| C | -0.559600 | -0.575499 | 3.213800 |
| C | -0.710499 | -0.221800 | 4.553599 |
| C | -1.611199 | 0.779599 | 4.914900 |
| C | -2.354900 | 1.424900 | 3.928700 |
| C | -2.202000 | 1.066799 | 2.588999 |
| C | -4.366899 | -0.884800 | -0.627900 |
| C | -4.951900 | -0.998700 | 0.645500 |
| C | -6.034800 | -1.847100 | 0.868099 |
| C | -6.554500 | -2.605900 | -0.180400 |
| C | -5.986099 | -2.512299 | -1.449299 |
| C | -4.903799 | -1.660600 | -1.668200 |
| C | -3.460100 | 2.069800 | -0.795100 |
| C | -2.541399 | 3.130900 | -0.872100 |
| C | -2.963899 | 4.456900 | -0.799400 |
| C | -4.317700 | 4.752100 | -0.642499 |
| C | -5.244799 | 3.714800 | -0.559399 |
| C | -4.819499 | 2.388700 | -0.635200 |
| Si | 1.058399 | 0.414800 | -0.376899 |
| Si | 2.907200 | -0.266400 | 0.958300 |
| C | 1.093099 | 2.307099 | -0.192900 |
| C | 0.728600 | 2.901900 | 1.028900 |
| C | 0.801299 | 4.281599 | 1.211100 |
| C | 1.242099 | 5.102299 | 0.173500 |
| C | 1.615799 | 4.532600 | -1.042500 |
| C | 1.546000 | 3.151100 | -1.220900 |
| C | 1.305799 | -0.054200 | -2.200999 |
| C | 0.558900 | 0.576300 | -3.213399 |
| C | 0.709599 | 0.223100 | -4.553300 |
| C | 1.610500 | -0.778000 | -4.915100 |
| C | 2.354599 | -1.423399 | -3.929299 |
| C | 2.201900 | -1.065799 | -2.589500 |
| C | 4.366899 | 0.884299 | 0.628499 |
| C | 4.951400 | 0.999600 | -0.645100 |
| C | 6.034300 | 1.848099 | -0.867099 |
| C | 6.554600 | 2.605600 | 0.182000 |
| C | 5.986700 | 2.510500 | 1.451100 |
| C | 4.904500 | 1.658799 | 1.669400 |
| C | 3.460100 | -2.070399 | 0.793899 |
| C | 2.541600 | -3.131600 | 0.872300 |
| C | 2.964100 | -4.457599 | 0.798700 |
| C | 4.317700 | -4.752599 | 0.639399 |
| C | 5.244500 | -3.715200 | 0.554899 |
| C | 4.819299 | -2.389099 | 0.631699 |
| H | -2.503399 | 0.016099 | -2.376099 |
| H | -0.378400 | -2.282299 | -1.849600 |
| H | -0.509599 | -4.715900 | -2.162800 |
| H | -1.298800 | -6.178600 | -0.311999 |
| H | -1.969200 | -5.163100 | 1.855499 |
| H | -1.853000 | -2.725999 | 2.173600 |
| H | 0.150300 | -1.358100 | 2.956900 |
| H | -0.121999 | -0.727100 | 5.314900 |
| H | -1.729800 | 1.056900 | 5.958999 |
| H | -3.055800 | 2.210200 | 4.199700 |
| H | -2.781800 | 1.595299 | 1.836300 |
| H | -4.556100 | -0.420700 | 1.477900 |
| H | -6.471400 | -1.918700 | 1.860999 |
| H | -7.398000 | -3.269299 | -0.007700 |
| H | -6.385100 | -3.102800 | -2.270099 |
| H | -4.470300 | -1.598599 | -2.663499 |
| H | -1.478499 | 2.928999 | -0.979200 |
| H | -2.231000 | 5.256799 | -0.858399 |
| H | -4.647900 | 5.785900 | -0.582800 |
| H | -6.301900 | 3.936600 | -0.436400 |
| H | -5.555300 | 1.591200 | -0.571099 |
| H | 2.503399 | -0.017700 | 2.376199 |
| H | 0.379400 | 2.281900 | 1.850600 |
| H | 0.510899 | 4.715300 | 2.164399 |

| | | | |
|---|---|---|---|
| H | 1.299600 | 6.178600 | 0.313900 |
| H | 1.969200 | 5.163600 | -1.854200 |
| H | 1.852600 | 2.726500 | -2.172899 |
| H | -0.151099 | 1.358699 | -2.956099 |
| H | 0.120799 | 0.728500 | -5.314300 |
| H | 1.728900 | -1.054900 | -5.959400 |
| H | 3.055599 | -2.208500 | -4.200700 |
| H | 2.782000 | -1.594400 | -1.837099 |
| H | 4.555000 | 0.422800 | -1.477900 |
| H | 6.470400 | 1.920800 | -1.860100 |
| H | 7.398100 | 3.269000 | 0.009699 |
| H | 6.386200 | 3.099899 | 2.272299 |
| H | 4.471400 | 1.595600 | 2.664900 |
| H | 1.478800 | -2.929800 | 0.981100 |
| H | 2.231399 | -5.257500 | 0.858699 |
| H | 4.647900 | -5.786400 | 0.579000 |
| H | 6.301399 | -3.936899 | 0.430200 |
| H | 5.554899 | -1.591600 | 0.566600 |
