# Supplementary material for: Dinuclear and tetranuclear group 10 metal complexes constructed from linear tetrasilane comprising both Si-H and Si-Si moieties
Source: Commun Chem. 2023 May 15;6:93. doi: 10.1038/s42004-023-00892-8 (PMC10185686; doi:10.1038/s42004-023-00892-8)
Supplement: Supplementary file 20 — Supplementary Data 18 [file 42004_2023_892_MOESM20_ESM.pdf]

The DFT-optimized Geometry for disilane **1b**$_{opt}$ (in XYZ format)

Si	2.969299	-0.185499	-0.831200
Si	0.940200	0.740400	0.055200
Cl	2.881400	0.025500	-2.923699
C	4.455299	0.834599	-0.279000
C	4.517800	2.214700	-0.539899
C	5.612299	2.970700	-0.127700
C	6.665100	2.362500	0.556500
C	6.620600	0.995599	0.823300
C	5.525000	0.238499	0.408500
C	3.302400	-2.009500	-0.498000
C	2.999199	-2.574300	0.751599
C	3.311900	-3.902700	1.036400
C	3.933200	-4.693999	0.072400
C	4.239100	-4.152999	-1.175999
C	3.927000	-2.824499	-1.457999
C	0.632400	2.289500	-0.999400
C	0.870700	3.577299	-0.490000
C	0.698300	4.710100	-1.284900
C	0.285999	4.579599	-2.609900
C	0.045600	3.310200	-3.135200
C	0.217500	2.179100	-2.339000
C	1.201300	1.262100	1.861100
C	2.331600	0.901500	2.613100
C	2.465000	1.280799	3.948600
C	1.468799	2.036100	4.563600
C	0.340300	2.411200	3.835800
C	0.208900	2.026500	2.503100
Si	-0.940300	-0.740500	-0.055200
Si	-2.969199	0.185599	0.831200
C	-0.632500	-2.289699	0.999300
C	-1.201500	-1.262200	-1.861100
Cl	-2.881300	-0.025500	2.923699
C	-4.455399	-0.834400	0.279100
C	-3.302200	2.009500	0.498100
C	-0.870700	-3.577399	0.489800
C	-0.217600	-2.179300	2.339000
C	-2.331899	-0.901799	-2.613100
C	-0.209000	-2.026500	-2.503200
C	-4.517899	-2.214600	0.539699
C	-5.525200	-0.238100	-0.408099
C	-2.999299	2.574100	-0.751800
C	-3.926199	2.824900	1.458099
C	-0.698300	-4.710299	1.284700
C	-0.045600	-3.310399	3.135000
C	-2.465400	-1.281100	-3.948500
C	-0.340400	-2.411200	-3.835900
C	-5.612500	-2.970400	0.127500
C	-6.620899	-0.995100	-0.822799
C	-3.311800	3.902499	-1.036599
C	-4.238000	4.153300	1.176099
C	-0.285999	-4.579900	2.609700
C	-1.469100	-2.036300	-4.563600
C	-6.665399	-2.361999	-0.556300
C	-3.932499	4.694199	-0.072400
H	3.707700	2.702600	-1.076200
H	5.644699	4.035800	-0.340999
H	7.519000	2.952999	0.878099
H	7.441100	0.515800	1.350400
H	5.505800	-0.828500	0.615900
H	2.499299	-1.982300	1.513800
H	3.054399	-4.319199	2.005699
H	4.173399	-5.731300	0.290600
H	4.720800	-4.766499	-1.932800
H	4.168400	-2.416700	-2.435900
H	1.193800	3.698399	0.540400
H	0.887699	5.695600	-0.867199
H	0.157200	5.461899	-3.231599
H	-0.268599	3.197700	-4.169600
H	0.035000	1.199400	-2.771800
H	3.135700	0.332499	2.154799
H	3.352399	0.990600	4.504800
H	1.571599	2.332999	5.604000
H	-0.443699	2.998399	4.306100
H	-0.680700	2.332300	1.956999
H	-1.193900	-3.698500	-0.540500
H	-0.035100	-1.199600	2.771800

H	-3.136000	-0.333100	-2.154599
H	0.680700	-2.332200	-1.957199
H	-3.707700	-2.702600	1.075699
H	-5.506000	0.828900	-0.615299
H	-2.499800	1.981899	-1.514099
H	-4.167300	2.417199	2.436200
H	-0.887699	-5.695800	0.866999
H	0.268599	-3.197999	4.169500
H	-3.352900	-0.991100	-4.504600
H	0.443599	-2.998199	-4.306299
H	-5.644799	-4.035600	0.340700
H	-7.441500	-0.515199	-1.349700
H	-3.054599	4.318800	-2.006100
H	-4.719300	4.767100	1.933099
H	-0.157200	-5.462199	3.231400
H	-1.571900	-2.333200	-5.604000
H	-7.519400	-2.952500	-0.877799
H	-4.172600	5.731400	-0.290700
