# Supplementary material for: Dinuclear and tetranuclear group 10 metal complexes constructed from linear tetrasilane comprising both Si-H and Si-Si moieties
Source: Commun Chem. 2023 May 15;6:93. doi: 10.1038/s42004-023-00892-8 (PMC10185686; doi:10.1038/s42004-023-00892-8)
Supplement: Supplementary file 21 — Supplementary Data 19 [file 42004_2023_892_MOESM21_ESM.pdf]

The DFT-optimized Geometry for Complex $\mathbf{2}_{\mathbf{opt}}$ (in XYZ format)

Pt	-1.765200	-0.000500	0.000000	C	-0.117699	2.490200	1.880800
Si	-0.077000	-1.757700	-0.106799	C	-0.502900	3.204900	-1.078899
Si	2.172200	-1.107300	0.421999	C	0.833499	3.421200	2.337099
N	-3.882600	-2.361600	0.044300	C	-0.355299	4.556200	-0.720600
C	-0.638299	-5.587000	1.617899	C	-1.208900	2.669799	4.067000
C	-3.091099	-1.500400	0.017600	C	4.326200	1.929600	1.358100
C	-1.132799	-2.126400	-2.784399	C	-1.239500	3.957000	-3.287699
C	3.606499	-1.625399	2.889500	C	3.559799	3.608699	-0.185100
C	5.331299	-4.127400	-1.374400	C	-1.082199	5.290399	-2.908400
C	-0.116100	-2.489900	-1.881200	C	3.463600	2.293999	0.312200
C	-0.501200	-3.205499	1.078300	C	-0.956699	2.934200	-2.382599
C	0.835799	-3.420100	-2.337600	C	0.762499	3.973000	3.616500
C	-0.353300	-4.556700	0.719500	C	-0.260400	3.601400	4.486699
C	-1.206800	-2.669600	-4.067599	C	1.521000	0.468499	-3.165200
C	4.327999	-1.927200	-1.356900	C	3.825200	1.562599	-4.265199
C	-1.236900	-3.958699	3.287000	C	1.737100	0.396700	-4.539900
C	3.561400	-3.607000	0.185400	C	4.480000	4.516100	0.337400
C	-1.079300	-5.292000	2.907300	C	5.248000	2.835600	1.884100
C	3.464999	-2.292200	-0.311600	C	2.890800	0.947300	-5.096099
C	-0.954699	-2.935499	2.382300	C	2.447399	1.084199	-2.306500
C	0.765300	-3.971599	-3.617199	N	-3.883600	2.359600	-0.044199
C	-4.770899	-3.494300	0.089100	C	-3.091800	1.498700	-0.017500
C	-0.257700	-3.600400	-4.487400	C	-4.772400	3.491999	-0.088900
C	1.520699	-0.467399	3.165300	C	-4.979099	3.867300	-1.562599
C	3.824900	-1.561000	4.265900	C	-4.109599	4.641600	0.681600
C	1.736400	-0.395600	4.540000	C	-6.097399	3.079600	0.565899
C	4.482399	-4.513799	-0.336799	H	-0.512200	-6.622299	1.309300
C	5.250499	-2.832600	-1.882599	H	-1.874400	-1.391400	-2.478200
C	-4.977199	-3.869899	1.562799	H	4.352100	-2.105099	2.261100
C	-4.107800	-4.643600	-0.681700	H	6.050799	-4.832299	-1.783199
C	-6.096199	-3.082399	-0.565400	H	1.658799	-3.712900	-1.692600
C	2.890099	-0.945899	5.096500	H	-0.016999	-4.812500	-0.282000
C	2.447399	-1.082999	2.306899	H	-2.004499	-2.362400	-4.740300
Si	-0.078000	1.757600	0.106599	H	4.276300	-0.926300	-1.775699
Si	2.171700	1.108400	-0.421800	H	-1.576100	-3.714399	4.291000
C	-0.641000	5.586100	-1.619300	H	2.904800	-3.927299	0.991800
C	-1.134300	2.126300	2.784000	H	-1.294999	-6.092400	3.610500
C	3.606499	1.626900	-2.888900	H	-1.091899	-1.903400	2.696100
C	5.328500	4.130299	1.375600	H	1.520300	-4.685900	-3.936100

H	-0.310500	-4.026499	-5.486200
H	0.619699	-0.018900	2.756700
H	4.729799	-1.991499	4.688100
H	1.004100	0.099400	5.172300
H	4.537000	-5.522200	0.066300
H	5.906600	-2.522899	-2.692100
H	-5.642000	-4.736600	1.623600
H	-4.023800	-4.123500	2.033100
H	-5.433700	-3.043300	2.114899
H	-4.768599	-5.515600	-0.666700
H	-3.924800	-4.360700	-1.721699
H	-3.154200	-4.914299	-0.222100
H	-6.790100	-3.927600	-0.543099
H	-6.552999	-2.245200	-0.029899
H	-5.941499	-2.789200	-1.607600
H	3.061200	-0.892200	6.168700
H	-0.515099	6.621600	-1.311099
H	-1.875400	1.390700	2.477900
H	4.351900	2.106700	-2.260300
H	6.047399	4.835599	1.784700
H	1.656500	3.714299	1.692199
H	-0.018800	4.812400	0.280699
H	-2.006500	2.362300	4.739800
H	4.274800	0.928899	1.777100
H	-1.578999	3.712199	-4.291399
H	2.903500	3.928600	-0.992000
H	-1.298400	6.090500	-3.611899
H	-1.093800	1.901899	-2.695999
H	1.516900	4.687799	3.935300
H	-0.313700	4.027699	5.485400
H	0.620100	0.019700	-2.756900
H	4.730100	1.993300	-4.687200
H	1.005000	-0.098400	-5.172400
H	4.534399	5.524400	-0.065900
H	5.903799	2.526399	2.694100
H	3.062199	0.893700	-6.168300
H	-5.644300	4.733800	-1.623399
H	-4.025800	4.121200	-2.033100
H	-5.435300	3.040499	-2.114500
H	-4.770600	5.513300	0.666600
H	-3.926299	4.358900	1.721599
H	-3.156099	4.912500	0.221900
H	-6.791599	3.924600	0.543600
H	-6.553999	2.242199	0.030500
H	-5.942300	2.786599	1.607999
