# Supplementary material for: Dinuclear and tetranuclear group 10 metal complexes constructed from linear tetrasilane comprising both Si-H and Si-Si moieties
Source: Commun Chem. 2023 May 15;6:93. doi: 10.1038/s42004-023-00892-8 (PMC10185686; doi:10.1038/s42004-023-00892-8)
Supplement: Supplementary file 22 — Supplementary Data 20 [file 42004_2023_892_MOESM22_ESM.pdf]

The DFT-optimized Geometry for Complex **5a**$_{\mathbf{opt}}$ (in XYZ format)

Pd	-1.399000	-0.080999	-0.048000
Si	-3.072000	1.667000	0.347000
Si	-0.229000	1.827000	0.605000
N	-3.627999	-2.279999	-0.367999
C	-3.944000	2.116000	-1.282999
C	-4.157000	1.187999	-2.317000
C	-4.821000	1.544000	-3.490000
C	-5.285999	2.847999	-3.661000
C	-5.081000	3.788999	-2.654000
C	-4.416000	3.424999	-1.482000
C	-4.349000	0.946999	1.565999
C	-5.728000	1.119999	1.369000
C	-6.658999	0.616000	2.278999
C	-6.227999	-0.071999	3.412999
C	-4.862000	-0.253000	3.628999
C	-3.936999	0.246999	2.712999
C	-0.050000	3.381000	-0.457000
C	-0.417999	3.337000	-1.813000
C	-0.237000	4.436999	-2.650000
C	0.323000	5.612000	-2.146999
C	0.699000	5.677999	-0.806000
C	0.514999	4.574000	0.027999
C	-0.129000	2.277000	2.443999
C	-0.699000	3.440999	2.988000
C	-0.634999	3.706999	4.355999
C	0.000999	2.811000	5.214000
C	0.568999	1.645999	4.698000
C	0.500999	1.385000	3.330999
C	-2.770000	-1.483999	-0.316000
C	-4.717000	-3.220000	-0.342000
C	-4.445000	-4.296000	-1.401000
C	-4.761999	-3.831999	1.064999
C	-6.011999	-2.455999	-0.649999
Pd	1.399000	0.080999	0.048000
Si	3.072000	-1.668000	-0.346000
Si	0.229000	-1.827000	-0.605000
N	3.627999	2.279999	0.366999
C	3.943000	-2.116000	1.283999
C	4.159000	-1.186999	2.317000
C	4.822000	-1.543000	3.490000
C	5.283999	-2.847999	3.663000
C	5.077000	-3.788999	2.655999
C	4.414000	-3.424999	1.484999
C	4.349000	-0.946999	-1.564999
C	5.728999	-1.121999	-1.369000
C	6.659999	-0.618000	-2.278999
C	6.228999	0.069999	-3.411999
C	4.863000	0.252000	-3.628999
C	3.937999	-0.246999	-2.711999
C	0.049000	-3.381000	0.457000
C	0.417000	-3.337000	1.813000
C	0.236000	-4.436999	2.650000
C	-0.323999	-5.610999	2.148000
C	-0.700000	-5.677999	0.806000
C	-0.514999	-4.574000	-0.027999
C	0.130000	-2.277000	-2.443999
C	0.700000	-3.440999	-2.988000
C	0.634999	-3.706999	-4.354999
C	0.000000	-2.811000	-5.214000
C	-0.566999	-1.645999	-4.699000
C	-0.499999	-1.385000	-3.330999
C	2.770000	1.484999	0.315000
C	4.717000	3.220000	0.339999
C	4.445000	4.297000	1.399000
C	4.761999	3.831999	-1.067999
C	6.011999	2.455999	0.647999
H	-2.791999	3.031000	0.914000
H	-3.793000	0.170000	-2.204000
H	-4.973000	0.806000	-4.273999
H	-5.801000	3.129999	-4.576000
H	-5.434999	4.809000	-2.779999
H	-4.258000	4.172000	-0.708000
H	-6.081999	1.658999	0.493000
H	-7.722000	0.765000	2.104999
H	-6.951000	-0.461999	4.124999
H	-4.515000	-0.783999	4.513000

H	-2.874999	0.088000	2.891000
H	-0.861000	2.428999	-2.216000
H	-0.538000	4.379000	-3.692000
H	0.461999	6.471999	-2.797000
H	1.129000	6.592999	-0.405999
H	0.810999	4.644000	1.071000
H	-1.212000	4.146000	2.338000
H	-1.087000	4.611999	4.751000
H	0.051000	3.017000	6.280000
H	1.063000	0.939000	5.361000
H	0.946999	0.472999	2.938000
H	-5.264000	-5.022000	-1.398000
H	-4.376000	-3.854000	-2.399000
H	-3.512000	-4.821000	-1.185000
H	-5.582999	-4.554000	1.117000
H	-3.826000	-4.348000	1.292000
H	-4.930000	-3.056000	1.817000
H	-6.856000	-3.152000	-0.630000
H	-6.185000	-1.671000	0.091000
H	-5.966000	-1.994999	-1.641000
H	2.791999	-3.031000	-0.914000
H	3.796000	-0.169000	2.203000
H	4.974999	-0.804000	4.272999
H	5.798999	-3.128999	4.578000
H	5.430000	-4.810000	2.784000
H	4.254000	-4.173000	0.712000
H	6.081999	-1.660999	-0.493000
H	7.722999	-0.768000	-2.103999
H	6.953000	0.459999	-4.124000
H	4.516999	0.782999	-4.512000
H	2.876000	-0.088000	-2.890000
H	0.860000	-2.428999	2.216000
H	0.537000	-4.378000	3.692000
H	-0.464000	-6.471999	2.797000
H	-1.130000	-6.592999	0.405999
H	-0.810999	-4.644000	-1.071000
H	1.212000	-4.146000	-2.338000
H	1.088000	-4.612999	-4.751000
H	-0.049000	-3.018000	-6.280000
H	-1.061000	-0.939000	-5.361000
H	-0.944999	-0.471999	-2.938000
H	5.264000	5.022000	1.395000
H	4.376000	3.854999	2.397000
H	3.512000	4.822000	1.183000
H	5.581999	4.554000	-1.119999
H	3.825000	4.348000	-1.293999
H	4.929000	3.056000	-1.819000
H	6.855000	3.153000	0.628000
H	6.184000	1.671999	-0.093000
H	5.966000	1.995999	1.639000
H	-2.014000	-2.592000	4.713000
H	-3.437000	-4.634000	4.754000
H	-3.328000	-6.246000	2.864000
H	-1.794000	-5.847000	0.977000
H	1.132000	-4.605000	3.100000
H	3.262000	-5.599000	3.841000
H	5.089000	-6.014000	2.205000
H	4.760000	-5.397000	-0.186000
H	2.646000	-4.383000	-0.927000
