# Supplementary material for: Dinuclear and tetranuclear group 10 metal complexes constructed from linear tetrasilane comprising both Si-H and Si-Si moieties
Source: Commun Chem. 2023 May 15;6:93. doi: 10.1038/s42004-023-00892-8 (PMC10185686; doi:10.1038/s42004-023-00892-8)
Supplement: Supplementary file 23 — Supplementary Data 21 [file 42004_2023_892_MOESM23_ESM.pdf]

The DFT-optimized Geometry for Complex **5b**$_{\mathbf{opt}}$ (in XYZ format)

Pd	-1.447800	-0.015600	-0.028600
Pd	1.447800	0.015700	-0.028700
Si	-2.988200	1.870100	0.417200
Si	-0.102000	1.886899	0.146799
Si	2.988200	-1.870300	0.416400
Si	0.102100	-1.886899	0.146200
N	-3.203800	-1.579400	-1.977800
N	-3.832400	-2.140699	0.012699
N	3.203699	1.580400	-1.977300
N	3.832500	2.140699	0.013400
C	-2.926899	-1.374300	-0.657799
C	-4.273700	-2.456199	-2.133400
C	-4.670700	-2.815599	-0.877400
C	-2.436599	-0.911499	-3.048500
C	-3.230399	0.201400	-3.730599
C	-1.849400	-1.891099	-4.063199
C	-3.881300	-2.191399	1.487800
C	-5.161200	-1.585100	2.061100
C	-3.624100	-3.594000	2.033799
C	-4.823899	-2.919100	-3.440099
C	-5.747500	-3.775999	-0.498000
C	2.926899	1.374699	-0.657400
C	4.670700	2.816100	-0.876600
C	4.273600	2.457199	-2.132700
C	3.881400	2.191000	1.488500
C	3.624300	3.593499	2.034799
C	5.161200	1.584299	2.061699
C	2.436300	0.912900	-3.048300
C	3.229900	-0.199900	-3.730699
C	1.849400	1.893000	-4.062700
C	5.747600	3.776099	-0.496799
C	4.823699	2.920499	-3.439200
C	-3.756399	1.681000	2.157500
C	-5.057900	2.142500	2.422499
C	-5.610999	2.067199	3.700600
C	-4.870900	1.531200	4.754000
C	-3.574600	1.075599	4.517899
C	-3.031300	1.148899	3.234999
C	-4.460500	2.061000	-0.789299
C	-4.639099	3.270299	-1.484700
C	-5.716600	3.465799	-2.350299
C	-6.659799	2.455899	-2.531400
C	-6.512700	1.250699	-1.844599
C	-5.424700	1.057900	-0.994599
C	-0.093400	3.215600	-1.219000
C	-1.040400	3.205400	-2.256299
C	-1.046900	4.181999	-3.252099
C	-0.092799	5.199000	-3.239199
C	0.866099	5.226200	-2.226500
C	0.862799	4.246400	-1.233100
C	0.118499	2.815699	1.792600
C	0.530600	2.103300	2.932700
C	0.652600	2.719200	4.176899
C	0.354400	4.075299	4.312699
C	-0.064799	4.802600	3.199500
C	-0.180700	4.179700	1.956899
C	3.756499	-1.681900	2.156699
C	5.057999	-2.143699	2.421499
C	5.611099	-2.068900	3.699600
C	4.871099	-1.533300	4.753300
C	3.574899	-1.077400	4.517400
C	3.031500	-1.150199	3.234500
C	4.460500	-2.060699	-0.790200
C	5.424600	-1.057500	-0.995100
C	6.512600	-1.249900	-1.845200
C	6.659700	-2.454700	-2.532599
C	5.716500	-3.464700	-2.351900
C	4.639099	-3.269700	-1.486199
C	-0.118300	-2.816400	1.791599
C	0.181000	-4.180400	1.955200
C	0.065300	-4.804000	3.197500
C	-0.353800	-4.077299	4.311200
C	-0.652099	-2.721100	4.176000
C	-0.530400	-2.104600	2.932100
C	0.093300	-3.215199	-1.220100
C	1.040300	-3.205000	-2.257399

C	1.046499	-4.181300	-3.253299
C	0.092200	-5.198100	-3.240800
C	-0.866700	-5.225300	-2.228099
C	-0.863200	-4.245699	-1.234399
H	-2.527100	3.301300	0.432200
H	2.527100	-3.301400	0.430799
H	-1.607099	-0.451000	-2.496099
H	-2.569200	0.737900	-4.419000
H	-4.065100	-0.190099	-4.320100
H	-3.630099	0.911299	-3.005300
H	-1.071900	-1.376400	-4.636000
H	-1.393199	-2.753700	-3.575999
H	-2.597100	-2.240300	-4.780900
H	-3.045300	-1.550200	1.782400
H	-5.038000	-1.458700	3.140400
H	-5.361499	-0.597199	1.644200
H	-6.035000	-2.223699	1.902300
H	-3.478600	-3.530200	3.115400
H	-4.464999	-4.269699	1.853600
H	-2.716700	-4.029699	1.610600
H	-4.997200	-2.091999	-4.133100
H	-4.162000	-3.637899	-3.934599
H	-5.784500	-3.414600	-3.282700
H	-6.426799	-3.371099	0.256000
H	-6.348699	-4.018400	-1.377099
H	-5.346200	-4.719300	-0.111999
H	3.045200	1.549799	1.782800
H	3.478700	3.529400	3.116400
H	4.465199	4.269100	1.854800
H	2.716900	4.029300	1.611600
H	5.038100	1.458200	3.140899
H	5.361100	0.596400	1.644799
H	6.035100	2.222699	1.902600
H	1.606700	0.452499	-2.495999
H	2.568700	-0.736100	-4.419300
H	4.064800	0.191500	-4.319900
H	3.629300	-0.910100	-3.005400
H	1.071800	1.378600	-4.635699
H	1.393299	2.755500	-3.575199
H	2.597100	2.242199	-4.780200
H	6.426799	3.370899	0.257100
H	6.348899	4.018700	-1.375799
H	5.346499	4.719400	-0.110500
H	4.996699	2.093700	-4.132700
H	4.162000	3.639799	-3.933200
H	5.784500	3.415600	-3.281700
H	-5.650500	2.567299	1.615799
H	-6.620899	2.431000	3.874800
H	-5.299400	1.473500	5.751499
H	-2.984299	0.666000	5.334300
H	-2.016200	0.800000	3.067700
H	-3.923100	4.075900	-1.340500
H	-5.824999	4.412600	-2.873999
H	-7.505200	2.607700	-3.197800
H	-7.245499	0.457599	-1.973900
H	-5.323600	0.103700	-0.485300
H	-1.797800	2.425600	-2.275699
H	-1.801800	4.151699	-4.033700
H	-0.097300	5.965399	-4.010000
H	1.614300	6.015099	-2.205900
H	1.618400	4.289300	-0.452700
H	0.760099	1.044000	2.835400
H	0.975900	2.142400	5.040100
H	0.442400	4.560400	5.281500
H	-0.309400	5.857200	3.299799
H	-0.518800	4.761199	1.103699
H	5.650500	-2.568199	1.614599
H	6.621099	-2.432800	3.873600
H	5.299700	-1.476000	5.750800
H	2.984600	-0.668200	5.334000
H	2.016399	-0.801199	3.067399
H	5.323600	-0.103499	-0.485300
H	7.245399	-0.456599	-1.974100
H	7.505100	-2.606199	-3.199099
H	5.824999	-4.411300	-2.876099
H	3.923100	-4.075400	-1.342299
H	0.519100	-4.761500	1.101599
H	0.309999	-5.858599	3.297299
H	-0.441699	-4.562900	5.279599
H	-0.975400	-2.144799	5.039500

H	-0.760000	-1.045299	2.835400	H	0.096600	-5.964199	-4.011800
H	1.797800	-2.425300	-2.276500	H	-1.615100	-6.013999	-2.207699
H	1.801499	-4.150899	-4.034900	H	-1.618799	-4.288700	-0.454100
