# Supplementary material for: Dinuclear and tetranuclear group 10 metal complexes constructed from linear tetrasilane comprising both Si-H and Si-Si moieties
Source: Commun Chem. 2023 May 15;6:93. doi: 10.1038/s42004-023-00892-8 (PMC10185686; doi:10.1038/s42004-023-00892-8)
Supplement: Supplementary file 24 — Supplementary Data 22 [file 42004_2023_892_MOESM24_ESM.pdf]

The DFT-optimized Geometry for Complex **6**$_{opt}$ (in XYZ format)

Pd	0.000000	0.001100	-0.003700
Pd	2.618800	0.970699	0.000899
Si	0.386700	2.269400	-0.002300
N	3.947300	3.460200	-1.405199
N	5.242999	-0.058099	1.413400
C	0.302600	3.394599	-1.548400
C	0.503299	2.806900	-2.811100
C	0.387699	3.543400	-3.989600
C	0.074100	4.903300	-3.937899
C	-0.127399	5.510400	-2.698400
C	-0.019800	4.761800	-1.524400
C	0.841000	3.303799	1.542600
C	0.460099	2.816500	2.806500
C	0.816299	3.473800	3.983700
C	1.562599	4.652999	3.929199
C	1.950699	5.158300	2.688399
C	1.598100	4.487000	1.515600
C	3.353000	2.644600	-0.801700
C	4.632200	4.425900	-2.216000
C	4.000699	5.802199	-1.964499
C	6.114399	4.424300	-1.815900
C	4.463400	4.014200	-3.685500
C	4.263199	0.176600	0.807100
C	6.388200	-0.349300	2.227600
C	6.803300	-1.805000	1.973800
C	7.515499	0.615499	1.833699
C	5.986899	-0.149300	3.696199
Pd	-0.468800	-2.751800	-0.004400
Si	1.771000	-1.467899	0.000400
N	1.028900	-5.145200	-1.406800
N	-2.672999	-4.513400	1.401500
C	2.793900	-1.957500	-1.541299
C	2.191499	-1.835099	-2.806999
C	2.893100	-2.102600	-3.982100
C	4.226800	-2.512800	-3.923700
C	4.846600	-2.643999	-2.681000
C	4.138600	-2.363299	-1.510300
C	2.433600	-2.378199	1.548000
C	2.200199	-1.802200	2.810499
C	2.587899	-2.437900	3.989600
C	3.234200	-3.674800	3.938700
C	3.479800	-4.266000	2.699400
C	3.078199	-3.626200	1.524600
C	0.617200	-4.223099	-0.804499
C	1.532499	-6.218600	-2.214999
C	3.036900	-6.361400	-1.945400
C	0.784700	-7.502499	-1.828399
C	1.278200	-5.861100	-3.686299
C	-1.979699	-3.780800	0.797099
C	-3.499200	-5.364699	2.208900
C	-4.968000	-5.011000	1.936900
C	-3.208600	-6.821899	1.822800
C	-3.144900	-5.109200	3.680899
Pd	-2.149399	1.783900	-0.002999
Si	-2.157500	-0.798600	-0.003300
N	-4.975099	1.680300	-1.397300
N	-2.575600	4.573400	1.401999
C	-3.090300	-1.436200	-1.548200
C	-2.684800	-0.968000	-2.811599
C	-3.265199	-1.438399	-3.989100
C	-4.283400	-2.392700	-3.935500
C	-4.705200	-2.871700	-2.695100
C	-4.110100	-2.402199	-1.522100
C	-3.279199	-0.922800	1.542500
C	-2.665200	-1.007300	2.805700
C	-3.411200	-1.029400	3.983700
C	-4.805700	-0.976500	3.930800
C	-5.439000	-0.895099	2.690599
C	-4.682600	-0.862799	1.517000
C	-3.968500	1.578200	-0.798199
C	-6.162099	1.782500	-2.197200
C	-7.037700	0.552500	-1.920800
C	-6.895399	3.073100	-1.805200
C	-5.736299	1.823199	-3.671900
C	-2.284900	3.606800	0.798700
C	-2.897799	5.717099	2.206599

C	-1.848300	6.805699	1.940799
C	-4.299500	6.202800	1.811299
C	-2.865800	5.282699	3.678899
H	0.759400	1.750100	-2.862500
H	0.542199	3.056499	-4.950199
H	-0.014999	5.482200	-4.853999
H	-0.376300	6.568400	-2.645800
H	-0.198800	5.252100	-0.570800
H	-0.131300	1.904099	2.859999
H	0.511500	3.066499	4.945300
H	1.840800	5.170200	4.844299
H	2.535899	6.074100	2.633800
H	1.926300	4.890100	0.560800
H	4.501999	6.553400	-2.582700
H	2.937400	5.789399	-2.218100
H	4.108100	6.090300	-0.914699
H	6.660500	5.154600	-2.420900
H	6.231199	4.689599	-0.761199
H	6.557100	3.437400	-1.977200
H	4.980100	4.733600	-4.328600
H	4.888200	3.021500	-3.858900
H	3.405000	3.993300	-3.956900
H	7.077700	-1.951000	0.925000
H	7.669200	-2.052400	2.595800
H	5.985300	-2.486800	2.221000
H	7.779800	0.492499	0.779499
H	7.210899	1.653000	1.997200
H	8.403100	0.412900	2.440899
H	6.844499	-0.362200	4.342099
H	5.664300	0.881000	3.870899
H	5.167500	-0.821500	3.962899
H	1.148999	-1.526500	-2.863800
H	2.399500	-1.991200	-4.945200
H	4.777299	-2.724700	-4.837100
H	5.886499	-2.959100	-2.623200
H	4.647400	-2.455800	-0.553999
H	1.707000	-0.833000	2.861300
H	2.386200	-1.968300	4.949999
H	3.540499	-4.173200	4.855199
H	3.979199	-5.231300	2.647399
H	3.264800	-4.113999	0.571099
H	3.445000	-7.166300	-2.564600
H	3.560600	-5.432200	-2.184899
H	3.219300	-6.606700	-0.895099
H	1.151299	-8.338599	-2.431800
H	0.943000	-7.739999	-0.772500
H	-0.289099	-7.391500	-2.002700
H	1.652000	-6.665100	-4.328099
H	0.208199	-5.733000	-3.872700
H	1.791699	-4.932300	-3.947200
H	-5.217899	-5.181000	0.885599
H	-5.616499	-5.641499	2.553099
H	-5.162200	-3.962799	2.179100
H	-3.432100	-6.994699	0.766200
H	-2.157600	-7.066300	2.000600
H	-3.829200	-7.493300	2.424200
H	-3.759699	-5.749299	4.321499
H	-2.091400	-5.334699	3.868699
H	-3.330100	-4.064399	3.942499
H	-1.899300	-0.216100	-2.864499
H	-2.923400	-1.060599	-4.950300
H	-4.740699	-2.760799	-4.850699
H	-5.495000	-3.618299	-2.641000
H	-4.442700	-2.803100	-0.567899
H	-1.579099	-1.060200	2.857999
H	-2.904900	-1.088200	4.944799
H	-5.391799	-0.995799	4.846399
H	-6.524899	-0.849400	2.637100
H	-5.197299	-0.781699	0.562800
H	-7.944600	0.604300	-2.531200
H	-6.498899	-0.366099	-2.167400
H	-7.331000	0.515999	-0.867600
H	-7.806899	3.175299	-2.401999
H	-7.172500	3.055000	-0.747299
H	-6.262100	3.946599	-1.983899
H	-6.624000	1.905700	-4.306900
H	-5.088399	2.683400	-3.862399
H	-5.194199	0.912300	-3.937899
H	-1.862700	7.107299	0.889400
H	-2.068299	7.683999	2.555700

H	-0.847100	6.443600	2.188700
H	-4.328700	6.482699	0.754400
H	-5.042899	5.419600	1.984400
H	-4.569100	7.077899	2.410599
H	-3.111699	6.136800	4.317700
H	-3.593900	4.487500	3.862399
H	-1.872200	4.914800	3.946900
