# Supplementary material for: Dinuclear and tetranuclear group 10 metal complexes constructed from linear tetrasilane comprising both Si-H and Si-Si moieties
Source: Commun Chem. 2023 May 15;6:93. doi: 10.1038/s42004-023-00892-8 (PMC10185686; doi:10.1038/s42004-023-00892-8)
Supplement: Supplementary file 25 — Supplementary Data 23 [file 42004_2023_892_MOESM25_ESM.pdf]

The DFT-optimized Geometry for Complex $\mathbf{7}_{\mathbf{opt}}$ (in XYZ format)

Ni	1.077899	0.376899	0.552600
Si	0.999999	-1.084000	-1.097399
N	3.857099	-0.078300	1.507300
N	3.642999	1.699300	0.305800
C	1.447500	-2.889199	-0.642800
C	0.979200	-3.427500	0.570500
C	1.238799	-4.747800	0.936399
C	1.987800	-5.570099	0.093100
C	2.466500	-5.060000	-1.112399
C	2.196400	-3.738600	-1.473500
C	1.891700	-0.710900	-2.739700
C	3.296399	-0.702899	-2.818899
C	3.959300	-0.407900	-4.010299
C	3.229000	-0.098999	-5.158399
C	1.836199	-0.093400	-5.104300
C	1.181100	-0.398600	-3.910800
C	3.442399	-1.267400	2.265100
C	4.276900	-2.505400	1.947399
C	3.346500	-0.972400	3.762200
C	2.940899	2.765099	-0.427000
C	3.573600	3.091700	-1.777400
C	2.723300	3.994299	0.454000
C	5.108799	0.540700	1.488199
C	4.971699	1.666599	0.727200
C	6.339099	0.010300	2.142899
C	6.014500	2.663200	0.349200
C	2.943700	0.633699	0.789299
Ni	-1.077899	-0.376899	-0.552600
Si	-0.999999	1.084000	1.097399
N	-3.857099	0.078300	-1.507300
N	-3.642999	-1.699300	-0.305800
C	-1.447500	2.889199	0.642800
C	-0.979200	3.427500	-0.570600
C	-1.238799	4.747800	-0.936399
C	-1.987800	5.570099	-0.093100
C	-2.466500	5.060000	1.112399
C	-2.196400	3.738600	1.473500
C	-1.891700	0.710900	2.739700
C	-3.296399	0.702899	2.818899
C	-3.959300	0.407999	4.010299
C	-3.229000	0.099099	5.158399
C	-1.836199	0.093400	5.104300
C	-1.181100	0.398700	3.910800
C	-3.442399	1.267400	-2.265100
C	-4.276900	2.505400	-1.947399
C	-3.346400	0.972400	-3.762200
C	-2.940899	-2.765099	0.427000
C	-3.573700	-3.091900	1.777200
C	-2.723200	-3.994299	-0.454100
C	-5.108799	-0.540600	-1.488099
C	-4.971699	-1.666599	-0.727200
C	-6.339099	-0.010200	-2.142799
C	-6.014400	-2.663200	-0.349200
C	-2.943700	-0.633699	-0.789299
H	0.398100	-2.793700	1.239600
H	0.859900	-5.135699	1.879500
H	2.195300	-6.599599	0.373900
H	3.048500	-5.693700	-1.777599
H	2.571000	-3.364200	-2.423200
H	3.883999	-0.933800	-1.932299
H	5.046399	-0.419100	-4.043799
H	3.742700	0.132900	-6.088000
H	1.258600	0.142700	-5.995000
H	0.093400	-0.401499	-3.886800
H	2.427799	-1.446500	1.892399
H	3.777099	-3.386600	2.358999
H	4.359299	-2.651699	0.867299
H	5.280400	-2.460800	2.379499
H	2.894399	-1.825700	4.278000
H	4.327100	-0.794899	4.215200
H	2.721200	-0.092900	3.938700
H	1.958700	2.311800	-0.616499
H	2.884699	3.722899	-2.347400
H	4.512300	3.644400	-1.679299
H	3.753500	2.182200	-2.356200
H	2.066199	4.703600	-0.057600

H	2.242099	3.708099	1.392900
H	3.661700	4.510100	0.682900
H	6.170499	-0.263700	3.188499
H	6.736200	-0.872900	1.629799
H	7.122299	0.771800	2.131700
H	5.688899	3.692899	0.524000
H	6.915400	2.503200	0.945999
H	6.302599	2.581600	-0.704900
H	-0.398100	2.793600	-1.239600
H	-0.859900	5.135699	-1.879500
H	-2.195300	6.599599	-0.374000
H	-3.048600	5.693700	1.777500
H	-2.571000	3.364200	2.423200
H	-3.883999	0.933900	1.932299
H	-5.046399	0.419200	4.043799
H	-3.742599	-0.132799	6.088000
H	-1.258600	-0.142600	5.995000
H	-0.093400	0.401499	3.886900
H	-2.427799	1.446500	-1.892399
H	-3.777099	3.386600	-2.358999
H	-4.359299	2.651699	-0.867400
H	-5.280400	2.460700	-2.379600
H	-2.894300	1.825700	-4.278000
H	-4.326999	0.794899	-4.215200
H	-2.721100	0.092900	-3.938700
H	-1.958800	-2.311800	0.616599
H	-2.884899	-3.723099	2.347300
H	-4.512400	-3.644500	1.679000
H	-3.753700	-2.182400	2.356100
H	-2.066099	-4.703600	0.057500
H	-2.241900	-3.707999	-1.392900
H	-3.661599	-4.510100	-0.683100
H	-6.170599	0.263800	-3.188299
H	-6.736200	0.873000	-1.629599
H	-7.122399	-0.771700	-2.131500
H	-5.688899	-3.692899	-0.524000
H	-6.915500	-2.503200	-0.945899
H	-6.302499	-2.581600	0.704900
H	-0.537100	-1.339900	-1.721499
H	-0.371664	-0.927189	-1.191250
H	0.537100	1.339900	1.721499
H	0.371664	0.927189	1.191250
