# Supplementary material for: Dinuclear and tetranuclear group 10 metal complexes constructed from linear tetrasilane comprising both Si-H and Si-Si moieties
Source: Commun Chem. 2023 May 15;6:93. doi: 10.1038/s42004-023-00892-8 (PMC10185686; doi:10.1038/s42004-023-00892-8)
Supplement: Supplementary file 26 — Supplementary Data 24 [file 42004_2023_892_MOESM26_ESM.pdf]

The DFT-optimized Geometry for Complex **8**$_{opt}$ (in XYZ format)

Pt	-3.723999	-0.029700	0.226699
Pt	-1.142200	0.312200	-0.752900
Cl	-6.472500	0.177999	2.757800
Si	-6.088599	-0.012300	0.654000
Si	-1.143799	-0.594400	1.478399
N	-3.637699	2.913999	1.230700
N	-3.861299	-2.800400	-1.176199
N	-2.296600	1.464100	-3.397100
C	-3.632800	1.798099	0.881299
C	-3.745000	4.264600	1.708000
C	-4.114900	5.154099	0.512600
C	-4.845199	4.298100	2.778200
C	-2.390400	4.674699	2.299800
C	-3.777600	-1.779300	-0.611199
C	-3.995800	-4.040400	-1.889400
C	-2.647600	-4.367899	-2.544400
C	-4.392500	-5.126600	-0.880500
C	-5.089800	-3.855500	-2.950599
C	-1.845599	1.024000	-2.400400
C	-2.567500	2.020300	-4.693700
C	-1.473199	3.048300	-5.015200
C	-2.550600	0.872900	-5.714200
C	-3.947599	2.690399	-4.658499
C	-7.008300	1.466599	-0.110899
C	-6.498800	2.087899	-1.262800
C	-7.177499	3.140600	-1.880199
C	-8.379800	3.602199	-1.346899
C	-8.900199	3.003400	-0.199000
C	-8.223400	1.946199	0.406999
C	-7.126900	-1.541199	0.202799
C	-7.905600	-1.560000	-0.966200
C	-8.655300	-2.682799	-1.321700
C	-8.645299	-3.815500	-0.509100
C	-7.879499	-3.817600	0.657900
C	-7.127599	-2.695699	1.004000
C	-1.435700	-2.445600	1.844300
C	-2.112599	-2.941999	2.970500
C	-2.232000	-4.313600	3.206400
C	-1.666299	-5.230100	2.322199
C	-0.982000	-4.761099	1.197900
C	-0.874300	-3.391000	0.964600
C	-1.522300	0.381400	3.078199
C	-0.534900	1.198699	3.657099
C	-0.771600	1.911900	4.834200
C	-2.011099	1.825399	5.468699
C	-3.012600	1.033700	4.906300
C	-2.770000	0.329199	3.726399
Pt	3.723900	0.029700	-0.226699
Pt	1.142200	-0.311999	0.753000
Cl	6.472400	-0.178300	-2.757800
Si	6.088499	0.012200	-0.654000
Si	1.143799	0.594400	-1.478399
N	3.637599	-2.914099	-1.230500
N	3.861399	2.800499	1.175999
N	2.296600	-1.463800	3.397200
C	3.632600	-1.798099	-0.881099
C	3.745200	-4.264700	-1.707699
C	4.115199	-5.154099	-0.512200
C	4.845399	-4.298100	-2.777899
C	2.390700	-4.675100	-2.299499
C	3.777600	1.779400	0.610999
C	3.995900	4.040599	1.888999
C	2.647700	4.368200	2.544000
C	4.392599	5.126700	0.879999
C	5.089800	3.855700	2.950300
C	1.845599	-1.023799	2.400500
C	2.567600	-2.019799	4.693900
C	1.473400	-3.047799	5.015600
C	2.550700	-0.872300	5.714200
C	3.947699	-2.689900	4.658700
C	7.008200	-1.466799	0.110899
C	6.498700	-2.088000	1.262800
C	7.177299	-3.140699	1.880299
C	8.379499	-3.602400	1.347000
C	8.899999	-3.003599	0.199100
C	8.223200	-1.946500	-0.406999

C	7.127000	1.540999	-0.202999
C	7.905600	1.559900	0.966000
C	8.655500	2.682600	1.321400
C	8.645600	3.815300	0.508699
C	7.879800	3.817199	-0.658300
C	7.127699	2.695400	-1.004299
C	1.435700	2.445600	-1.844400
C	2.112599	2.941800	-2.970700
C	2.232000	4.313400	-3.206699
C	1.666499	5.229999	-2.322600
C	0.982200	4.761099	-1.198200
C	0.874400	3.391100	-0.964800
C	1.522100	-0.381500	-3.078099
C	0.534700	-1.198799	-3.656900
C	0.771400	-1.912200	-4.834000
C	2.010899	-1.825800	-5.468499
C	3.012399	-1.034100	-4.906100
C	2.769900	-0.329500	-3.726299
H	-3.338399	5.111099	-0.256500
H	-5.063800	4.836699	0.072199
H	-4.213299	6.190099	0.850400
H	-4.952199	5.319599	3.156099
H	-5.801500	3.974000	2.359999
H	-4.588800	3.639000	3.611300
H	-1.607700	4.628000	1.537799
H	-2.454500	5.701400	2.673299
H	-2.112300	4.014400	3.125000
H	-1.868700	-4.471300	-1.785600
H	-2.730200	-5.309400	-3.096000
H	-2.352299	-3.579499	-3.242000
H	-3.630400	-5.227500	-0.103499
H	-5.347599	-4.880700	-0.409900
H	-4.493500	-6.082700	-1.403400
H	-5.213900	-4.789199	-3.507500
H	-6.042900	-3.594699	-2.483700
H	-4.815500	-3.065500	-3.655999
H	-0.487999	2.576200	-5.017600
H	-1.658400	3.485200	-6.001600
H	-1.464800	3.850499	-4.272799
H	-1.576300	0.377000	-5.719800
H	-3.319700	0.132400	-5.475999
H	-2.748799	1.269300	-6.714700
H	-4.173599	3.121700	-5.638400
H	-4.726200	1.963499	-4.410600
H	-3.968199	3.491200	-3.914000
H	-5.550799	1.745600	-1.674800
H	-6.765500	3.603299	-2.773799
H	-8.908500	4.425099	-1.821500
H	-9.836799	3.359700	0.223000
H	-8.640799	1.489700	1.301199
H	-7.944600	-0.677199	-1.600399
H	-9.259100	-2.666000	-2.225999
H	-9.237600	-4.687000	-0.776700
H	-7.876099	-4.691500	1.305000
H	-6.547599	-2.709799	1.923900
H	-2.546400	-2.250799	3.687399
H	-2.762899	-4.664500	4.088299
H	-1.750100	-6.297900	2.508999
H	-0.524599	-5.467700	0.508200
H	-0.324899	-3.033600	0.095300
H	0.433899	1.274399	3.168200
H	0.013600	2.534700	5.257799
H	-2.196400	2.373100	6.389600
H	-3.989300	0.965800	5.378799
H	-3.583400	-0.247600	3.292900
H	3.338599	-5.111099	0.256799
H	5.064000	-4.836499	-0.071800
H	4.213800	-6.190099	-0.849899
H	4.952600	-5.319599	-3.155600
H	5.801700	-3.973799	-2.359600
H	4.589000	-3.639100	-3.611000
H	1.607900	-4.628399	-1.537600
H	2.454899	-5.701799	-2.672999
H	2.112399	-4.014900	-3.124799
H	1.868800	4.471499	1.785099
H	2.730299	5.309699	3.095499
H	2.352399	3.579800	3.241700
H	3.630500	5.227500	0.103000
H	5.347800	4.880799	0.409500
H	4.493600	6.082800	1.402799

H	5.214000	4.789500	3.507099
H	6.043000	3.594900	2.483400
H	4.815500	3.065900	3.655700
H	0.488099	-2.575799	5.017900
H	1.658600	-3.484600	6.002099
H	1.465000	-3.850200	4.273300
H	1.576400	-0.376400	5.719800
H	3.319800	-0.131800	5.475900
H	2.749000	-1.268500	6.714800
H	4.173800	-3.121000	5.638700
H	4.726300	-1.963000	4.410700
H	3.968299	-3.490699	3.914299
H	5.550699	-1.745600	1.674900
H	6.765300	-3.603400	2.774000
H	8.908200	-4.425299	1.821600
H	9.836499	-3.360099	-0.222900
H	8.640700	-1.490100	-1.301299
H	7.944600	0.676999	1.600300
H	9.259300	2.665800	2.225700
H	9.237900	4.686599	0.776299
H	7.876400	4.691200	-1.305400
H	6.547699	2.709500	-1.924100
H	2.546300	2.250500	-3.687499
H	2.762999	4.664200	-4.088700
H	1.750200	6.297800	-2.509500
H	0.524799	5.467800	-0.508599
H	0.324999	3.033700	-0.095500
H	-0.434099	-1.274399	-3.167999
H	-0.013899	-2.534999	-5.257500
H	2.196100	-2.373600	-6.389300
H	3.989100	-0.966300	-5.378600
H	3.583300	0.247299	-3.292900
